# Regiodivergent Gold-Catalyzed Rearrangement–Addition Reactions of Sulfenylated Propargylic Carboxylates with Indoles

**DOI:** 10.1021/acs.orglett.4c02853

**Published:** 2024-08-29

**Authors:** Nagnath Y. More, Paige A. Rist, Aniket Gupta, Paul W. Davies

**Affiliations:** School of Chemistry, University of Birmingham, Edgbaston, Birmingham B15 2TT, U.K.

## Abstract

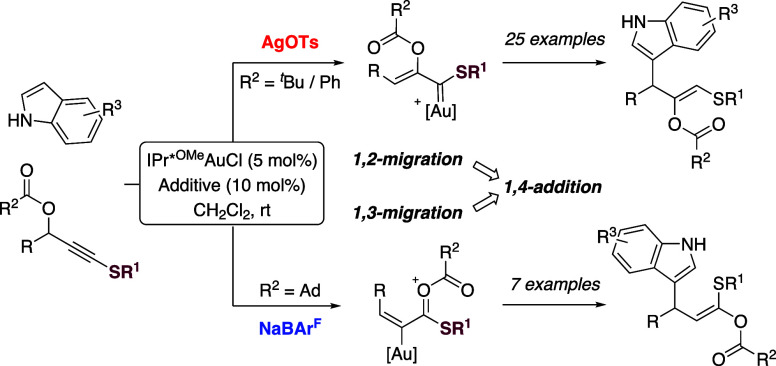

Sulfenylated propargylic carboxylates were introduced to investigate the influence of sulfur substitution in gold-catalyzed alkyne activation pathways. Regiodivergent gold-catalyzed rearrangement and indole capture reactions proceed under mild conditions to give functionalized indole products bearing sulfenylated (*Z*)-enol carboxylate motifs. Pathways involving both 1,2- and 1,3-carboxylate migrations are achieved selectively, with indole being added in a 1,4 relationship to the sulfenyl group in each case. High levels of selectivity are influenced by the catalyst system, counterion, and carboxylate group.

The use of heteroatom-substituted triple bonds has developed as a powerful strategy for addressing reactivity and regioselectivity challenges in gold catalysis, most notably with ynamides that underpin an array of novel transformations derived from highly α-selective additions dictated by the alkyne’s N-substituent.^1^ The analogous S-substituted alkynes, alkynyl thioethers, are much less well explored in this field despite the appeal of sulfur-containing molecules for synthetic, materials, electronic, and medical applications.^2,3^ Intriguingly, outcomes vary between α- and β-selective addition across the alkynyl thioethers in different gold-catalyzed intermolecular reactions.^4,5^ This observation raises the enticing prospect that selective regiodivergent outcomes might be possible from alkynyl thioethers.^5a^

Propargylic carboxylates have proven to be powerful motifs for the generation of molecular complexity. Diverse outcomes can be accessed under π-acid catalysis due to the dynamic relationship between 1,2- and 1,3-carboxylate rearrangement and propargylic activation pathways (Scheme 1a).^6^ Reactive species such as **A**–**C**, each with multiple electrophilic sites for subsequent reactions, can thus be accessed from a common precursor with outcomes influenced by the substitution pattern as well as the reactant and choice of reaction conditions. Our interest in sulfenyl substituents in gold catalysis^5,7^ led us to examine whether sulfenylated propargylic carboxylates could be viable substrates to explore any sulfenyl directing effect. Following alkyne activation, α- and β-addition to the alkynyl thioether component would map to 1,3- and 1,2-carboxylate migration of these putative substrates, respectively, potentially opening access to the unexplored sulfenylated organogold intermediates **S-A** and **S-B** (Scheme 1b).

**Scheme 1:**
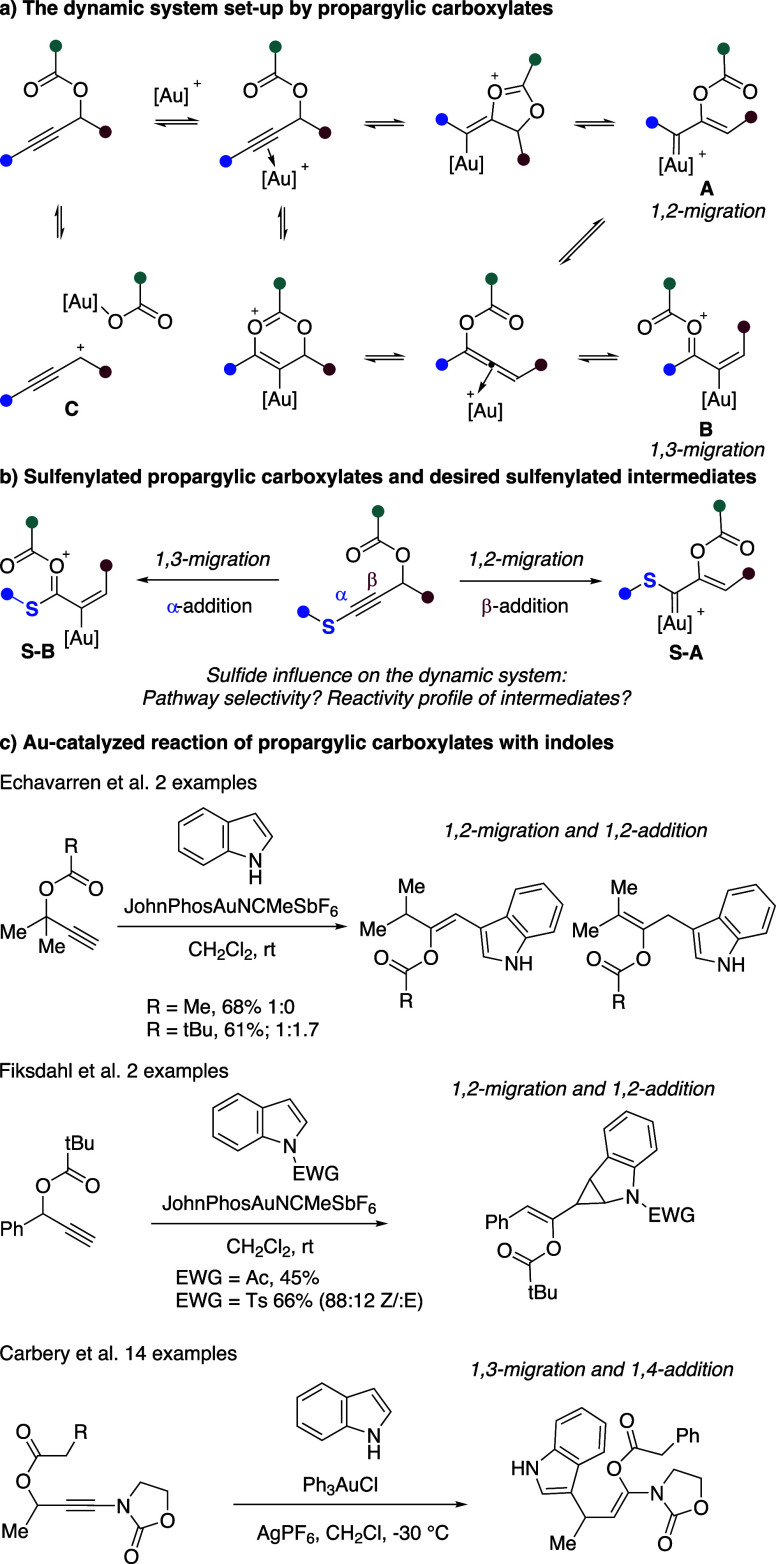
Reactivity from Propargylic Carboxylates

In this study, we examined the gold-catalyzed reactivity of sulfenylated propargylic carboxylates in reactions with indoles. Despite the importance of C3-modified indoles, intermolecular reactions with alkynes under gold catalysis are beset by challenges, including competing indole complexation and double addition pathways.^8^ The few studies using propargylic carboxylates alongside indoles show a limited scope. The groups of Echavarren and Fiksdahl have independently reported two examples each with terminal alkynes leading to products of 1,2-migration and then 1,2-addition (Scheme 1C).^9,10^ Carbery’s group reported reactions of ynamide-derived propargylic carboxylates, where the strongly donating effect of the nitrogen substituent favored α-selective addition and 1,3-carboxylate migration when using oxazolidinone substituents, but led to carboxylate elimination with more strongly donating sulfonamide substituents.^11^ Here we show that productive outcomes for both 1,2- and 1,3-carboxylate migration pathways can be realized from sulfenylated propargylic carboxylates, allowing selective access to regioisomeric functionalized indoles.

We explored the reactivity of 4-(methylthio)but-3-yn-2-yl pivalate **1a** with 1*H*-indole **2a** (Table 1). Two products were observed, **3aa** and **4aa**, consistent with 1,2- and 1,3-carboxylate migration to **S-A** and **S-B**, respectively. In both cases, 1,4-addition of the indole and deprotonation and protodeauration would follow. Single alkene diastereomers were formed in each case. The *Z* geometry in **3aa** is consistent with that in sulfenylated enol carboxylates accessed upon combining propargylic carboxylates with allyl sulfides.^12^

**Table 1:**
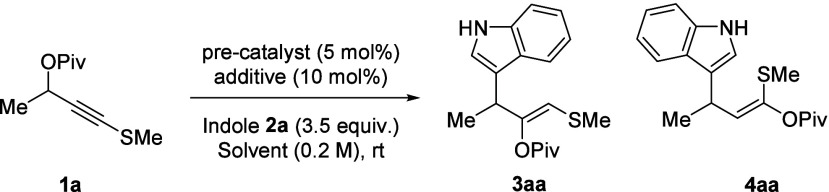
Influence of Reaction Conditions^a^

		yield (%)^b^		
entry	gold precatalyst/additive in CH_2_Cl_2_ unless specified	**1a**	**3aa**	**4aa**
1	KAuCl_4_	–	–	23
2	JohnPhosAuCl/AgOTs	41	22	8
3	IPrAuCl/AgOTs	13	60	5
4	IPr*^OMe^AuCl/AgOTs	–	87	<5
5	IPr*^OMe^Au(NCMe)SbF_6_	31	39	15
6	IPr*^OMe^AuCl/AgOTf	10	68	13
7	IPr*^OMe^AuCl/NaBAr^F^	8	35	50
8	AgOTs	89	–	–
9	IPr*^OMe^AuCl	>90	–	–
10	TsOH	–	–	–
11	IPr*^OMe^AuCl/AgOTs^c^	11	52	<5
12	IPr*^OMe^AuCl/AgOTs in toluene	–	–	–
13	IPr*^OMe^AuCl/AgOTs in CH_3_CN	9	62	12
14	IPr*^OMe^AuCl/AgOTs in CH_3_NO_2_	11	80	9

a Reactions were carried out using **1a** (1.0 equiv, 0.2 mmol), **2a** (3.5 equiv.), a precatalyst (5 mol %), and an additive (10 mol %) in a solvent (0.2 M) at room temperature for 48–60 h. Abbreviations: IPr, 1,3-bis(2,6-diisopropyl)imidazol-2-ylidene; JohnPhos, 2-(di-*tert*-butylphosphino)biphenyl; IPr*^OMe^, 1,3-bis(2,6-dibenzhydryl-4-methoxyphenyl)-2,3-dihydro-1*H*-imidazol-2-ylidene; OTf, trifluoromethanesulfonate; OTs, *p*-toluenesulfonate; BAr^F^, tetrakis[3,5-bis(trifluoromethyl)phenyl]borate.

b Yields were determined by ^1^H NMR spectroscopy using a known concentration of methyl 2,5-dinitrobenzoate.

c With 2 equiv. of **2a**.

Reaction with KAuCl_4_ gave a complex mixture with **4aa** in low yield (Table 1, entry 1). Using Au(I) complexes with AgOTs in CH_2_Cl_2_ at room temperature led to a mixture of **3aa** and **4aa**, with the highest conversion and selectivity for **3aa** being achieved with the sterically bulky NHC IPr*^OMe^ ligand (entries 2–4). The use of other counterions (entries 5–7) led to poorer selectivity and conversion; notably, the use of ^–^BAr^F^ favored compound **4aa** (vide infra). No reaction was observed in the absence of a precatalyst or a silver salt, and the use of TsOH led to decomposition (entries 8–10). A reasonable excess of indole was needed for clean reaction (entry 4 vs entry 11). The reaction was ineffective in toluene, but good yields of **3aa** were obtained in acetonitrile or nitromethane, albeit with a decreased selectivity (entries 12−14).

The wider scope for the selective formation of motif **3** was then explored (Scheme 2). Enantioenriched **(*****S*****)-1a** led to racemic product **3aa**, consistent with planar sulfanyl gold carbene **S-A**. Substitution was tolerated at all benzenoid positions of the indole (**3aa**–**3ae**). Using a benzoyl migrating group led to a yield that was slightly higher than that of pivaloyl (**3ba** vs **3aa**). The reaction also accommodates aryl fluorides (**3ad**), chlorides (**3ac**), bromides (**3ae** and **3af**), iodides (**3bg**), and a free hydroxy group (**3ah**). While an electron-donating alkoxy group (**3ai**) works well, a deactivated 5-carboxylic ester indole gave no product (**3aj**). Fused system **3ak** was also formed cleanly. Methyl groups at indole positions N1, C2, and C3 were all well tolerated (**3al–3an**, respectively). C2 functionalization occurred with 3-methylindole (**3am** and **3bm**), meaning that if a C3 attack did occur then the subsequent rearrangement is selective for a 1,2-migration of the functionalized allyl group (see the Supporting Information for analysis). Regioisomeric C3-functionalized **3an** is obtained using 2-methylindole.

**Scheme 2:**
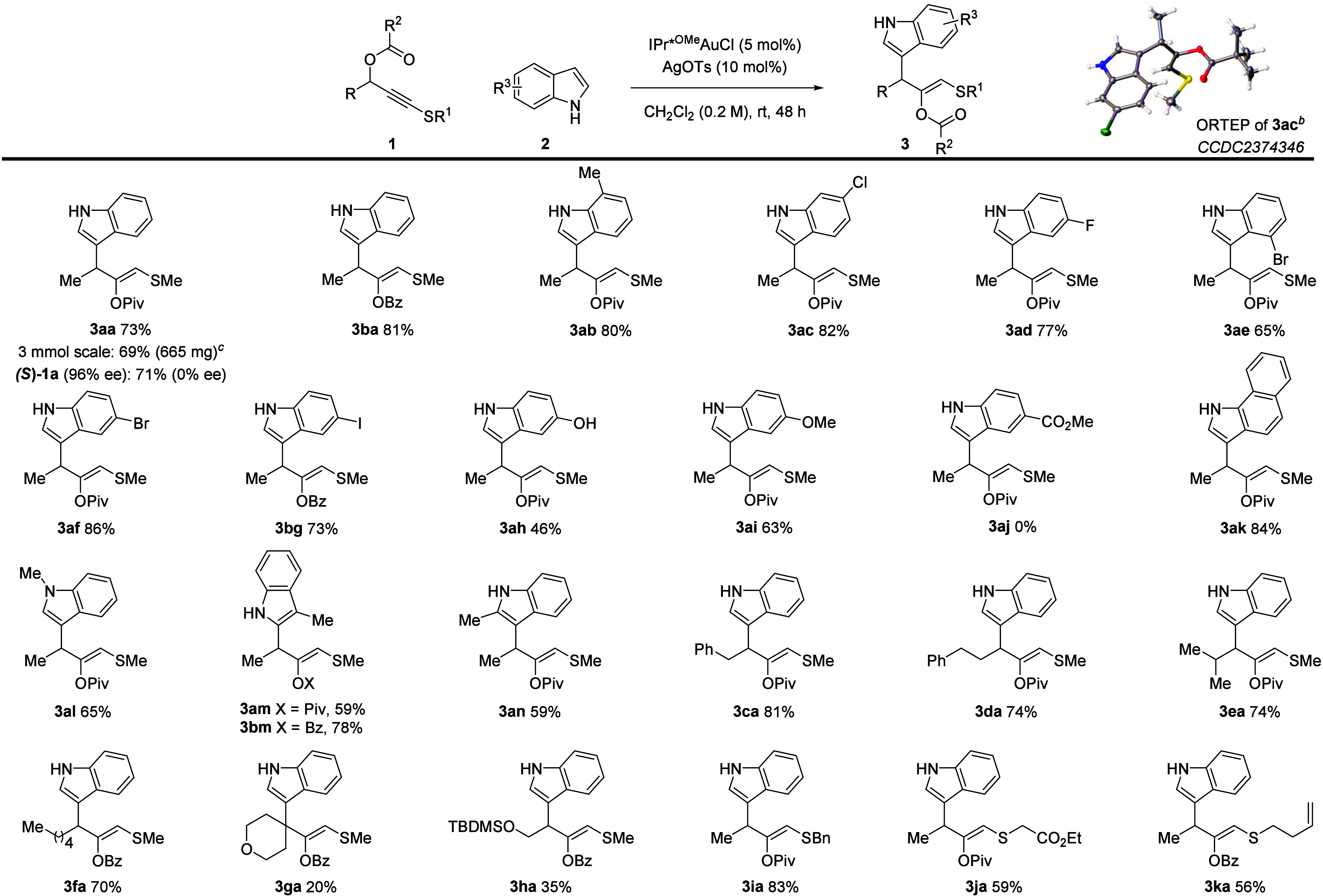
Scope of the 1,2-Carboxylate Migration and Indole Addition Process General conditions: **1** (1.0 equiv.), **2** (3.5 equiv.), IPr*^OMe^AuCl (5 mol %), and AgOTs (10 mol %) in CH_2_Cl_2_ (0.2 M). Yields refer to isolated products. At a 50% ellipsoid probability. Reaction performed on a 3.0 mmol scale.

While substrates with an aryl group on the propargylic position afforded complex reaction mixtures, more remote aryl substituents (**3ca** and **3da**) were well tolerated, with no reaction between the putative vinyl gold carbene and the phenyl group. Longer and larger substituents like butyl (**3fa**) and isopropyl (**3ea**) work well, while attempts to include a protected hydroxy group (**3ha**) and a quaternary center (**3ga**) were successful but low yielding. Products with more elaborate *S* substituents were also accessible, allowing for benzyl, alkene and ester inclusion (**3ia**, **3ja** and **3ka**).

Returning to the significant counterion effect seen in the initial survey when switching from the more basic ^–^OTs to ^–^BAr^F^ (Table 1, entry 7),^13^ we sought to favor regioisomeric product **4** via 1,3-carboxylate migration. Changing the substituent at the migrating carboxylate group proved to be significant (Scheme 3). Relative to the pivaloyl group, acetyl and benzoyl substituents favored **3** over **4**, but changing to the adamantyl system resulted in excellent conversion and good selectivity for **4** over **3**, indicating greater steric encumbrance around the carboxylate motif in the pathway to **S-A** (Scheme 1b). Applying these conditions led to compounds **4** in good yields tolerating changes to the indole (**4lb** and **4lf**) and the propargylic position (**4nb** and **4ob**), with a small amount of 1,2-migration outcome **3mb** observed alongside the formation of **4mb** using a larger *S*-benzyl substituent.

**Scheme 3:**
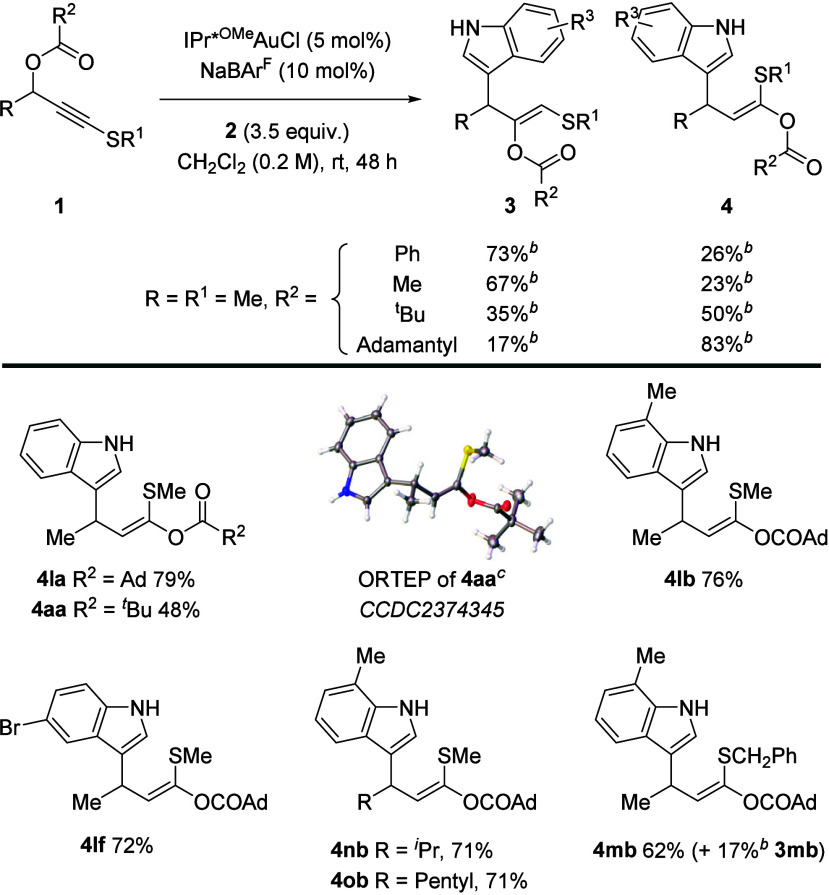
Selective Synthesis of Indole Products from a 1,3-Carboxylate Migration General conditions: **1** (1.0 equiv., 0.1 mmol), **2** (3.5 equiv., 0.35 mmol), IPr*^OMe^AuCl (5 mol %), and NaBAr^F^ (10 mol %) in CH_2_Cl_2_ (0.2 M). Yields of isolated products. Yields determined by ^1^H NMR spectroscopy of a product mixture with a known amount of methyl 2,5-dinitrobenzoate. At a 50% ellipsoid probability.

The reactivity of the functionalized indoles was then briefly explored (Scheme 4). Hydrolysis of the enol pivalate moieties occurs under mild conditions. α-Sulfenyl α′-(3-indolyl) ketone **5** was prepared from **3da** in good yield, illustrating the potential of the gold-mediated process as a selective alternative to strategies such as ketone indolylation for compounds with two enolizable positions. **4aa** undergoes enol carboxylate hydrolysis and substitution of the resulting thioester to yield methyl ester **6**. Oxidation of **3aa**, **3ad** and **3da** proceeds to give the novel enol sulfinyl functional group in **7** as a mix of diastereomers. Spontaneous rearrangement occurs in chloroform to afford 1,2-β-keto-α-oxy sulfide derivatives **8**, presumably by pivaloyl migration to the sulfoxide, elimination, and the capture at the sulfonium.

**Scheme 4:**
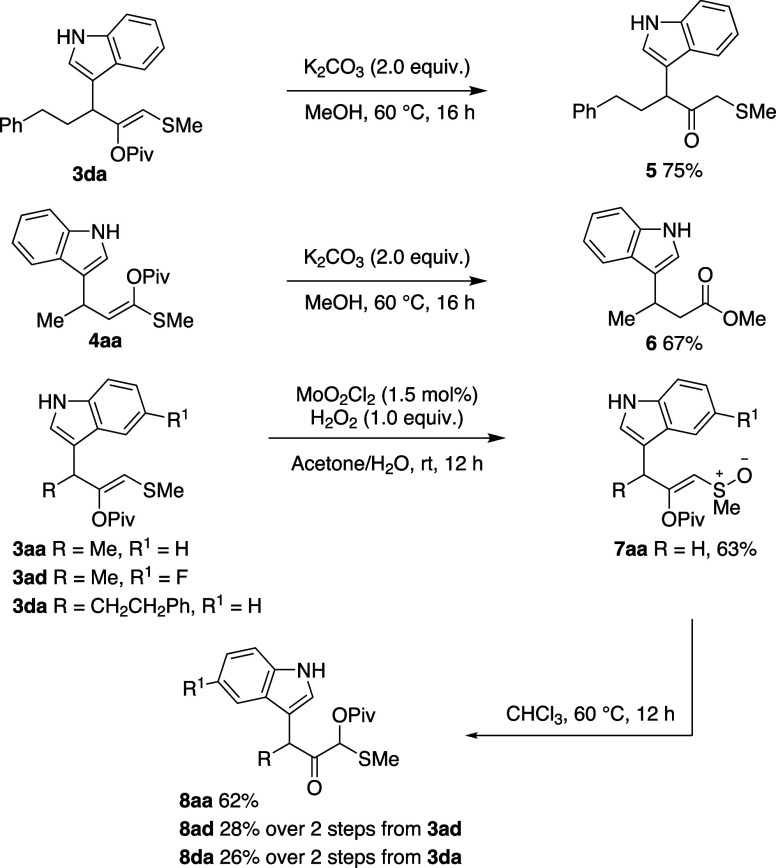
Transformation of the Funtionalised Indoles

The potential for reactions with other aromatic nucleophiles was briefly explored. 1,3,5-Trimethoxybenzene and 2,4-dimethyl pyrrole led to more complex mixtures. While *N*,*N*-dimethyl aniline was unreactive under standard conditions, 1,2-migration–arylation product **9** was obtained in good yield using IPrAu·NCCH_3_·SbF_6_ (Scheme 5), showing promise for the future development of varied transformations.

**Scheme 5:**
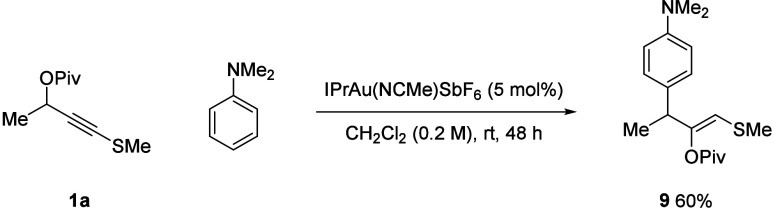
Extending the 1,2-Migration and Arylation Reaction with an Aniline Nucleophile

Here we have introduced sulfenylated propargylic carboxylates as tools for the regiodivergent synthesis under π-acid catalysis. Productive and selective pathways for both 1,2- and 1,3-carboxylate rearrangement are accessible under gold catalysis, as illustrated by the development of two rearrangement and indole addition reactions. The alkynyl thioether motif allows for tunable reaction outcomes with the choice of counterion and migrating groups being particularly influential here for pathway selectivity. Though counterions can play a multifaceted role in gold catalysis, the higher gold affinity^13c^ of ^–^OTs may be stabilizing the cationic character of gold required for the 1,2-migration pathway and gold carbene **S-A** versus the S/O-centered cations required for 1,3-migration through **S-B** (Scheme 1b). These regiodivergent syntheses proceed under mild reaction conditions from readily accessible building blocks for the selective formation of functionalized indole motifs.

## Data Availability

The data underlying this study are available in the published article and its Supporting Information.

## Supporting Information Available

Experimental details, compound characterization data, X-ray crystallographic data, and copies of NMR spectra (PDF)
