# Supplementary material for: Regiodivergent Gold-Catalyzed Rearrangement–Addition Reactions of Sulfenylated Propargylic Carboxylates with Indoles
Source: Org Lett. 2024 Aug 29;26(36):7713–7. doi: 10.1021/acs.orglett.4c02853 (PMC11406574; doi:10.1021/acs.orglett.4c02853)
Supplement: Supplementary file 1 — ol4c02853_si_001.pdf [file ol4c02853_si_001.pdf]

SUPPLEMENTARY INFORMATION

Regiodivergent gold-catalyzed rearrangement-addition reactions of sulfenylated propargylic carboxylates with indoles

Nagnath Y. More, Paige A. Rist, Aniket Gupta and Paul W. Davies*

School of Chemistry, University of Birmingham

Edgbaston, Birmingham, B15 2TT, UK.

E-mail: p.w.davies@bham.ac.uk

General Information 2

Experimental Procedures and Data 3

Preparation of Starting Materials 3

Catalysis Reactions 12

Transformation of catalysis products 31

HPLC traces for chirality transfer test 36

Crystallographic details 44

^{1}H NMR and ^{13}C NMR Spectra 47

References 108

General Information

All reagents were purchased from commercial sources and used without further purification. Unless stated otherwise, reactions were performed under an atmosphere of argon using standard Schlenk techniques. Dried glassware was heated using a heat-gun dried under reduced pressure, then cycles of argon backfill and evacuation were performed three times. All reactions were stirred using Teflon-coated magnetic stirrer bars. Dry ice/acetone baths were used to obtain -78 °C. Anhydrous THF, toluene, dichloromethane and acetonitrile were dried and degassed using a Pure-Solv MD solvent purification system; all other anhydrous solvents were dried over 4 Å molecular sieves. TLC analysis was undertaken using Merck silica gel 60 F254 (aluminium support) TLC plates; plates were visualized under UV light (254 nm), or through a vanillin or potassium permanganate stain. Manual flash column chromatography was undertaken using Sigma Aldrich 60 Å silica gel; automated flash column chromatography was undertaken using a Teledyne Isco Combiflash NextGen 100 instrument, using either Teledyne Isco Redisep RediSep® Normal-phase, RediSep Rf Gold® Normal-Phase, or InterChim Puriflash IR Silica flash columns. Mass spectra were obtained using Waters GCT Premier (EI), Waters LCT (ES), Waters Synapt (ES) or Bruker MicroTOF spectrometers, each fitted with TOF detector). The HRMS for most compounds reports the mass of neutral species when both the calculated and measured values are reported as neutrals within Waters MassLynx (V4.1 used). High resolution spectra used a lock-mass to adjust the calibrated mass scale. High resolution spectra used a lock-mass to adjust the calibrated mass scale. IR spectra were recorded on Perkin Elmer spectrum 100 FT-IR spectrometer. NMR spectra were obtained using a Bruker AVIII300, AVIII400, or Avance NEO 400 spectrometer; chemical shifts are stated in ppm and are measured relative to residual non-deuterated solvent. 1D ^{13}C NMR spectra were recorded using the UDEFT or PENDANT pulse sequences from the Bruker standard pulse program library. HSQC and HMBC spectra were recorded using the Bruker standard pulse program library. For ^{1}H NMR, multiplicities are denoted by the following notation: s (singlet), d (doublet), t (triplet), q (quadruplet), quin (quintuplet), br (broad), m (multiplet), app. (apparent). NMR spectra were processed using MestReNova 10.0.

Experimental Procedures and Data

Preparation of Starting Materials

Alcohols prepared according to the literature procedure.[1] IPr*^{OMe}AuCl prepared according to the literature procedure.[2]

General procedure for the synthesis of sulfenylated propargylic alcohols (GP1)

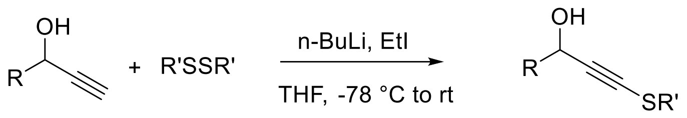

To a dry and degassed 3-necked round-bottom flask was charged the terminal alkyne (1.0 equiv.) and anhydrous THF (0.25 M). The solution was cooled to –78 °C, and *n*-butyllithium (2.5 M in hexanes, 2.0 equiv.) or LHMDS (1 M in THF, 2.0 equiv.) was added dropwise. The reaction was stirred for 1 h at –78 °C, at which point the corresponding dimethyl or diaryl sulfide (1.3 equiv.) was added dropwise (if a liquid) or in a single portion (if a solid) followed by addition of ethyl iodide (1.3 equiv.). The reaction was allowed to warm to rt and stirred overnight. The reaction was quenched by the addition of NH_4Cl (sat. aq.) and extracted with EtOAc. Organic extracts were dried over Na_2SO_4, and the solvent removed under reduced pressure affording the corresponding alcohols (**TA**).

General procedure for the synthesis of sulfenylated propargylic carboxylates (GP2)

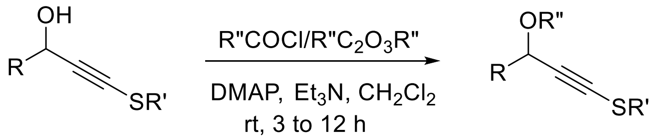

TA **1a-o**

The crude propargylic thioether **TA** (1.0 equiv.) was dissolved in CH_2Cl_2 (0.5 M), followed by addition of Et_3N (3.0 equiv.), DMAP (10 mol%), anhydride or alkoxyl chloride (1.5 equiv.) and the reaction mixture was stirred at rt for 12 h. On completion, $NaHCO_3$ (sat. aq.) was added until effervescence was no longer observed, before the solution was extracted with CH_2Cl_2 (3 × 2mL/mmol) and then dried with Na_2SO_4. The solution was concentrated under reduced pressure and was dry-loaded onto a minimum amount of silica. The product was purified by flash column chromatography to obtain the propargylic carboxylates **1**.

4-(Methylthio)but-3-yn-2-ol (TA1)

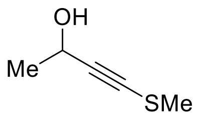

Prepared according to **GP1** using 3-butyne-2-ol (1.40 g, 20.0 mmol), *n*-butyllithium (16.0 mL, 2.5 M in hexanes, 40.0 mmol), ethyl iodide (2.10 mL, 26.0 mmol), dimethyl disulfide (2.31 mL, 26.0 mmol), and THF (80 mL); column chromatography (0 to 10% EtOAc in hexane) afforded **TA1** as a colorless liquid (1.81 g, 78%); **IR** (Neat): υ_{max}/cm^{-1} 3334 (br, OH stretch), 2982, 2930, 2182, 1421, 1370, 1313, 1123, 1075, 1048, 959; **^{1}H NMR** (400 MHz, $CDCl_3$); δ 4.60 (qd, *J* = 6.8, 5.3 Hz, 1H), 2.38 (s, 3H), 1.84 (d, *J* = 5.3 Hz, 1H), 1.46 (d, *J* = 6.8 Hz, 3H); **^{13}C NMR** (101 MHz, $CDCl_3$) δ 94.6, 76.3, 59.3, 24.4, 19.2; **HRMS** (EI+) m/z: $[M]^+$ Calcd for C_5H_8OS 116.0290; Found 116.0291.

4-(Methylthio)but-3-yn-2-yl pivalate 1a

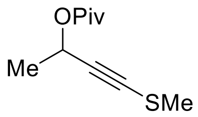

Prepared according to **GP2** using **TA1** (1.00 g, 9.0 mmol), pivaloyl chloride (1.60 mL, 13.5 mmol), triethylamine (1.90 mL, 13.5 mmol), DMAP (105 mg, 0.9 mmol), and CH_2Cl_2 (18 mL); column chromatography (5% EtOAc in hexane) afforded **1a** as a colorless liquid (1.45 g, 76%); **IR** (Neat): ν_{max}/cm^{-1} 2975, 2933, 2874, 2170, 1728 (C=O), 1277, 1151, 1068, 1039, 955, 855, 771; **^{1}H NMR** (400 MHz, $CDCl_3$) δ 5.51 (q, *J* = 6.8 Hz, 1H), 2.37 (s, 3H), 1.47 (d, *J* = 6.8 Hz, 3H), 1.20 (s, 9H); **^{13}C NMR** (101 MHz, $CDCl_3$) δ 177.5, 91.5, 77.0, 61.0, 38.8, 27.2, 21.3, 19.2; **HRMS** (ESI+) m/z: [M+Na] Calcd for $C_{10}H_{16}O_2SNa$ 223.0769; Found 223.0768.

4-(Methylthio)but-3-yn-2-yl benzoate (1b)

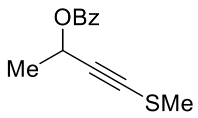

Prepared according to **GP2** using **TA1** (500 mg, 4.30 mmol), benzoyl chloride (749 μL, 6.45 mmol), triethylamine (903 μL, 6.46 mmol), DMAP (52.5 mg, 0.43 mmol) in CH_2Cl_2 (9 mL); column chromatography (5% EtOAc in hexane) afforded **1b** as a colorless liquid (750 mg, 75%); **IR** (Neat): ν_{max}/cm^{-1} 2989, 2931, 2874, 2171, 1717 (C=O), 1451, 1314, 1259, 1095, 1068, 708; **^{1}H NMR** (300 MHz, $CDCl_3$) δ 8.09 – 8.05 (m, 2H), 7.60 – 7.53 (m, 1H), 7.48 – 7.40 (m, 2H), 5.78 (q, *J* = 6.7 Hz, 1H), 2.39 (s, 3H), 1.62 (d, *J* = 6.7 Hz, 3H); **^{13}C NMR** (101 MHz, $CDCl_3$) δ 165.6, 133.2, 130.1, 129.8, 128.4, 91.3, 77.7, 61.8, 21.5, 19.1; **HRMS** (ESI+) m/z: [M+Na] Calcd for $C_{12}H_{12}O_2SNa$ 243.0456; Found 243.0462.

4-(Methylthio)-1-phenylbut-3-yn-2-ol (TA2)

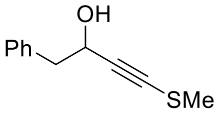

Prepared according to **GP1** using 1-phenylbut-3-yn-2-ol (600 mg, 4.10 mmol), dimethyl disulfide (474 µL, 5.33 mmol), ethyl iodide (428 µL, 5.33 mmol), *n* butyllithium (3.30 mL, 2.5 M in hexanes, 8.20 mmol), and THF (16 mL); column chromatography (15% EtOAc in hexane) afforded **TA2** as a colorless liquid (466 mg, 59%); **IR** (Neat): ν_{max}/cm^{-1} 3368 (br, OH stretch), 3028, 2926, 2183, 1454, 1380, 1313, 1079, 1028, 1048, 976,747, 697; **^{1}HNMR** (400 MHz, $CDCl_3$) δ 7.34 – 7.31 (m, 2H), 7.31– 7.27 (m, 3H), 4.69 – 4.65 (m, 1H), 3.06 – 2.96 (m, 2H), 2.35 (s, 3H), 1.83 (d, *J* = 5.9 Hz, 1H); **^{13}C NMR** (101 MHz, $CDCl_3$) δ 136.6, 130.0, 128.6, 127.1, 93.0, 78.2, 64.1, 44.2, 19.8; **HRMS** (ESI+) m/z: [M+H] Calcd for $C_{11}H_{13}OS$ 193.0687; Found 193.0683.

4-**(Methylthio)-1-phenylbut-3-yn-2-yl pivalate (1c)**

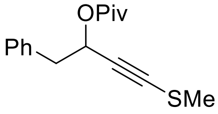

Prepared according to **GP2** using **TA2** (1.00 g, 5.5 mmol), pivaloyl chloride (1.00 mL, 8.25 mmol), triethylamine (1.10 mL, 8.25 mmol), DMAP (105 mg, 0.55 mmol), and CH_2Cl_2 (18 mL); column chromatography (10% EtOAc in hexane) afforded **1c** as a colorless amorphous solid (1.01 g, 69%); **IR** (Neat): ν_{max}/cm^{-1} 2969, 2934, 2175, 1723 (C=O) 1455, 1293, 1275, 1144, 1113, 1048, 940 746, 698; **^{1}H NMR** (400 MHz, $CDCl_3$) δ 7.31 – 7.27 (m, 2H), 7.25 – 7.22 (m, 3H), 5.61 (t, *J* = 6.8 Hz, 1H), 3.11 – 3.03 (m, 2H), 2.34 (s, 3H), 1.14 (s, 9H); **^{13}C NMR** (101 MHz, $CDCl_3$) δ 177.4, 136.3, 129.9, 128.4, 127.0, 90.2, 78.6, 65.2, 41.3, 38.8, 27.1, 19.2; **HRMS** (ESI+) m/z: [M+Na] Calcd for $C_{16}H_{20}O_2SNa$ 299.1082; Found 299.1081.

1-(Methylthio)-5-phenylpent-1-yn-3-ol (TA3)

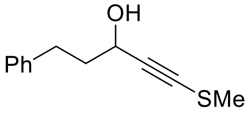

Prepared according to **GP1** using 5-phenylpent-1-yn-3-ol (2.50 g, 15.6 mmol), dimethyl disulfide (1.80 mL, 20.3 mmol), ethyl iodide (1.60 mL, 20.3 mmol), LiHMDS (31.2 mL, 1.0 M in hexanes, 31.2 mmol), and THF (62.0 mL, 0.25 M); column chromatography (10% EtOAc in hexane) afforded **TA3** as a colorless liquid (2.30 g, 72%); **IR** (Neat): ν_{max}/cm^{-1} 3293 (br, OH stretch), 3027, 2927, 2181, 1496, 1455, 1314, 1049, 1012, 748, 700; **^{1}H NMR** (400 MHz, $CDCl_3$) δ 7.31 – 7.27 (m, 2H), 7.22 – 7.17 (m, 3H), 4.49 – 4.42 (td, *J* = 6.5, 5.6 Hz, 1H), 2.79 (t, *J* = 7.8 Hz, 2H), 2.39 (s, 3H), 2.09 – 1.99 (m, 2H), 1.82 – 1.74 (m, 1H); **^{13}C NMR** (101 MHz, $CDCl_3$) δ 141.3, 128.64, 128.59, 126.2, 93.6,

77.6, 62.7, 39.3, 31.6, 19.3; **HRMS** (ESI+) m/z: [M+Na] Calcd for $C_{12}H_{14}OSNa$ 229.0663; Found 229.0661.

1-(Methylthio)-5-phenylpent-1-yn-3-yl pivalate 1d

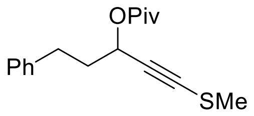

Prepared according to **GP2** using **TA2** (1.51 g, 5.7 mmol), pivaloyl chloride (1.30 mL, 8.55 mmol), triethylamine (1.20 mL, 8.55 mmol), DMAP (89.8 mg, 0.57 mmol), and CH_2Cl_2 (12 mL); column chromatography (5% EtOAc in hexane) afforded **1d** as a clear oil (1.30 g, 60%); **IR** (Neat): ν_{max}/cm^{-1} 2971, 2931, 2179, 1730 (C=O), 1455, 1276, 1140, 1080, 747, 698; **^{1}H NMR** (400 MHz, $CDCl_3$) δ 7.31 – 7.27 (m, 2H), 7.21 – 7.17 (m, 3H), 5.44 (t, *J* = 6.5 Hz, 1H), 2.80 – 2.72 (m, 2H), 2.38 (s, 3H), 2.15 – 2.02 (m, 2H), 1.22 (s, 9H); **^{13}C NMR** (101 MHz, $CDCl_3$) δ 177.5, 141.0, 128.7, 128.5, 126.3, 90.4, 78.1, 64.2, 38.9, 36.5, 31.5, 27.2, 19.3; **HRMS** (ES+) m/z: [M+Na] Calcd for $C_{17}H_{22}O_2SNa$ 313.1238; Found 313.1240.

4-Methyl-1-(methylthio)pent-1-yn-3-yl pivalate (1e)

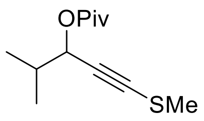

Prepared according to **GP2** using 4-methyl-1-(methylthio)pent-1-yn-3-ol (280 mg, 1.99 mmol), pivaloyl chloride (368 μL, 2.98 mmol), triethylamine (414 μL, 2.99 mmol), DMAP (23.7 mg, 0.20 mmol) and CH_2Cl_2 (4 mL); column chromatography (5% EtOAc in hexane) afforded **1e** as a clear oil (374 mg, 84%); **IR** (Neat): ν_{max}/cm^{-1} 2968, 2932, 2186, 1730 (C=O), 1479, 1460, 1280, 1154, 1136, 975; **^{1}H NMR** (400 MHz, $CDCl_3$) δ 5.27 – 5.25 (m, 1H), 2.37 (s, 3H), 2.04 – 1.95 (m, 1H), 1.21 (s, 9H), 1.00 (d, *J* = 6.3 Hz, 3H), 0.99 (d, *J* = 6.3 Hz, 3H); **^{13}C NMR** (101 MHz, $CDCl_3$) δ 177.6, 89.6, 77.8, 69.6, 39.0, 32.8, 27.2, 19.3, 18.3, 18.0; **HRMS** (EI+) m/z: [M] Calcd for $C_{12}H_{20}O_2S$ 228.1184; Found 228.1174.

1-(Methylthio)oct-1-yn-3-yl benzoate (1f)

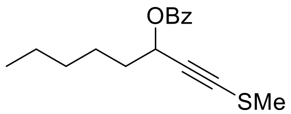

Prepared according to **GP2** using 1-(methylthio)oct-1-yn-3-ol (1.30 g, 7.55 mmol), benzoyl chloride (1.31 mL, 11.3 mmol), triethylamine (1.60 mL, 11.3 mmol), DMAP (92.1 mg, 0.76 mmol) in CH_2Cl_2 (15 mL); column chromatography (5% EtOAc in hexane) afforded colorless liquid **1f** (1.50 g, 69%); **IR** (Neat): ν_{max}/cm^{-1} 2954, 2929, 2860, 2178, 1718 (C=O), 1451, 1260, 1095, 1068, 1025, 709, 686; **^{1}H NMR** (400-MHz, $CDCl_3$) δ 8.08 – 8.05 (m, 2H), 7.59 – 7.55 (m, 1H), 7.47 – 7.43 (m, 2H), 5.70 (t, *J* = 6.6 Hz, 1H), 2.38 (s, 3H), 1.93 – 1.87 (m, 2H), 1.55 – 1.49 (m, 2H), 1.36 – 1.32 (m,

4H), 0.92 – 0.88 (m, 3H); **^{13}C NMR** (101 MHz, $CDCl_3$) δ 165.8, 133.3, 130.2, 129.9, 128.5, 90.7, 78.2, 65.6, 35.0, 31.5, 25.0, 22.6, 19.3, 14.1; **HRMS** (ESI+): m/z [M+Na] Calcd for $C_{16}H_{20}O_2SNa$ 299.1082; Found 299.1076.

4-((Methylthio)ethynyl)tetrahydro-2H-pyran-4-ol (TA4)

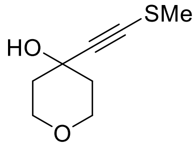

Prepared according to **GP1** using 4-ethynyltetrahydro-2H-pyran-4-ol (590 mg, 4.68 mmol), *n*-butyllithium (3.80 mL, 2.5 M in hexanes, 9.40 mmol), dimethyl disulfide (542 μL, 6.11 mmol), ethyl iodide (491 μL, 6.11 mmol), and THF (19 mL); column chromatography (30% EtOAc in hexane) afforded **TA4** as a colorless liquid (600 mg, 60%); **IR** (Neat): ν_{max}/cm^{-1} 3367 (br, OH stretch), 2956, 2861, 1466, 1424, 1233, 1093, 989; **^{1}H NMR** (400 MHz, $CDCl_3$) δ 3.91 – 3.86 (m, 2H), 3.67 – 3.61 (m, 2H), 2.39 (s, 3H), 2.15 (s, 1H), 1.95 – 1.90 (m, 2H), 1.83 – 1.77 (m, 2H); **^{13}C NMR** (101 MHz, $CDCl_3$) δ 94.8, 66.8, 65.0, 40.1, 39.9, 19.4; **HRMS** (Cl-) m/z [M-CH_3]$^-$ Calcd for $C_7H_9O_2S$ 157.0329; Found 157.0330.

4-((Methylthio)ethynyl)tetrahydro-2H-pyran-4-yl benzoate 1g

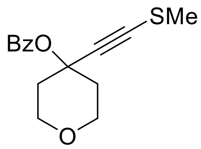

Prepared according to **GP2** using (**TA4)** (300 mg, 1.76 mmol), benzoyl chloride (306 μL, 2.64 mmol), triethylamine (368 μL, 2.64 mmol), DMAP (21.5 mg, 0.18 mmol) in CH_2Cl_2 (3.5 mL); column chromatography (20% EtOAc in hexane) afforded **1g** (300 mg, 64%) as a colorless liquid; **IR** (Neat): ν_{max}/cm^{-1} 2961, 2858, 2178, 1719 (C=O), 1450, 1268, 1159, 1091, 993, 698; **^{1}H NMR** (400 MHz, $CDCl_3$) δ 8.04 – 8.02 (m, 2H), 7.57 (tt, *J* = 7.1, 1.2 Hz, 1H), 7.43 (t, *J* = 7.6 Hz, 2H), 3.92 – 3.87 (m, 2H), 3.83 – 3.77 (m, 2H), 2.41 (s, 3H), 2.37 – 2.33 (m, 1H), 2.34 – 2.32 (m, 1H), 2.23 – 2.18 (m, 2H); **^{13}C NMR** (101 MHz, $CDCl_3$) δ 164.7, 133.1, 130.8, 129.8, 128.5, 91.4, 77.4, 73.8, 64.5, 37.7, 19.5; **HRMS** (EI+): m/z [M]$^+$ Calcd for $C_{15}H_{16}O_3S$ 276.0815; Found 276.0821.

1-((*tert*-Butyldimethylsilyl)oxy)-4-(methylthio)but-3-yn-2-ol (TA5)

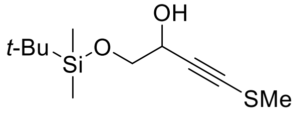

Prepared according to **GP1** using 1-((tert-butyldimethylsilyl)oxy)-4-(methylthio)but-3-yn-2-ol (230 mg, 1.15 mmol), *n*-butyllithium (0.92 mL, 2.5 M in hexanes, 2.30 mmol), dimethyldisulfide (132 μL, 1.5 mmol), ethyl iodide (120 μL, 1.50 mmol) and THF (4.6 mL); column chromatography (5% EtOAc in hexane) afforded **TA5** as a colorless liquid (200 mg, 57%); **IR** (Neat): νmax/cm^{-1} 3396 (br, O-H), 2953, 2928, 2857, 2176, 1471, 1252, 1108, 833, 770; **^{1}H NMR** (400 MHz, $CDCl_3$) δ

4.49 – 4.46 (m, 1H), 3.77 (dd, *J* = 10.0, 3.8 Hz, 1H), 3.64 (dd, *J* = 10.0, 7.1 Hz, 1H), 2.60 (d, *J* = 4.3 Hz, 1H), 2.38 (s, 3H), 0.91 (s, 9H), 0.9 (d, *J* = 1.6 Hz, 6H); **^{13}C NMR** (101 MHz, $CDCl_3$) δ 90.7, 67.0, 64.1, 26.0, 19.1, 18.5; **HRMS** (ESI+): m/z [M+Na] Calcd for $C_{11}H_{22}O_2SSiNa$ 269.1007; Found 269.1006.

1-((*tert*-Butyldimethylsilyl)oxy)-4-(methylthio)but-3-yn-2-yl benzoate (1h)

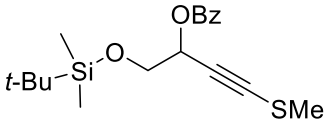

Prepared according to **GP2** using 1-((tert-butyldimethylsilyl)oxy)-4-(methylthio)but-3-yn-2-ol (**TA5)** (230 mg, 0.93 mmol), benzoyl chloride (162 μL, 1.4 mmol), triethylamine (195 μL, 1.4 mmol), DMAP (21.5 mg, 0.09 mmol) in CH_2Cl_2 (2 mL); column chromatography (5% EtOAc in hexane) afforded **1h** as a colorless liquid (200 mg, 57%); **IR** (Neat): ν_{max}/cm^{-1} 2971, 2931, 2179, 1730 (C=O), 1455, 1276, 1140, 1080, 747, 698; **^{1}H NMR** (400 MHz, $CDCl_3$) δ 8.10 – 8.05 (m, 2H), 7.60 – 7.54 (m, 1H), 7.44 (t, *J* = 7.7 Hz, 2H), 5.81 – 5.78 (m, 1H), 3.93 (d, *J* = 5.3 Hz, 2H), 2.38 (s, 3H), 0.86 (s, 9H), 0.08 (s, 3H), 0.05 (s, 3H); **^{13}C NMR** (101 MHz, $CDCl_3$) δ 165.8, 133.2, 130.1, 130.0, 128.5, 88.1, 79.2, 66.4, 65.1, 25.9, 19.1, 18.4, 5.2; **HRMS** (ESI+): m/z [M+Na] Calcd for $C_{18}H_{26}O_3SSiNa$ 373.1270; Found 373.1278.

4-(Benzylthio)but-3-yn-2-ol (TA6)

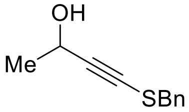

Prepared according to **GP1** using 3-butyne-2-ol (700 mg, 10.0 mmol), *n* butyllithium (8.00 mL, 2.5 M in hexanes, 20.0 mmol), dibenzyl disulfide (3.2 g, 13.0 mmol), ethyl iodide (1.03 mL, 13.0 mmol) and THF (40 mL); column chromatography (10% EtOAc in hexane) afforded 4-(benzylthio)but-3-yn-2-ol (**TA6)** as a colorless liquid (1.44 g, 75%); **IR** (Neat): ν_{max}/cm^{-1} 3369 (br, OH stretch), 2926, 2175, 1602, 1495, 1454, 1048, 1028, 909, 746, 695; ^{1}H NMR (400 MHz, $CDCl_3$) δ 7.37 – 7.28 (m, 5H), 4.59 – 4.53 (m, 1H), 3.92 (s, 2H), 1.70 (d, *J* = 4.8 Hz, 1H), 1.41 (d, *J* = 6.6 Hz, 3H); ^{13}C NMR (101 MHz, CDCl3) δ 136.6, 129.3, 128.7, 127.9, 97.4, 74.5, 59.3, 40.2, 24.3; **HRMS** (ASAP+) m/z: [M+H] Calcd for $C_{11}H_{13}OS$ 193.0687; Found 193.0685.

4-(Benzylthio)but-3-yn-2-yl pivalate (1i)

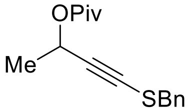

Prepared according to **GP2** using 4-(benzylthio)but-3-yn-2-ol **(TA6)** (250 mg, 1.30 mmol), pivaloyl chloride (240 μL, 1.95 mmol), triethylamine (271 μL, 1.95 mmol), DMAP (15.9 mg, 0.13 mmol) and CH_2Cl_2 (2.6 mL); column

chromatography (5% EtOAc in hexane) afforded **1i** as a colorless oil (249 mg, 69%); **IR** (Neat): ν_{max}/cm^{-1} 2972, 2935, 2178, 1727 (C=O), 1455, 1276, 1154, 1129, 1038, 955; **^{1}H NMR** (400 MHz, $CDCl_3$) δ 7.33 – 7.28 (m, 5H), 5.47 (q, *J* = 6.7 Hz, 1H), 3.95 – 3.88 (m, 2H), 1.42 (d, *J* = 6.7 Hz, 3H), 1.19 (s, 9H); **^{13}C NMR** (101 MHz, $CDCl_3$) δ 177.5, 136.7, 129.2, 128.7, 127.9, 94.3, 75.3, 61.0, 40.2, 38.8, 27.2, 21.2; **HRMS** (ESI+): m/z [M+Na] Calcd for $C_{16}H_{20}O_2SNa$ 299.1082; Found 299.1086.

4-((2-Ethoxy-2-oxoethyl)thio)but-3-yn-2-yl pivalate (1j)

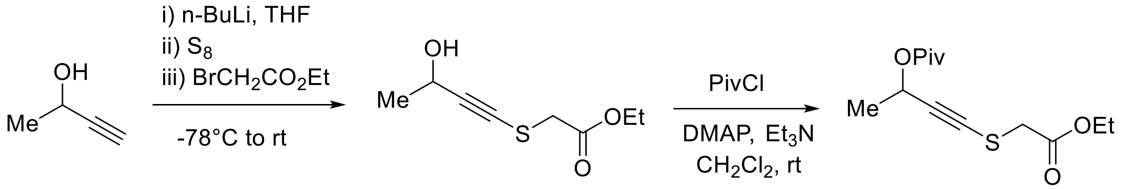

Prepared using a modified literature method:[3] To a dry and degassed three-necked round-bottom flask was charged but-3-yn-2-ol (1.40 g, 20.0 mmol) and anhydrous THF (80 mL). The resulting solution was cooled to –78 °C, and *n*-BuLi (16.0 mL, 2.5 M in hexanes, 22.0 mmol) was added dropwise. After stirring for 1 h at –78 °C, S_8 (832 mg, 26.0 mmol) was added, and the reaction mixture was allowed to warm to 0 °C, whereupon it was stirred for 2 h. Ethyl bromoacetate (2.22 mL, 20.0 mmol) was added dropwise, and the reaction mixture stirred overnight at rt. The reaction was quenched with NH_4Cl (sat. aq.) and extracted with Et_2O. Ethereal extracts were washed with brine and dried over Na_2SO_4. The solvent was removed *in vacuo*, and the resulting residue in column chromatography (40% EtOAc in hexane) to give ethyl 2-((3-hydroxybut-1-yn-1-yl)thio)acetate as a colorless liquid (1.10 g, 30%); **IR** (Neat): ν_{max}/cm^{-1} 3432 (br, OH), 2982, 2932, 2183, 1730 (C=O), 1368, 1269, 1122, 1024, 959, 861; **^{1}H NMR** (400 MHz, $CDCl_3$) δ 4.58 (q, *J* = 6.6 Hz, 1H), 4.22 (q, *J* = 7.2 Hz, 2H), 3.45 (s, 2H), 1.43 (d, *J* = 6.6 Hz, 3H), 1.29 (t, *J* = 7.1 Hz, 3H); **^{13}C NMR** (101 MHz, $CDCl_3$) δ 168.2, 97.2, 72.7, 61.9, 59.1, 37.4, 24.0, 14.2; **HRMS** (CI+) m/z: [M+Na] Calcd for $C_8H_{12}O_3SNa$ 211.0405; Found 211.0406.

Ethyl 2-((3-hydroxybut-1-yn-1-yl)thio)acetate (230 mg, 1.22 mmol) was used in **GP2** with pivaloyl chloride (225 μL, 1.83 mmol), triethylamine (255 μL, 1.83 mmol), DMAP (14.9 mg, 0.12 mmol) and CH_2Cl_2 (2.5 mL); column chromatography (20% EtOAc in hexane) afforded **1j** as a colorless liquid (200 mg, 57%, inseparable impurities were seen by NMR); **IR** (Neat): ν_{max}/cm^{-1} 2980, 2178, 1728 (C=O), 1479, 1276, 1154, 1129, 1052, 957; **^{1}H NMR** (400 MHz,

$CDCl_3$) δ 5.50 (q, *J* = 6.8 Hz, 1H), 4.26 – 4.21 (q, *J* = 7.1 Hz, 2H), 3.48 (s, 2H), 1.47 (d, *J* = 6.8 Hz, 3H), 1.30 (t, *J* = 7.1 Hz, 3H), 1.20 (s, 9H); **^{13}C NMR** (101 MHz, $CDCl_3$) δ 177.3, 168.1, 94.0, 73.5, 61.9, 60.7, 38.6, 37.4, 27.0, 21.0, 14.2; **HRMS** (CI+) m/z: [M+Na] Calcd for $C_{13}H_{20}O_4SNa$ 295.0980; Found 295.0978.

4-(But-3-en-1-ylthio)but-3-yn-2-yl pivalate (1k)

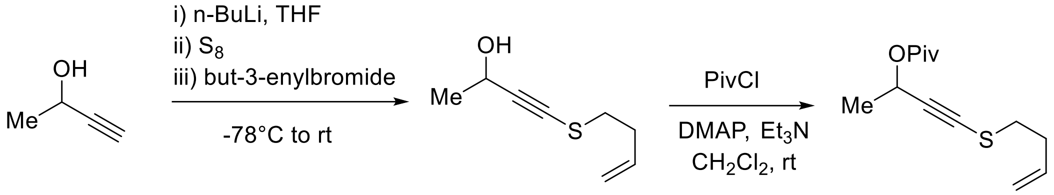

Prepared using a modified literature method:[3] To a dry and degassed three-necked round-bottom flask was charged but-3-yn-2-ol (1.40 g, 20.0 mmol) and anhydrous THF (80 mL). The resulting solution was cooled to -78 °C, and *n*-BuLi (16.0 mL, 2.5 M in hexanes, 22.0 mmol) was added dropwise. After stirring for 1 h at –78 °C, S_8 (832 mg, 26.0 mmol) was added, and the reaction mixture was allowed to warm to 0 °C, whereupon it was stirred for 2 h. 4-Bromo-1-butene (2.64 mL, 20.0 mmol) was added dropwise, and the reaction mixture stirred overnight at rt. The reaction was quenched with NH_4Cl (sat. aq.) and extracted with Et_2O. Ethereal extracts were washed with brine and dried over Na_2SO_4. The solvent was removed *in vacuo* to give 4-(but-3-en-1-ylthio)but-3-yn-2-ol (1.00 g) as colorless liquid. The crude material was used for the next step without further purification. **1k** was prepared according to **GP2** using 4-(but-3-en-1-ylthio)but-3-yn-2-ol (1.00 g, 6.50 mmol) and pivaloyl chloride; column chromatography (10% EtOAc in hexane) afforded **1k** (800 mg, 52%, inseparable minor impurities were seen by ^{1}H NMR) as a yellow oil; **IR** (Neat): ν_{max}/cm^{-1} 2980, 2178, 1728 (C=O), 1479, 1276, 1154, 1129, 1052, 957; **^{1}H NMR** (400 MHz, $CDCl_3$) δ 5.89 – 5.75 (m, 1H), 5.51 (q, *J* = 6.8 Hz, 1H), 5.11 – 5.06 (m, 2H), 2.81 – 2.69 (m, 2H), 2.47 (q, *J* = 7.3 Hz, 2H), 1.48 (d, *J* = 6.8 Hz, 3H), 1.20 (s, 9H); **^{13}C NMR** (101 MHz, $CDCl_3$) δ 177.5, 135.7, 117.0, 93.0, 75.2, 61.1, 38.8, 34.6, 33.4, 27.2, 21.3; **HRMS** (CI+) m/z: [M+H] Calcd for $C_{13}H_{21}O_2S$ 241.1262; Found 241.1268.

4-(Methylthio)but-3-yn-2-yl (3r,5r,7r)-adamantane-1-carboxylate (1l)

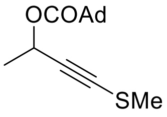

Prepared according to **GP2** using 4-(methylthio)but-3-yn-2-ol (**TA1**) (500 mg, 4.30 mmol), 1-adamantanecarbonyl chloride (939 mg, 4.73 mmol), triethylamine

(898 μL, 6.45 mmol), DMAP (87.5 mg, 0.43 mmol) and CH_2Cl_2 (9 mL); column chromatography (5% EtOAc in hexane) afforded **1l** as a colorless liquid (420 mg, 60%); **IR** (Neat): ν_{max}/cm^{-1} 2905, 2851, 2183, 1725 (C=O), 1452, 1222, 1067, 1036, 953, 696; **^{1}H NMR** (400 MHz, $CDCl_3$) δ 5.51 (q, *J* = 6.8 Hz, 1H), 2.35 (s, 3H), 2.00 – 1.99 (m, 3H), 1.88 (d, *J* = 3.3 Hz, 6H), 1.75 –1.68 (m, 6H), 1.44 (d, *J* = 6.8 Hz, 3H); **^{13}C NMR** (101 MHz, $CDCl_3$) δ 176.6, 91.6, 76.9, 60.7, 40.7, 38.8, 36.6, 28.1, 21.4, 19.2; **HRMS** (ESI-TOF): m/z [M+Na] Calcd for $C_{16}H_{22}O_2SNa$ 301.1238; Found 301.1241.

4-(Benzylthio)but-3-yn-2-yl (3r,5r,7r)-adamantane-1-carboxylate (1m)

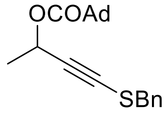

Prepared according to **GP2** using **TA6** (388 mg, 2.02 mmol), 1-adamantanecarbonyl chloride (441 mg, 2.22 mmol), triethylamine (421 μL, 3.03 mmol), DMAP (24.7 mg, 0.20 mmol) and CH_2Cl_2 (9 mL); column chromatography (5% EtOAc in hexane) afforded **1m** as a colorless liquid (420 mg, 60%); **IR** (Neat): ν_{max}/cm^{-1} 2904, 2851, 2178, 1725 (C=O), 1495, 1453, 1222, 1067, 1036, 953, 696; **^{1}H NMR** (400 MHz, $CDCl_3$) δ 7.34 – 7.26 (m, 5H), 5.48 (q, *J* = 6.7 Hz, 1H), 3.95 – 3.87 (m, 2H), 2.02 (s, 3H), 1.88 (d, *J* = 2.8 Hz, 6H), 1.75 – 1.68 (m, 6H), 1.41 (d, *J* = 6.7 Hz, 3H); **^{13}C NMR** (101 MHz, $CDCl_3$) δ 176.6, 136.7, 129.2, 128.7, 127.9, 94.4, 75.2, 60.7, 40.8, 40.2, 38.8, 36.6, 28.1, 21.3; **HRMS** (ESI-TOF): m/z [M+Na] Calcd for $C_{22}H_{26}O_2SNa$ 377.1551; Found 377.1552.

4-Methyl-1-(methylthio)pent-1-yn-3-yl (3r,5r,7r)-adamantane-1-carboxylate (1n)

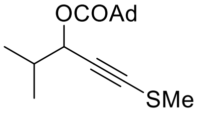

Prepared according to **GP2** using 4-methyl-1-(methylthio)pent-1-yn-3-ol (400 mg, 2.78 mmol), 1-adamantanecarbonyl chloride (606 mg, 3.00 mmol), triethylamine (580 μL, 4.17 mmol), DMAP (33.9 mg, 0.28 mmol) and CH_2Cl_2 (5.6 mL); column chromatography (5% EtOAc in hexane) afforded **1n** as a colorless liquid which was contaminated with residue from 1-adamantanecarbonyl chloride, 1:1); **IR** (Neat): ν_{max}/cm^{-1} 2905, 2851, 2185, 1727 (C=O), 1697, 1453, 1221, 1067, 969; **^{1}H NMR** (400 MHz, $CDCl_3$) δ 5.26 (d, *J* = 5.6 Hz, 1H), 2.36 (s, 3H), 2.02 – 1.98 (m, 4H), 1.89 (d, *J* = 3.1 Hz, 6H), 1.72 – 1.67 (m, 6H), 0.99 (d, *J* = 4.1 Hz, 3H), 0.97 (d, *J* = 4.1 Hz, 3H); **^{13}C NMR** (101 MHz, $CDCl_3$) δ 176.6, 89.8, 77.7, 69.3, 41.0, 38.9, 38.8, 36.7, 36.6, 32.9, 28.1, 28.0, 19.4, 18.4, 18.0; **HRMS** (ESI+): m/z [M+Na] Calcd for $C_{18}H_{26}O_2SNa$ 329.1551; Found 329.1551.

1-(Methylthio)oct-1-yn-3-yl (3r,5r,7r)-adamantane-1-carboxylate (1o)

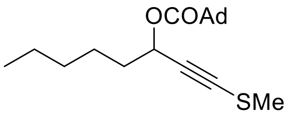

Prepared according to **GP2** using 1-(methylthio)oct-1-yn-3-ol (326 mg, 1.89 mmol), 1-adamantanecarbonyl chloride (414 mg, 2.08 mmol), triethylamine (394 µL, 2.80 mmol), DMAP (23.2 mg, 0.19 mmol), and CH_2Cl_2 (3.8 mL); column chromatography (100% hexane) afforded **1o** as a colorless liquid which was contaminated with residue from 1-adamantanecarbonyl chloride, 7:3); **IR** (Neat): ν_{max}/cm^{-1} 2954, 2929, 2178, 1718 (C=O), 1451, 1260, 1095, 1025, 709; **^{1}H NMR** (400 MHz, $CDCl_3$) δ 5.43 (t, *J* = 8.0 Hz, 1H), 2.37 (s, 3H), 2.03 – 2.00 (m, 3H), 1.90 (d, *J* = 4.0 Hz, 6H), 1.77 – 1.74 (m, 6H), 1.69 – 1.67 (m, 2H), 1.43 – 1.38 (m, 2H), 1.33 – 1.28 (m, 4H), 0.91 – 0.87 (m, 3H); **^{13}C NMR** (101 MHz, $CDCl_3$) δ 176.7, 91.0, 64.4, 38.8, 36.6, 34.8, 31.4, 28.1, 27.8, 24.9, 22.6, 19.3, 14.1; **HRMS** (ESI+) m/z: [M+Na] Calcd for $C_{20}H_{30}O_2SNa$ 357.1864; Found 357.1870.

Catalysis Reactions

General procedure for the catalysis reactions via 1, 2-carboxylate migration (GP3):

A mixture of propargyl carboxylates (1.0 equiv.) and indole (3.5 equiv.) in anhydrous CH_2Cl_2 (0.2 M) was prepared in a heat-gun dried Schlenk tube under argon, before IPr*^{OMe}AuCl (5 mol%) followed by AgOTs (10 mol%) was added. The solution was stirred at rt for 48 h. The reaction mixture was filtered through a pad of celite and the celite was washed with EtOAc (3 × ~1 mL/0.2 mmol). The solution was concentrated under reduced pressure and the product was purified by flash column chromatography.

3aa

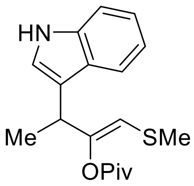

Prepared according to **GP3** using **1a** (40.0 mg, 0.20 mmol), indole **2a** (82.0 mg, 0.70 mmol), IPr*^{OMe}AuCl (11.8 mg, 0.01 mmol), AgOTs (5.6 mg, 0.02 mmol) in CH_2Cl_2 (1.0 mL); column chromatography (20% EtOAc in hexane) afforded **3aa** as a colorless amorphous solid (47.0 mg, 73%); **IR** (Neat): ν_{max}/cm^{-1} 3409 (br, NH stretch), 2924, 1744 (C=O), 1458, 1367, 1188, 1057, 737, 689; **^{1}H NMR** (300 MHz, $CDCl_3$) δ 8.03 (s, 1H), 7.75 (d, *J* = 7.9 Hz, 1H), 7.36 (d, *J* = 7.9 Hz, 1H), 7.22 – 7.16 (m, 1H), 7.14 – 7.09 (m, 1.2 Hz, 1H), 7.05 (s, 1H), 5.44 (d, *J* = 1.2 Hz, 1H), 3.98 (q, *J* = 7.1 Hz, 1H), 2.11 (s, 3H), 1.52 (d, *J* = 7.1 Hz, 3H), 1.26 (s, 9H); **^{13}C NMR** (101 MHz, $CDCl_3$) δ 176.1, 150.3, 136.5, 126.9, 122.2,

121.8, 120.2, 119.6, 117.5, 113.8, 111.1, 39.3, 35.3, 27.3, 18.6, 16.9; **HRMS** (ESI+) m/z: [M+Na] Calcd for $C_{18}H_{23}NO_2SNa$ 340.1347; Found 340.1354.

Larger scale reaction protocol

To the mixture of **1a** (601 mg, 3.00 mmol) and indole **2a** (1.23 g, 10.5 mmol) in anhydrous CH_2Cl_2 (15.0 mL) in a heat-gun dried Schlenk tube under argon, was added $IPr*^{OMe}AuCl$ (177 mg, 0.15 mmol) followed by AgOTs (84.5 mg, 0.3 mmol). The solution was stirred at rt for 48 h. The reaction mixture was filtered through a pad of celite and the celite was washed with EtOAc (~10 × 1 mL). The solution was concentrated under reduced pressure and the product was purified by flash column chromatography (eluted with 0 to 20% EtOAc in hexane) to obtain product **3aa** (665 mg, 69%).

1,4-Addition confirmed by the structure confirmation of 3aa

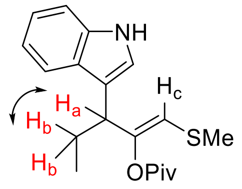

δ H_a = 5.42 , H_b = 1.52 , H_c = 5.42

In the **^{1}H NMR (NOESY)** spectrum, a clear doublet was observed for the methyl group H_b in the aliphatic region at δ = 1.52 and a quartet for the allylic proton H_a at δ = 3.96. In addition, there is strong NOE correlation between H_a (δ 3.96, q) with H_b (δ 1.52, s). There is weak NOE correlation between H_c (δ 5.42, d) and H_a (δ 3.96, q).

^1H-^1H NOESY (400 MHz, $CDCl_3$) for 3aa

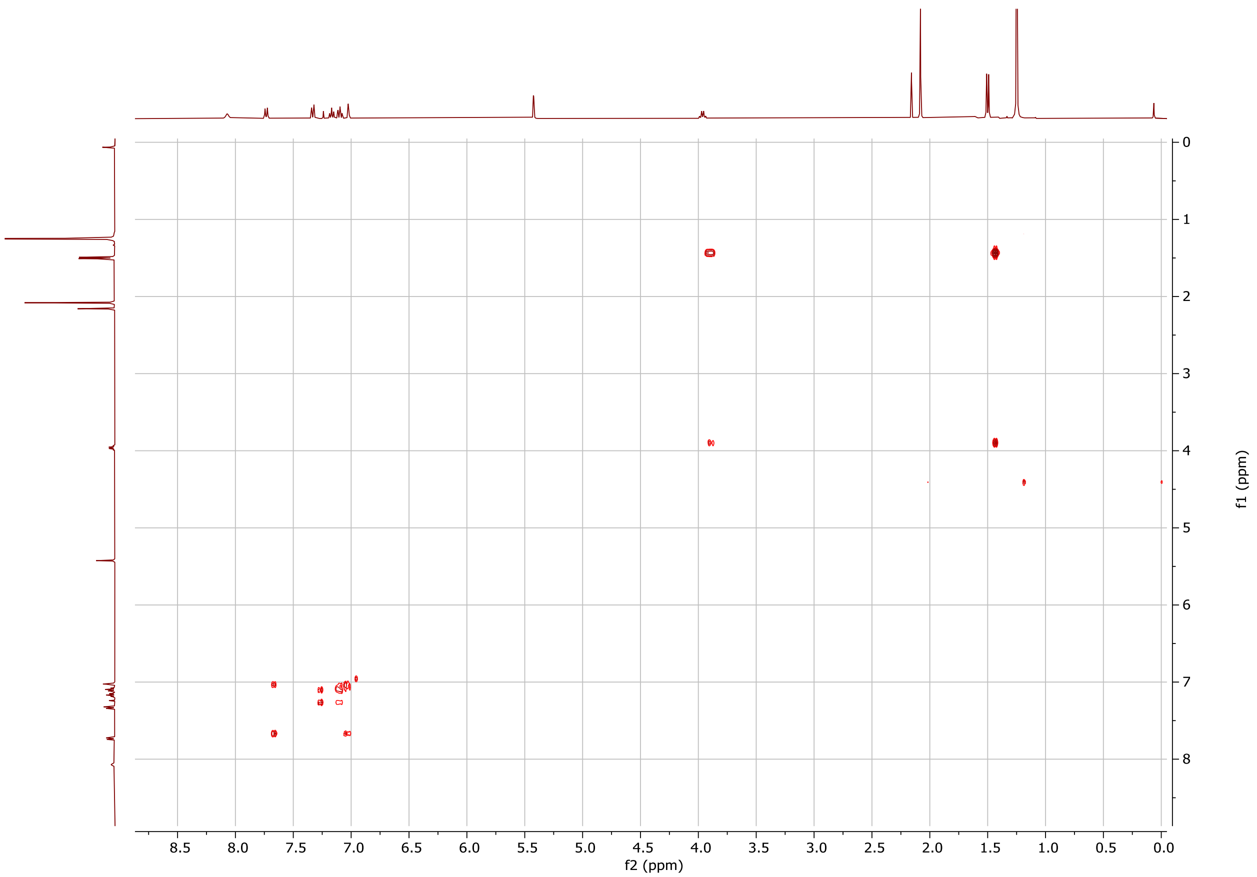

3ba

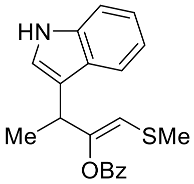

Prepared according to **GP3** using **1b** (44.0 mg, 0.20 mmol), indole **2a** (82.0 mg, 0.70 mmol), IPr*^{OMe}AuCl (11.8 mg, 0.01 mmol), AgOTs (5.6 mg, 0.02 mmol) in CH_2Cl_2 (1.0 mL); column chromatography (20% EtOAc in hexane) afforded **3ba** as a colorless oil (58.0 mg, 81%); **IR** (Neat): ν_{max}/cm^{-1} 3405 (br, NH stretch), 2970, 2921, 1723 (C=O), 1451, 1267, 1229, 1084, 1064, 1024, 741, 704; **^{1}H NMR** (400 MHz, $CDCl_3$) δ 8.14 – 8.10 (m, 2H), 8.09 (s, 1H), 7.82 (d, *J* = 7.9 Hz, 1H), 7.59 (tt, *J* = 7.7, 1.2 Hz, 1H), 7.46 (t, *J* = 7.9 Hz, 2H), 7.37 (d, *J* = 8.0 Hz, 1H), 7.23 – 7.18 (m, 1H), 7.16 – 7.12 (m, 1H), 7.09 (d, *J* = 2.3 Hz, 1H), 5.63 – 5.60 (m, 1H), 4.14 (q, *J* = 7.1 Hz, 1H), 2.13 (s, 3H), 1.61 (d, *J* = 7.1 Hz, 3H); **^{13}C NMR** (101 MHz, $CDCl_3$) δ 164.1, 150.5, 136.6, 133.5, 130.2, 129.6, 128.6, 126.8, 122.2, 121.9, 120.2, 119.6, 117.3, 114.2, 111.2, 35.7, 18.7, 16.9; **HRMS** (ESI+) m/z: [M+Na] Calcd for $C_{20}H_{23}N_2O_2S$ 355.1480; Found 355.1487.

3ab

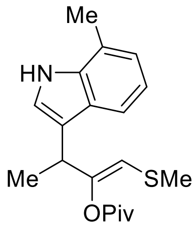

Prepared according to **GP3** using **1a** (40.0 mg, 0.20 mmol), **2b** (91.8 mg, 0.70 mmol), IPr*^{OMe}AuCl (11.8 mg, 0.01 mmol), AgOTs (5.6 mg, 0.02 mmol) in CH_2Cl_2 (1.0 mL); column chromatography (20% EtOAc in hexane) afforded **3ab** as a colorless liquid (53.0 mg, 80%); **IR** (Neat): ν_{max}/cm^{-1} 3404 (br, NH stretch), 2971, 1727 (C=O), 1478, 1279, 1116, 1043, 782, 747; **^{1}H NMR** (400 MHz, $CDCl_3$) δ 8.04 (s, 1H), 7.62 (d, *J* = 8.5 Hz, 1H), 7.06 (s, 1H), 7.04 (d, *J* = 4.8 Hz, 1H), 7.02 – 7.00 (m, 1H), 5.46 (d, *J* = 1.2 Hz, 1H), 4.02 – 3.96 (m, 1H), 2.50 (s, 3H), 2.10 (s, 3H), 1.52 (d, *J* = 7.1 Hz, 3H), 1.30 (s, 9H); **^{13}C NMR** (101 MHz, $CDCl_3$) δ 176.1, 150.3, 136.1, 126.4, 122.7, 121.5, 120.3, 119.8, 117.9, 117.8, 113.7, 39.3, 35.4, 27.3, 18.6, 16.8, 16.7; HRMS (ESI+) m/z: [M+NH_4] Calcd for $C_{19}H_{29}N_2O_2S$ 349.1950; Found 349.1953.

3ac

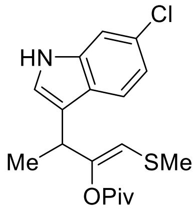

Prepared according to **GP3** using **1a** (40.0 mg, 0.20 mmol), **2c** (111.0 mg, 0.70 mmol), IPr*^{OMe}AuCl (11.8 mg, 0.01 mmol), AgOTs (5.6 mg, 0.02 mmol) in CH_2Cl_2 (1.0 mL); column chromatography (30% EtOAc in hexane) afforded **3ac** as a colorless solid (57.0 mg, 82%); **IR** (Neat): ν_{max}/cm^{-1} 3397 (br, NH stretch), 2971, 1727 (C=O), 1477, 1279, 1125, 972, 794, 747; **^{1}H NMR** (400 MHz, $CDCl_3$) δ 8.17 (s, 1H), 7.66 (d, *J* = 8.0 Hz, 1H), 7.33 (d, *J* = 2.0 Hz, 1H), 7.07 (dd, *J* = 8.0, 2.0 Hz, 1H), 7.01 (m, 1H), 5.40 (d, *J* = 2.0 Hz, 1H), 3.93 (q, *J* = 8.0 Hz, 1H), 2.10 (s, 3H), 1.50 (d, *J* = 8.0 Hz, 3H), 1.27 (s, 9H); **^{13}C NMR** (101 MHz, $CDCl_3$) δ 176.2, 149.9, 136.9, 128.1, 125.5, 122.5, 121.1, 120.3, 117.5, 114.0, 111.1, 39.3, 35.2, 27.3, 18.5, 16.8; **HRMS** (ESI+) m/z: [M+NH_4] Calcd for $C_{18}H_{26}ClN_2O_2S$ 369.1404; Found 369.1409.

3ad

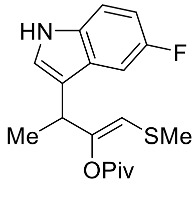

Prepared according to **GP3** using **1a** (40.0 mg, 0.20 mmol), **2d** (94.6 mg, 0.70 mmol), IPr*^{OMe}AuCl (11.8 mg, 0.01 mmol), AgOTs (5.6 mg, 0.02 mmol) in CH_2Cl_2 (1.0 mL); column chromatography (15% EtOAc in hexane) afforded **3ad** as a colorless amorphous solid (52.0 mg, 77%); **IR** (Neat): ν_{max}/cm^{-1} 3398 (br, NH stretch), 2971, 1728 (C=O), 1487, 1328, 1273, 1122, 932, 791; **^{1}H NMR** (400 MHz, $CDCl_3$) δ 8.14 (s, 1H), 7.38 (dd, *J* = 9.8, 2.5 Hz, 1H), 7.23 (dd, *J* = 9.0, 4.3 Hz, 1H), 7.05 (d, *J* = 2.4 Hz, 1H), 6.90 (td, *J* = 9.0, 2.5 Hz, 1H), 5.42 (d, *J* = 1.2 Hz, 1H), 3.89 (q, *J* = 7.1 Hz, 1H), 2.09 (s, 3H),

1.48 (d, J = 7.1 Hz, 3H), 1.24 (s, 9H); **^{13}C NMR** (101 MHz, $CDCl_3$) δ 176.1, 157.8 (d, $J_{C\text{-}F}$ = 234.0 Hz), 149.9, 133.0, 127.2 (d, $J_{C\text{-}F}$ = 9.5 Hz), 123.6, 117.5 (d, $J_{C\text{-}F}$ = 4.8 Hz), 113.9, 111.8 (d, $J_{C\text{-}F}$ = 9.5 Hz), 110.6 (d, $J_{C\text{-}F}$ = 27.6 Hz), 105.0 (d, $J_{C\text{-}F}$ = 27.6 Hz), 39.3, 35.3, 27.3, 18.5, 16.9; **HRMS** (ESI+) m/z: [M+Na] Calcd for $C_{18}H_{22}FNO_2SNa$ 358.1253; Found 358.1255.

3ae

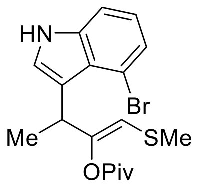

Prepared according to **GP3** using **1a** (40.0 mg, 0.20 mmol), **2e** (137 mg, 0.70 mmol), IPr*^{OMe}AuCl (11.8 mg, 0.01 mmol), AgOTs (5.6 mg, 0.02 mmol) in CH_2Cl_2 (1.0 mL); column chromatography (15% EtOAc in hexane) afforded **3ae** as a colorless solid (43.0 mg, 65%); **IR** (Neat): ν_{max}/cm^{-1} 3361 (br, NH stretch), 2971, 2872, 1725 (C=O), 1478, 1396, 1128, 910, 739; **^{1}H NMR** (400 MHz, $CDCl_3$) δ 8.44 (s, 1H), 7.28 (d, J = 7.8 Hz, 1H), 7.25 (d, J = 7.8 Hz, 1H), 7.11 (d, J = 1.9 Hz, 1H), 6.97 (app t, J = 7.8 Hz, 1H), 5.60 (d, J = 1.0 Hz, 1H), 4.75 (q, J = 7.1 Hz, 1H), 2.16 (s, 3H), 1.45 (d, J = 7.1 Hz, 3H), 1.19 (s, 9H); **^{13}C NMR** (101 MHz, $CDCl_3$) δ 175.6, 150.6, 137.5, 124.8, 124.4, 123.7, 122.7, 118.6, 114.1, 113.6, 110.8, 39.3, 34.8, 27.3, 20.4, 17.0; **HRMS** (ESI+) m/z: [M+Na] Calcd for $C_{18}H_{22}{}^{79}BrNO_2SNa$ 418.0452; Found 418.0451.

3af

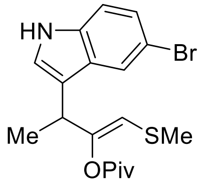

Prepared according to **GP3** using **1a** (40.0 mg, 0.20 mmol), **2f** (137 mg, 0.70 mmol), IPr*^{OMe}AuCl (11.8 mg, 0.01 mmol), AgOTs (5.6 mg, 0.02 mmol) in CH_2Cl_2 (1.0 mL); column chromatography (15% EtOAc in hexane) afforded **3af** as a colorless liquid (68.0 mg, 86% yield); **IR** (Neat): ν_{max}/cm^{-1} 3361 (br, NH stretch), 2971, 2872, 1725 (C=O), 1478, 1396, 1128, 910, 739; **^{1}H NMR** (400 MHz, $CDCl_3$) δ 8.31 (s, 1H), 7.90 (d, J = 2.0 Hz, 1H), 7.31 (dd, J = 8.4, 2.0 Hz, 1H), 7.27 (d, J = 8.4, 1H), 7.08 (d, J = 2.5 Hz, 1H), 5.54 (d, J = 1.3 Hz, 1H), 4.01 (q, J = 7.1 Hz, 1H), 2.19 (s, 3H), 1.55 (d, J = 7.1 Hz, 3H), 1.31 (s, 9H); **^{13}C NMR** (101 MHz, $CDCl_3$) δ 176.0, 149.8, 135.1, 128.5, 125.0, 123.1, 122.4, 117.0, 113.9, 112.8, 112.7, 39.3, 35.2, 27.3, 18.6, 16.9; **HRMS** (ESI+TOF): m/z [M+Na] Calcd for $C_{18}H_{22}{}^{79}BrNO_2SNa$ 418.0452; Found 418.0462.

3bg

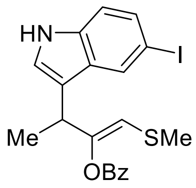

Prepared according to **GP3** using **1b** (46.6 mg, 0.20 mmol), **2g** (170 mg, 0.70 mmol), IPr*^{OMe}AuCl (11.8 mg, 0.01 mmol), AgOTs (5.6 mg, 0.02 mmol) in CH_2Cl_2 (1.0 mL); column chromatography (15% EtOAc in hexane) afforded **3bg** as a colorless amorphous solid (69.0 mg, 73%); **IR** (Neat): ν_{max}/cm^{-1} 3392 (br, NH stretch), 2970, 2921, 1721 (C=O), 1450, 1268, 1229, 1083, 1064, 793, 704; **^{1}H NMR** (400 MHz, $CDCl_3$) δ 8.20 (s, 1H), 8.11 – 8.06 (m, 3H), 7.62 – 7.56 (m, 1H), 7.48 – 7.40 (m, 3H), 7.12 (d, *J* = 8.5 Hz, 1H), 7.03 (d, *J* = 2.4 Hz, 1H), 5.64 (d, *J* = 1.2 Hz, 1H), 4.06 (q, *J* = 7.1 Hz, 1H), 2.16 (s, 3H), 1.57 (d, *J* = 7.1 Hz, 3H); **^{13}C NMR** (101 MHz, $CDCl_3$); δ 163.9, 150.1, 135.6, 133.5, 130.5, 130.2, 129.4, 129.3, 128.8, 128.6, 122.7, 116.8, 114.4, 113.3, 83.1, 35.6, 18.7, 17.0; **HRMS** (ESI+): m/z [M+Na] Calcd for $C_{20}H_{18}INO_2SNa$ 486.0001; Found 486.0008.

3ah

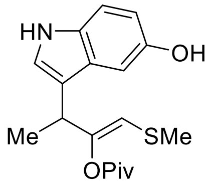

Prepared according to **GP3** using **1a** (40.0 mg, 0.20 mmol), **2h** (93.2 mg, 0.70 mmol), IPr*^{OMe}AuCl (11.8 mg, 0.01 mmol), AgOTs (5.6 mg, 0.02 mmol) in CH_2Cl_2 (1.0 mL); column chromatography (30% EtOAc in hexane) afforded as an off white solid (30.0 mg, 46% yield); **IR** (Neat): ν_{max}/cm^{-1} 3513 (br, OH stretch), 3393 (br, NH stretch), 2971, 2871, 1719 (C=O), 1626, 1584, 1457, 1283, 1140, 1027, 931, 866; **^{1}H NMR** (300 MHz, $CDCl_3$) δ 8.17 (s, 1H), 7.40 (dd, *J* = 9.8, 2.5 Hz, 1H), 7.25 (dd, *J* = 9.0, 4.7 Hz, 1H), 7.07 (d, *J* = 3.2 Hz, 1H), 6.92 (td, *J* = 9.0, 2.6 Hz, 1H), 5.44 (d, *J* = 1.2 Hz, 1H), 3.95 – 3.87 (m, 1H), 2.11 (s, 3H), 1.50 (d, *J* = 7.2 Hz, 3H), 1.26 (s, 9H); **^{13}C NMR** (101 MHz, $CDCl_3$) δ 176.5, 150.1, 149.6, 131.7, 127.5, 122.8, 116.6, 113.9, 112.0, 111.8, 104.6, 39.4, 35.4, 27.3, 18.4, 16.8; **HRMS** (ESI+) m/z: [M+Na] Calcd for $C_{18}H_{23}NO_3SNa$ 356.1296; Found 356.1298.

3ai

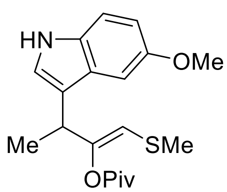

Prepared according to **GP3** using **1a** (40.0 mg, 0.20 mmol), **2i** (103 mg, 0.70 mmol), IPr*^{OMe}AuCl (11.8 mg, 0.01 mmol), AgOTs (5.6 mg, 0.02 mmol) in CH_2Cl_2 (1.0 mL); column chromatography (15% EtOAc in hexane) afforded **3ai** as a colorless liquid (43.0 mg, 63%); **IR** (Neat): ν_{max}/cm^{-1} 3386 (br, NH stretch), 2969, 2872, 1732 (C=O), 1625, 1487, 1210, 1118, 1015, 923, 800; **^{1}H NMR** (400 MHz, $CDCl_3$) δ 8.01 (s, 1H), 7.23 (d, *J* = 7.0 Hz, 1H), 7.22 (s, 1H), 7.01 (d, *J* = 2.4 Hz, 1H), 6.85 (dd,

J = 8.7, 2.5 Hz, 1H), 5.45 (d, *J* = 1.1 Hz, 1H), 3.92 (q, *J* = 7.1 Hz, 1H), 3.87 (s, 3H), 2.11 (s, 3H), 1.51 (d, *J* = 7.1 Hz, 3H), 1.27 (s, 9H); **^{13}C NMR** (101 MHz, $CDCl_3$) δ 176.0, 154.0, 150.2, 131.6, 127.3, 122.5, 117.1, 113.8, 112.5, 111.8, 101.9, 56.1, 39.3, 35.3, 27.3, 18.5, 16.9; **HRMS** (ESI+) m/z: [M+Na] Calcd for $C_{19}H_{25}NNaO_3S$ 370.1453; Found 370.1452.

3ak

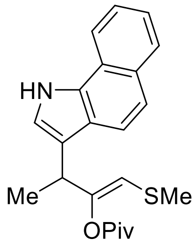

Prepared according to **GP3** using **1a** (40 mg, 0.20 mmol), **2k** (117 mg, 0.70 mmol), $IPr^{*OMe}AuCl$ (11.8 mg, 0.01 mmol), AgOTs (5.6 mg, 0.02 mmol) in CH_2Cl_2 (1.0 mL); column chromatography (10% EtOAc in hexane) afforded **3ak** as a colorless amorphous solid (62.0 mg, 84%); **IR** (Neat): ν_{max}/cm^{-1} 3409 (br, NH stretch), 2924, 1744 (C=O), 1458, 1367, 1188, 1057, 737, 689; **^{1}H NMR** (400 MHz, $CDCl_3$) δ 8.93 (s, 1H), 8.00 (d, *J* = 8.2 Hz, 1H), 7.94 (d, *J* = 7.3 Hz, 1H), 7.87 (d, *J* = 8.8 Hz, 1H), 7.54 – 7.47 (m, 2H), 7.46 – 7.41 (m, 1H), 7.08 (d, *J* = 4.0 Hz, 1H), 5.44 (d, *J* = 4.0 Hz, 1H), 4.07 (q, *J* = 7.1 Hz, 1H), 2.06 (s, 3H), 1.56 (d, *J* = 7.1 Hz, 3H), 1.31 (s, 9H); **^{13}C NMR** (101 MHz, $CDCl_3$) δ 176.3, 150.4, 131.1, 130.6, 128.9, 125.4, 124.0, 122.8, 121.9, 120.4, 120.2, 120.1, 119.6, 119.0, 114.0, 39.4, 35.3, 27.3, 18.7, 16.8; **HRMS** (ESI+): m/z [M+Na] Calcd for $C_{22}H_{25}NO_2SNa$ 390.1504; Found 390.1512.

3al

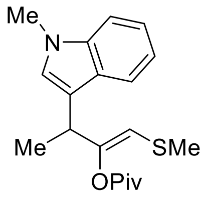

Prepared according to **GP3** using **1a** (40.0 mg, 0.20 mmol), **2l** (91.8 mg, 0.70 mmol), $IPr^{*OMe}AuCl$ (11.8 mg, 0.01 mmol), AgOTs (5.6 mg, 0.02 mmol) in CH_2Cl_2 (1.0 mL); column chromatography (20% EtOAc in hexane) afforded **3al** as a colorless amorphous solid (43.0 mg, 65%); **IR** (Neat): ν_{max}/cm^{-1} 2971, 2934, 1740 (C=O), 1612, 1478, 1329, 1114, 739; **^{1}H NMR** (400 MHz, $CDCl_3$) δ 7.75 (d, *J* = 7.9 Hz, 1H), 7.30 (d, *J* = 8.2 Hz, 1H), 7.25 – 7.20 (m, 1H), 7.14 – 7.10 (m, 1H), 6.92 (s, 1H), 5.45 (d, *J* = 1.2 Hz, 1H), 3.98 (q, *J* = 7.1 Hz, 1H), 3.77 (s, 3H), 2.11 (s, 3H), 1.52 (d, *J* = 7.1 Hz, 3H), 1.28 (s, 9H); **^{13}C NMR** (101 MHz, $CDCl_3$) δ 176.0, 150.4, 137.2, 127.3, 126.5, 121.7, 120.3, 119.1, 115.9, 113.6, 109.1, 39.3, 35.2, 32.8, 27.3, 18.7, 16.8; **HRMS** (ESI+): m/z [M+Na] Calcd for $C_{19}H_{25}NO_2SNa$ 354.1504; Found 354.1514.

3bm

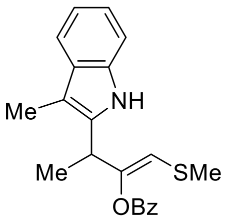

Prepared according to **GP3** using **1b** (44.0 mg, 0.20 mmol), **2m** (91.8 mg, 0.70 mmol), IPr*^{OMe}AuCl (11.8 mg, 0.01 mmol), AgOTs (5.6 mg, 0.02 mmol) in CH_2Cl_2 (1.0 mL); column chromatography (20% EtOAc in hexane) afforded **3am** as a colorless oil (55.0 mg, 78%); **IR** (Neat): ν_{max}/cm^{-1} 3398 (br, NH stretch), 2980, 2921, 1724 (C=O), 1450, 1267, 1226, 1085, 1064, 796, 732, 704; **^{1}H NMR** (400 MHz, $CDCl_3$) δ 8.15 (s, 1H), 8.07 (d, *J* = 7.2 Hz, 2H), 7.63 – 7.57 (m, 1H), 7.52 (d, *J* = 7.8 Hz, 1H), 7.45 (app. t, *J* = 7.5 Hz, 2H), 7.34 (d, *J* = 7.8 Hz, 1H), 7.17 (app. t, *J* = 7.8 Hz, 1H), 7.11 (app. t, *J* = 7.8 Hz, 1H), 5.75 (d, *J* = 1.3 Hz, 1H), 4.23 (qd, *J* = 7.1, 1.2 Hz, 1H), 2.27 (s, 3H), 2.24 (s, 3H), 1.53 (d, *J* = 7.1 Hz, 3H); **^{13}C NMR** (101 MHz, $CDCl_3$) δ 164.1, 147.7, 135.5, 134.4, 133.7, 130.3, 129.3, 129.1, 128.7, 121.6, 119.2, 118.5, 115.9, 110.8, 108.1, 35.6, 17.9, 17.0, 8.6; **HRMS** (ESI+) m/z: [M+Na] Calcd for $C_{21}H_{21}NO_2SNa$ 374.1191; Found 374.1193.

3am

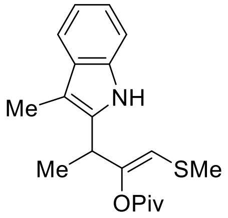

Prepared according to **GP3** using **1a** (40.0 mg, 0.20 mmol), **2m** (91.8 mg, 0.70 mmol), IPr*^{OMe}AuCl (11.8 mg, 0.01 mmol), AgOTs (5.6 mg, 0.02 mmol) in CH_2Cl_2 (1.0 mL); column chromatography (20% EtOAc in hexane) afforded **3am** as a colorless oil (39.5 mg, 59%); **IR** (Neat): ν_{max}/cm^{-1} 3293 (br, OH stretch), 3027, 2927, 2181, 1496, 1455, 1314, 1049, 1012, 748, 700; **^{1}H NMR** (400 MHz, $CDCl_3$) δ 8.01 (s, 1H), 7.52 – 7.49 (m, 1H), 7.31 – 7.29 (m, 1H), 7.17 – 7.07 (m, 2H), 5.62 (d, *J* = 1.5 Hz, 1H), 4.11 (qd, *J* = 7.2, 1.5 Hz, 1H), 2.26 (s, 3H), 2.23 (s, 3H), 1.44 (d, *J* = 7.2 Hz, 3H), 1.20 (s, 9H); **^{13}C NMR** (101 MHz, $CDCl_3$) δ 176.1, 147.4, 135.5, 134.7, 129.3, 121.6, 119.1, 118.4, 115.5, 110.7, 107.9, 39.3, 35.2, 27.2, 17.9, 17.0, 8.6; **HRMS** (ESI+) m/z: [M+Na] Calcd for $C_{19}H_{25}NO_2SNa$ 354.1504; Found 354.1514.

Determination of connectivity in 3am.

Direct reaction at indole C2 position or addition to C3 followed by selective 1,2 migration of the functionalised allyl unit	Addition to C3 followed by selective [2,3]-rearrangement	Addition to C3 followed by selective 1,2 migration of the methyl group	Addition to C2 followed by rearrangement

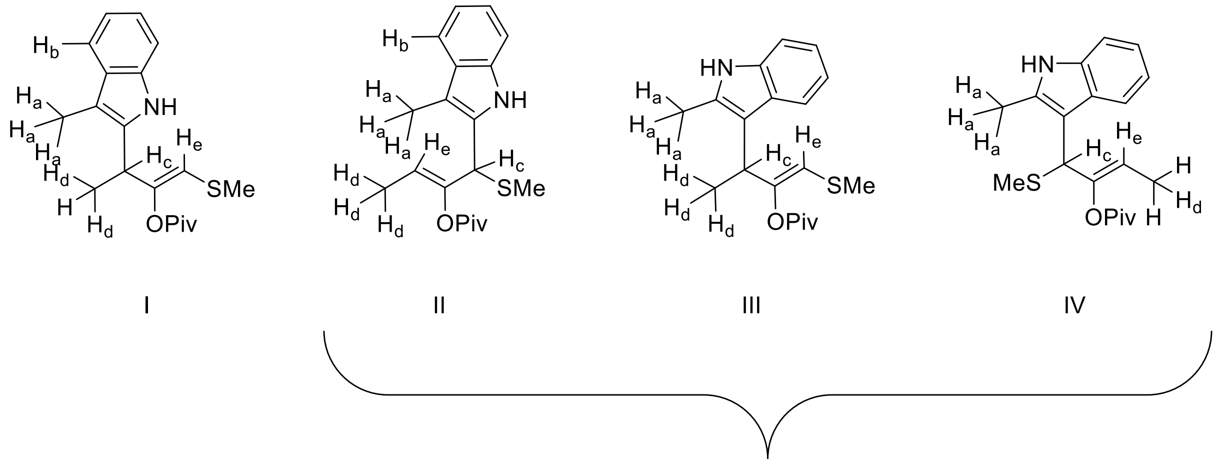

I II III IV

observed product

δ H_a = 2.26 , H_b = 7.51 , H_c = 4.11, H_d = 1.43, H_e= 5.62

other possible regioisomers not observed

^{1}H NMR (400 MHz, $CDCl_3$)

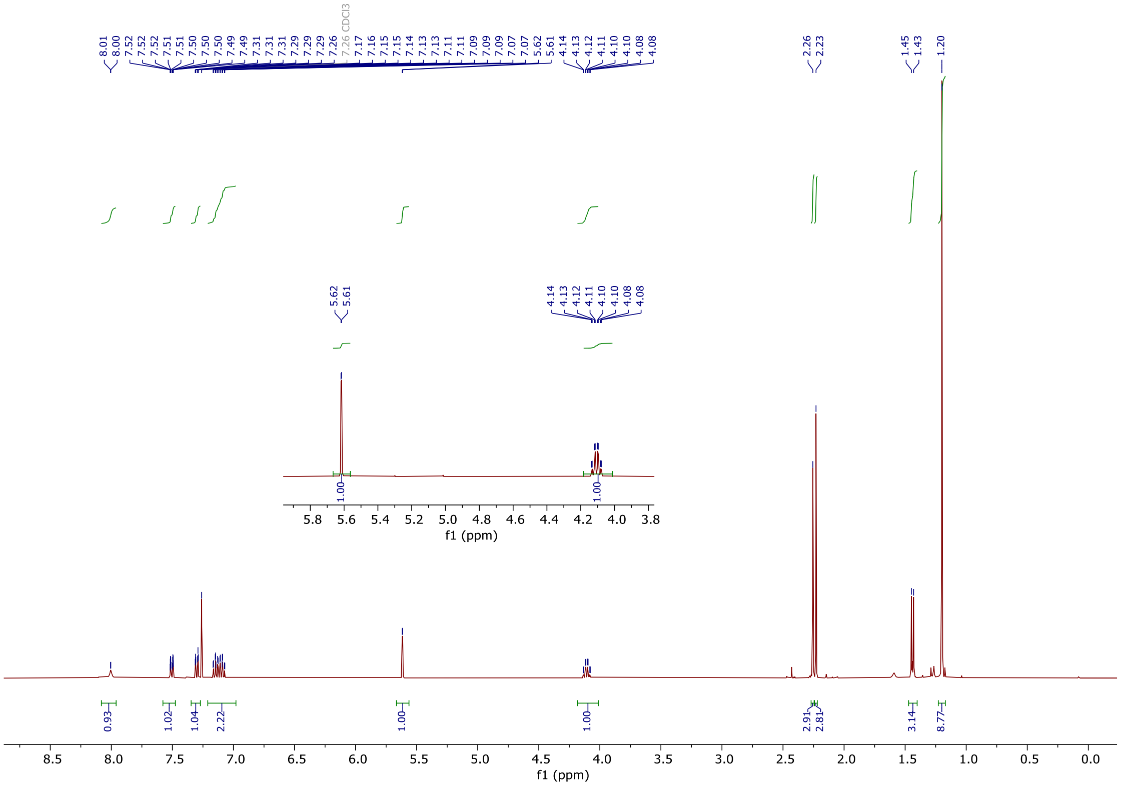

1. ^{1}H NMR spectra. The resonance at 4.11 ppm is a single proton with a ^{3}J coupling to a CH_3 group and a ^{4}J coupling to a CH. The chemical shift is compatible with an activated aliphatic position, validated using ^{13}C and HSQC NMR spectra, where the ^{1}H resonance at 4.11 ppm corresponds to ^{13}C at 35.1 ppm. This is consistent with structures I and III but not the vinylic position required in structures II and IV.
2. Furthermore, the resonance at 5.62 is a doublet for a vinylic proton with a ^{4}J coupling, inconsistent with structures II and IV and assigned as H_e.

^1H-^1H NOESY (400 MHz, $CDCl_3$) for 3am

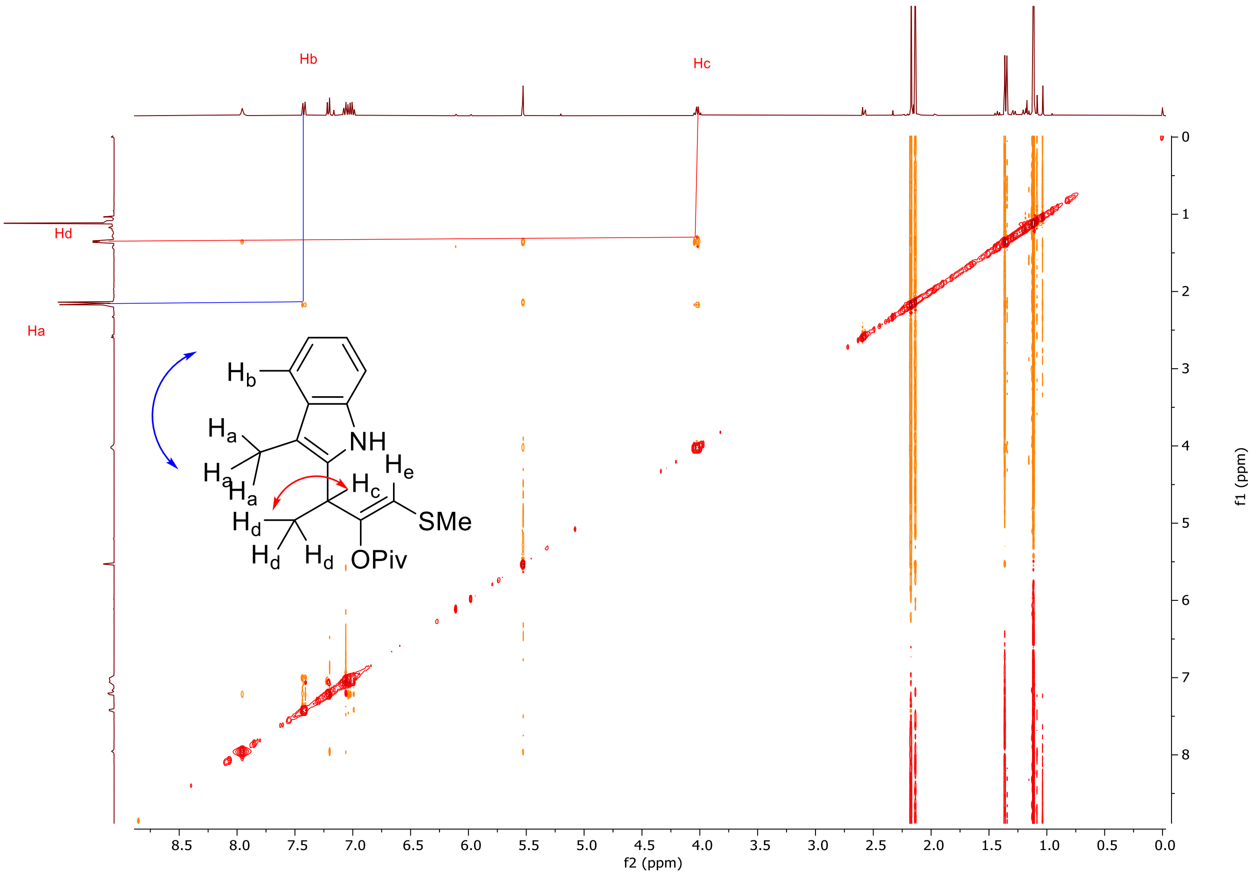

3. There is NOESY correlation between the methyl group on the indole (H_a δ 2.25, s) and indole aromatic H_b (δ 7.50). There is no NOESY correlation between N-H proton and the methyl group H_a. There is a very strong correlation between H_c and H_d as shown in spectra. This supports structure I only.

^{1}H-^{13}C HMBC (400 MHz, $CDCl_3$) for 3am

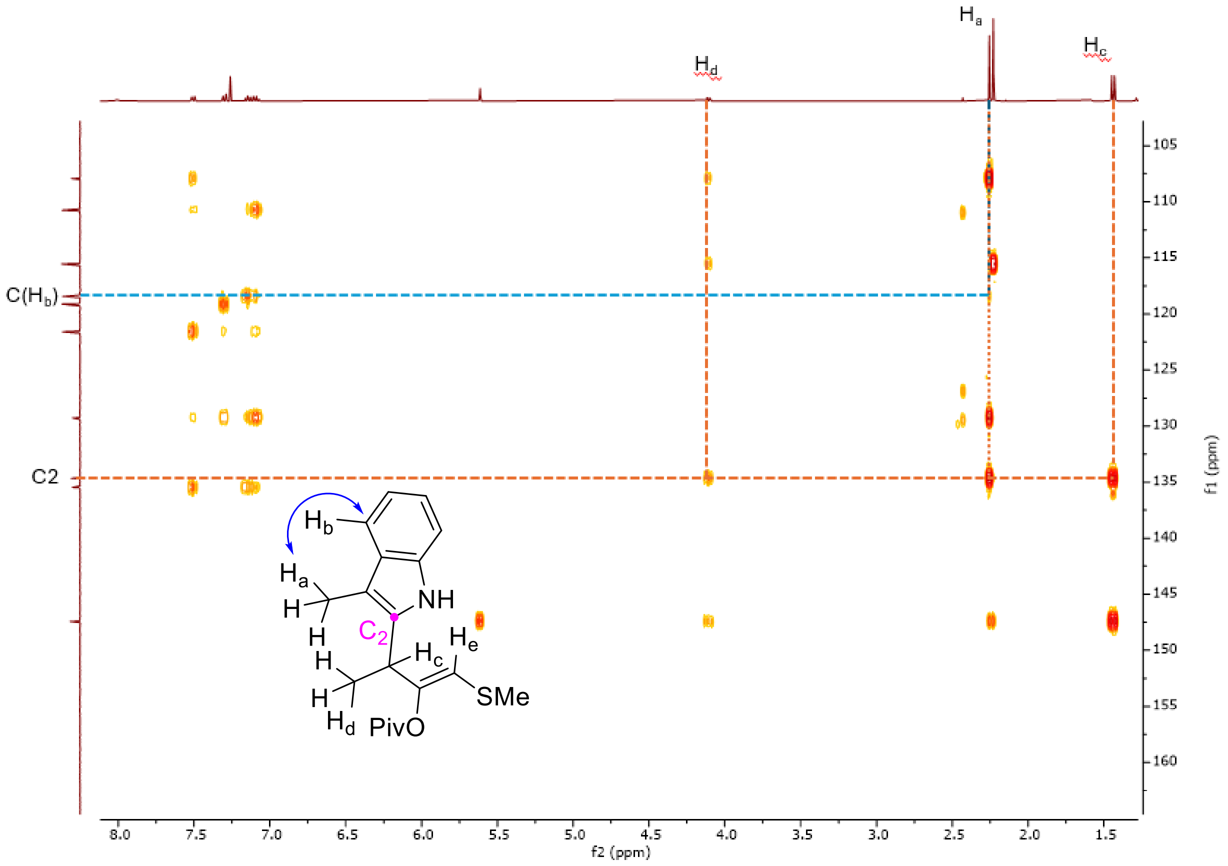

4. HMBC analysis shows a correlation between $C(H_b)$ = 118.4 with H_a = 2.26. This confirms structure I and eliminates the possibilities of structure II.
5. HMBC analysis also shows correlation between the indole C_2 (134.7) with methyl groups at 2.26 ppm (H_a) and 1.43 ppm (H_d) and the qd at 4.11(H_c)

3an

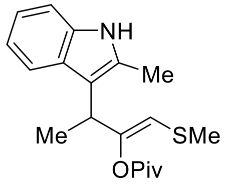

Prepared according to **GP3** using **1a** (20.3 mg, 0.10 mmol), **2n** (45.9 mg, 0.35 mmol), IPr*^{OMe}AuCl (5.9 mg, 0.005 mmol), AgOTs (2.8 mg, 0.01 mmol) in CH_2Cl_2 (0.5 mL); column chromatography (10% Et_2O in hexane) afforded **3an** as a colorless oil (19.5 mg, 59%); **IR** (Neat): ν_{max}/cm^{-1} 3394 (br, NH stretch), 2972, 2930, 2873, 1723 (C=O), 1459, 1429, 1128, 740, 601; **^{1}H NMR** (400 MHz, $CDCl_3$) δ 7.77 (s, 1H), 7.60 – 7.57 (m, 1H), 7.27 – 7.19 (m, 1H), 7.14 – 6.97 (m, 2H), 5.57 (d, *J* = 1.8 Hz, 1H), 4.07 (qd, *J* = 7.2, 1.8 Hz, 1H), 2.36 (s, 3H), 2.18 (s, 3H), 1.51 (d, *J* = 7.2 Hz, 3H), 1.14 (s, 9H); **^{13}C NMR** (101 MHz, $CDCl_3$) δ 176.0, 149.6, 135.4, 131.6, 127.7, 120.9, 119.5, 119.3, 113.4, 112.0, 110.3, 39.2, 34.2, 27.2, 18.1, 16.9, 12.2. **HRMS** (ESI+) m/z: [M+Na] Calcd for $C_{19}H_{25}NNaO_2S$ 354.1504; Found 354.1514.

3ca

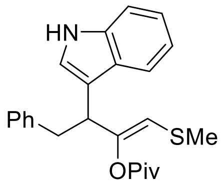

Prepared according to **GP3** using **1c** (27.6 mg, 0.10 mmol), **2a** (41.0 mg, 0.35 mmol), IPr*^{OMe}AuCl (5.9 mg, 0.005 mmol), AgOTs (2.8 mg, 0.01 mmol) in CH_2Cl_2 (0.5 mL); column chromatography (20% EtOAc in hexane) afforded **3ca** as an off-white solid (29.0 mg, 81%); **IR** (Neat): ν_{max}/cm^{-1} 3364 (br, NH stretch), 2967, 2922, 1725 (C=O), 1458, 1432, 1122, 1103, 737, 698; **^{1}H NMR** (400 MHz, $CDCl_3$) δ 7.95 (s, 1H), 7.74 (d, *J* = 7.9 Hz, 1H), 7.32 (d, *J* = 7.9 Hz, 1H), 7.20 – 7.08 (m, 5H), 7.07 – 7.02 (m, 2H), 6.89 (d, *J* = 2.5 Hz, 1H), 5.50 (d, *J* = 1.1 Hz, 1H), 4.16 (dd, *J* = 9.2, 5.2 Hz, 1H), 3.33 (dd, *J* = 13.8, 5.2 Hz, 1H), 3.17 (dd, *J* = 13.8, 9.2 Hz, 1H), 2.08 (s, 3H), 1.24 (s, 9H); **^{13}C NMR** (101 MHz, $CDCl_3$) δ 176.0, 148.5, 140.3, 136.5, 129.0, 128.2, 127.0, 126.0, 122.9, 122.1, 120.3, 119.6, 115.1, 114.6, 111.1, 43.2, 39.4, 38.6, 27.3, 16.9; **HRMS** (ESI+) m/z: [M+Na] Calcd for $C_{24}H_{27}NNaO_2S$ 416.1660; Found 416.1668.

3da

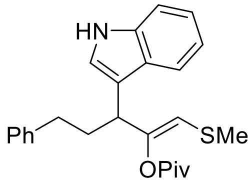

Prepared according to **GP3** using **1d** (58.0 mg, 0.20 mmol), **2a** (82.0 mg, 0.70 mmol), IPr*^{OMe}AuCl (11.8 mg, 0.01 mmol), AgOTs (5.6 mg, 0.02 mmol) in CH_2Cl_2 (1.0 mL); column chromatography (20% EtOAc in hexane) afforded **3da** as colorless amorphous solid (60.0 mg, 74%); **IR** (Neat): ν_{max}/cm^{-1} 3405 (br, NH stretch), 2970, 2926, 1737 (C=O), 1478, 1455, 1118, 740, 699; **^{1}H NMR** (400 MHz, $CDCl_3$) δ 8.20 (s, 1H), 7.77 (d, *J* = 7.9 Hz, 1H), 7.46 (d, *J* = 8.1 Hz, 1H),

7.36 – 7.26 (m, 4H), 7.22 – 7.21 (m, 3H), 7.15 (d, *J* = 2.3 Hz, 1H), 5.66 – 5.62 (m, 1H), 3.94 (dd, *J* = 9.2, 5.7 Hz, 1H), 2.75 – 2.70 (m, 2H), 2.40 – 2.21 (m, 2H), 2.21 (s, 3H), 1.30 (s, 9H); **^{13}C NMR** (101 MHz, $CDCl_3$) δ 175.8, 148.9, 142.2, 136.6, 128.6, 128.4, 127.1, 125.9, 122.4, 122.2, 120.0, 119.6, 115.2, 114.3, 111.2, 40.8, 39.3, 34.2, 34.0, 27.3, 16.9; **HRMS** (ESI+) m/z: [M+Na] Calcd for $C_{25}H_{29}NNaO_2S$ 430.1817; Found 430.1827.

3ea

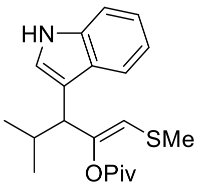

Prepared according to **GP3** using **1e** (45.6 mg, 0.20 mmol), **2a** (82.0 mg, 0.70 mmol), IPr*^{OMe}AuCl (11.8 mg, 0.01 mmol), AgOTs (5.6 mg, 0.02 mmol) in CH_2Cl_2 (1.0 mL); column chromatography (20% EtOAc in hexane) afforded **3ea** as colorless amorphous solid (51.0 mg, 74%); **IR (Neat** ν_{max}/cm^{-1} 3473 (br, NH stretch), 2969, 1727 (C=O), 1457, 1365, 1229, 740; **^{1}H NMR** (400 MHz, $CDCl_3$) δ 8.09 (s, 1H), 7.68 (d, *J* = 7.9 Hz, 1H), 7.34 (d, *J* = 7.9 Hz, 1H), 7.20 – 7.14 (m, 1H), 7.13 – 7.08 (m, 1H), 7.02 (d, *J* = 2.4 Hz, 1H), 5.66 (s, 1H), 3.63 (d, *J* = 7.5 Hz, 1H), 2.34 – 2.22 (m, 1H), 2.15 (s, 3H), 1.24 (s, 9H), 1.07 (d, *J* = 6.8 Hz, 3H), 0.91 (d, *J* = 6.8 Hz, 3H); **^{13}C NMR** (101 MHz, $CDCl_3$) δ 175.4, 148.8, 136.1, 127.9, 122.5, 121.9, 119.9, 119.5, 114.7, 114.2, 111.1, 48.5, 39.3, 30.7, 27.4, 21.8, 20.9, 17.1; **HRMS** (ESI+) m/z: [M+H] Calcd for $C_{20}H_{27}NO_2SNa$ 368.1660; Found 368.1667.

3fa

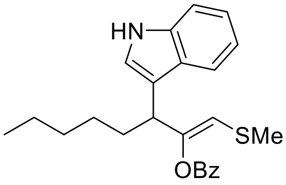

Prepared according to **GP3** using **1f** (22.0 mg, 0.10 mmol), **2a** (41.0 mg, 0.35 mmol), IPr*^{OMe}AuCl (5.9 mg, 0.005 mmol), AgOTs (2.8 mg, 0.01 mmol) in CH_2Cl_2 (0.5 mL); column chromatography (25% EtOAc in hexane) afforded **3fa** as a colorless amorphous solid (55.0 mg, 70%); **IR** (Neat): ν_{max}/cm^{-1} 3404 (br, NH stretch), 2925, 2857, 1724 (C=O), 1452, 1263, 1232, 1217, 1096, 1064, 740, 704; **^{1}H NMR** (400 MHz, $CDCl_3$) δ 8.10 – 8.08 (m, 3H), 7.77 (d, *J* = 7.9 Hz, 1H), 7.59 (t, *J* = 7.4 Hz, 1H), 7.45 (t, *J* = 7.7 Hz, 2H), 7.36 (d, *J* = 8.0 Hz, 1H), 7.20 (t, *J* = 8.0 Hz, 1H), 7.12 (t, *J* = 8.0 Hz, 1H), 7.05 (d, *J* = 2.4 Hz, 1H), 5.68 (d, *J* = 0.8 Hz, 1H), 3.97 – 3.94 (m, 1H), 2.14 (s, 3H), 2.09 – 1.89 (m, 2H), 1.43 –1.24 (m, 2H), 1.31 – 1.19 (m, 4H), 0.83 (t, *J* = 7.0 Hz, 3H); **^{13}C NMR** (101 MHz, $CDCl_3$) δ 163.9, 149.8, 136.6, 133.4, 130.2, 129.6, 128.7, 128.6, 127.1, 122.4, 122.1, 121.4, 121.3, 120.6, 120.0, 119.5, 118.7, 115.6, 114.3, 111.2, 111.2, 41.7, 32.6, 31.9, 27.6, 22.6, 17.0, 14.2; **HRMS** (ESI+): m/z [M+Na] Calcd for $C_{24}H_{27}NO_2SNa$ 416.1660; Found 416.1657.

3ga

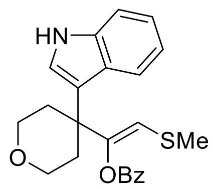

Prepared according to **GP3** using **1g** (27.6 mg, 0.10 mmol), **2a** (41.0 mg, 0.35 mmol), IPr*^{OMe}AuCl (5.9 mg, 0.005 mmol), AgOTs (2.8 mg, 0.01 mmol) in CH_2Cl_2 (0.5 mL); column chromatography (25% EtOAc in hexane) afforded **3ga** as a colorless amorphous solid (8.00 mg, 20%); **IR** (Neat): ν_{max}/cm^{-1} 3402 (br, NH stretch), 2980, 2970, 1726 (C=O), 1450, 1378, 1228, 1083, 1064, 733, 702; **^{1}H NMR** (400 MHz, $CDCl_3$) δ 8.17 (s, 1H), 8.08 – 8.05 (m, 2H), 7.83 (d, *J* = 8.1 Hz, 1H), 7.59 (t, *J* = 7.4 Hz, 1H), 7.48 – 7.39 (m, 3H), 7.23 (t, *J* = 7.4 Hz, 1H), 7.14 – 7.09 (m, 2H), 5.66 (s, 1H), 3.87 – 3.83 (m, , 2H), 3.68 – 3.62 (m, 2H), 2.36 – 2.33 (m, 4H), 2.09 (s, 3H); **^{13}C NMR** (101 MHz, $CDCl_3$) δ 163.5, 150.6, 137.2, 133.5, 130.4, 129.4, 128.6, 126.0, 123.8, 122.2, 121.9, 119.7, 115.9, 111.5, 64.4, 43.5, 33.6, 17.1 (One C_{arom} resonance not observed); **HRMS** (ESI-TOF): m/z [M+Na] Calcd for $C_{23}H_{23}NO_3SNa$ 416.1296; Found 416.1306.

3ha

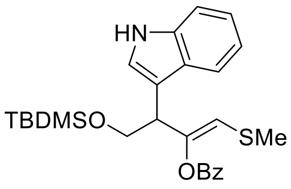

Prepared according to **GP3** using **1h** (22.0 mg, 0.10 mmol), **2a** (41.0 mg, 0.35 mmol), IPr*^{OMe}AuCl (5.9 mg, 0.005 mmol), AgOTs (2.8 mg, 0.01 mmol) in CH_2Cl_2 (0.5 mL); column chromatography (25% EtOAc in hexane) afforded **3ha** as a colorless amorphous solid (16.0 mg, 35%); **IR** (Neat): ν_{max}/cm^{-1} 3407 (br, NH stretch), 2952, 2926, 2855, 1728 (C=O), 1457, 1249, 1084, 1065, 833, 775, 739, 704; **^{1}H NMR** (400 MHz, $CDCl_3$) δ 8.12 – 8.08 (m, 2H), 8.04 (s, 1H), 7.82 (d, *J* = 7.4 Hz, 1H), 7.58 (t, *J* = 7.4 Hz, 1H), 7.45 (t, *J* = 7.4 Hz, 2H), 7.35 (d, *J* = 8.0 Hz, 1H), 7.28 (d, *J* = 2.3 Hz, 1H), 7.21 – 7.17 (m, 1H), 7.15 – 7.11 (m, 1H), 5.71 (d, *J* = 0.8 Hz, 1H), 4.16 – 4.10 (m, 3H), 2.13 (s, 3H), 0.88 (s, 9H), 0.03 (s, 3H), 0.01 (s, 3H); **^{13}C NMR** (101 MHz, $CDCl_3$) δ 163.9, 146.7, 136.1, 133.4, 130.2, 129.7, 128.6, 127.4, 123.2, 122.1, 120.1, 119.7, 116.6, 113.8, 111.0, 64.2, 44.2, 26.0, 18.4, 17.0, -5.3; **HRMS** (ESI+): m/z [M+Na] Calcd for $C_{26}H_{33}NO_3SSiNa$ 490.1848; Found 490.1846.

3ia

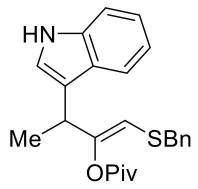

Prepared according to **GP3** using **1i** (27.9 mg, 0.10 mmol), **2a** (41.0 mg, 0.35 mmol), IPr*^{OMe}AuCl (5.9 mg, 0.005 mmol), AgOTs (2.8 mg, 0.01 mmol) in CH_2Cl_2 (0.5 mL); column chromatography (15% EtOAc in hexane) afforded **3ia**

as a colorless amorphous solid (65.5 mg, 83%); **IR** (Neat): ν_{max}/cm^{-1} 3405 (br, NH stretch), 2970, 1737 (C=O), 1455, 1116, 1029, 740, 699; **^{1}H NMR** (400 MHz, $CDCl_3$) δ 8.07 (s, 1H), 7.70 (d, *J* = 7.9 Hz, 1H), 7.36 (d, *J* = 7.9 Hz, 1H), 7.25 – 7.18 (m, 4H), 7.13 (t, *J* = 7.5 Hz, 1H), 7.09 – 7.6 (m, m, 2H), 6.93 (d, *J* = 2.4 Hz, 1H), 5.47 (d, *J* = 1.4 Hz, 1H), 3.97 (q, *J* = 7.1 Hz, 1H), 3.71 – 3.64 (m, 2H), 1.49 (d, *J* = 7.1 Hz, 3H), 1.29 (s, 9H); **^{13}C NMR** (101 MHz, $CDCl_3$) δ 176.1, 151.6, 137.7, 136.5, 129.0, 128.5, 127.1, 126.8, 122.1, 121.8, 120.2, 119.6, 117.1, 111.2, 110.8, 39.3, 37.7, 35.5, 27.3, 18.4; **HRMS** (ESI+): m/z [M+NH_4] Falcd for $C_{24}H_{31}N_2O_2S$ 416.1660; Found 416.1663.

3ja

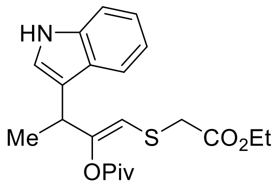

Prepared according to **GP3** using **1j** (54.6 mg, 0.20 mmol), **2a** (82.0 mg, 0.70 mmol), IPr*^{OMe}AuCl (11.8 mg, 0.01 mmol), AgOTs (5.6 mg, 0.02 mmol) in CH_2Cl_2 (1.0 mL); column chromatography (20% EtOAc in hexane) afforded **3ja** as colorless oil (45.0 mg, 59%); **IR** (Neat): ν_{max}/cm^{-1} 3332 (br, NH stretch), 2970, 1748 (C=O), 1716 (C=O), 1457, 1368, 1299, 1245, 1101, 1020, 783, 748; **^{1}H NMR** (400 MHz, $CDCl_3$) δ 8.10 (s, 1H), 7.72 (d, *J* = 7.9 Hz, 1H), 7.34 (d, *J* = 8.1 Hz, 1H), 7.20 – 7.16 (m, 1H), 7.13 – 7.09 (m, 1H), 7.03 (d, *J* = 2.3 Hz, 1H), 5.66 (d, *J* = 1.2 Hz, 1H), 4.10 – 3.97 (m, 3H), 3.19 (s, 2H), 1.52 (d, *J* = 7.1 Hz, 3H), 1.25 (s, 9H), 1.16 (t, *J* = 7.1 Hz, 3H); **^{13}C NMR** (101 MHz, $CDCl_3$) δ 175.9, 169.8, 152.6, 136.5, 126.8, 122.2, 121.8, 120.1, 119.6, 117.1, 111.1, 110.2, 61.5, 39.3, 35.4, 35.0, 27.3, 18.6, 14.1.; **HRMS** (ESI+) m/z: [M+Na] Calcd for $C_{21}H_{27}NaNO_4S$ 412.1558; Found 412.1561.

3ka

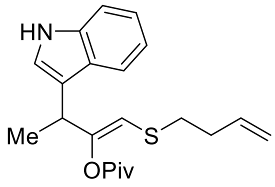

Prepared according to **GP3** using **1k** (48.0 mg, 0.20 mmol), **2a** (82.0 mg, 0.70 mmol), IPr*^{OMe}AuCl (11.8 mg, 0.01 mmol), AgOTs (5.6 mg, 0.02 mmol) in CH_2Cl_2 (1.0 mL); column chromatography (15% EtOAc in hexane) afforded **3ka** as a colorless amorphous solid (58.0 mg, 56%); **IR** (Neat): ν_{max}/cm^{-1} 3293 (br, OH stretch), 3027, 2927, 2181, 1496, 1455, 1314, 1049, 1012, 748, 700; **^{1}H NMR** (400 MHz, $CDCl_3$) δ 8.02 (s, 1H), 7.78 – 7.75 (m, 1H), 7.36 (dt, *J* = 8.1, 1.0 Hz, 1H), 7.22 – 7.16 (m, 1H), 7.15 – 7.09 (m, 1H), 7.05 (d, *J* = 2.3 Hz, 1H), 5.76 – 5.66 (m, 1H), 5.44 (d, *J* = 1.1 Hz, 1H), 4.99 – 4.93 (m, 2H), 3.98 (q, *J* = 7.1 Hz, 1H), 2.52 (t, *J* = 7.5 Hz, 2H), 2.21 (q, *J* = 7.5 Hz, 2H), 1.52 (d, *J* = 7.1 Hz, 3H), 1.27 (s, 9H); **^{13}C NMR** (101 MHz, $CDCl_3$) δ 176.0, 151.5, 136.6, 136.4, 126.9, 122.2, 121.7, 120.4, 119.6, 117.5, 116.2, 111.9, 111.1, 39.3, 35.5, 34.6,

33.0, 27.4, 18.5; **HRMS** (ESI+) m/z: [M+Na] Calcd for $C_{21}H_{27}NO_2SNa$ 380.1660; Found 380.1661.

General procedure for catalysis reactions favouring 1,3-carboxylate migration (GP4)

To the mixture of propargyl carboxylate (1.0 equiv.) and indole (3.5 equiv.) dissolved in anhydrous CH_2Cl_2 (0.2 M) in a heat-gun dried Schlenk tube under argon, was added $IPr^{*OMe}AuCl$ (5 mol%) followed by $NaBAr^F$ (10 mol%). The solution was stirred at rt for 18 h. The reaction mixture was filtered through a pad of celite and the celite was washed with EtOAc (3 × ~1 mL/0.2 mmol). The solution was concentrated under reduced pressure and the product was purified by flash column chromatography to obtain arylated products.

4aa

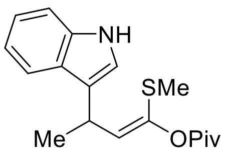

Prepared according to **GP4** using **1a** (40.0 mg, 0.20 mmol), **2a** (82.0 mg, 0.70 mmol), $IPr^{*OMe}AuCl$ (11.8 mg, 0.01 mmol), AgOTs (5.6 mg, 0.02 mmol) in CH_2Cl_2 (1.0 mL); column chromatography (20% EtOAc in hexane) afforded **4aa** as green amorphous solid (30.2 mg, 48%); **IR** (Neat): ν_{max}/cm^{-1} 3354 (br, NH stretch), 2967, 2927, 1712 (C=O), 1456, 1283, 1120, 1009, 992, 908, 741; **^{1}H NMR** (400 MHz, $CDCl_3$) δ 7.98 (s, br, 1H), 7.74 (d, *J* = 8.0 Hz, 1H), 7.35 (d, *J* = 8.0 Hz, 1H), 7.24 – 7.07 (m, 2H), 7.04 (dd, *J* = 2.5, 1.0 Hz, 1H), 5.65 (d, *J* = 9.9 Hz, 1H), 4.30 – 4.22 (m, 1H), 2.29 (s, 3H), 1.53 (d, *J* = 7.1 Hz, 3H), 1.28 (s, 9H); **^{13}C NMR** (101 MHz, $CDCl_3$) δ 176.8, 141.7, 136.7, 131.2, 126.8, 122.2, 120.5, 120.3, 119.8, 119.4, 111.3, 39.2, 31.1, 27.3, 21.1, 15.7; **HRMS** (ES+) m/z: [M+H] Calcd for $C_{18}H_{24}NO_2S$ 318.1528; Found 318.1530.

4la

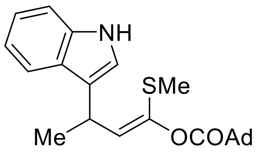

Prepared according to **GP4** using **1l** (55.8 mg, 0.20 mmol), **2a** (82.0 mg, 0.70 mmol), $IPr^{*OMe}AuCl$ (11.8 mg, 0.01 mmol), AgOTs (5.6 mg, 0.02 mmol) in CH_2Cl_2 (1.0 mL); column chromatography (20% EtOAc in hexane) afforded **4la** as green amorphous solid (30.2 mg, 79%); **IR** (Neat): ν_{max}/cm^{-1} 3407 (br, NH stretch), 2970, 2905, 2851, 1735 (C=O), 1483, 1211, 1180, 1048, 968, 738; **^{1}H NMR** (400 MHz, $CDCl_3$) δ 7.99 (s, 1H), 7.73 (d, *J* = 7.6 Hz, 1H), 7.32 (d, *J* = 8.1 Hz, 1H), 7.17 (app td, *J* = 7.6, 1.4 Hz, 1H), 7.11 (app td, *J* = 7.6, 1.3 Hz, 1H), 7.01 (d, *J* = 1.5 Hz, 1H), 5.62 (d, *J* = 9.9 Hz, 1H), 4.23 (dq, *J* = 9.9, 7.0 Hz, 1H), 2.27 (s, 3H), 2.05 – 2.02 (m, 3H), 1.96 (d, *J* = 3.2 Hz, 6H), 1.75 – 1.69

(m, 6H), 1.51 (d, *J* = 7.0 Hz, 3H); **^{13}C NMR** (101 MHz, $CDCl_3$) δ 175.9, 141.6, 136.7, 136.6, 131.0, 126.8, 122.2, 120.5, 119.8, 119.4, 111.3, 41.2, 38.9, 36.5, 31.1, 28.0, 21.1, 15.6; **HRMS** (ESI+) m/z: [M+Na] Calcd for $C_{24}H_{29}NO_4SNa$ 418.1817; Found 418.1828.

4lb

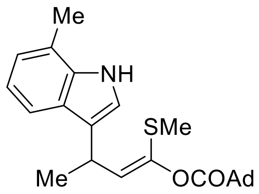

Prepared according to **GP4** using **1l** (27.9 mg, 0.10 mmol), **2b** (45.9 mg, 0.35 mmol), IPr*^{OMe}AuCl (11.8 mg, 0.01 mmol), AgOTs (5.6 mg, 0.02 mmol) in CH_2Cl_2 (1.0 mL); column chromatography (15% EtOAc in hexane) afforded **4lb** as a colorless liquid (30.8 mg, 76%); **IR** (Neat): ν_{max}/cm^{-1} 3381 (br, NH stretch), 2970, 2905, 2851, 1736 (C=O), 1452, 1215, 1047, 905, 792; **^{1}H NMR** (400 MHz, $CDCl_3$) δ 7.89 (s, 1H), 7.60 (d, *J* = 7.9 Hz, 1H), 7.09 – 6.99 (m, 3H), 5.63 (d, *J* = 9.9 Hz, 1H), 4.31 – 4.20 (m, 1H), 2.47 (s, 3H), 2.28 (s, 3H), 2.05 (s, 3H), 1.97 (d, *J* = 2.6 Hz, 6H), 1.79 – 1.70 (m, 6H), 1.53 (d, *J* = 7.0 Hz, 3H); **^{13}C NMR** (101 MHz, $CDCl_3$) δ 175.9, 141.4, 136.3, 131.0, 126.3, 122.7, 120.8, 120.4, 120.2, 119.7, 117.5, 41.2, 39.0, 38.9, 36.5, 31.2, 29.8, 28.0, 21.2, 16.7, 15.7; **HRMS** (ESI+): m/z [M+NH$_4$] Calcd for $C_{25}H_{35}N_2O_2S$ 427.2419; Found 427.2427.

4lf

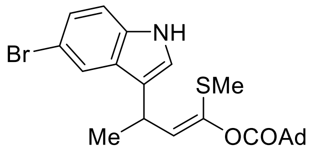

Prepared according to **GP4** using **1l** (27.9 mg, 0.10 mmol), **2f** (68.6 mg, 0.35 mmol), IPr*^{OMe}AuCl (5.9 mg, 0.005 mmol), AgOTs (2.8 mg, 0.01 mmol) in CH_2Cl_2 (0.5 mL); column chromatography (15% EtOAc in hexane) afforded **4lf** as a colorless amorphous solid (34.2 mg, 72%); **IR** (Neat): ν_{max}/cm^{-1} 3414 (br, NH stretch), 2905, 2851, 1732 (C=O), 1472, 1323, 1210, 1180, 1046, 781, 745, 730; **^{1}H NMR** (400 MHz, $CDCl_3$) δ 8.04 (s, 1H), 7.85 (d, *J* = 1.9 Hz, 1H), 7.24 (dd, *J* = 8.0, 1.9 Hz, 1H), 7.18 (d, *J* = 8.0 Hz, 1H), 7.01 (dd, *J* = 2.5, 1.0 Hz, 1H), 5.55 (d, *J* = 9.9 Hz, 1H), 4.20 – 4.12 (m, 1H), 2.28 (s, 3H), 2.05 – 2.02 (m, 3H), 1.97 (d, *J* = 3.0 Hz, 6H), 1.77 - 1.68 (m, 6H), 1.48 (d, *J* = 6.9 Hz, 3H); **^{13}C NMR** (101 MHz, $CDCl_3$) δ 175.9, 141.8, 135.3, 130.3, 128.6, 124.9, 122.4, 121.8, 120.0, 112.7, 112.7, 41.3, 38.9, 36.5, 30.9, 28.0, 21.1, 15.4; **HRMS** (ESI+): m/z [M+NH$_4$] Calcd for $C_{24}H_{32}{}^{79}BrN_2O_2S$ 491.1368; Found 491.1378.

4mb

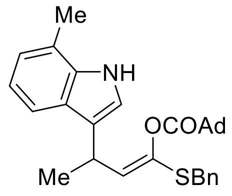

Prepared according to **GP4** using **1m** (27.9 mg, 0.10 mmol), **2b** (45.9 mg, 0.35 mmol), IPr*^{OMe}AuCl (5.9 mg, 5 mol%, 0.005 mmol), AgOTs (2.8 mg, 0.01 mmol) in CH_2Cl_2 (0.5 mL); Analysis of the ^{1}H NMR spectra of the crude reaction mixture in the presence of a known amount of methyl-2,5-dinitrobenzoate indicated the presence of **3mb** in 17% assigned by analogy to compounds **3**; column chromatography (20% EtOAc in hexane) afforded **4mb** as a colorless amorphous solid (30 mg, 62%); **IR** (Neat): ν_{max}/cm^{-1} 3404 (br, NH stretch), 2905, 2851, 1736 (C=O), 1452, 1210, 1044, 867, 731, 696; **^{1}H NMR** (400 MHz, $CDCl_3$) δ 7.80 (s, 1H), 7.37 (d, *J* = 7.0 Hz, 1H), 7.34 – 7.26 (m, 4H), 7.25 (s, 1H), 7.01 – 6.94 (m, 2H), 6.92 (d, *J* = 2.0 Hz, 1H), 5.69 (d, *J* = 9.9 Hz, 1H), 4.10 – 3.99 (m, 1H), 3.94 – 3.84 (m, 2H), 2.44 (s, 3H), 2.02 (s, 3H), 1.93 (d, *J* = 2.7 Hz, 6H), 1.78 – 1.66 (m, 6H), 1.29 (d, *J* = 7.0 Hz, 3H) **^{13}C NMR** (101 MHz, $CDCl_3$) δ 175.8, 140.1, 138.3, 136.2, 134.2, 129.1, 128.7, 127.3, 126.2, 122.6, 120.7, 120.3, 120.2, 119.7, 117.6, 41.2, 38.9, 37.1, 36.6, 31.4, 28.0, 21.0, 16.7; **HRMS** (ESI-TOF): m/z [M+Na] Calcd for $C_{31}H_{35}NO_2SNa$ 508.2286; Found 508.2290.

4nb

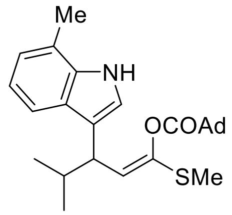

Prepared according to **GP4** using **1n** (30.6 mg, 0.10 mmol), **2b** (45.9 mg, 0.35 mmol), IPr*^{OMe}AuCl (5.9 mg, 0.005 mmol), AgOTs (2.8 mg, 0.01 mmol) in CH_2Cl_2 (0.5 mL); column chromatography (10% EtOAc in hexane) afforded **4nb** (31.8 mg, 71% yield) as a colorless liquid; **IR** (Neat): ν_{max}/cm^{-1} 3397 (br, NH stretch), 2970, 2908, 2851, 1728 (C=O), 1452, 1364, 1216, 1062, 748; **^{1}H NMR** (400 MHz, $CDCl_3$): δ 7.88 (s, 1H), 7.54 (d, *J* = 7.1 Hz, 1H), 7.24 (s, 1H), 7.05 – 7.00 (m, 1H), 6.97 (t, *J*, 1H), 5.70 (d, *J* = 9.9 Hz, 1H), 3.85 (dd, *J* = 10.3, 8.1 Hz, 1H), 2.45 (s, 3H), 2.20 (s, 1H), 2.17 (s, 3H), 2.02 (s, 3H), 1.94 (d, *J* = 2.7 Hz, 6H), 1.76 – 1.65 (m, 6H), 1.00 (d, *J* = 6.60 Hz, 3H), 0.93 (d, *J* = 6.60 Hz, 3H); **^{13}C NMR** (101 MHz, $CDCl_3$): δ 176.0, 142.2, 136.1, 128.3, 126.6, 122.6, 121.1, 120.4, 119.6, 119.0, 117.5, 44.2, 41.2, 39.0, 36.6, 32.7, 28.0, 21.4, 20.3, 16.7, 15.5; **HRMS** (ESI+): m/z [M+Na] Calcd for $C_{27}H_{35}NO_2SNa$ 460.2286; Found 460.2280.

4ob

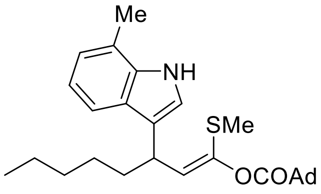

Prepared according to **GP4** using **1o** (27.9 mg, 0.1 mmol), **2b** (45.9 mg, 0.35 mmol), IPr*^{OMe}AuCl (5.9 mg, 0.005 mmol), AgOTs (2.8 mg, 0.01 mmol) in CH_2Cl_2 (0.5 mL); column chromatography (15% EtOAc in hexane) afforded **4ob** as a colorless amorphous solid (32.9 mg, 71%); **IR** (Neat): ν_{max}/cm^{-1} 3412 (br, NH stretch), 2906, 2852, 1733 (C=O), 1452, 1209, 1179, 1046, 960, 781, 744, 792; **^{1}H NMR** (400 MHz, $CDCl_3$) δ 7.88 (s, 1H), 7.58 (d, *J* = 7.8 Hz, 1H), 7.06 – 6.97 (m, 3H),5.59 (d, *J* = 10.2 Hz, 1H), 4.09 (td, *J* = 9.4, 5.8 Hz, 1H), 2.47 (s, 3H), 2.24 (s, 3H), 2.02 (s, 3H), 1.97 (d, *J* = 2.6 Hz, 7H), 1.79 – 1.68 (m, 7H), 1.53 – 1.37 (m, 2H), 1.38 – 1.30 (m, 4H), 0.89 (t, *J* = 7.0 Hz, 3H); **^{13}C NMR** (101 MHz, $CDCl_3$) δ 175.8, 142.0, 136.2, 130.0, 126.4, 122.6, 120.4, 120.4, 119.9, 119.6, 117.5, 41.2, 38.9, 36.8, 36.6, 35.5, 32.0, 28.0, 27.3, 22.8, 16.7, 15.5, 14.3; **HRMS** (ESI-TOF): m/z [M+Na] Calcd for $C_{29}H_{39}NO_2SNa$ 488.2599; Found 488.2604.

Transformation of catalysis products

5

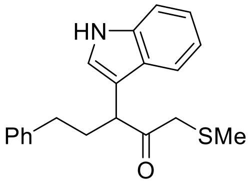

In a dram vial was dissolved **3da** (20.0 mg, 0.05 mmol) MeOH (1.0 mL). To this solution, was added K_2CO_3 (13.5 mg, 0.10 mmol.) and the reaction mixture was stirred at 60 °C overnight. The residue was diluted with water, and the aqueous phase was extracted with EtOAc (2 × 10 mL). The combined organic phases were dried over magnesium sulfate, filtered, and concentrated *in vacuo*. Column chromatography (40% EtOAc in hexane) afforded **5** as an amorphous solid (12.0 mg, 75%); **IR** (Neat): ν_{max}/cm^{-1} 3408 (br, NH stretch), 2921, 1698 (C=O), 1495, 1455, 1339, 1229, 1010, 740, 698; **^{1}H NMR** (400 MHz, $CDCl_3$) δ 7.62 – 7.56 (m, 1H), 7.39 (dt, *J* = 8.1, 0.9 Hz, 1H), 7.31 – 7.25 (m, 4H), 7.24 – 7.11 (m, 5H), 7.08 (s, 1H), 4.33 (t, *J* = 7.2 Hz, 1H), 3.26 (d, *J* = 14.0 Hz, 1H), 3.05 (d, *J* = 14.0 Hz, 1H), 2.67 – 2.58 (m, 2H), 2.57 – 2.42 (m, 1H), 2.26 – 2.12 (m, 1H), 2.01 (s, 3H).; **^{13}C NMR** (101 MHz, $CDCl_3$) δ 204.4, 142.0, 136.4, 128.6, 128.5, 126.8, 126.0, 122.8, 122.7, 120.1, 119.8, 113.2, 111.5, 46.4, 33.9, 33.1, 15.8; **HRMS** (CI+) m/z: [M+H] Calcd for $C_{20}H_{22}NOS$ 324.1422; Found 324.1424.

6

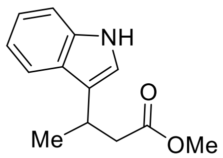

To the mixture of **4aa** (20.0 mg, 0.065 mmol) in MeOH (1.0 mL), was added K_2CO_3 (17.4 mg, 0.13 mmol) and stirred at rt overnight. The residue was diluted with water, and the aqueous phase was extracted with EtOAc (2 × 10 mL). The combined organic phases were dried over magnesium sulfate, filtered, and concentrated *in vacuo*. Column chromatography (40% EtOAc in hexane) afforded **6** as an amorphous solid (10.0 mg, 67%); **^{1}H NMR** (400 MHz, $CDCl_3$): δ 7.97 (s, 1H), 7.67 (dd, *J* = 8.0, 0.9 Hz, 1H), 7.35 – 7.32 (m, 1H), 7.23 – 7.17 (m, 1H), 7.12 (ddd, *J* = 7.5, 7.1, 1.1 Hz, 1H), 7.00 (d, *J* = 2.1 Hz, 1H), 3.66 (s, 3H), 3.64 – 3.56 (m, 1H), 2.84 (dd, *J* = 15.0, 6.1 Hz, 1H), 2.59 (dd, *J* = 15.0, 8.5 Hz, 1H), 1.43 (d, *J* = 6.9 Hz, 3H); **^{13}C NMR** (101 MHz, $CDCl_3$): δ 173.5, 136.6, 126.5, 122.2, 121.0, 120.1, 119.4, 119.3, 111.3, 51.7, 42.4, 28.1, 21.2; **HRMS** (ESI+): m/z $[M+H]^+$ Calcd for $C_{13}H_{16}NO_2$ 217.1097; Found 217.1100.

7aa

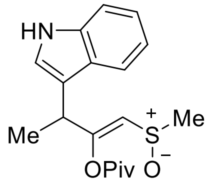

To the mixture of **3aa** (50.0 mg, 160 μmol) and H_2O_2 (160 μmol, 30%) in acetone:water (2:1, 1.0 mL:0.5 mL), was added $Mo_2O_2Cl_2$ (1.0 mg, 3.4 μmol). The reaction mixture was stirred for 18 h at rt. After full consumption of the starting material, the mixture was diluted with water and extracted with CH_2Cl_2. The combined organic phases were dried over magnesium sulfate, filtered, and concentrated *in vacuo*. Column chromatography (40% to 100% EtOAc in hexane) afforded **7aa** as an amorphous solid (33.0 mg, 63%, 1.2:1 mixture of diastereomers); Major isomer **^{1}H NMR** (400 MHz, $CDCl_3$) δ 8.56 (s, 1H), 7.59 (t, *J* = 7.9 Hz, 1H), 7.33 (d, *J* = 3.3 Hz, 1H), 7.16 – 7.14 (m, 1H), 7.08 (t, *J* = 7.4 Hz, 2H), 6.98 (d, *J* = 2.3 Hz, 1H), 5.84 (d, *J* = 1.3 Hz 1H), 3.90 (q, *J* = 7.1 Hz, 1H), 2.52 (s, 3H), 1.57 (d, *J* = 7.1 Hz, 3H), 1.25 (s, 9H); Minor isomer **^{1}H NMR** (400 MHz, $CDCl_3$) δ 8.56 (s, 1H), 7.59 (t, *J* = 7.9 Hz, 1H), 7.35 (d, *J* = 3.3 Hz, 1H), 7.20 – 7.16 (m, 1H), 7.08 (t, *J* = 7.4 Hz, 3H), 6.96 (d, *J* = 2.4 Hz, 1H), 5.99 (d, *J* = 1.3 Hz, 1H), 4.00 (q, *J* = 7.1 Hz, 1H), 2.61 (s, 3H), 1.53 (d, *J* = 7.1Hz, 3H), 1.18 (s, 9H); Both isomers **^{13}C NMR** (101 MHz, $CDCl_3$): δ 176.5, 176.1, 160.7, 160.1, 136.6, 136.5, 126.4, 126.3, 123.2, 123.0, 122.4, 122.3, 122.2, 119.8, 119.8, 119.5, 119.5, 115.0, 114.9, 111.6, 111.5, 111.4, 39.9 (2C), 39.5, 39.4, 36.0, 35.9, 27.1, 27.1, 18.2, 17.6; **HRMS** (ESI+): m/z [M+Na] Calcd for $C_{18}H_{23}NO_3SNa$ 356.1296; Found 356.1301.

8aa

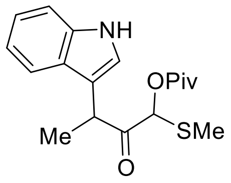

Compound **3aa** (20.0 mg, 0.06 mmol) was heated in $CHCl_3$ (1.0 mL) at 60°C. After 12 h, the reaction mixture was concentrated *in vacuo* and the crude residue purified with column chromatography (0% to 60% EtOAc in hexane) to afford **8aa** as an amorphous solid (12.4 mg, 62%, 1.2:1 mixture of diastereoisomers); Major isomer **^{1}H NMR** (400 MHz, $CDCl_3$) δ 8.16 (s, 1H), 7.73 (d, *J* = 7.8 Hz, 1H), 7.34 (s, 1H), 7.20 – 7.17 (m, 2H), 7.06 (d, *J* = 2.5 Hz, 1H), 6.09 (s, 1H), 4.49 (q, *J* = 7.2 Hz, 1H), 1.86 (s, 3H), 1.63 (d, *J* = 7.2 Hz, 3H), 1.24 (s, 9H). Minor isomer **^{1}H NMR** (400 MHz, $CDCl_3$) δ 8.20 (s, 1H), 7.68 (d, *J* = 7.8 Hz, 1H), 7.36 (s, 1H), 7.24 – 7.20 (m, 1H), 7.18 – 7.10 (m, 2H), 6.02 (s, 1H), 4.69 (q, *J* = 7.2 Hz, 1H), 2.08 (s, 3H), 1.52 (d, *J* = 7.2 Hz, 3H), 1.21 (s, 9H); Both isomers **^{13}C NMR** (101 MHz, $CDCl_3$) δ 201.3, 200.1, 177.8, 177.2, 136.3, 136.2, 126.7, 126.5, 122.64, 122.60, 122.5, 122.3, 120.0, 119.8, 119.1, 118. 8, 114.6, 114.4, 111.6, 111.4, 77.6, 76.8, 39.9, 39.10, 39.08, 38.2, 27.2, 27.1, 18.9, 17.4, 11.5, 11.4; **HRMS** (ESI+): m/z [M+Na] Calcd for $C_{18}H_{23}NO_4SNa$ 356.1296; Found 356.1304.

8ad

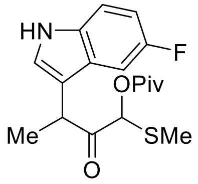

To the mixture of **3ad** (45.0 mg, 134 μmol) and H_2O_2 (15.2 μL, 134 μmol, 30%) in acetone:water (2:1, 1 mL:0.5 mL), was added $Mo_2O_2Cl_2$ (0.40 mg, 0.14 μmol). The reaction mixture was stirred for 18 h at rt. After full consumption of the starting material, the mixture was diluted with water and extracted with CH_2Cl_2. The combined organic phases were dried over magnesium sulfate, filtered, and concentrated *in vacuo*. The crude reaction mixture was used without further purification. The crude sulfoxide was heated in $CHCl_3$ (1.0 mL) at 60°C for 12 h without further purification to give rearranged product **8ad** as a colorless amorphous solid (13.2 mg, 28% over two steps as a 1.5:1 mixture of diastereomers); Major isomer **^{1}H NMR** (400 MHz, $CDCl_3$) for major isomer: δ 8.22 (s, 1H), 7.37 (dd, *J* = 9.6, 2.4 Hz, 1H), 7.25 – 7.21 (m, 1H), 6.95 – 6.92 (m, 1H), 6.06 (s, 1H), 5.97 (s, 1H), 4.40 (q, *J* = 7.5 Hz, 1H), 4.40 (q, *J* = 7.3 Hz, 1H), 1.85 (s, 3H), 1.60 (d, *J* = 7.5 Hz, 3H), 1.22 (s, 9H); **^{13}C NMR** (101 MHz, $CDCl_3$) δ 201.1, 177.8, 158.0 (d, $J_{C\text{-}F}$ = 236.0 Hz), 132.7, 127.0 (d, $J_{C\text{-}F}$ = 10.1 Hz), 124.4, 114.8 (d, $J_{C\text{-}F}$ = 4.8 Hz), 112.0 (d, $J_{C\text{-}F}$ = 9.8 Hz), 110.8 (d, $J_{C\text{-}F}$ = 26.6 Hz), 104.1 (d, $J_{C\text{-}F}$ = 23.9 Hz), 77.4, 39.8, 39.1, 27.1, 18.7, 11.4; Minor isomer **^{1}H NMR** (400 MHz, $CDCl_3$) for: δ 8.17 (s, 1H),, 7.29 (dd, *J* = 9.6, 2.4 Hz, 1H), 7.22 – 7.19 (m, 2H), 6.91 – 6.88 (m, 1H), 5.97 (s, 1H), 4.55 (q, *J* = 7.1 Hz, 1H), 2.06 (s, 3H), 1.48 (d, *J* = 7.1 Hz, 3H), 1.20 (s, 9H);

^{13}**C NMR** (101 MHz, $CDCl_3$) δ 200.0, 177.4, 158.0 (d, $J_{C\text{-}F}$ = 236.0 Hz) 132.8, 126.9 (d, $J_{C\text{-}F}$ = 10.1 Hz), 124.4, 114.5 (d, $J_{C\text{-}F}$ = 4.8 Hz), 112.2 (d, $J_{C\text{-}F}$ = 9.8 Hz), 110.9 (d, $J_{C\text{-}F}$ = 26.6 Hz), 103.8 (d, $J_{C\text{-}F}$ = 23.9 Hz), 76.7, 39.1, 38.7, 27.1, 17.3, 11.4; **HRMS** (ESI+): m/z [M+Na] Calcd for $C_{18}H_{22}FNO_3SNa$ 374.1202; Found 374.1203.

8da

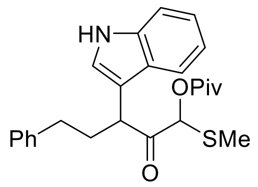

To the mixture of **3da** (40.0 mg, 98 μmol) and H_2O_2 (11.2 μL, 98 μmol, 30%) in acetone:water (2:1, 1 mL:0.5 mL), was added $Mo_2O_2Cl_2$ (0.30 mg, 0.09 μmol). The reaction mixture was stirred for 18 h at rt. After full consumption of the starting material, the mixture was diluted with water and extracted with CH_2Cl_2. The combined organic phases were dried over magnesium sulfate, filtered, and concentrated *in vacuo*. The crude reaction mixture was used without further purification. The crude sulfoxide was heated in $CHCl_3$ (1.0 mL) at 60°C for 12 h without further purification to give rearranged product **8da** an amorphous solid (11.0 mg, 26% over two steps, inseparable 1.7:1 mixture of diastereoisomers); Major isomer ^{1}**H NMR** (400 MHz, $CDCl_3$) δ 8.19 (s, 1H), 7.71 (d, *J* = 8.0 Hz, 1H), 7.35 (d, *J* = 1.2 Hz, 1H), 7.24 – 7.18 (m, 3H), 7.17 (dd, *J* = 3.0, 1.5 Hz, 1H), 7.16 – 7.13 (m, 3H), 7.05 (d, *J* = 2.5 Hz, 1H), 6.02 (s, 1H), 4.36 (m, 1H), 2.72 – 2.58 (m, 2H), 2.31 – 2.13 (m, 2H), 1.72 (s, 3H), 1.24 (s, 9H); Both isomers ^{13}**C NMR** (101 MHz, $CDCl_3$) δ 200.8, 199.0, 177.7, 177.0, 141.9, 141.5, 136.4, 136.2, 128.7, 128.6, 128.5, 128.5, 127.0, 126.8, 126.1, 126.0, 123.2, 123.0, 122.5, 122.3, 120.0, 119.8, 119.4, 118.9, 112.8, 112.4, 111.6, 111.3, 78.6, 76.7, 44.8, 43.9, 39.10, 39.07, 35.3, 33.7, 33.7, 33.3, 27.1 (st), 11.6, 11.5; **HRMS** (ESI+): m/z [M+Na] Calcd for $C_{25}H_{29}NO_3SNa$ 446.1766; Found 446.1767.

9

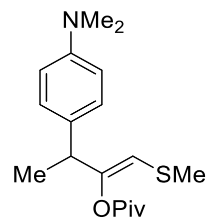

Prepared according to **GP3** using **1a** (40.0 mg, 0.20 mmol, *N*,*N*-dimethyl aniline (84.8 mg, 0.70 mmol), and IPrAu(CH_3CN)SbF_6 (8.6 mg, 0.01 mmol); column chromatography (10% EtOAc in hexane) afforded **9** as a colorless liquid (25.0 mg, 60%); ^{1}**H NMR** (400 MHz, $CDCl_3$) δ 7.09 (d, *J* = 8.7 Hz, 2H), 6.69 (d, *J* = 8.7 Hz, 2H), 5.48 (d, *J* = 1.3 Hz, 1H), 3.62 (q, *J* = 7.3 Hz, 1H), 2.92 (s, 6H), 2.21 (s, 3H), 1.37 (d, *J* = 7.3 Hz, 3H), 1.20 (s, 9H); ^{13}**C NMR** (101 MHz, $CDCl_3$) δ 175.7, 150.9, 149.7, 130.6, 128.5, 113.7, 112.8, 42.6, 40.9, 39.3, 27.3, 19.2, 17.0. HRMS (ESI+): m/z [M+H] Calcd for $C_{18}H_{28}NO_2S$ 322.1841; Found 322.1848.

(*S*)-4-(Methylthio)but-3-yn-2-yl pivalate (*S*-1a)

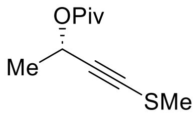

Prepared according to **GP2** using (S)-4-(methylthio)but-3-yn-2-ol (532 mg, 4.60 mmol) which was prepared according to **GP1** from commercially available (S)-but-3-yn-2-ol; column chromatography (5% EtOAc in hexane) afforded **1a** as a colorless liquid (1.45 g, 76%); **IR** (Neat): ν_{max}/cm^{-1} 2975, 2933, 2874, 2170, 1728 (C=O), 1277, 1151, 1068, 1039, 955, 855, 771; **^{1}H NMR** (400 MHz, $CDCl_3$) δ 2.36 (s, 3H), 1.63 (s, 6H), 1.17 (s, 9H); **^{13}C NMR** (101 MHz, $CDCl_3$) δ 176.8, 94.0, 76.2, 72.2, 39.2, 29.0, 27.2, 19.4; **HRMS** (ESI+) m/z: [M+Na] Calcd for $C_{10}H_{16}O_2SNa$ 223.0769; Found 223.0775.

(*rac*)-3aa

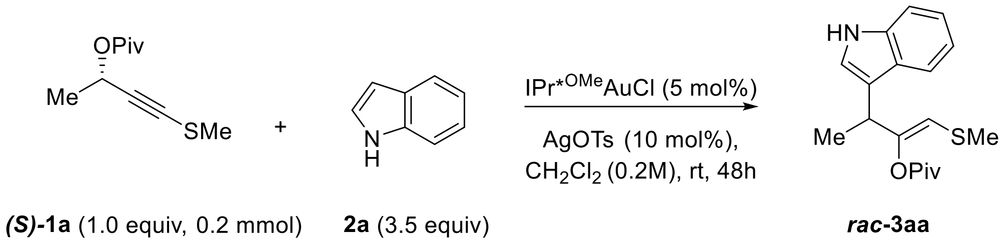

To the mixture of **(*S*)-1a** (40.1 mg, 0.20 mmol) and indole **2a** (82.0 mg, 0.70 mmol), dissolved in anhydrous CH_2Cl_2 (1.0 mL) in a heat-gun dried Schlenk tube under argon, was added $IPr^{*OMe}AuCl$ (11.2 mg, 0.01 mmol) followed by AgOTs (5.6 mg, 0.02 mmol). The solution was stirred at rt for 18 h. The reaction mixture was filtered through a pad of celite and the celite was washed with EtOAc (3 × ~1 mL). The solution was concentrated under reduced pressure and the product was purified by flash column chromatography (10 g silica cartridge, eluted with 0 to 10% EtOAc in hexane) to obtain product (***rac***)-**3aa** (46.0 mg, 71%). HPLC data showed this to be a racemate.

HPLC traces for chirality transfer test

Racemic starting material 1a

Column: IB; Mobile phase: IPA/Hexane (0:100); 0.75 mL/minute

```
Data File C:\Chem32\3\Data\Nagnath\NMM_5_10_2022 2022-10-10 12-54-17\RJM--0014.D
Sample Name: SM

    ====================================================================
    Acq. Operator   : SYSTEM                        Seq. Line :    4
    Acq. Instrument : LC-FC                          Location :    15
    Injection Date  : 10-Oct-22 1:45:27 PM                Inj :    4
                                                   Inj Volume : 5.000 µl
    Method          : C:\Chem32\3\Data\Nagnath\NMM_5_10_2022 2022-10-10 12-54-17\NM_SM_IB_0%_
                      15min_0.75 mLmin.M (Sequence Method)
    Last changed    : 10-Oct-22 10:58:16 AM by SYSTEM
    Sample Info     : NM
```

Additional Info : Peak(s) manually integrated

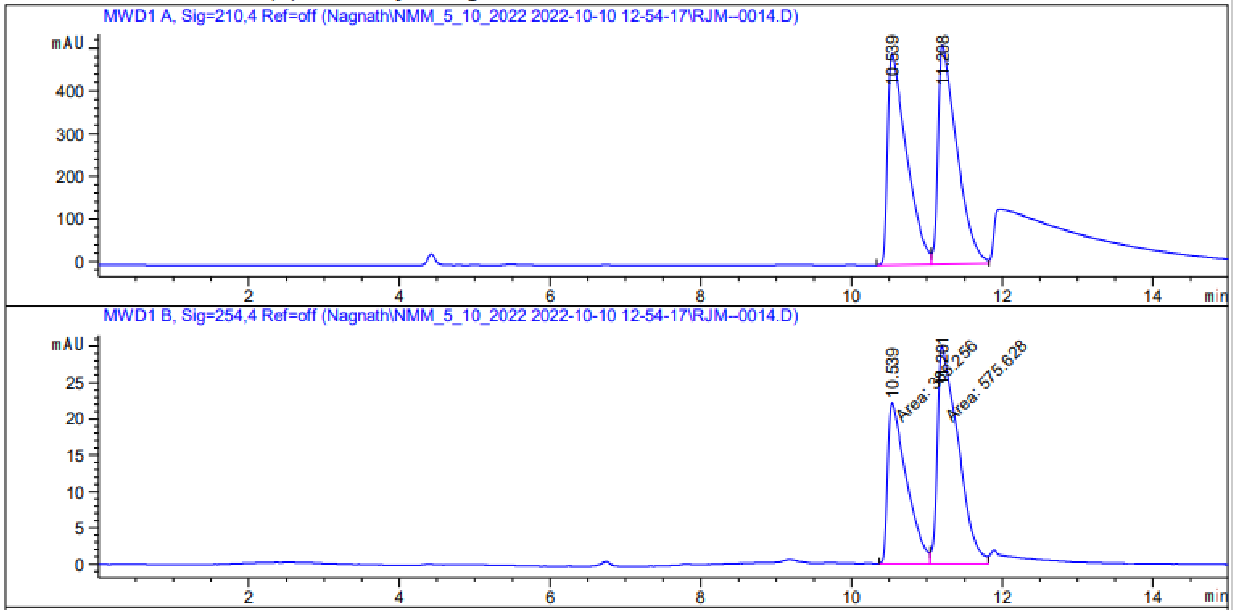

===
Fraction Information
===

Data File C:\Chem32\3\Data\Nagnath\NMM_5_10_2022 2022-10-10 12-54-17\RJM--0014.D
Sample Name: SM

No Fractions found.
===
===
==
Area Percent Report
==

Sorted By : Signal
Multiplier : 1.0000
Dilution : 1.0000
Use Multiplier & Dilution Factor with ISTDs

Signal 1: MWD1 A, Sig=210,4 Ref=off

Peak #	RetTime [min]	Type	Width [min]	Area [mAU*s]	Height [mAU]	Area %
1	10.539	BV	0.2556	8639.92383	494.97318	48.9349
2	11.208	VV	0.2509	9016.02734	513.35272	51.0651

Totals : 1.76560e4 1008.32590

Signal 2: MWD1 B, Sig=254,4 Ref=off

Peak #	RetTime [min]	Type	Width [min]	Area [mAU*s]	Height [mAU]	Area %
1	10.539	MF	0.2902	385.25601	22.12541	40.0939
2	11.201	FM	0.3198	575.62769	29.99543	59.9061

Totals : 960.88370 52.12083

Optically active starting material (*S*-1a):

Column: IB; Mobile phase: IPA/Hexane (0:100); 0.75 mL/minute

```
Data File C:\Chem32\3\Data\Nagnath\NMM_5_10_2022 2022-10-10 14-06-50\RJM--0012.D
Sample Name: SM_Chiral

    =====================================================================
    Acq. Operator   : SYSTEM                         Seq. Line :    2
    Acq. Instrument : LC-FC                           Location :   17
    Injection Date  : 10-Oct-22 2:25:03 PM                 Inj :    2
                                                    Inj Volume : 5.000 µl
    Method          : C:\Chem32\3\Data\Nagnath\NMM_5_10_2022 2022-10-10 14-06-50\NM_SM_IB_0%_
                      15min_0.75 mLmin.M (Sequence Method)
    Last changed    : 10-Oct-22 10:58:16 AM by SYSTEM
    Sample Info     : NM

  Additional Info : Peak(s) manually integrated
```

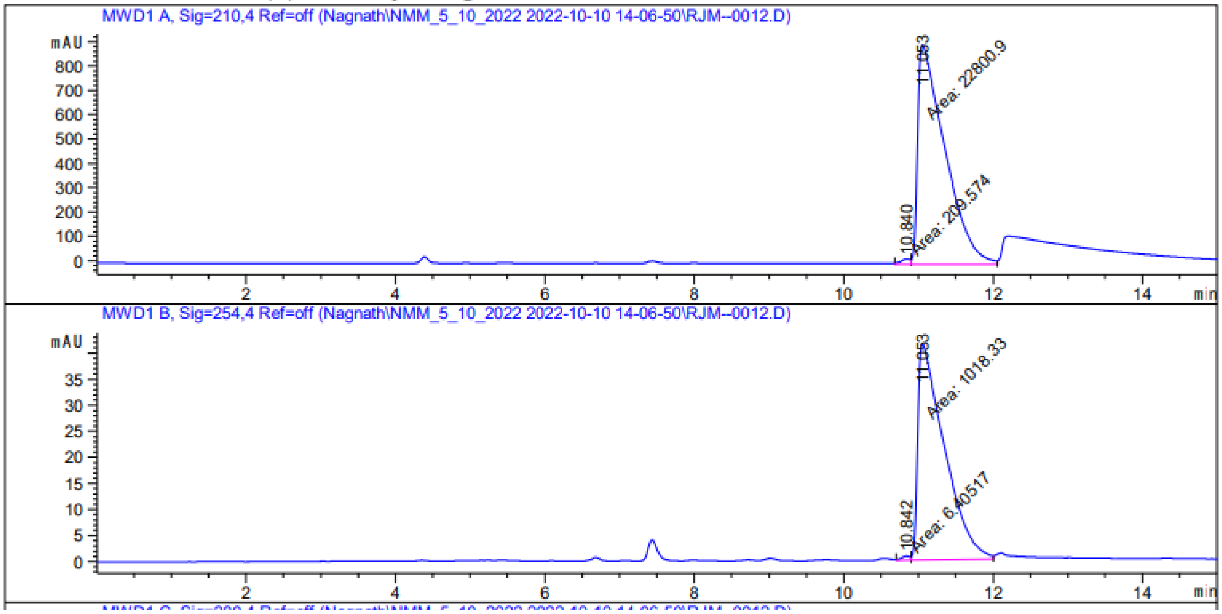

```
Data File C:\Chem32\3\Data\Nagnath\NMM_5_10_2022 2022-10-10 14-06-50\RJM--0012.D
Sample Name: SM_Chiral

    No Fractions found.
    =====================================================================
    =====================================================================
    =====================================================================
                             Area Percent Report
    =====================================================================

    Sorted By             :      Signal
    Multiplier            :      1.0000
    Dilution              :      1.0000
    Use Multiplier & Dilution Factor with ISTDs


    Signal 1: MWD1 A, Sig=210,4 Ref=off

    Peak RetTime Type  Width     Area      Height     Area
      #   [min]        [min]   [mAU*s]     [mAU]        %
    ----|-------|----|-------|----------|----------|--------|
       1  10.840 MF    0.1531  209.57407   22.82008    0.9108
       2  11.053 FM    0.4205 2.28009e4  903.68579  99.0892

    Totals :                  2.30105e4  926.50587
```

Product 3aa from reaction of (rac)-1a

Column: IB; Mobile phase: IPA/Hexane (5:95); 1 mL/minute

```
Data File C:\Chem32\3\Data\Nagnath\NMM_5_10_2022 2022-10-10 14-46-59\RJM--0014.D
Sample Name: 1,2

    =====================================================================
    Acq. Operator   : SYSTEM                         Seq. Line :   4
    Acq. Instrument : LC-FC                           Location :   16
    Injection Date  : 10-Oct-22 3:38:20 PM                 Inj :   4
                                                    Inj Volume : 5.000 µl
    Method          : C:\Chem32\3\Data\Nagnath\NMM_5_10_2022 2022-10-10 14-46-59\NM_Prod_IB_5%_
                      15min_1 mLmin.M (Sequence Method)
    Last changed    : 10-Oct-22 10:59:19 AM by SYSTEM
    Sample Info     : NM
```

Additional Info : Peak(s) manually integrated

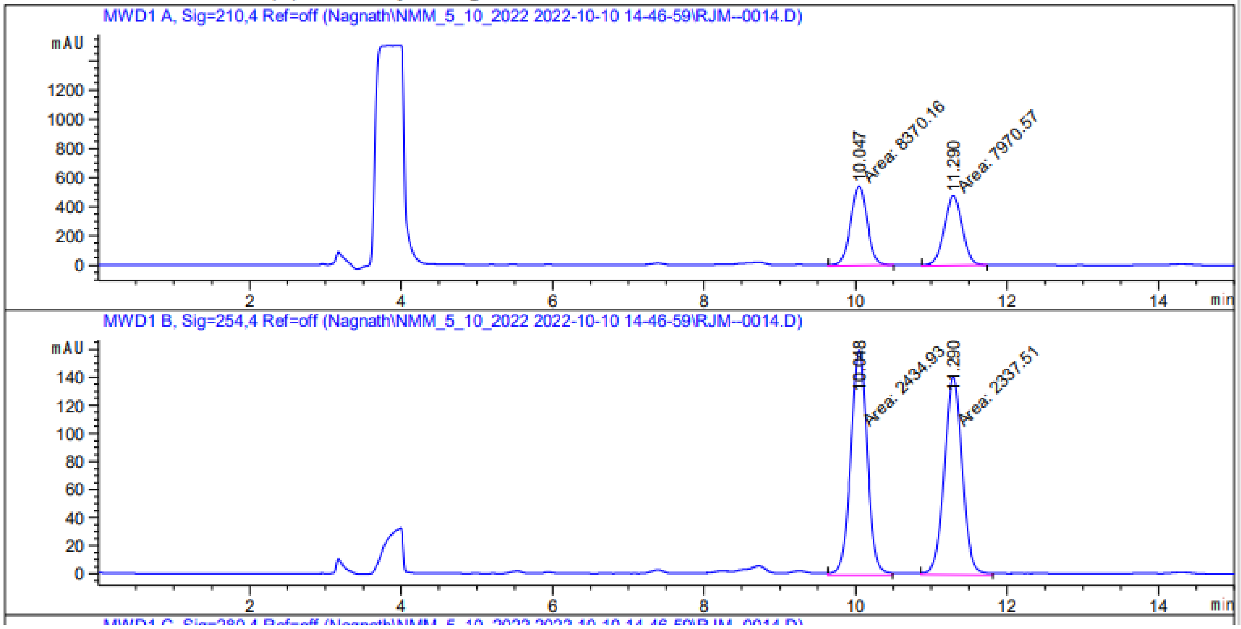

===
Fraction Information
===
No Fractions found.
===
===

C-FC 10-Oct-22 4:06:07 PM SYSTEM Page 1 of 2

ata File C:\Chem32\3\Data\Nagnath\NMM_5_10_2022 2022-10-10 14-46-59\RJM--0014.D
ample Name: 1,2

===
Area Percent Report
===

Sorted By : Signal
Multiplier : 1.0000
Dilution : 1.0000
Use Multiplier & Dilution Factor with ISTDs

Signal 1: MWD1 A, Sig=210,4 Ref=off

Peak #	RetTime [min]	Type	Width [min]	Area [mAU*s]	Height [mAU]	Area %
1	10.047	MM	0.2556	8370.16016	545.72552	51.2227
2	11.290	MM	0.2770	7970.57178	479.60681	48.7773

Totals : 1.63407e4 1025.33234

Product 3aa from reaction of (*S*)-1a

Column: IB; Mobile phase: IPA/Hexane (5:95); 1 mL/minute

Data File C:\Chem32\3\Data\Nagnath\NMM_5_10_2022 2022-10-10 14-46-59\RJM--0021.D
Sample Name: 1,2_Chiral

===
Acq. Operator : SYSTEM Seq. Line : 5
Acq. Instrument : LC-FC Location : 18
Injection Date : 10-Oct-22 3:54:51 PM Inj : 1
Inj Volume : 5.000 µl
Method : C:\Chem32\3\Data\Nagnath\NMM_5_10_2022 2022-10-10 14-46-59\NM_Prod_IB_5%_15min_1 mLmin.M (Sequence Method)
Last changed : 10-Oct-22 10:59:19 AM by SYSTEM
Sample Info : NM

Additional Info : Peak(s) manually integrated

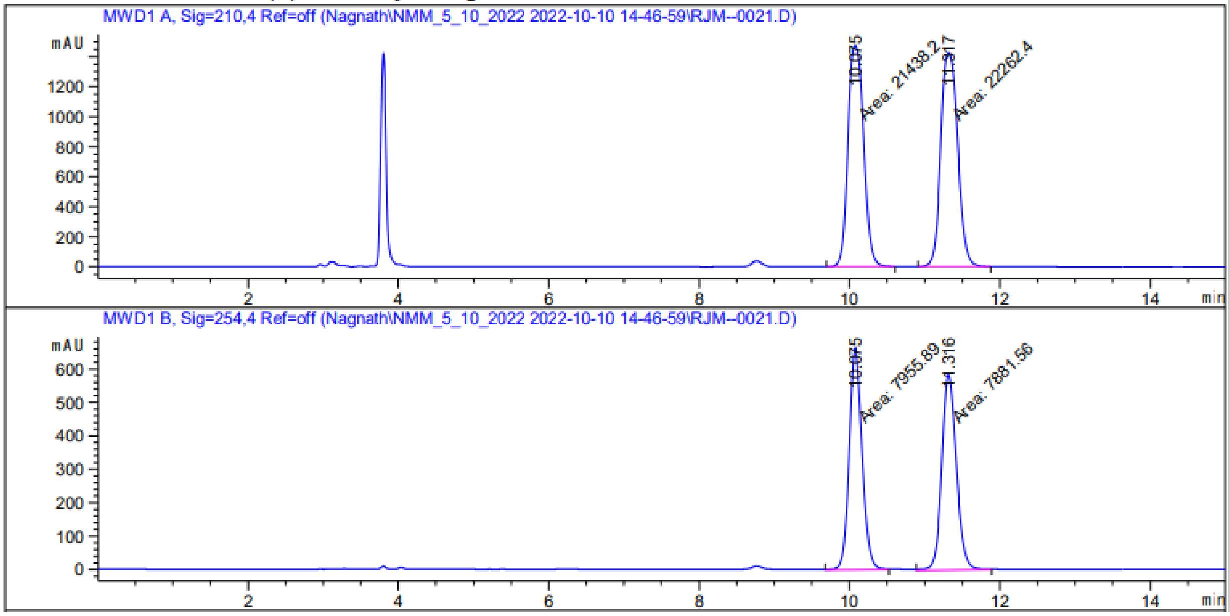

===

Fraction Information

===

No Fractions found.

===

===

Data File C:\Chem32\3\Data\Nagnath\NMM_5_10_2022 2022-10-10 14-46-59\RJM--0021.D
Sample Name: 1,2_Chiral

===

Area Percent Report

===

Sorted By : Signal
Multiplier : 1.0000
Dilution : 1.0000
Use Multiplier & Dilution Factor with ISTDs

Signal 1: MWD1 A, Sig=210,4 Ref=off

Peak #	RetTime [min]	Type	Width [min]	Area [mAU*s]	Height [mAU]	Area %
1	10.075	MM	0.2421	2.14382e4	1475.96008	49.0570
2	11.317	MM	0.2599	2.22624e4	1427.85681	50.9430

Totals : 4.37005e4 2903.81689

Crystallographic details

The datasets for **3ac** and **4aa** were measured on an Agilent SuperNova diffractometer using an Atlas detector. The data collections were driven and processed and absorption corrections were applied using CrysAlisPro.[4] Both structures were solved using ShelXT[5] and refined by a full-matrix least-squares procedure on F^2 in ShelXL.[6] Figures and reports were produced using OLEX2.[7] All non-hydrogen atoms were refined with anisotropic displacement parameters. In both structures the hydrogen atom bonded to N(1) was located in the electron density and freely refined. All remaining hydrogen atoms in both structures were fixed as riding models and the isotropic thermal parameters (U_{iso}) were based on the U_{eq} of the parent atoms.

Both structures occupy centrosymmetric space groups such that there is a racemic mixture of enantiomers at C(1) in the unit cell.

Crystal data for **3ac** (Crystals were grown from ethyl acetate solution layered with hexane): $C_{18}H_{22}ClNO_2S$ (M =351.87 g/mol): monoclinic, space group $P2_1/c$ (no. 14), a = 14.4619(5) Å, b = 9.4257(2) Å, c = 14.6087(5) Å, β = 113.408(4)°, V = 1827.47(11) Å^3, Z = 4, T = 99.97(13) K, μ(Cu Kα) = 2.982 mm^{-1}, *Dcalc* = 1.279 g/cm^3, 33623 reflections measured (11.476° ≤ 2Θ ≤ 146.058°), 3641 unique (R_{int} = 0.0487, R_{sigma} = 0.0200) which were used in all calculations. The final R_1 was 0.0323 (I > 2σ(I)) and wR_2 was 0.0826 (all data).

Crystal data for **4aa**: (Crystals were grown from pentane diffusion into a saturated $CDCl_3$ solution): $C_{18}H_{23}NO_2S$ (M =317.43 g/mol): monoclinic, space group $P2_1/c$ (no. 14), a = 12.7478(6) Å, b = 13.4259(6) Å, c = 10.4584(5) Å, β = 104.619(5)°, V = 1732.01(14) Å^3, Z = 4, T = 100(2) K, μ(Cu Kα) = 1.705 mm^{-1}, *Dcalc* = 1.217 g/cm^3, 6710 reflections measured (7.166° ≤ 2Θ ≤ 145.81°), 3360 unique (R_{int} = 0.0257, R_{sigma} = 0.0321) which were used in all calculations. The final R_1 was 0.0409 (I > 2σ(I)) and wR_2 was 0.1062 (all data).

CCDC 2374345-2374346 (**4aa** and **3ac** respectively) contain the supplementary crystallographic data for this paper. These data can be obtained free of charge from The Cambridge Crystallographic Data Centre via www.ccdc.cam.ac.uk/data_request/cif.

Thermal ellipsoid plot for 3ac (shown at 50% probability levels)

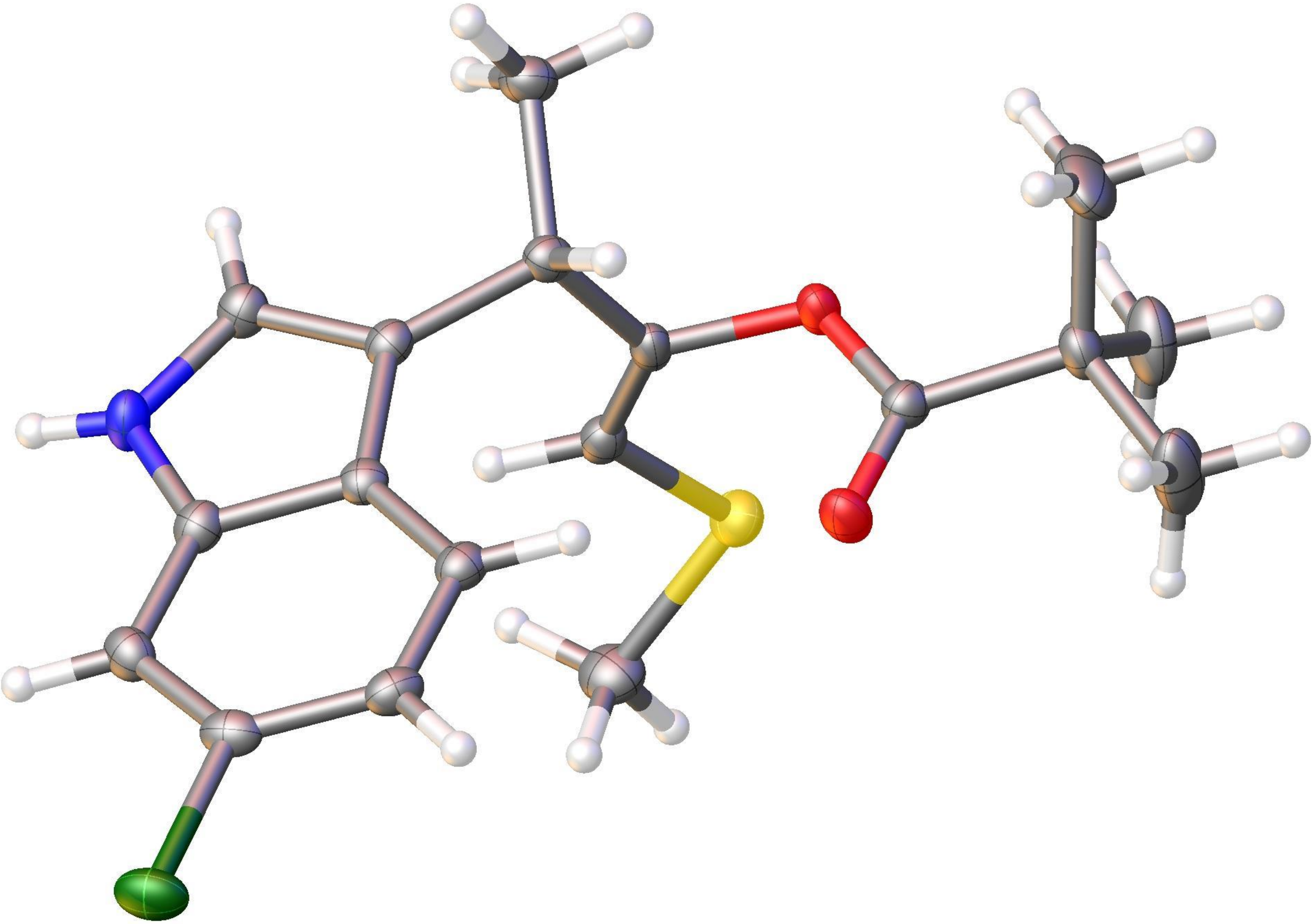

Thermal ellipsoid plot for 4aa (shown at 50% probability levels)

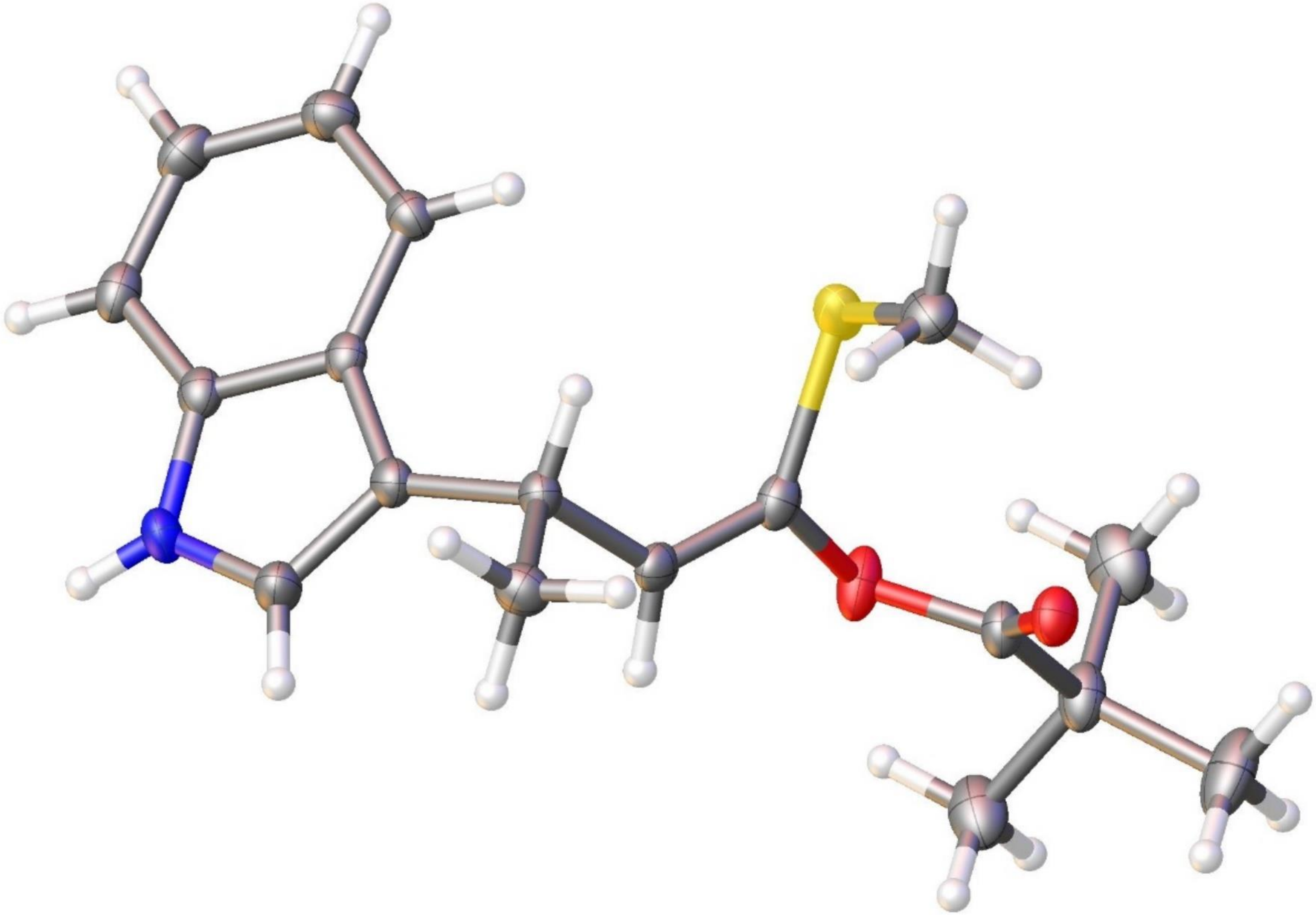

^{1}H NMR and ^{13}C NMR Spectra

TA1 ^{1}H NMR (400 MHz, $CDCl_3$) & ^{13}C NMR (101 MHz, $CDCl_3$):

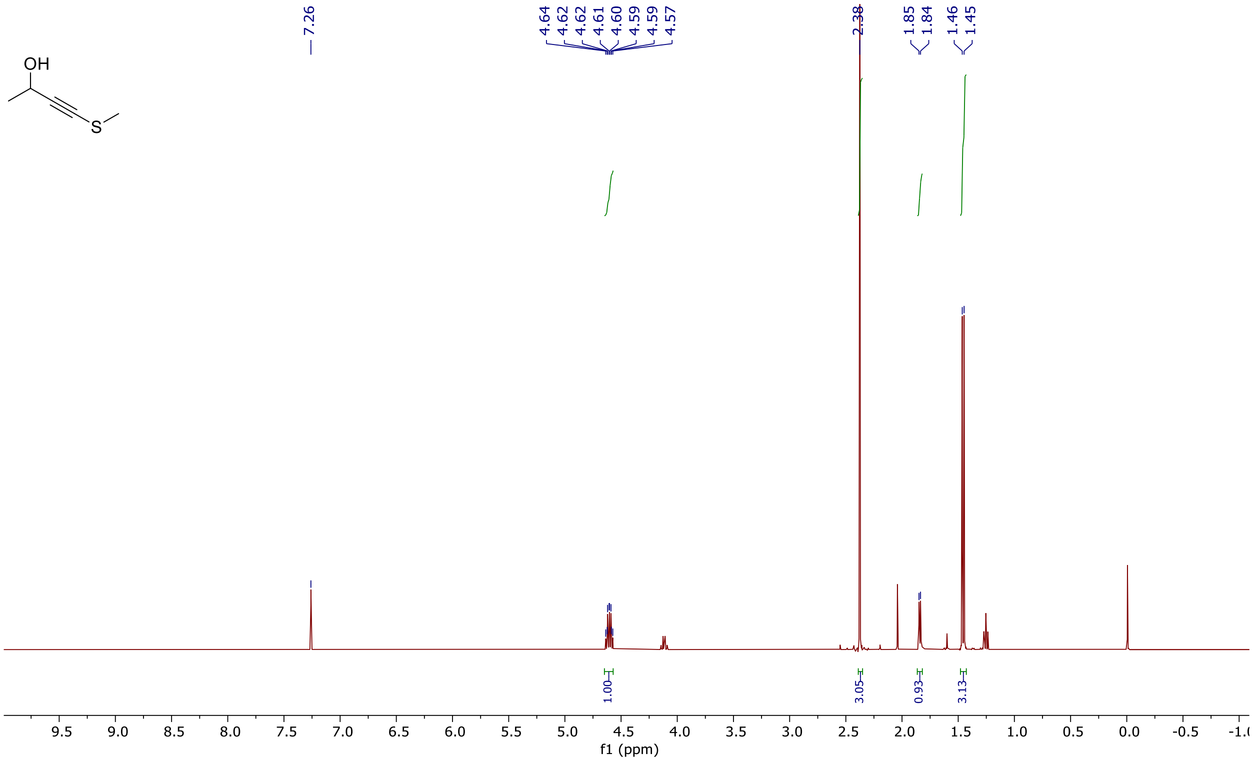

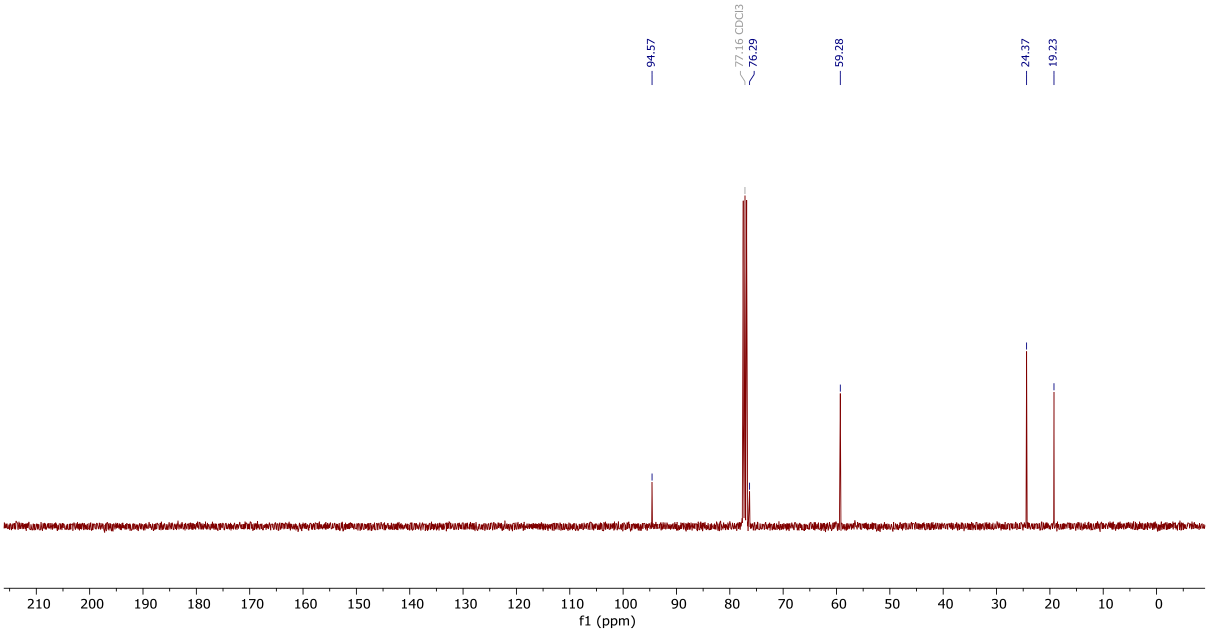

TA2 ^{1}H NMR (400 MHz, $CDCl_3$) & ^{13}C NMR (101 MHz, $CDCl_3$):

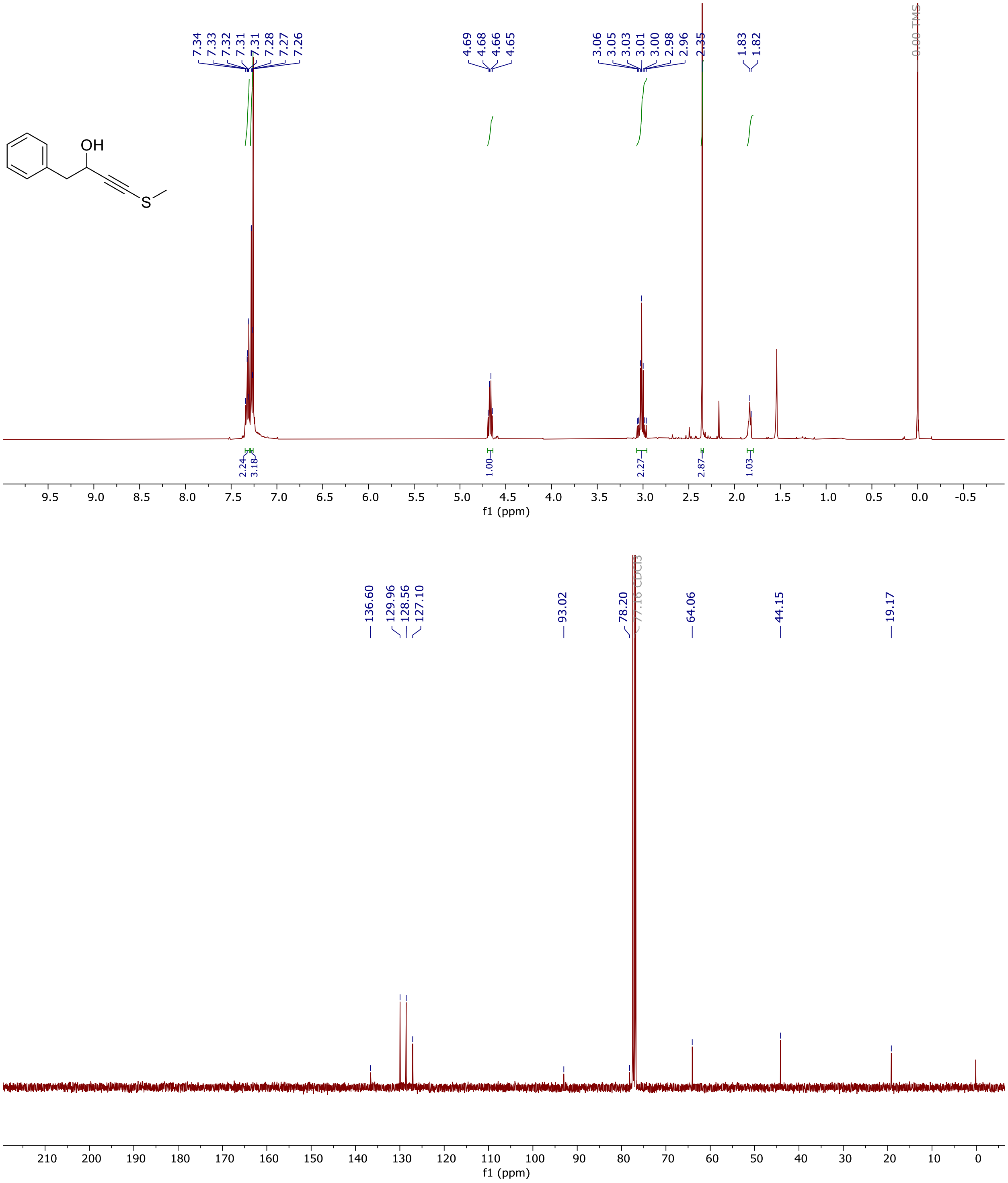

TA3 ^{1}H NMR (400 MHz, $CDCl_3$) & ^{13}C NMR (101 MHz, $CDCl_3$):

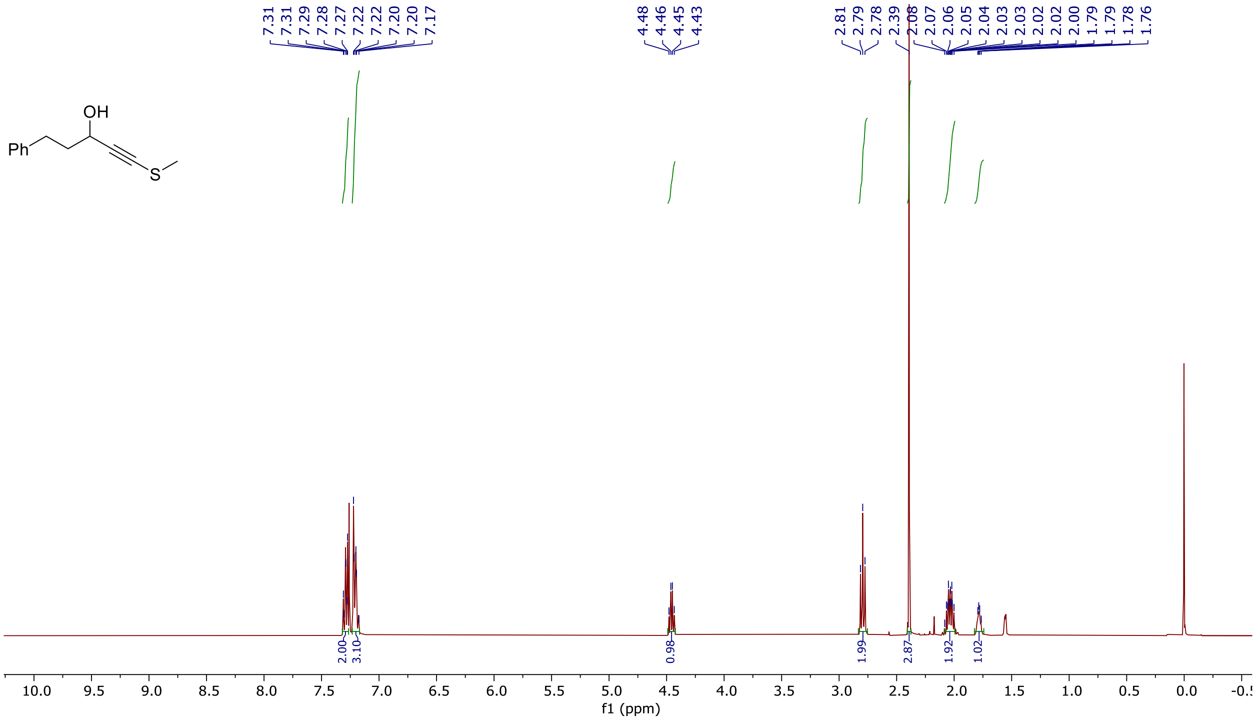

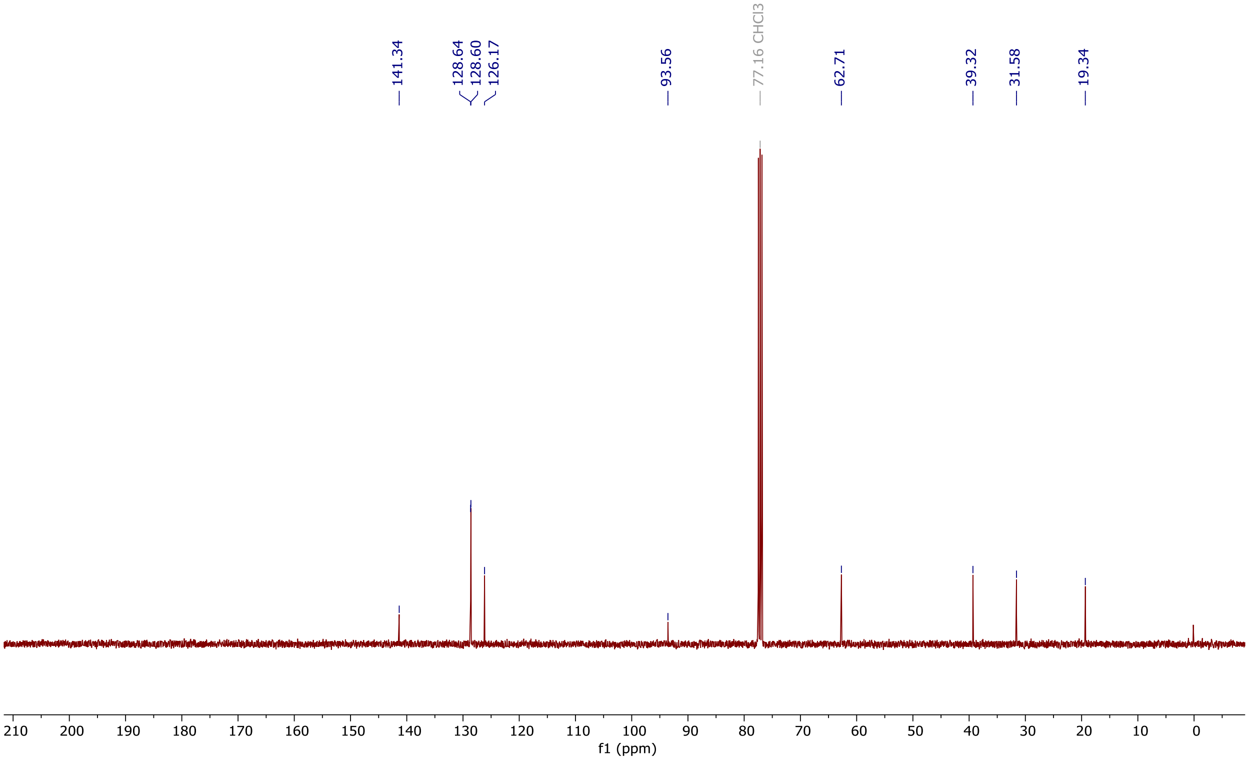

TA4 ^{1}H NMR (400 MHz, $CDCl_3$) & ^{13}C NMR (101 MHz, $CDCl_3$):

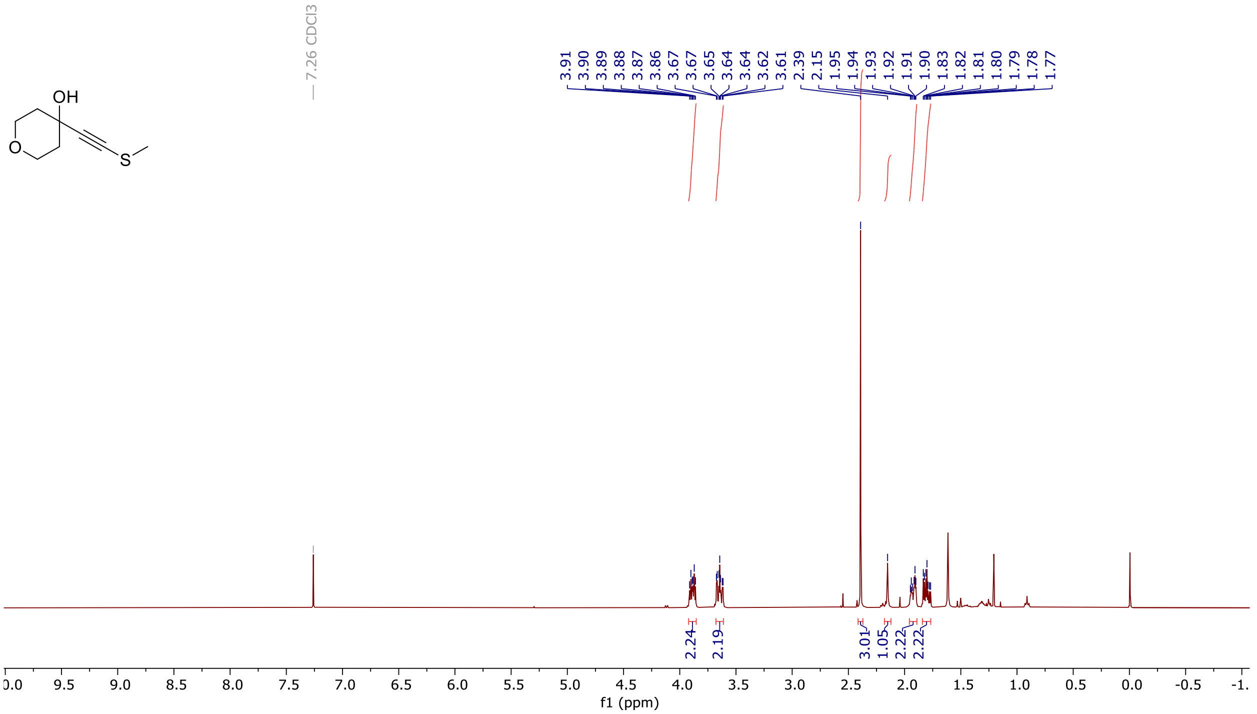

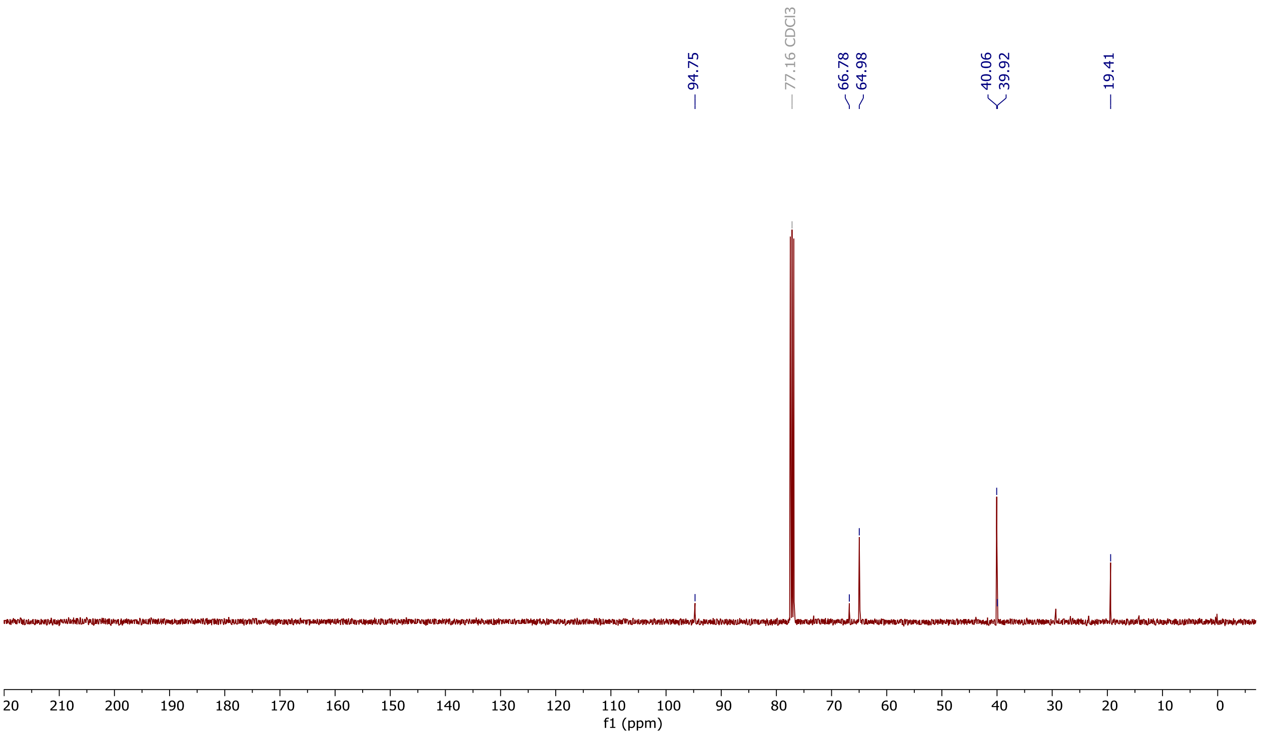

TA5 ^{1}H NMR (400 MHz, $CDCl_3$) & ^{13}C NMR (101 MHz, $CDCl_3$):

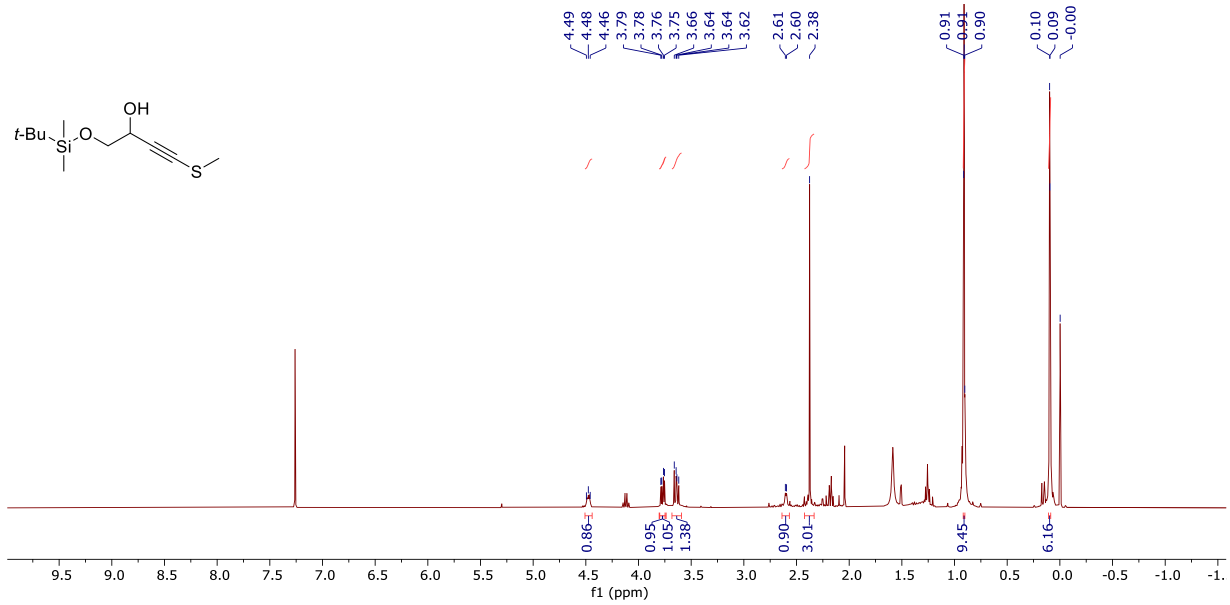

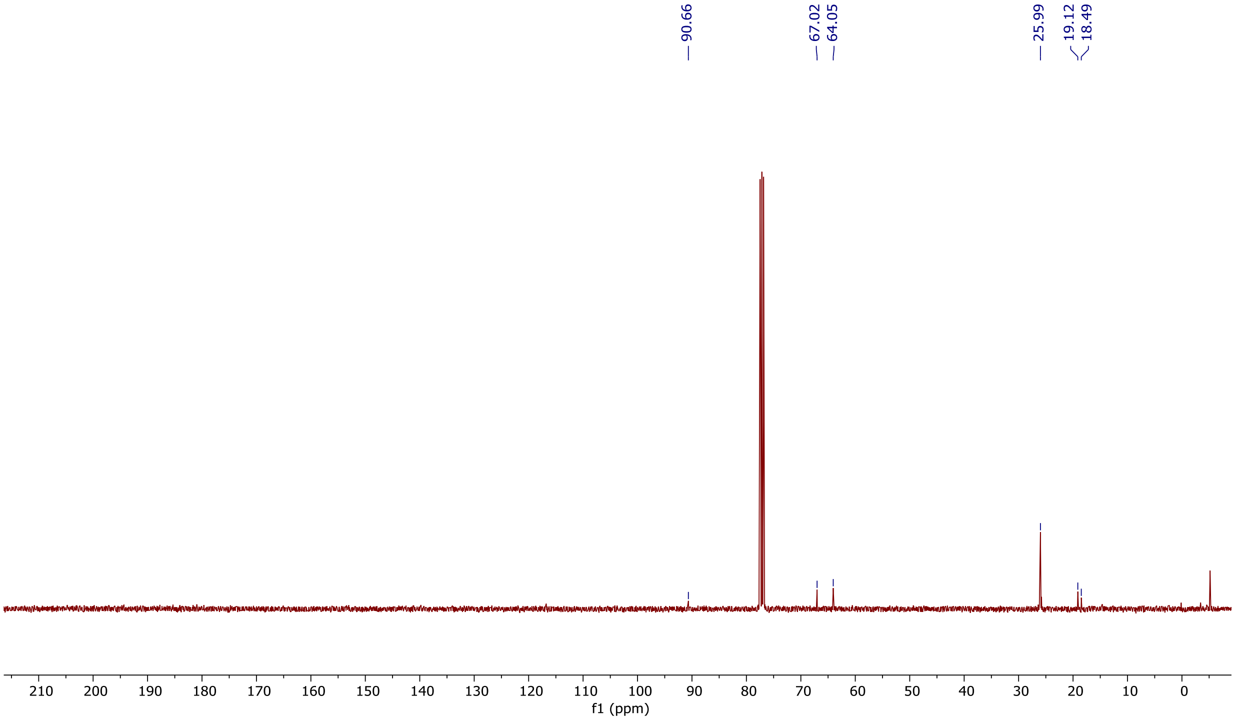

TA6 ^{1}H NMR (400 MHz, $CDCl_3$) & ^{13}C NMR (101 MHz, $CDCl_3$):

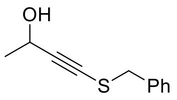

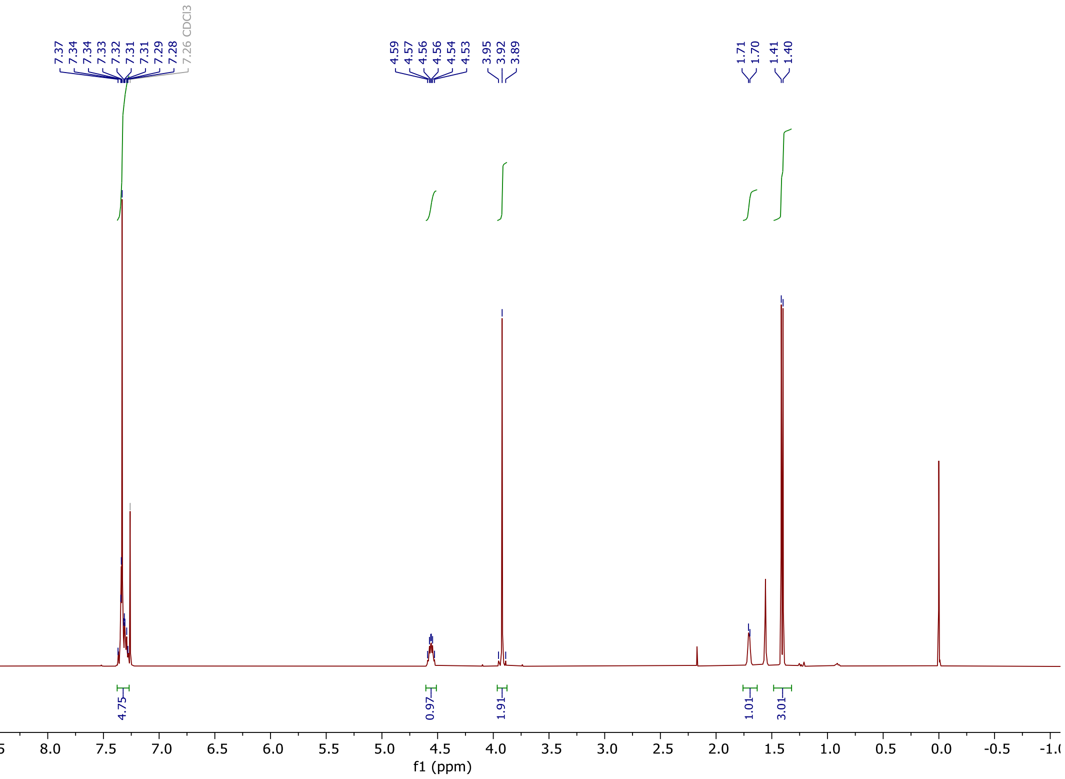

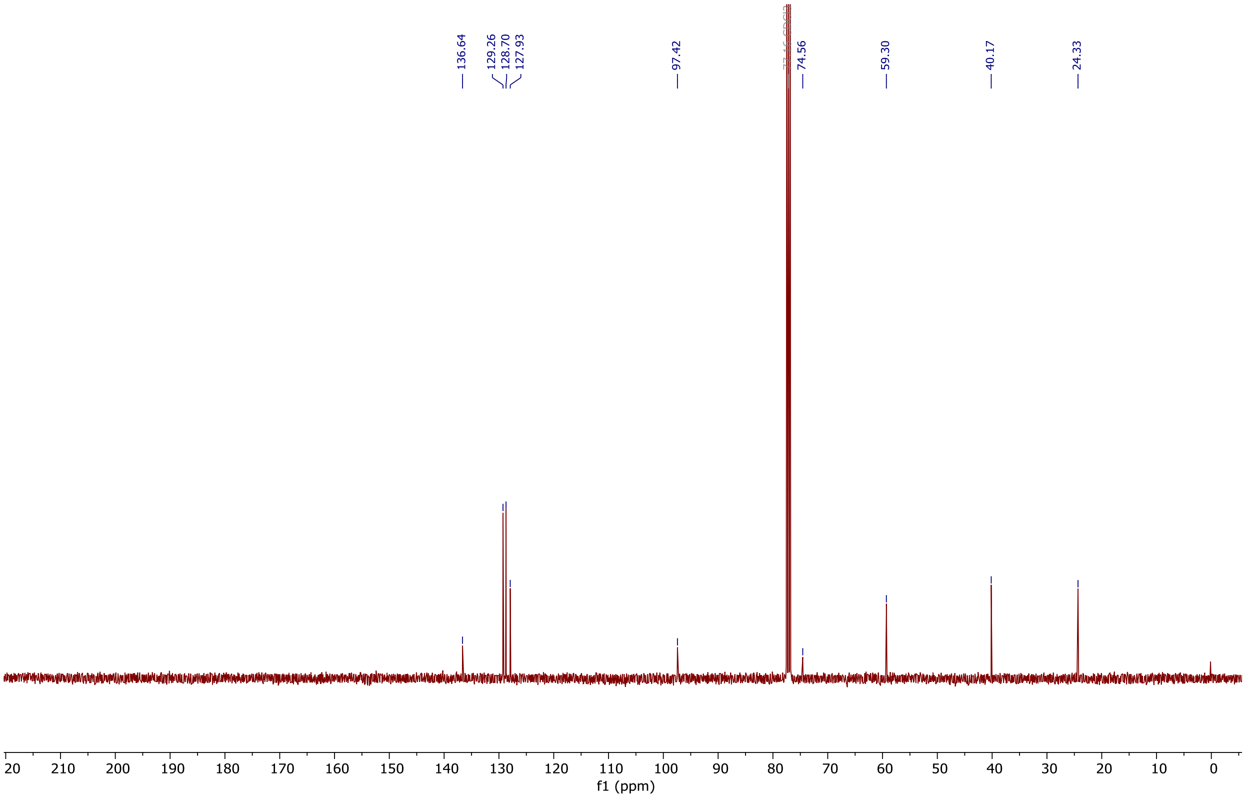

1a ^{1}H NMR (400 MHz, $CDCl_3$) & ^{13}C NMR (101 MHz, $CDCl_3$):

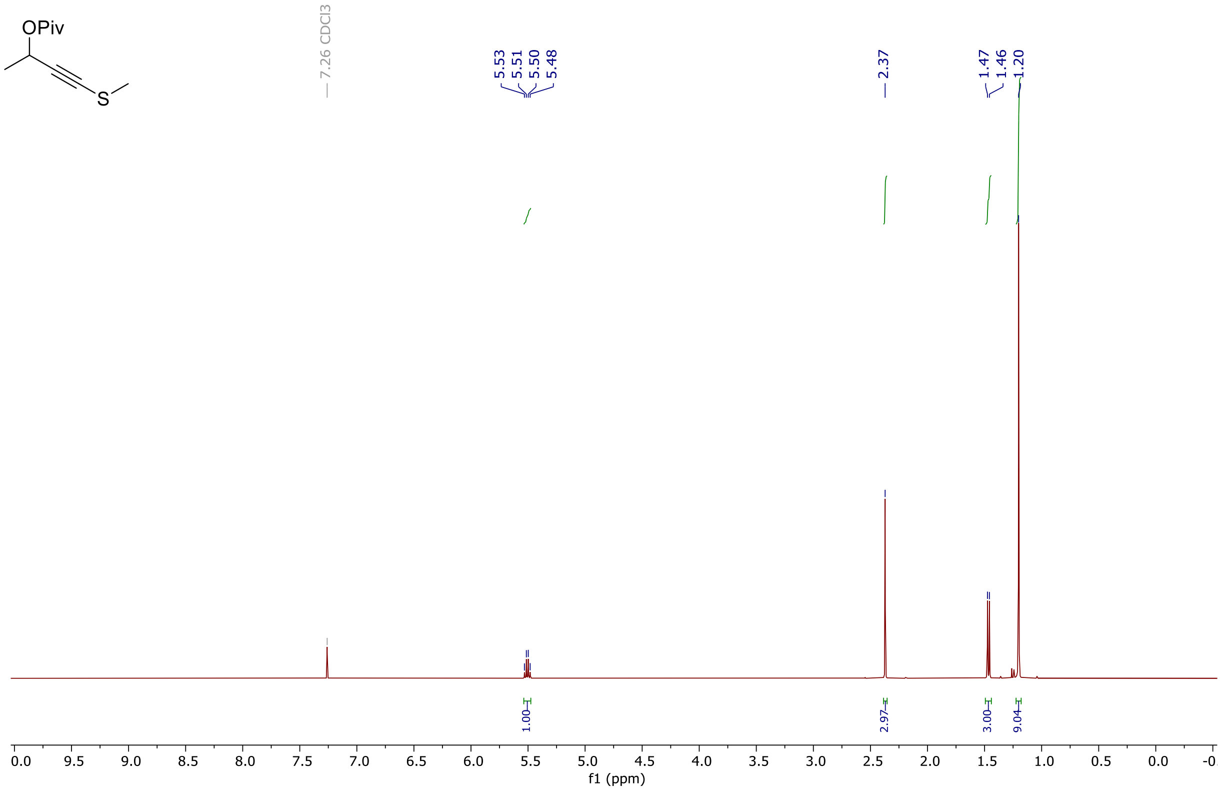

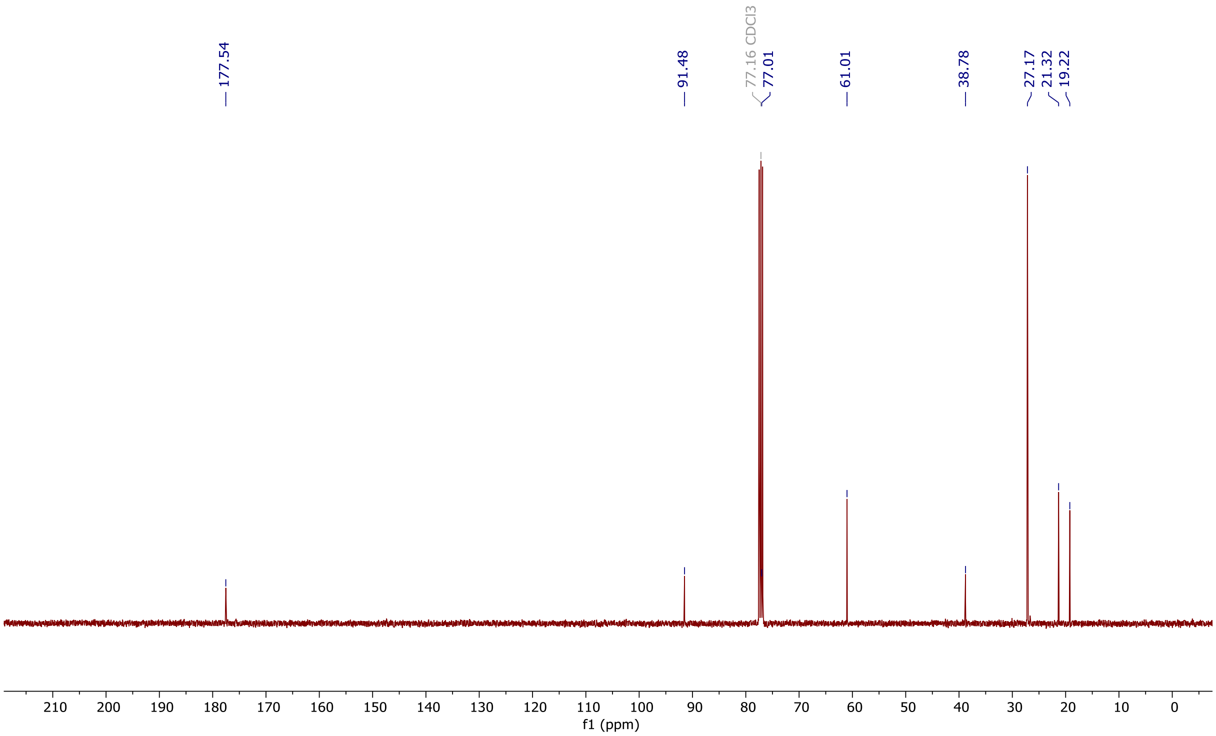

1b ^{1}H NMR (300 MHz, $CDCl_3$) & ^{13}C NMR (101 MHz, $CDCl_3$):

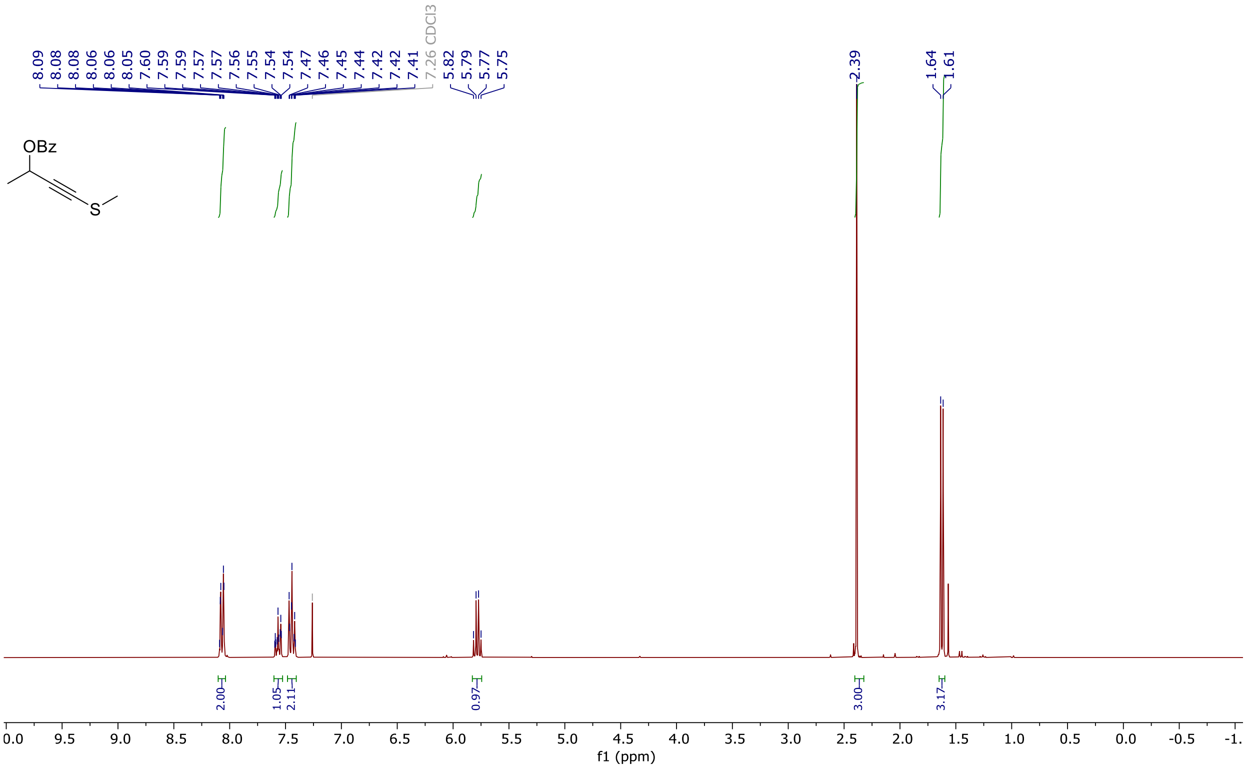

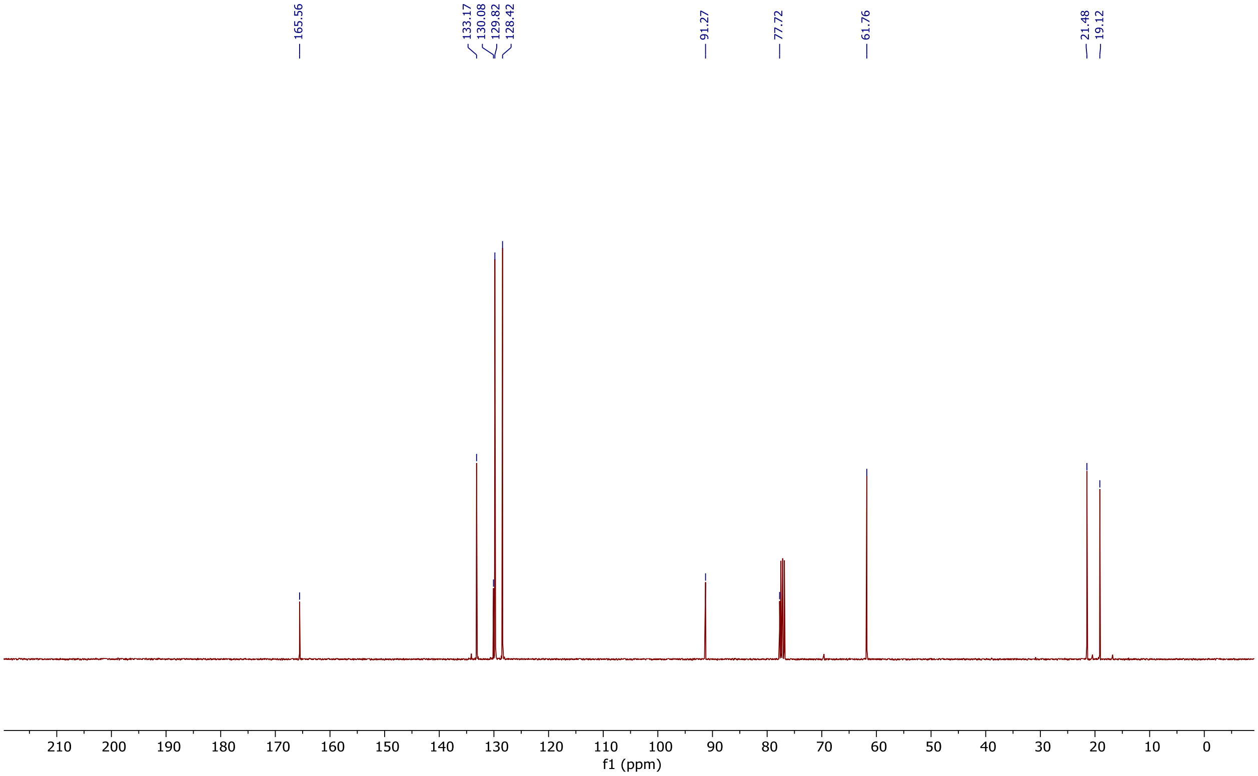

1c ^{1}H NMR (400 MHz, $CDCl_3$) & ^{13}C NMR (101 MHz, $CDCl_3$):

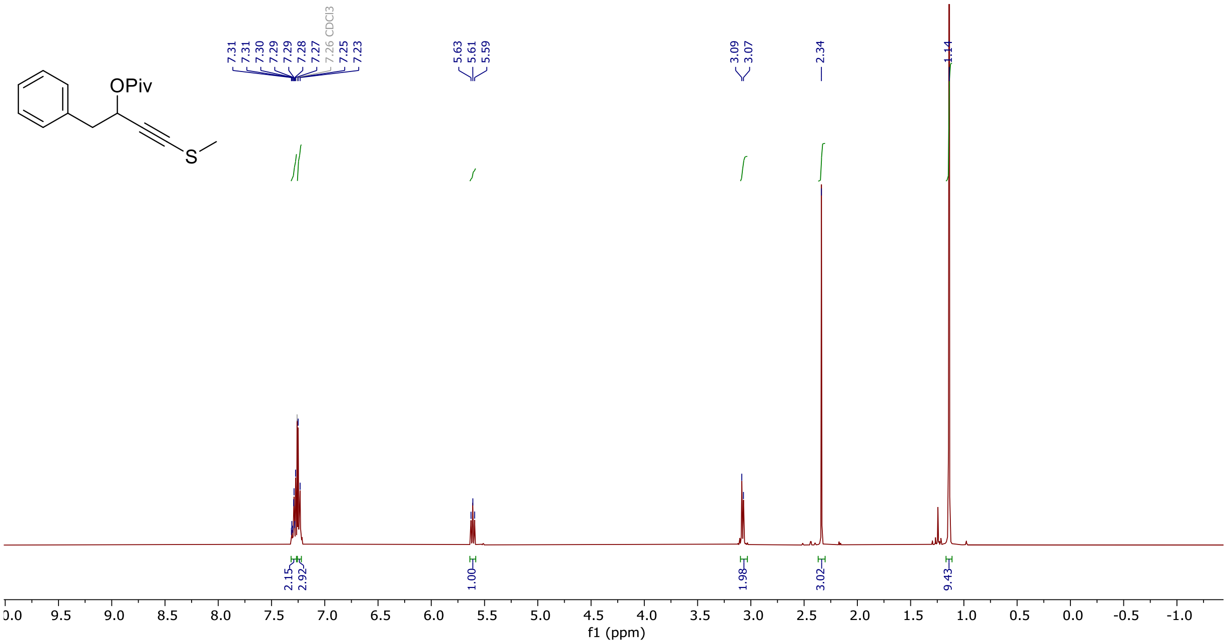

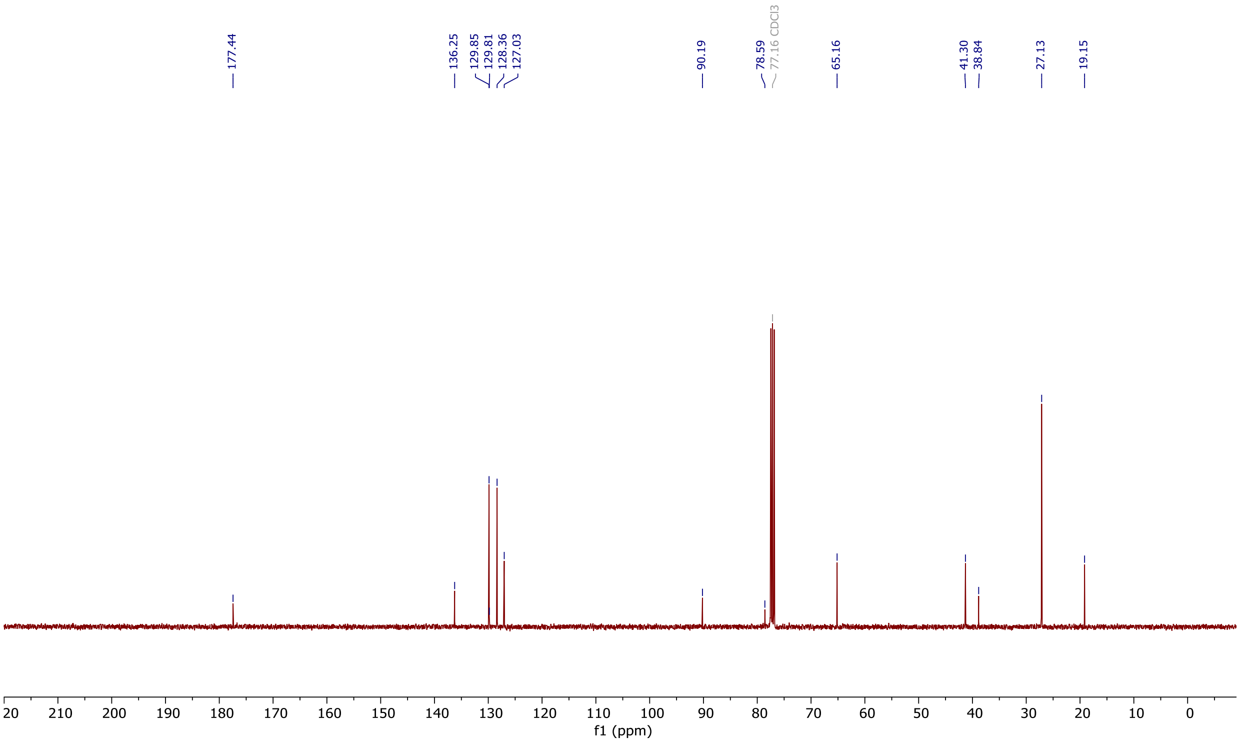

1d ^{1}H NMR (400 MHz, $CDCl_3$) & ^{13}C NMR (101 MHz, $CDCl_3$):

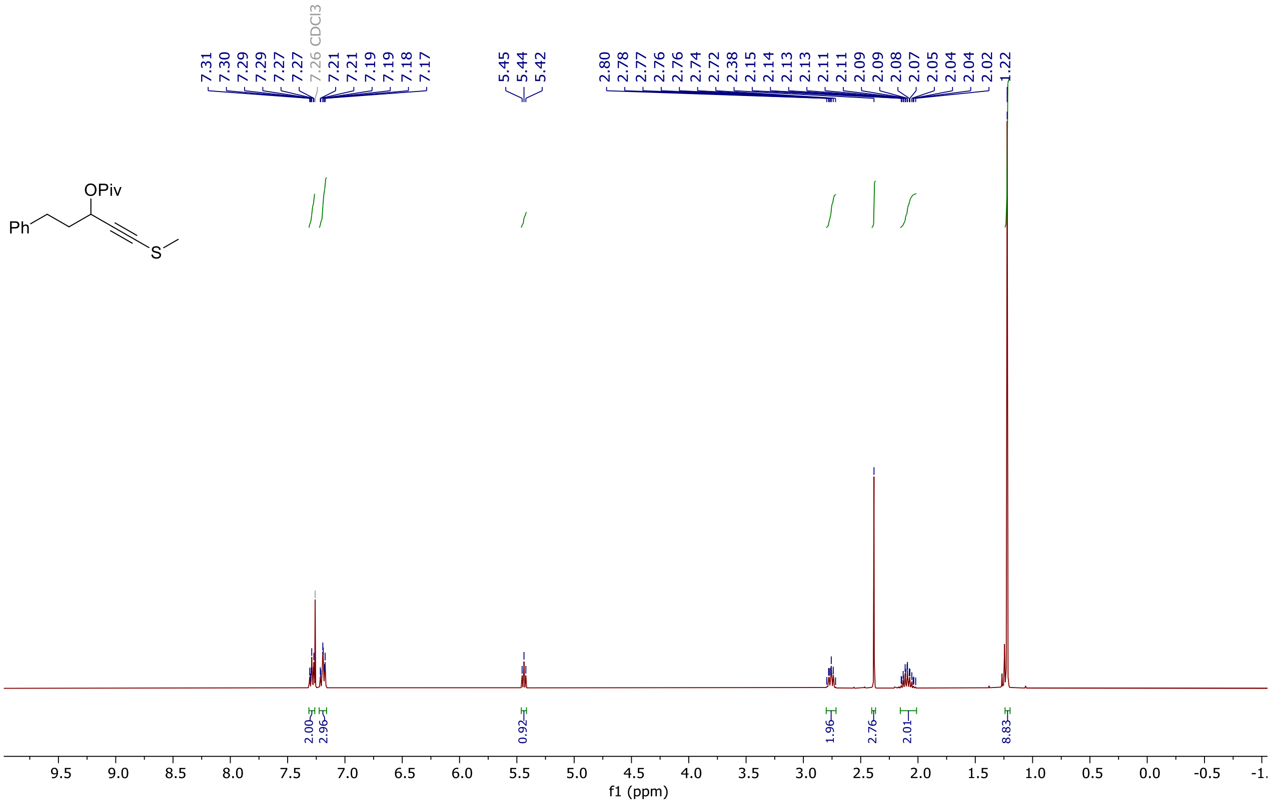

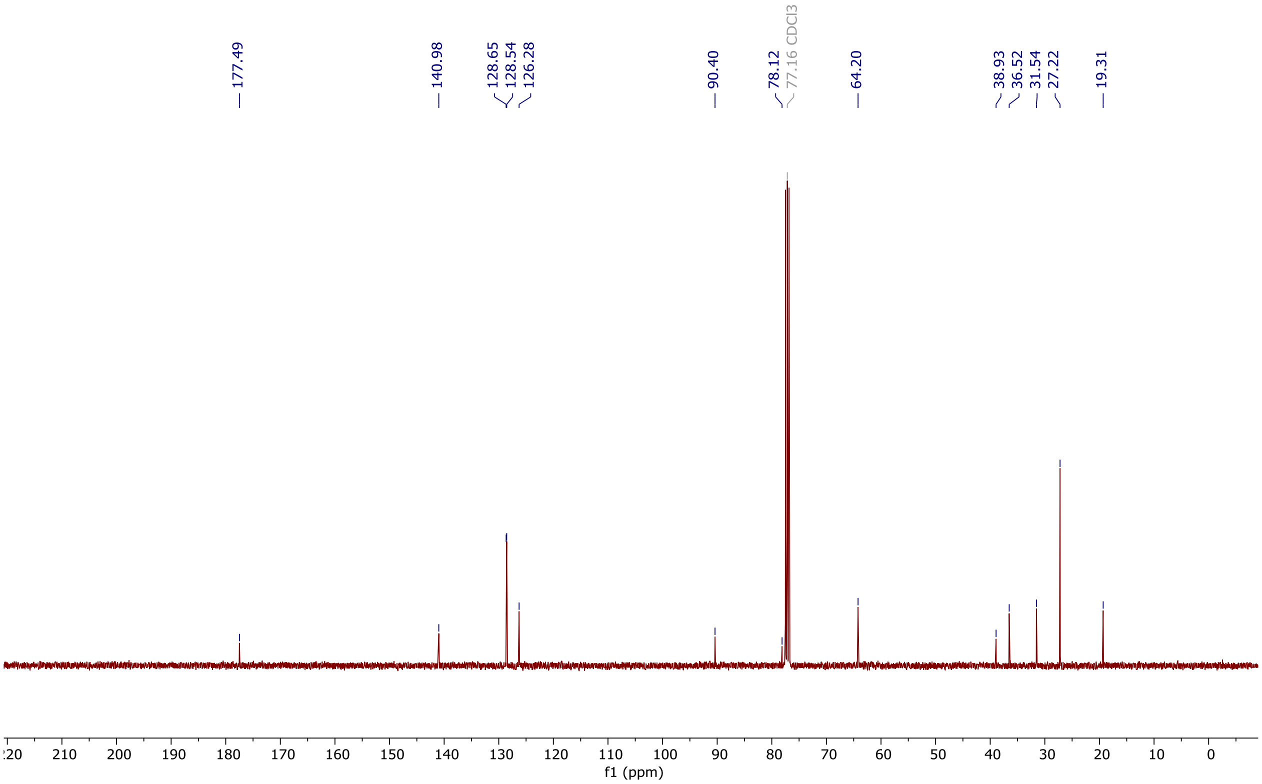

1e ^{1}H NMR (400 MHz, $CDCl_3$) & ^{13}C NMR (101 MHz, $CDCl_3$):

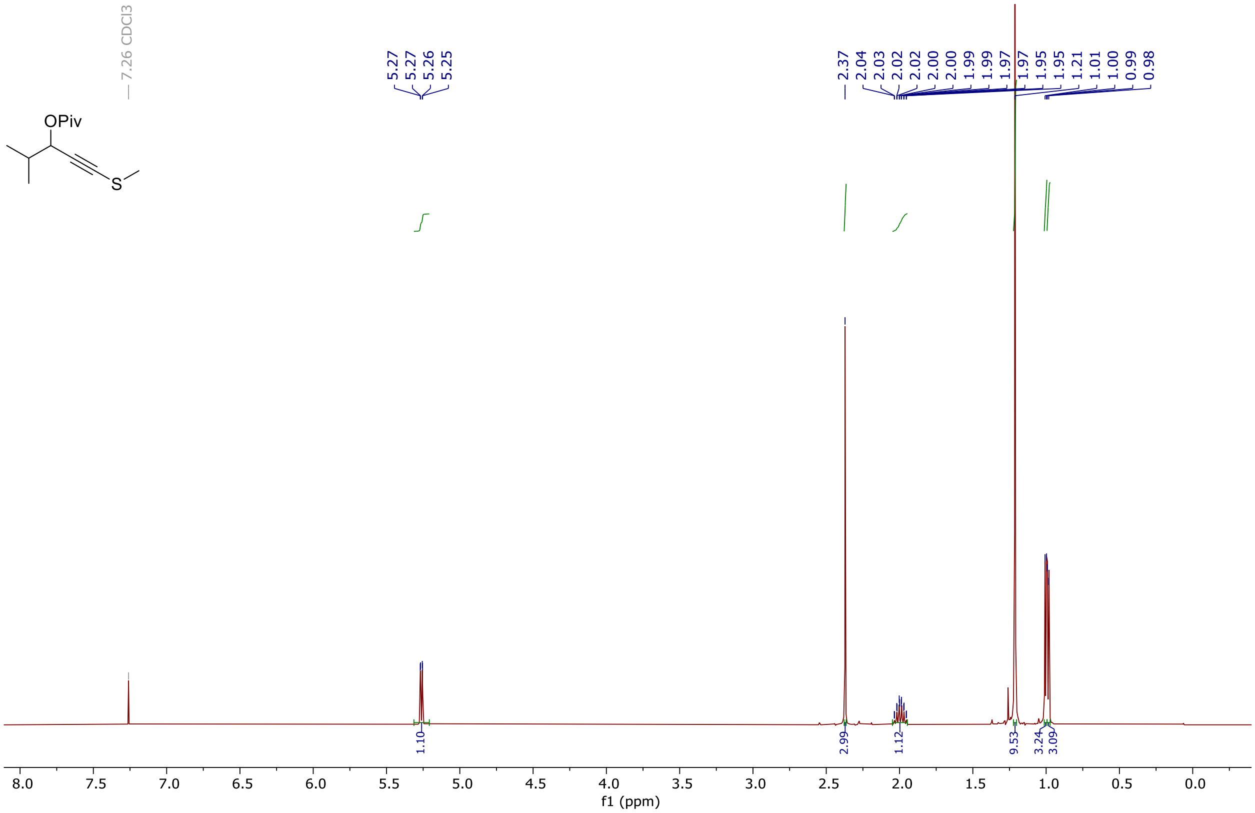

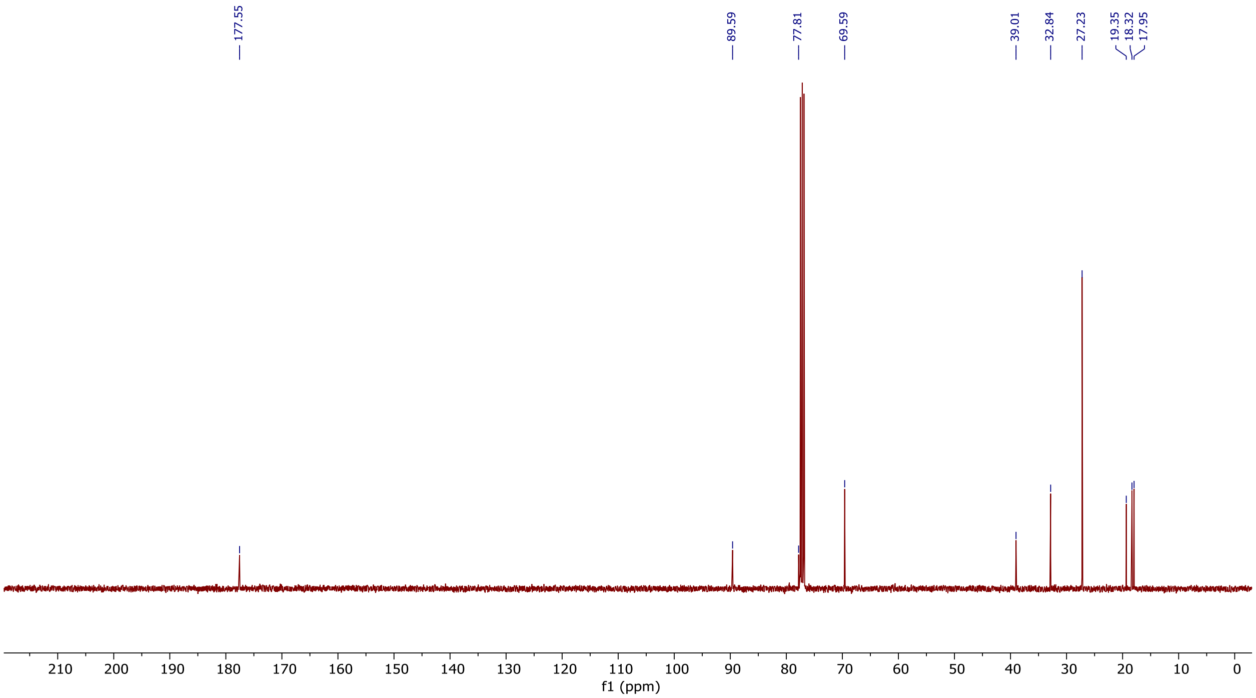

1f ^{1}H NMR (400 MHz, $CDCl_3$) & ^{13}C NMR (101 MHz, $CDCl_3$):

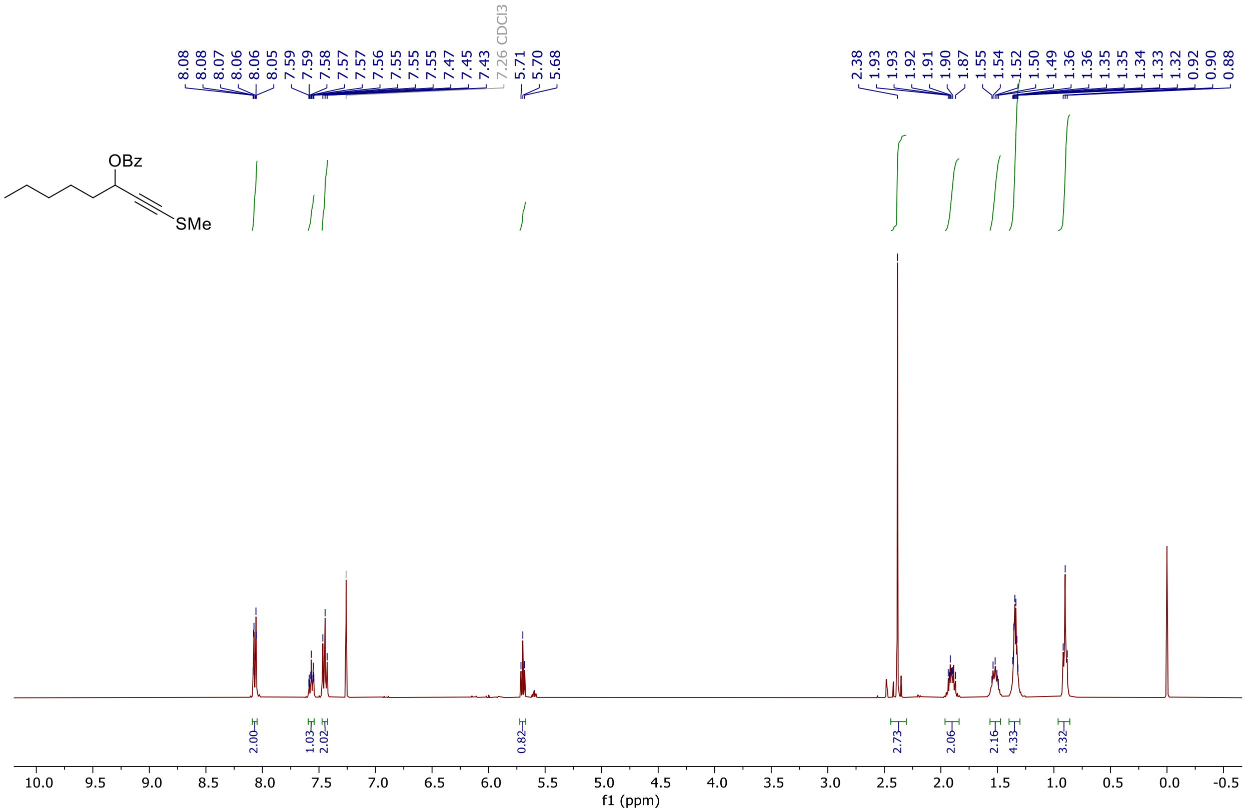

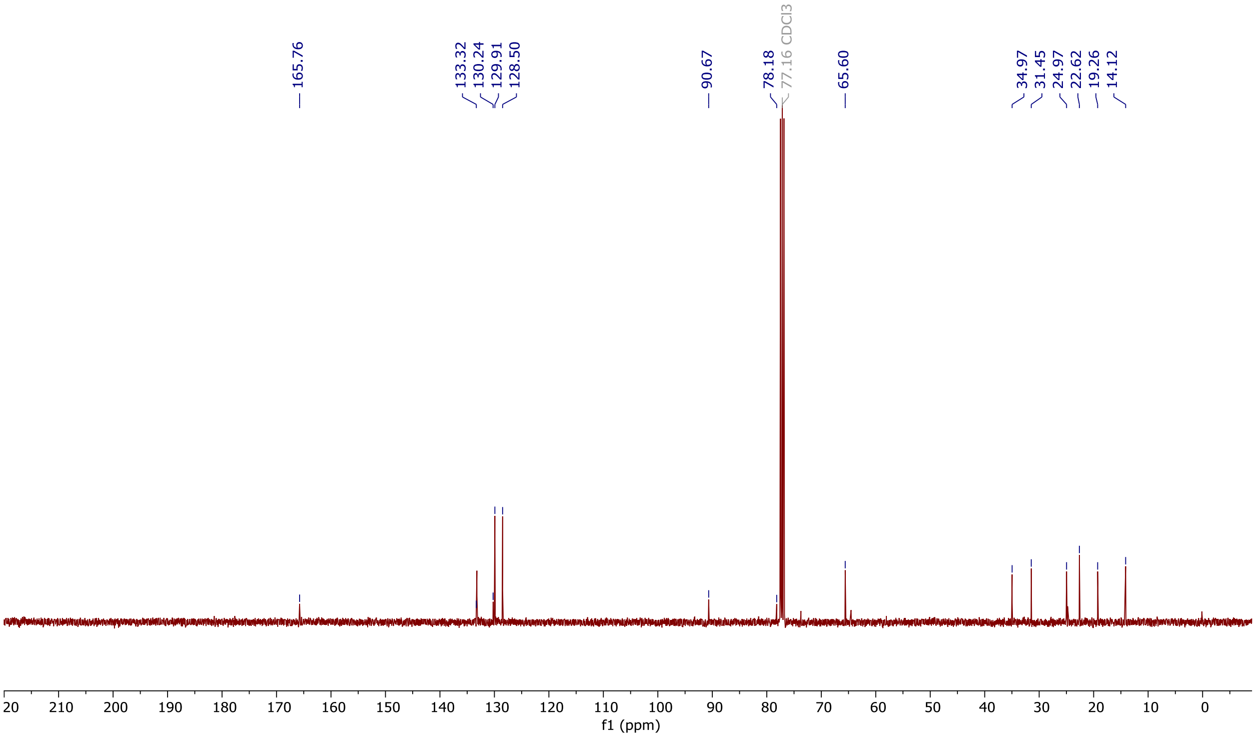

1g ^{1}H NMR (400 MHz, $CDCl_3$) & ^{13}C NMR (101 MHz, $CDCl_3$):

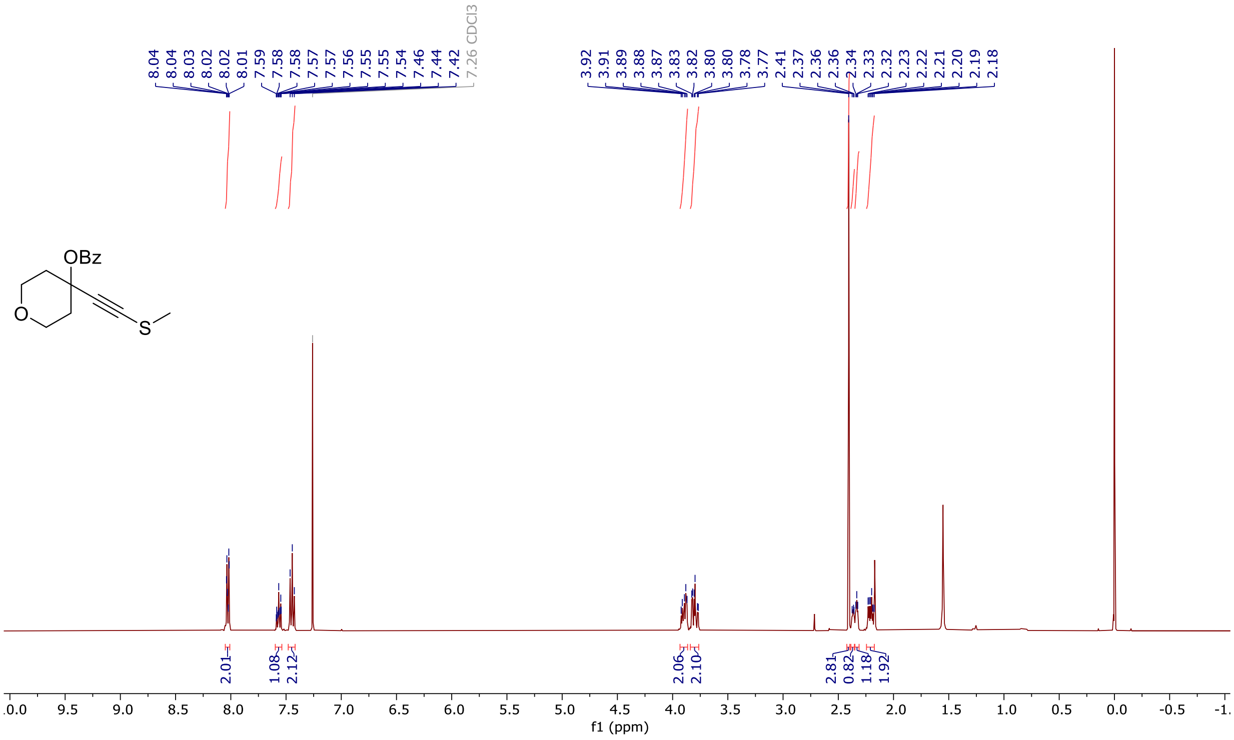

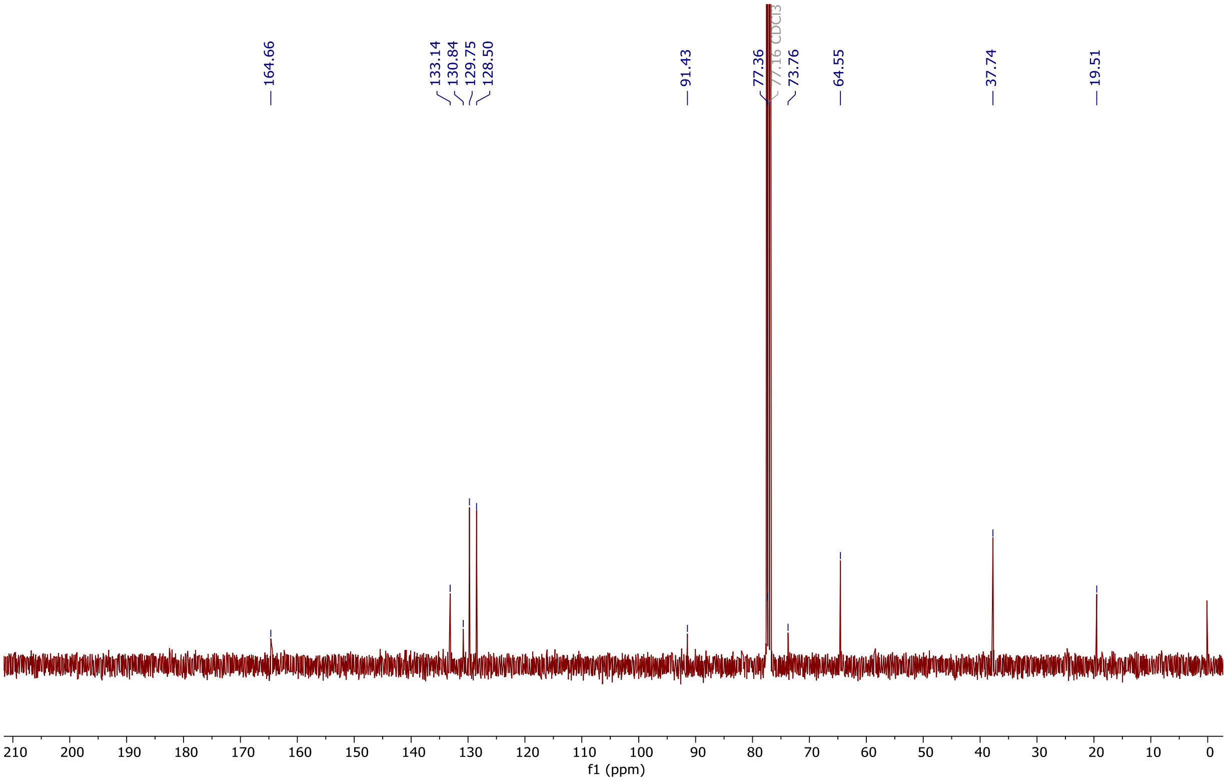

1h ^{1}H NMR (400 MHz, $CDCl_3$) & ^{13}C NMR (101 MHz, $CDCl_3$):

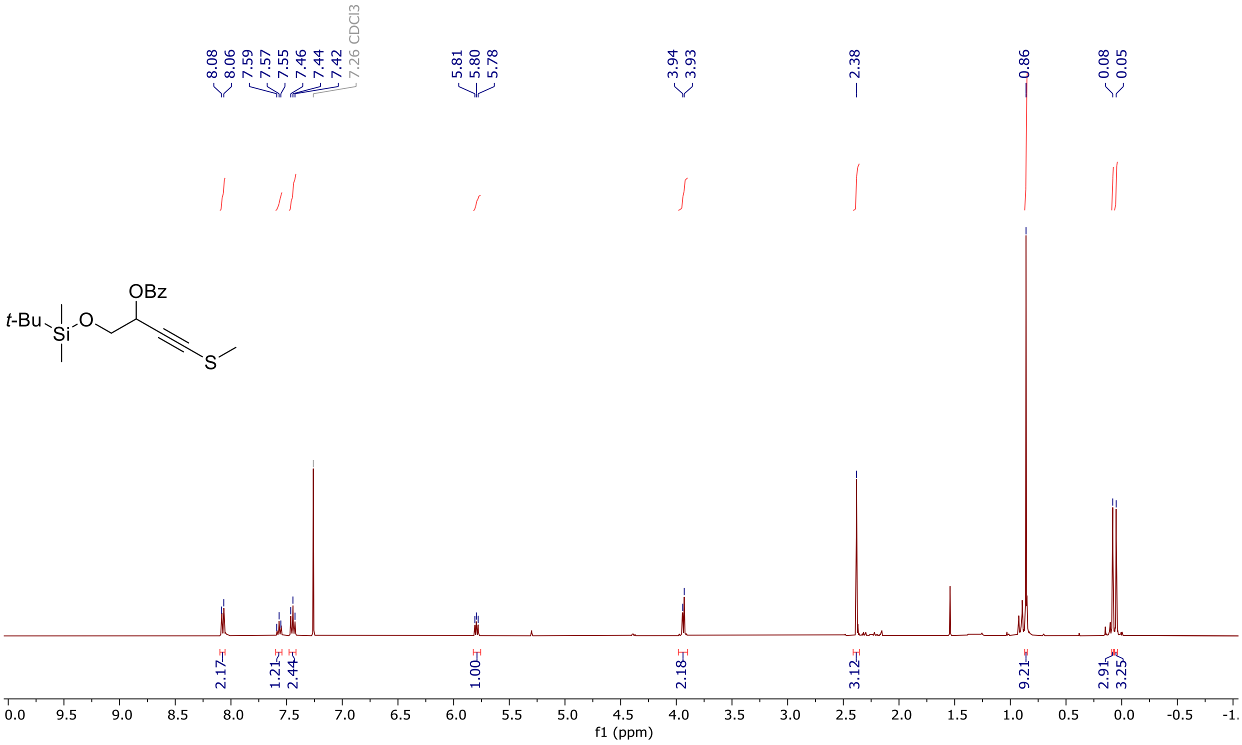

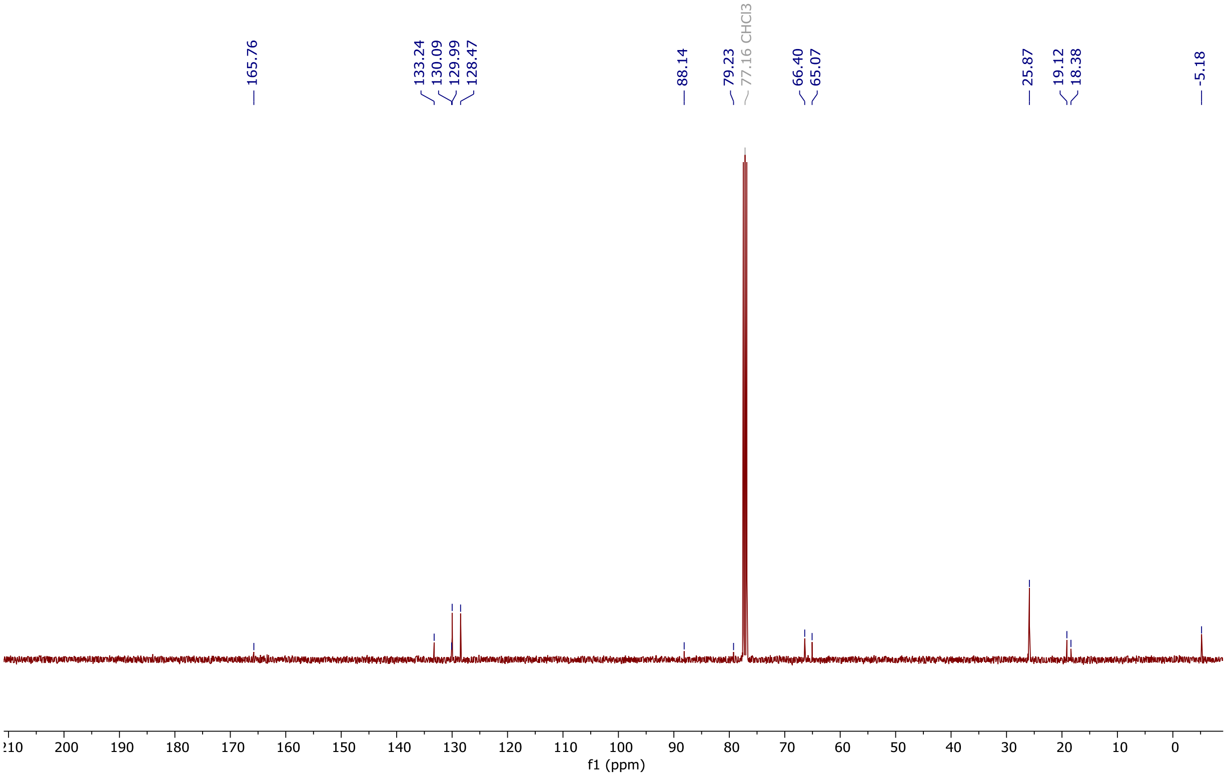

1i ^1H NMR (400 MHz, $CDCl_3$) & ^{13}C NMR (101 MHz, $CDCl_3$):

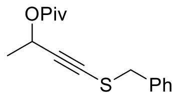

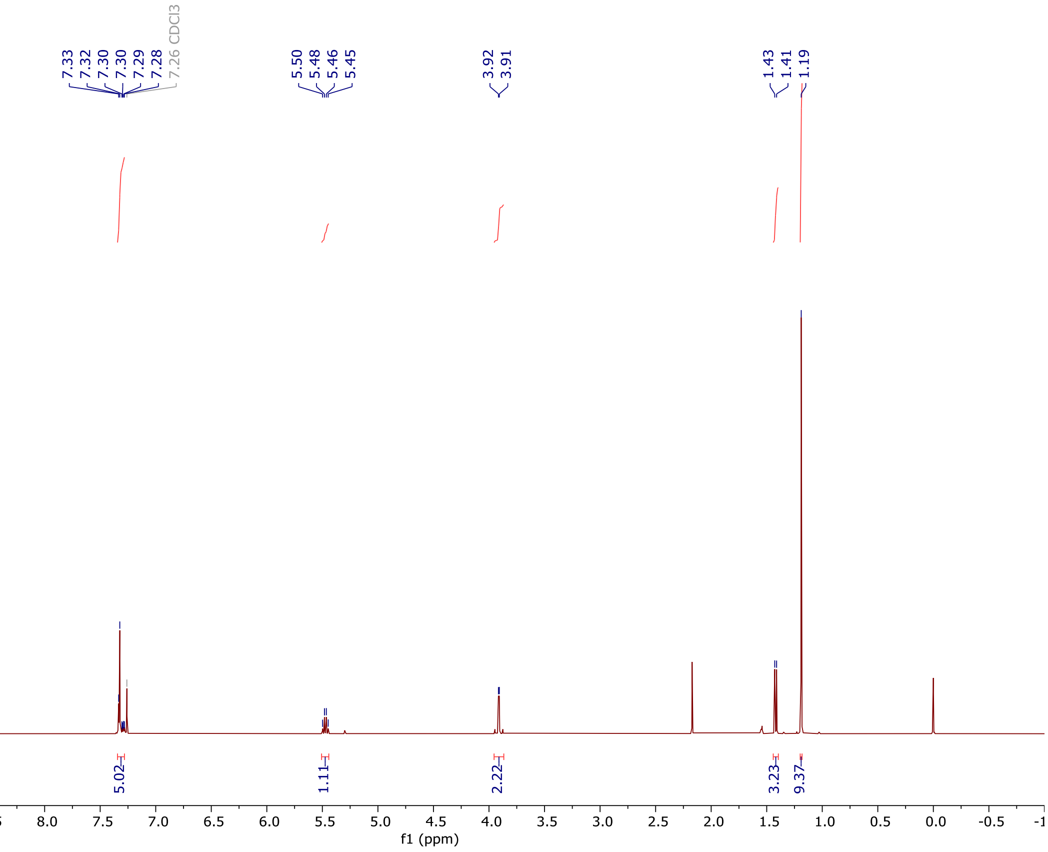

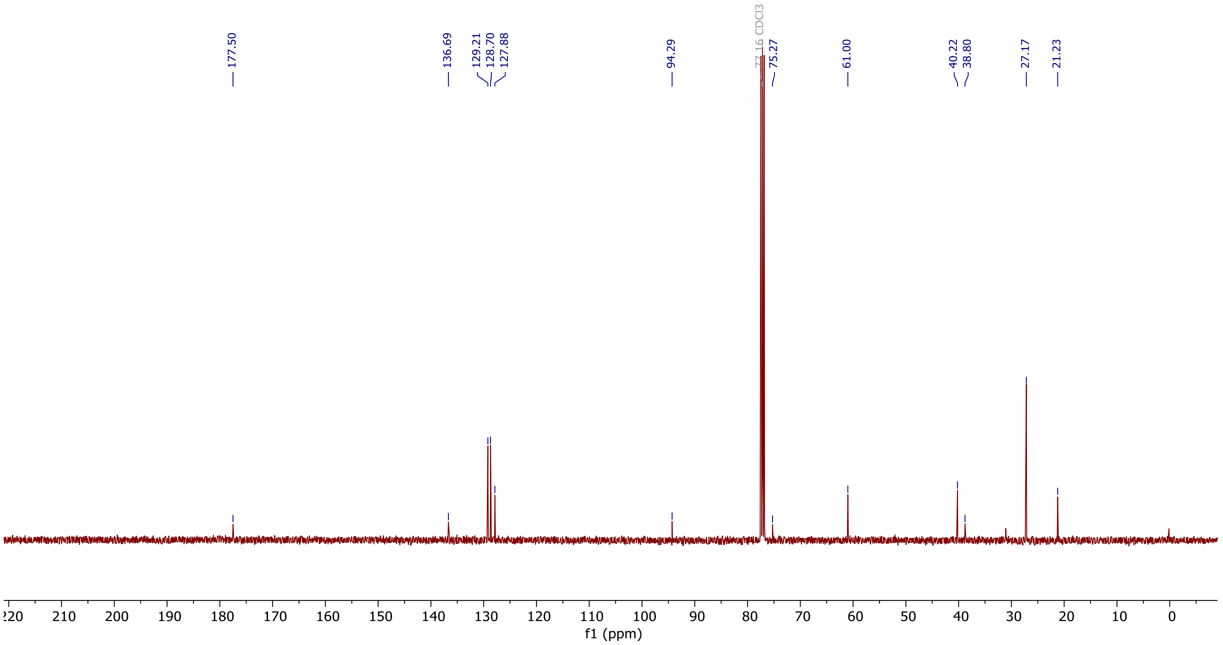

1j ^{1}H NMR (400 MHz, $CDCl_3$) & ^{13}C NMR (101 MHz, $CDCl_3$):

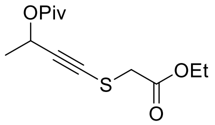

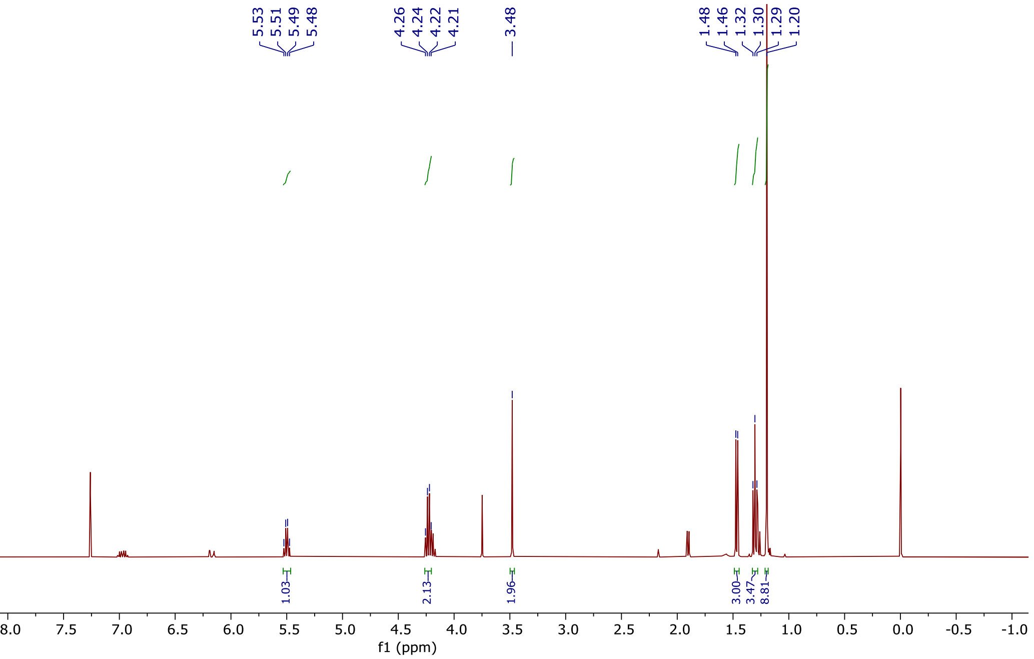

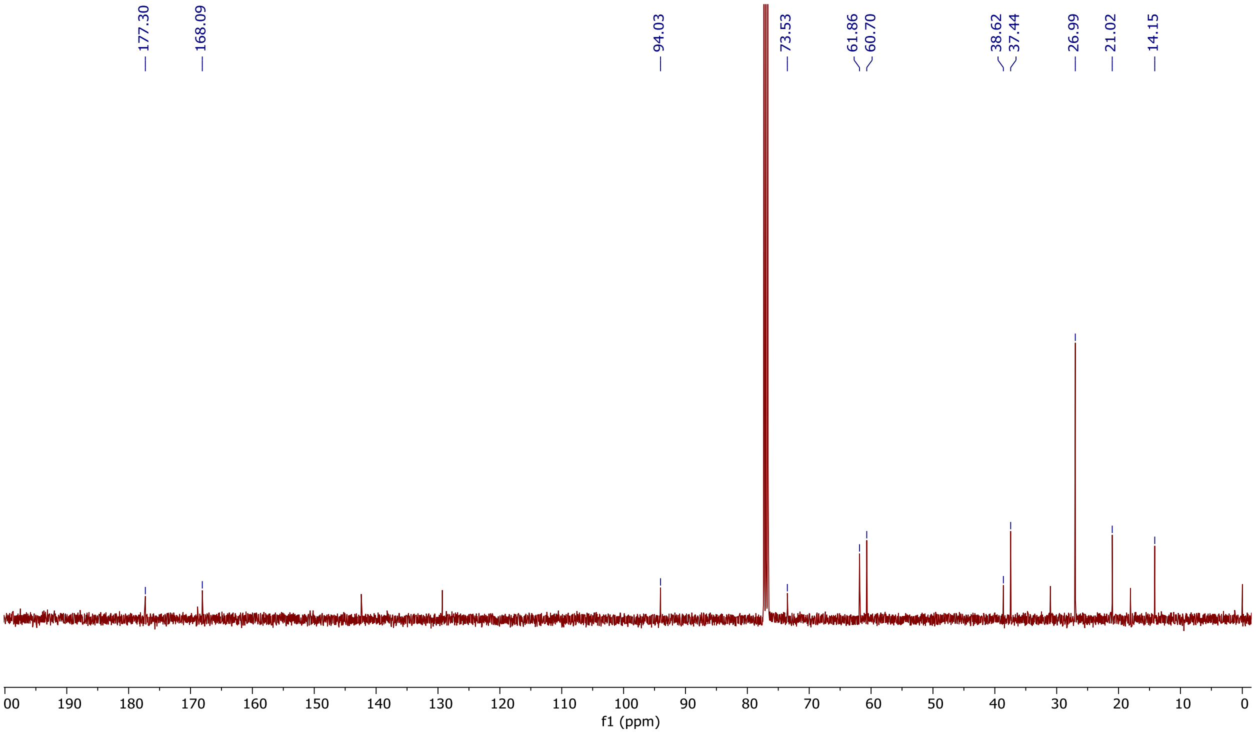

Ethyl 2-((3-hydroxybut-1-yn-1-yl)thio)acetate ^{1}H NMR (400 MHz, $CDCl_3$) & ^{13}C NMR (101 MHz, $CDCl_3$):

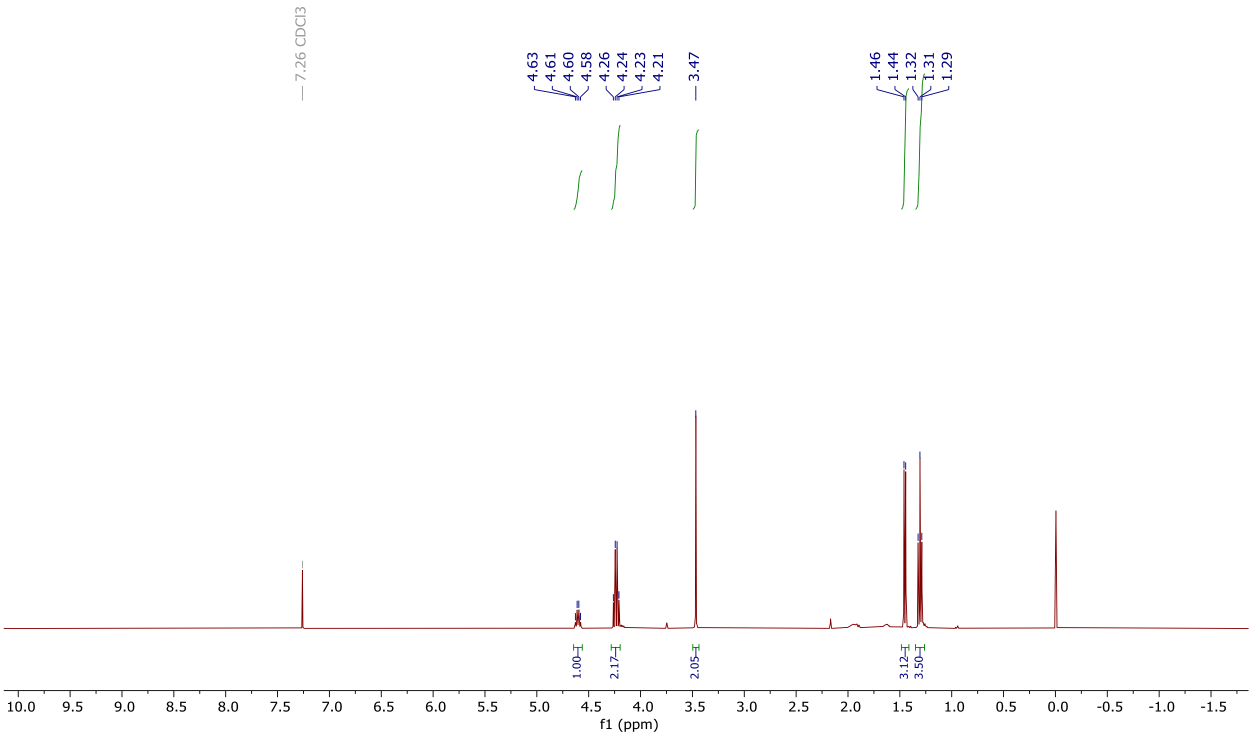

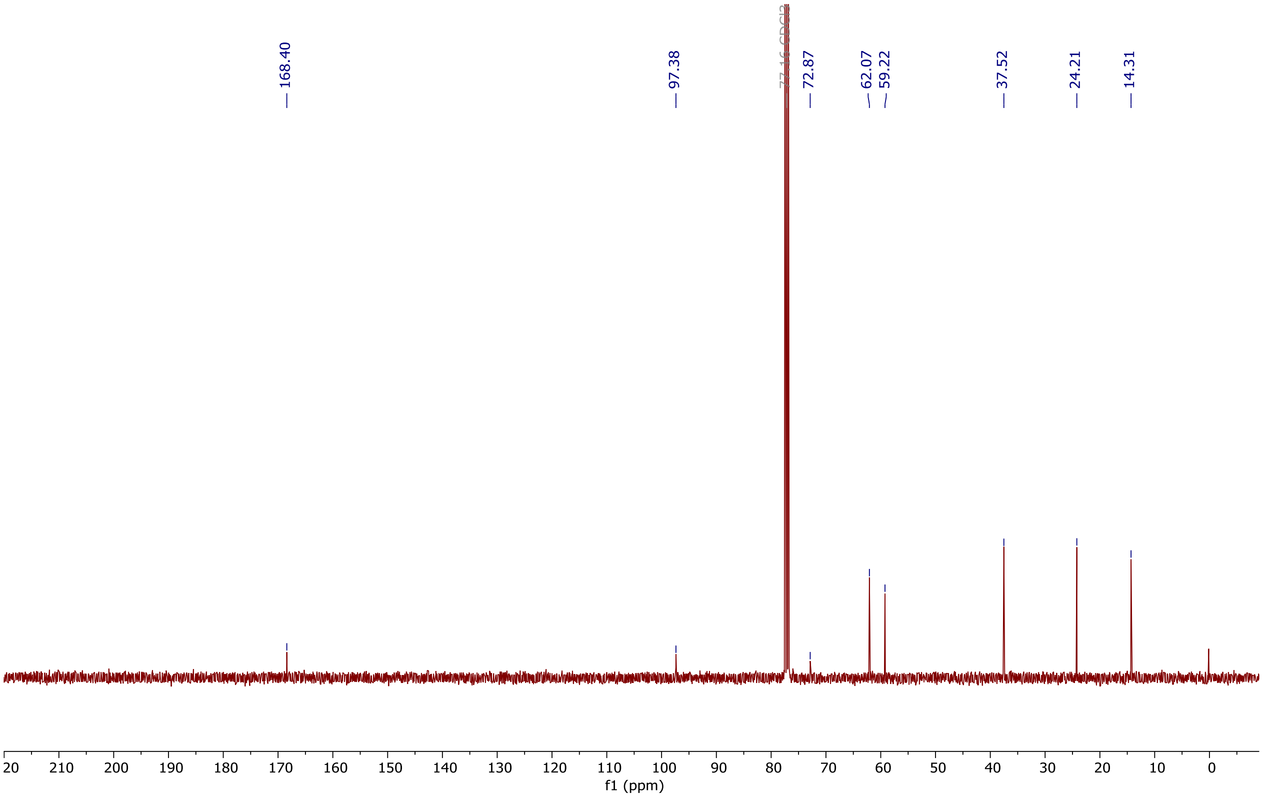

1k ^{1}H NMR (400 MHz, $CDCl_3$) & ^{13}C NMR (101 MHz, $CDCl_3$):

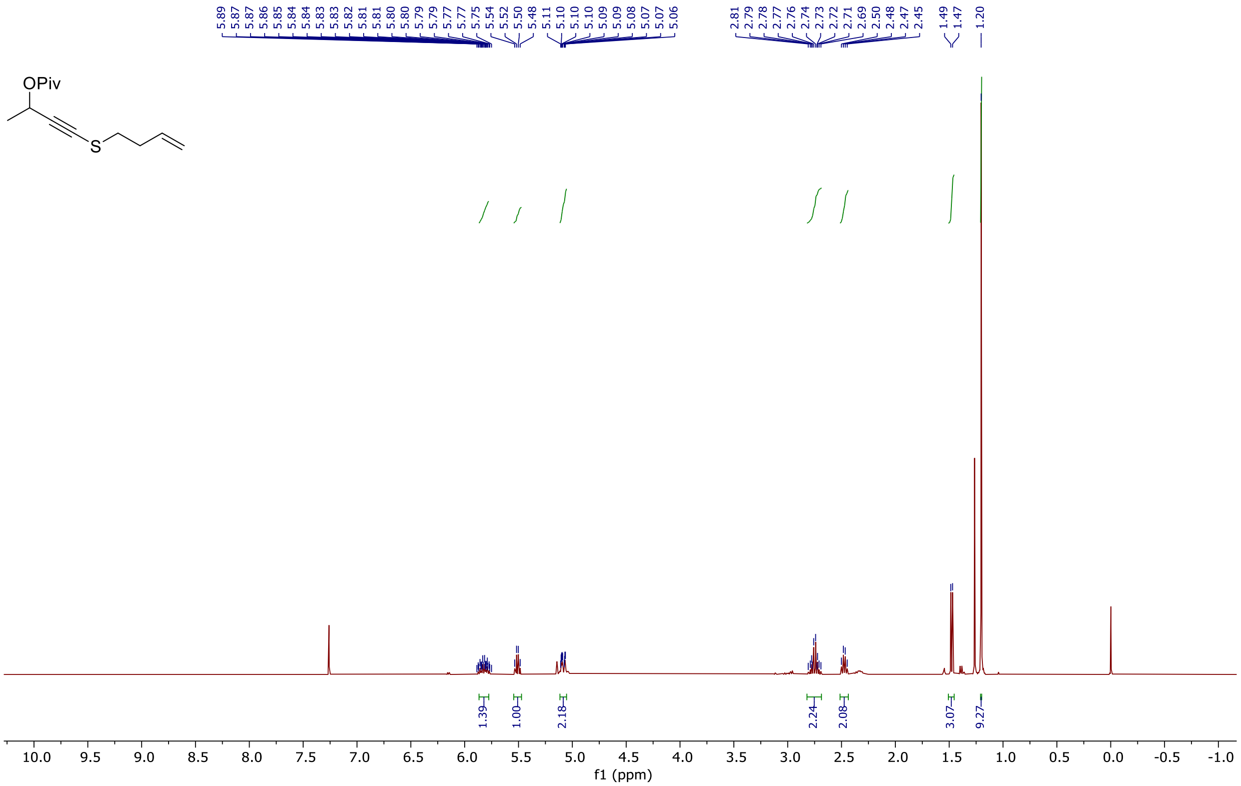

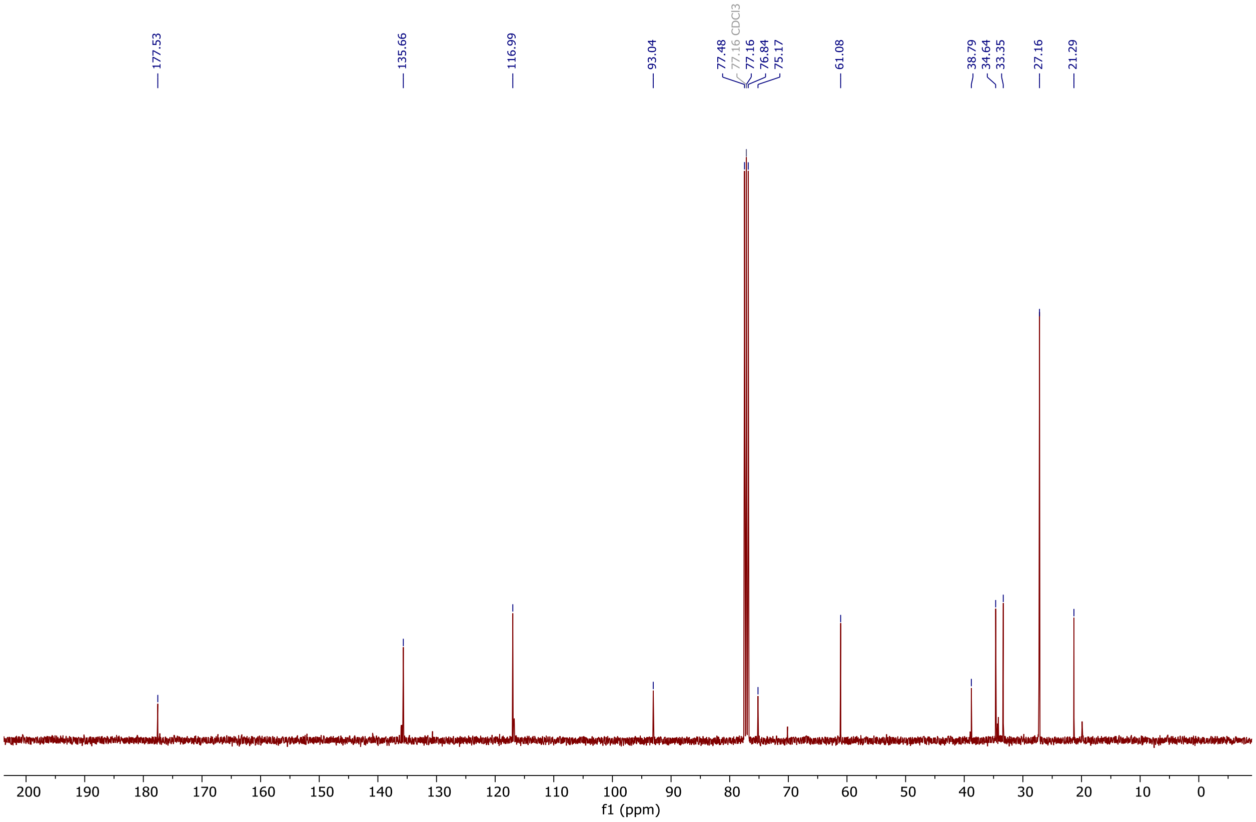

1l ^{1}H NMR (400 MHz, $CDCl_3$) & ^{13}C NMR (101 MHz, $CDCl_3$):

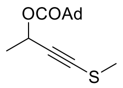

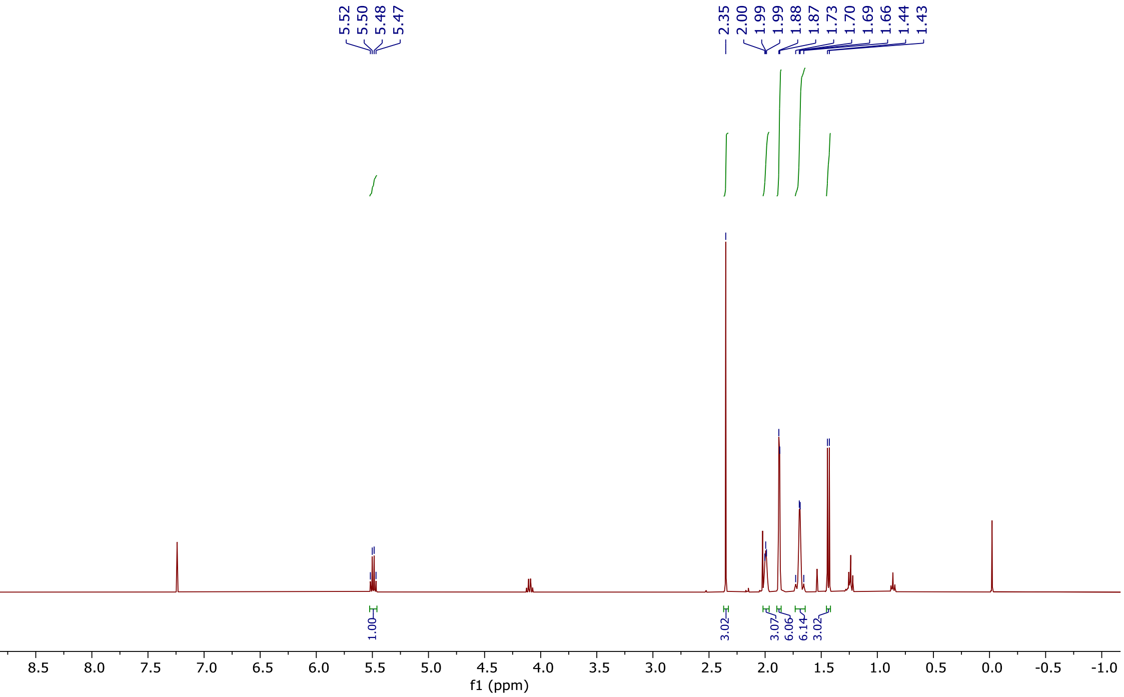

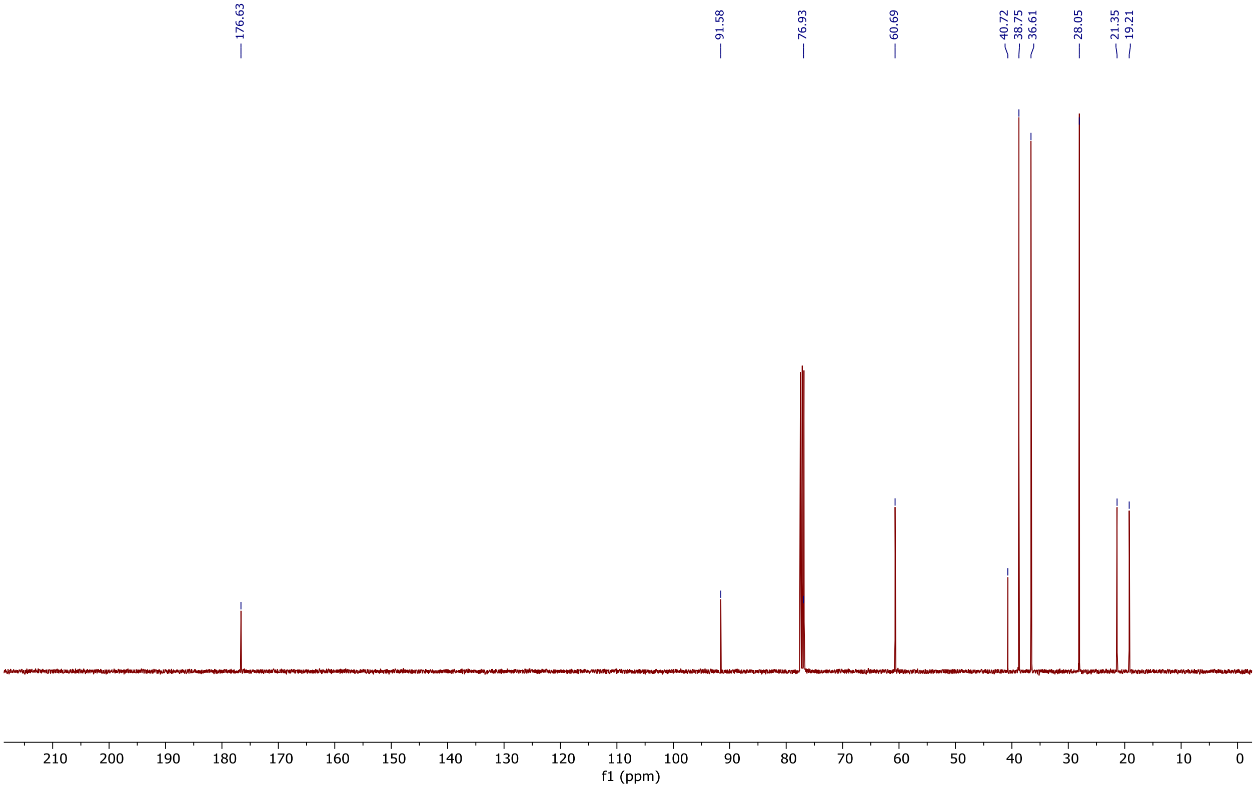

1m ^{1}H NMR (400 MHz, $CDCl_3$) & ^{13}C NMR (101 MHz, $CDCl_3$):

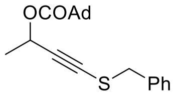

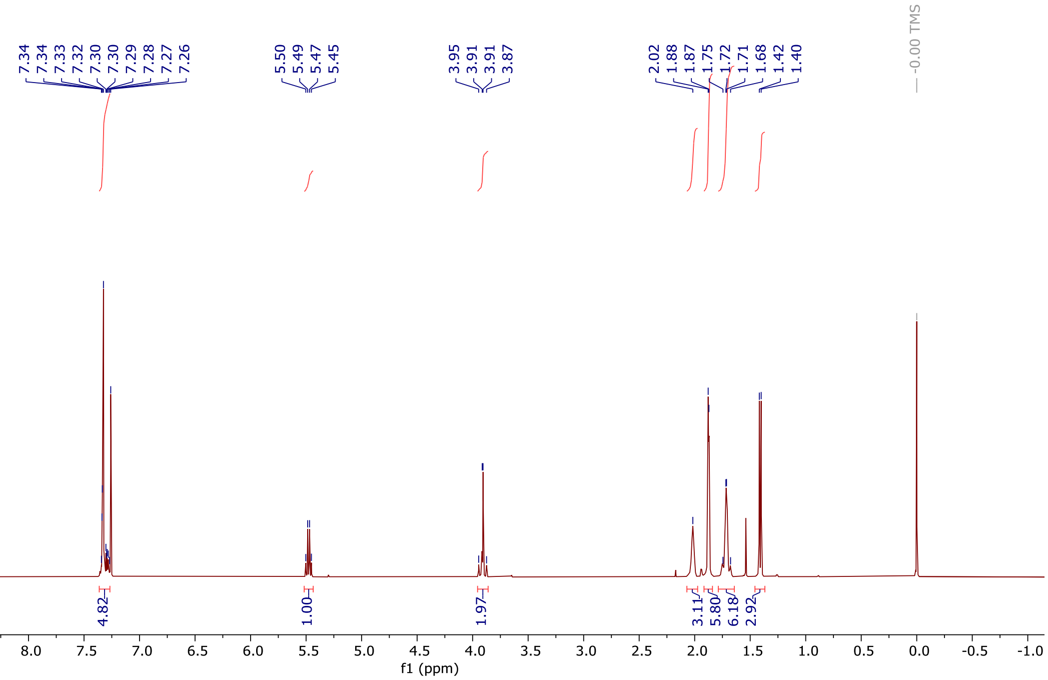

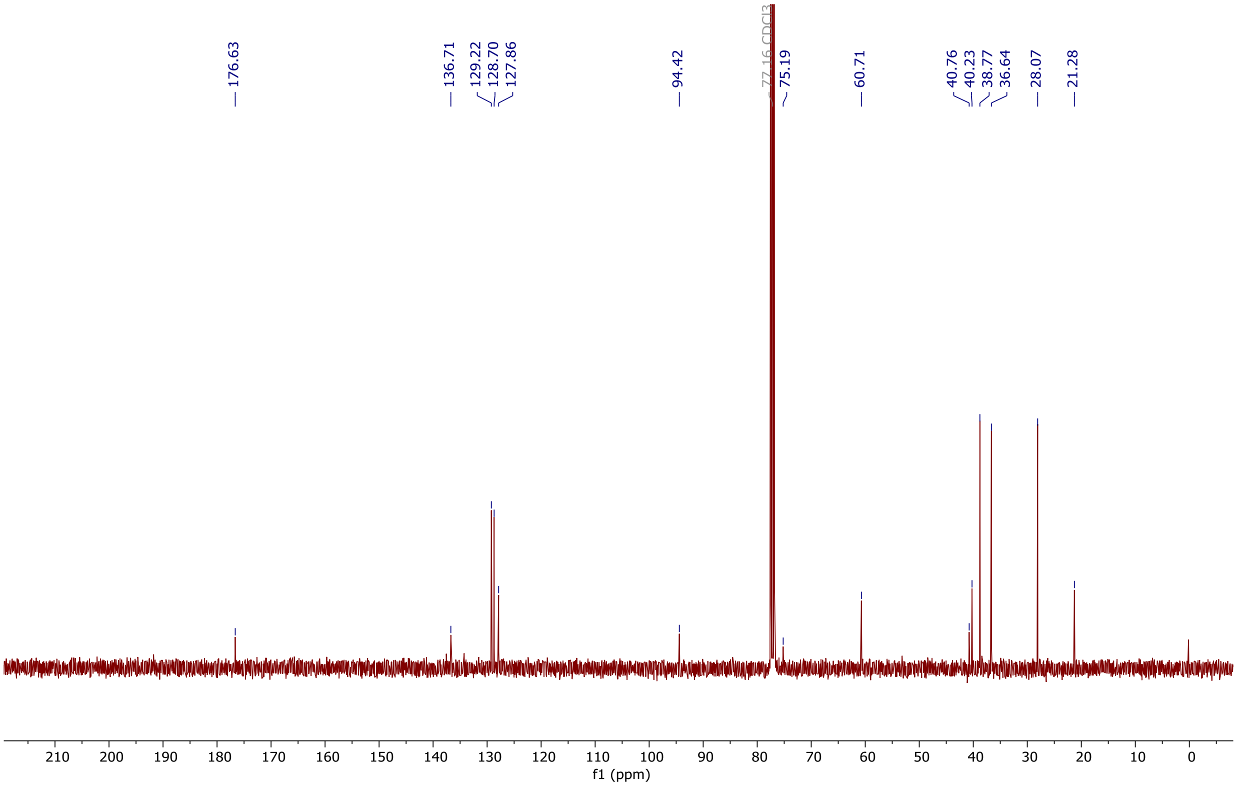

1n ^{1}H NMR (400 MHz, $CDCl_3$) & ^{13}C NMR (101 MHz, $CDCl_3$):

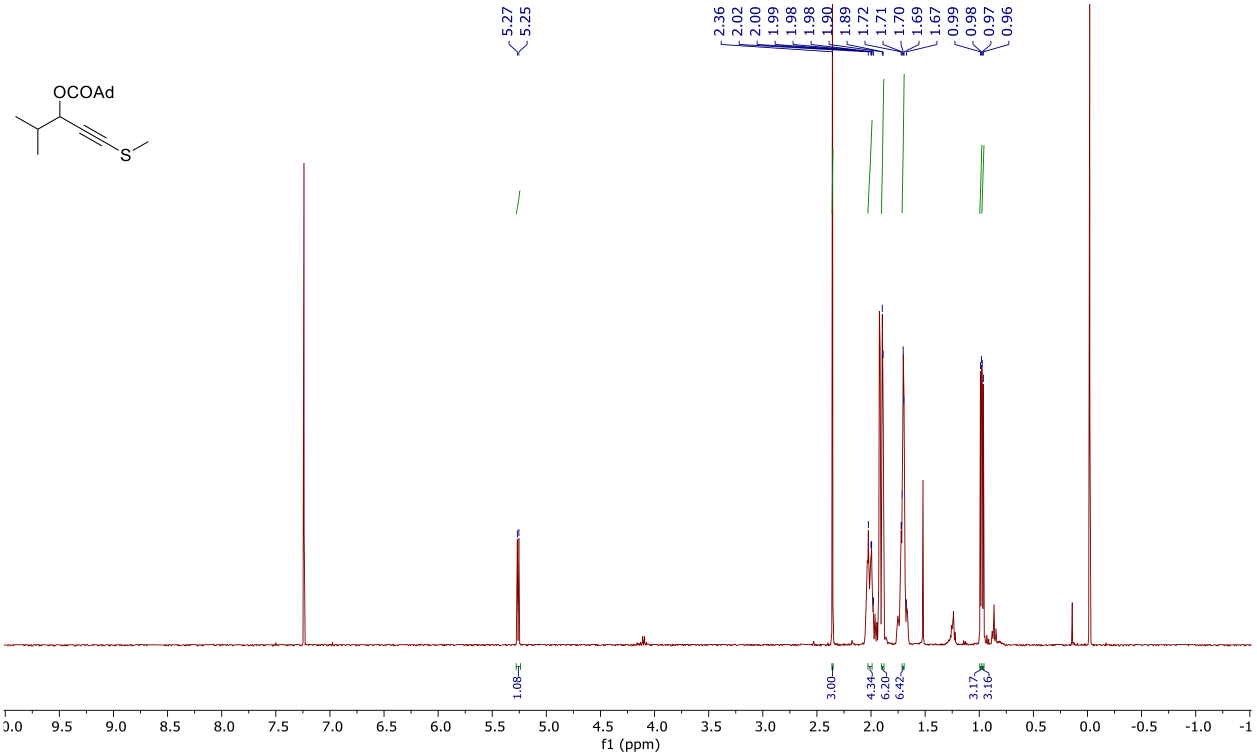

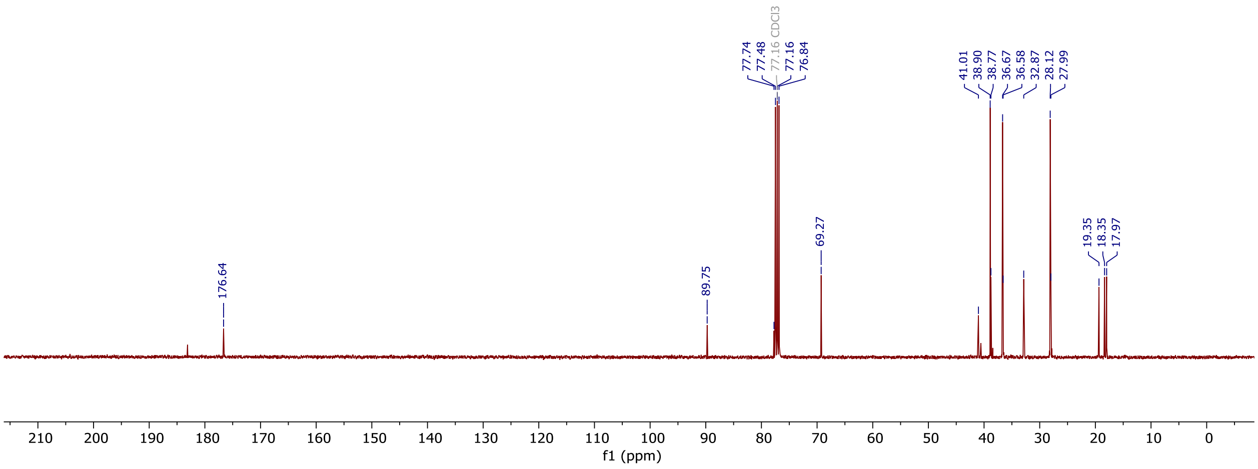

1o ^{1}H NMR (400 MHz, $CDCl_3$) & ^{13}C NMR (101 MHz, $CDCl_3$):

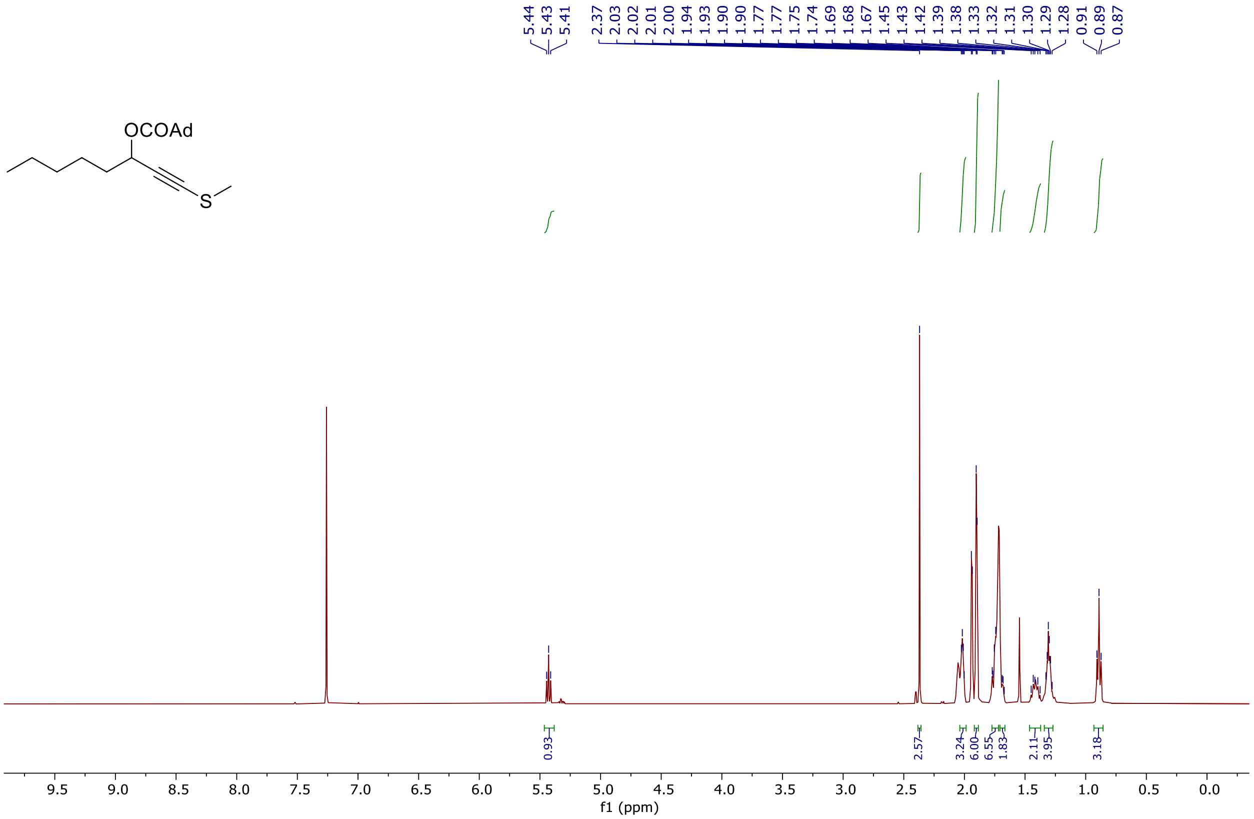

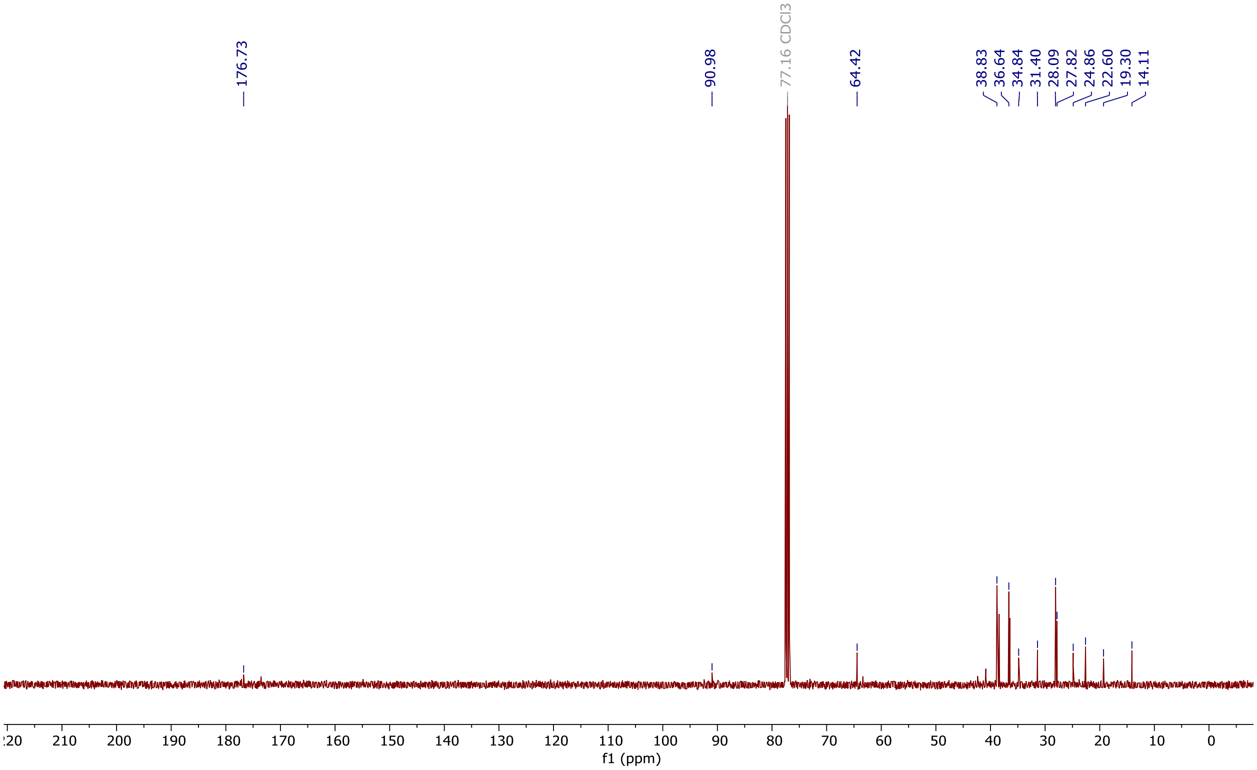

3aa ^{1}H NMR (300 MHz, $CDCl_3$) & ^{13}C NMR (101 MHz, $CDCl_3$):

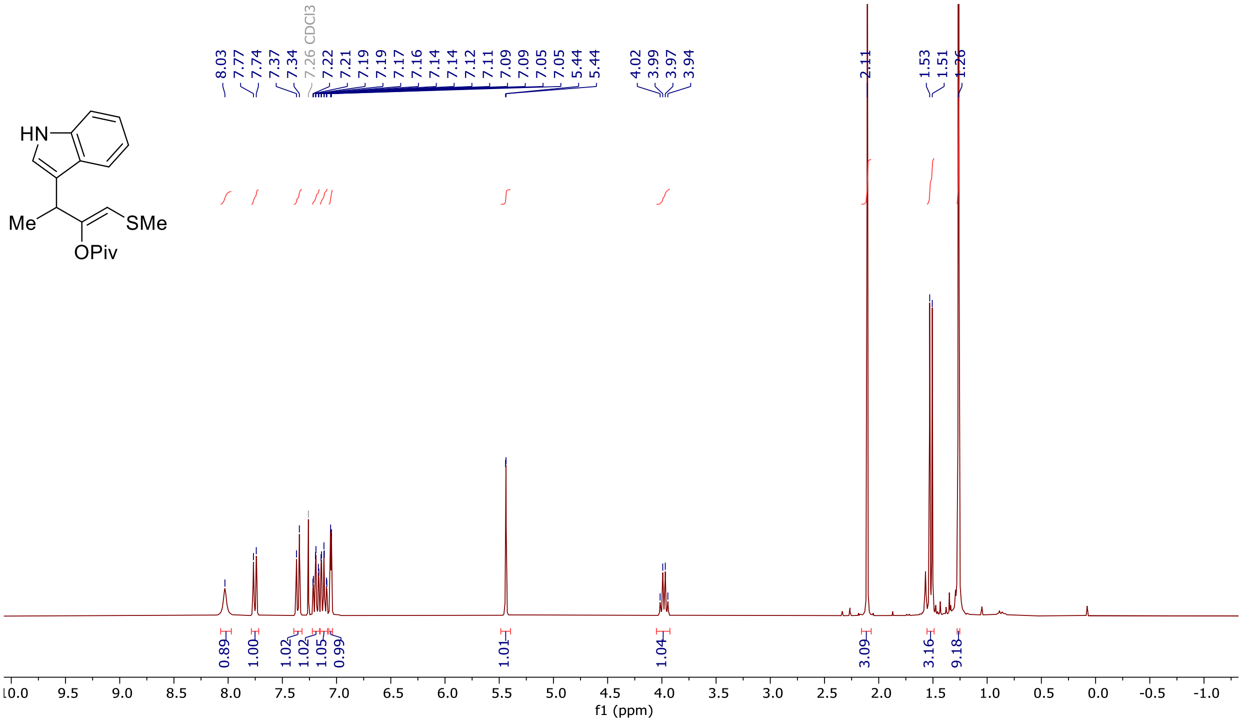

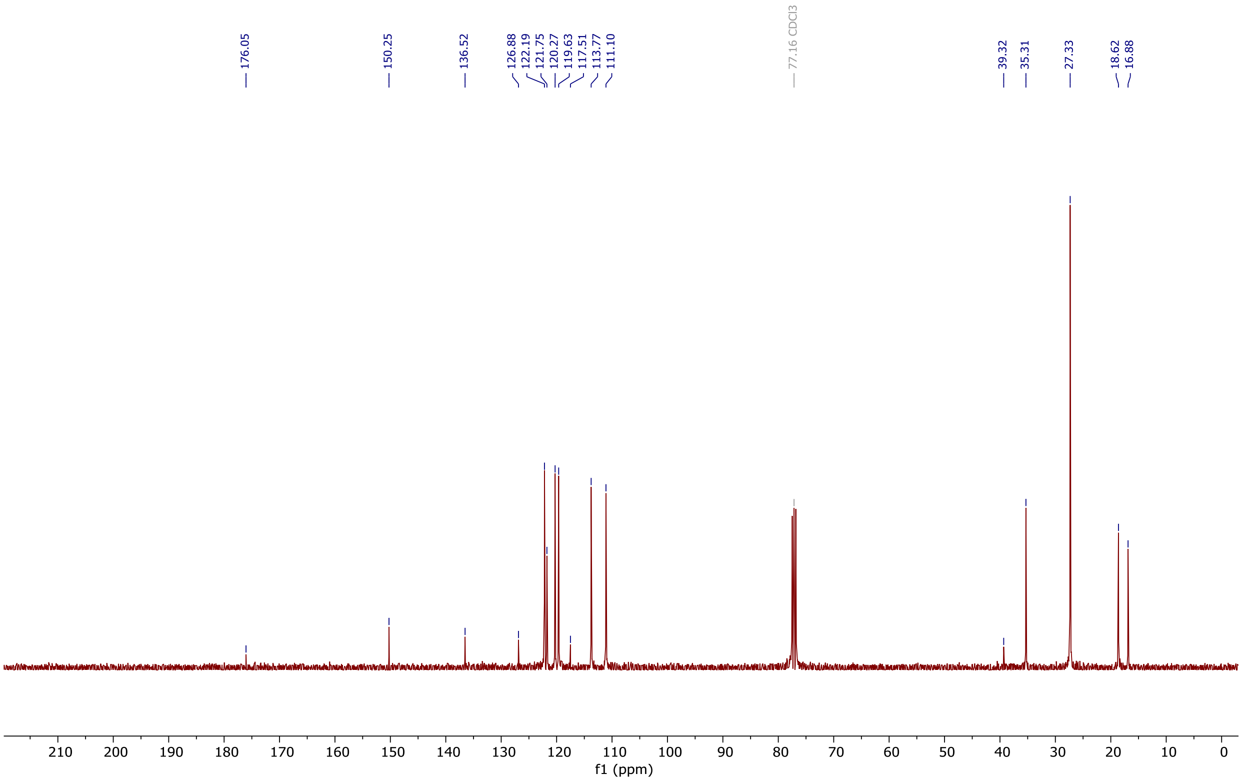

3ba ^{1}H NMR (400 MHz, $CDCl_3$) & ^{13}C NMR (101 MHz, $CDCl_3$):

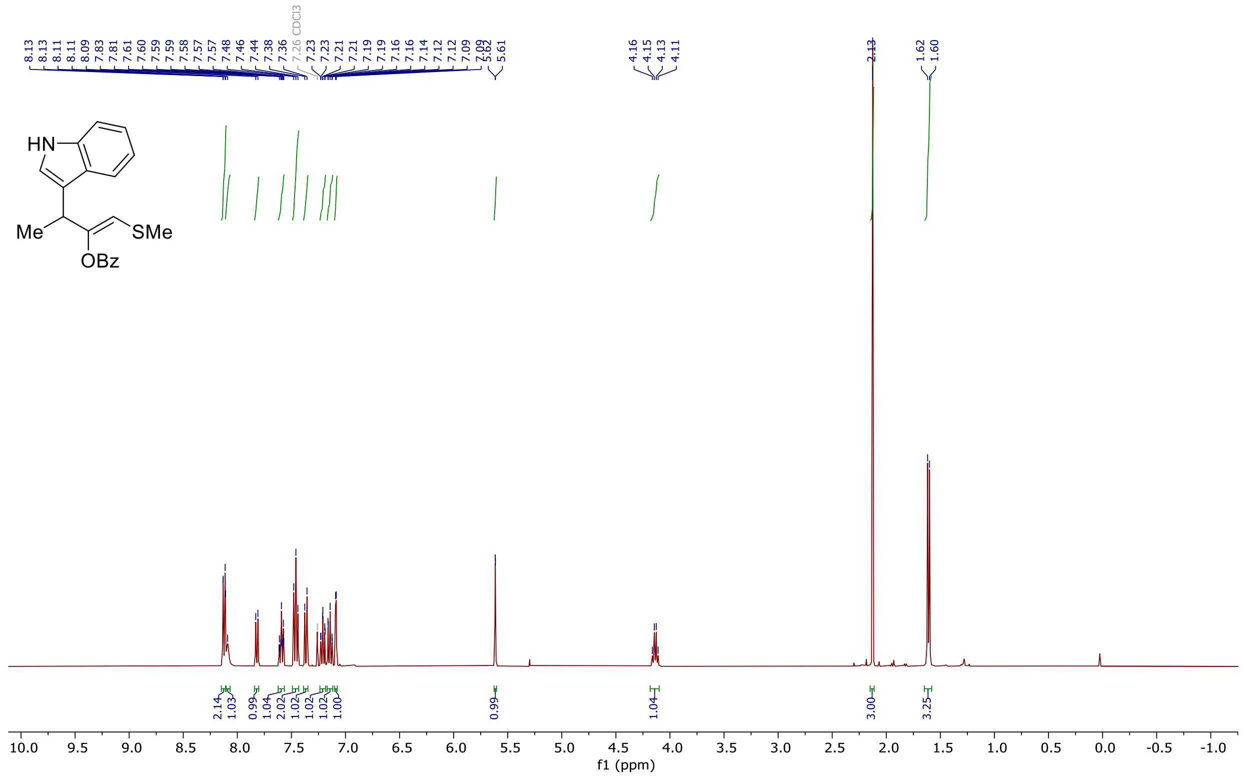

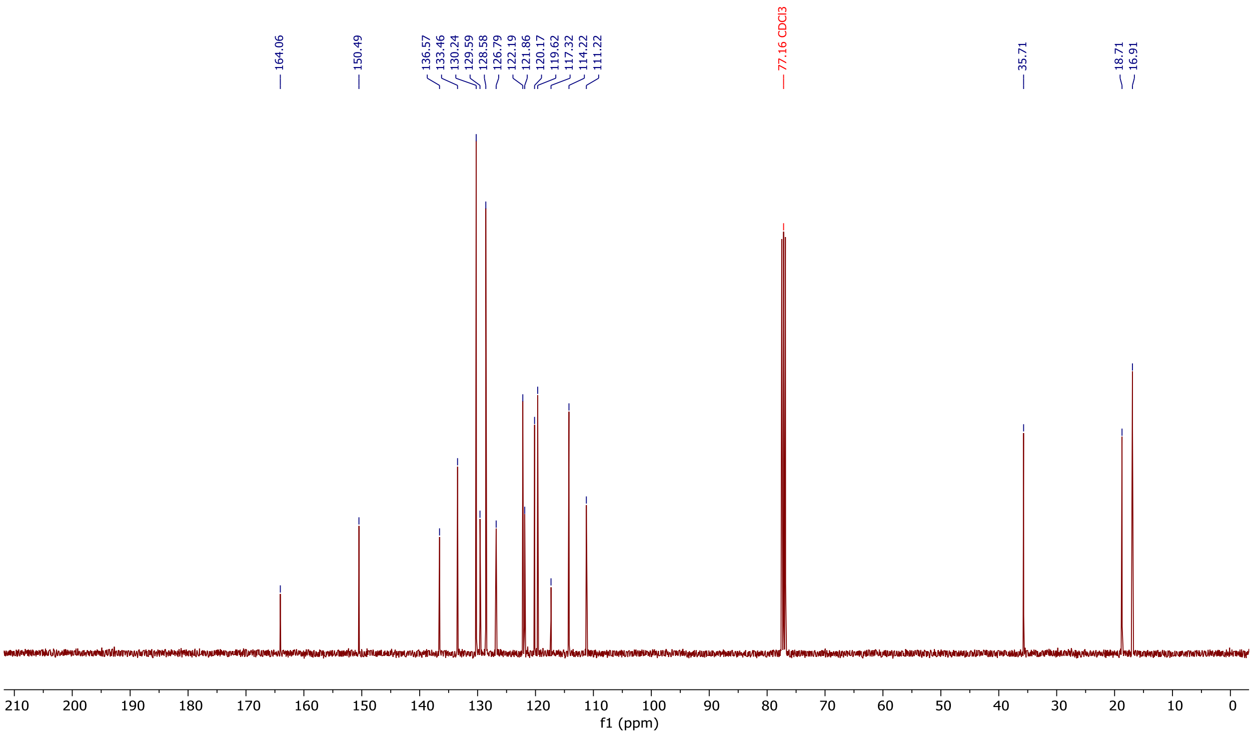

3ab ^{1}H NMR (400 MHz, $CDCl_3$) & ^{13}C NMR (101 MHz, $CDCl_3$):

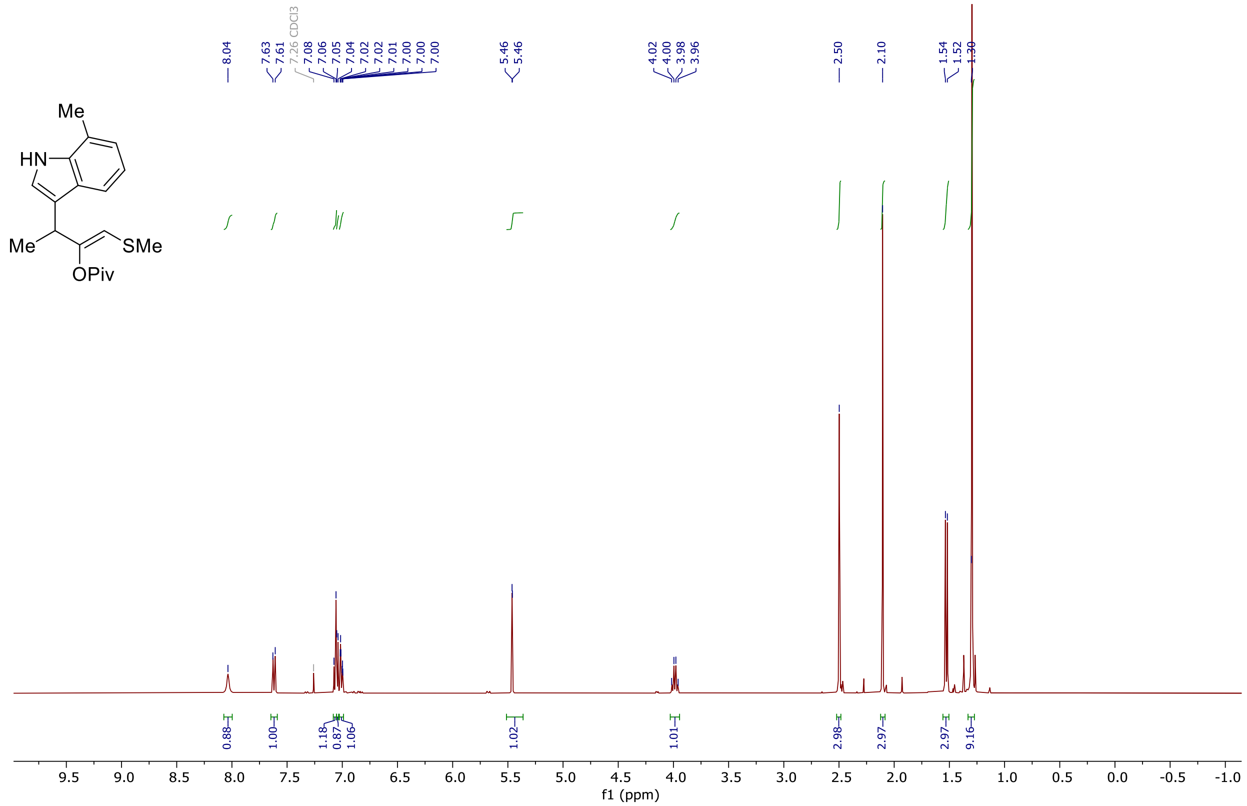

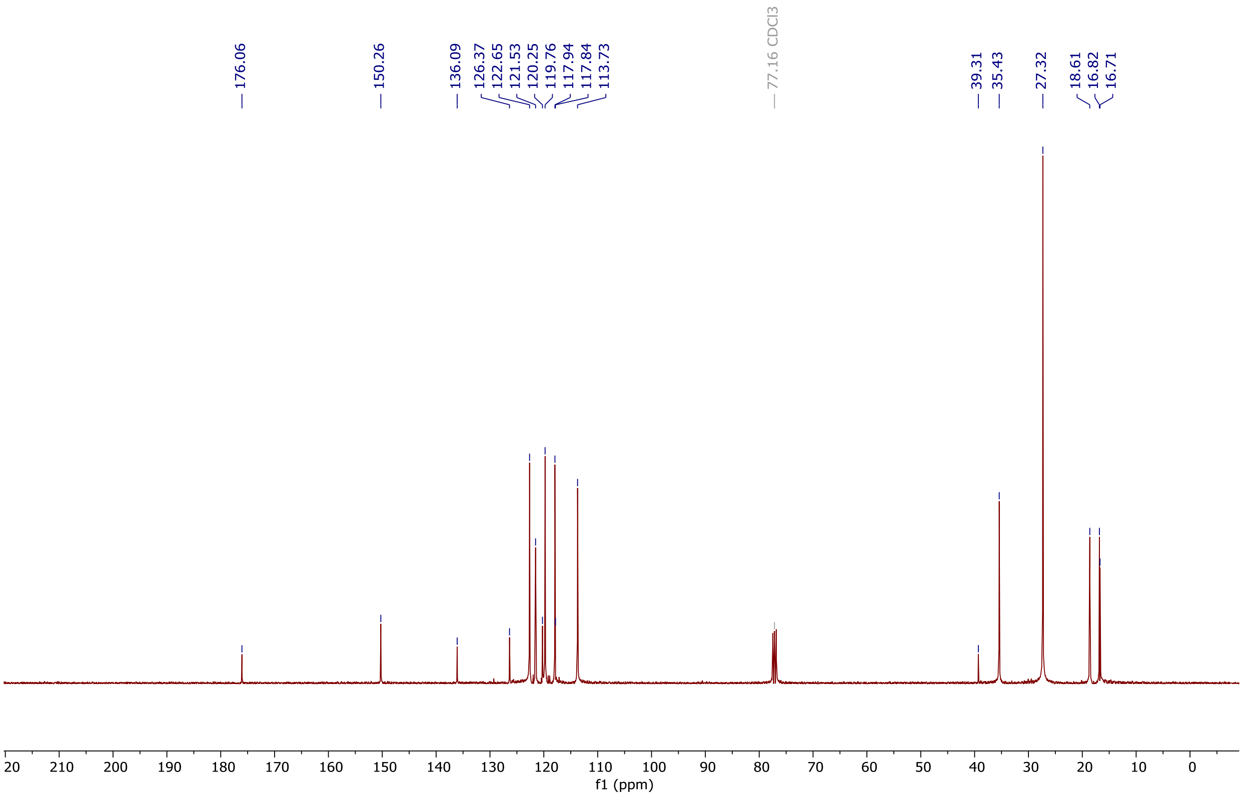

3ac ^{1}H NMR (400 MHz, $CDCl_3$) & ^{13}C NMR (101 MHz, $CDCl_3$):

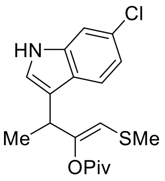

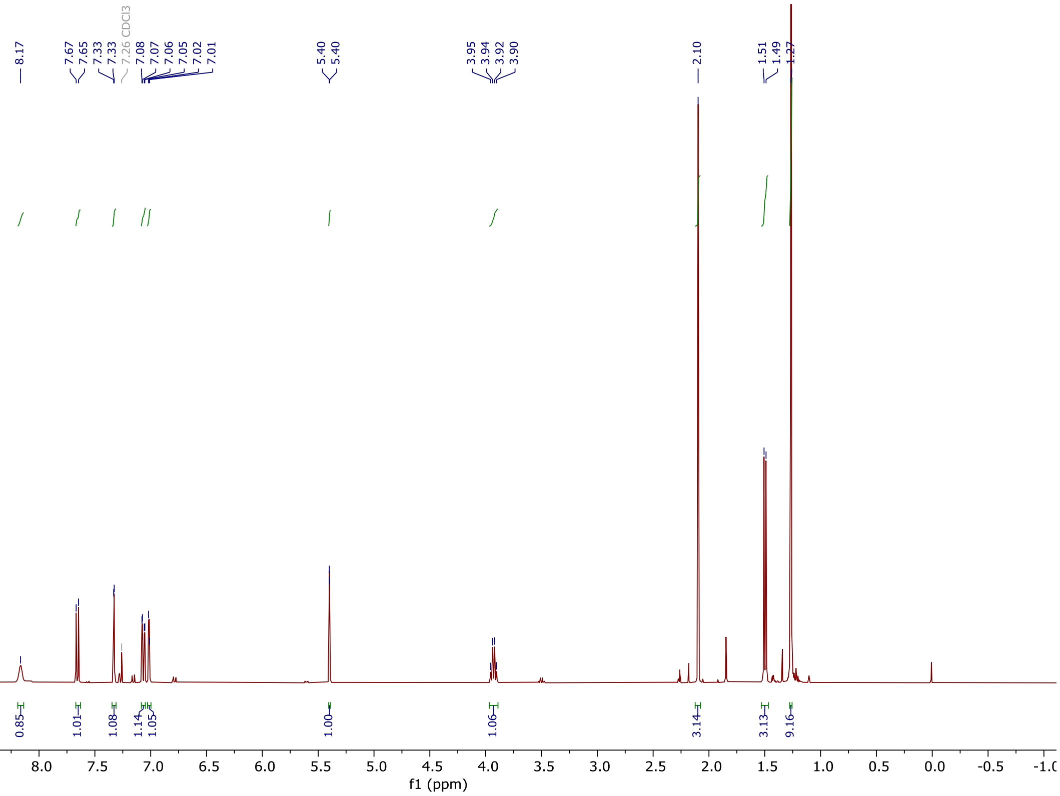

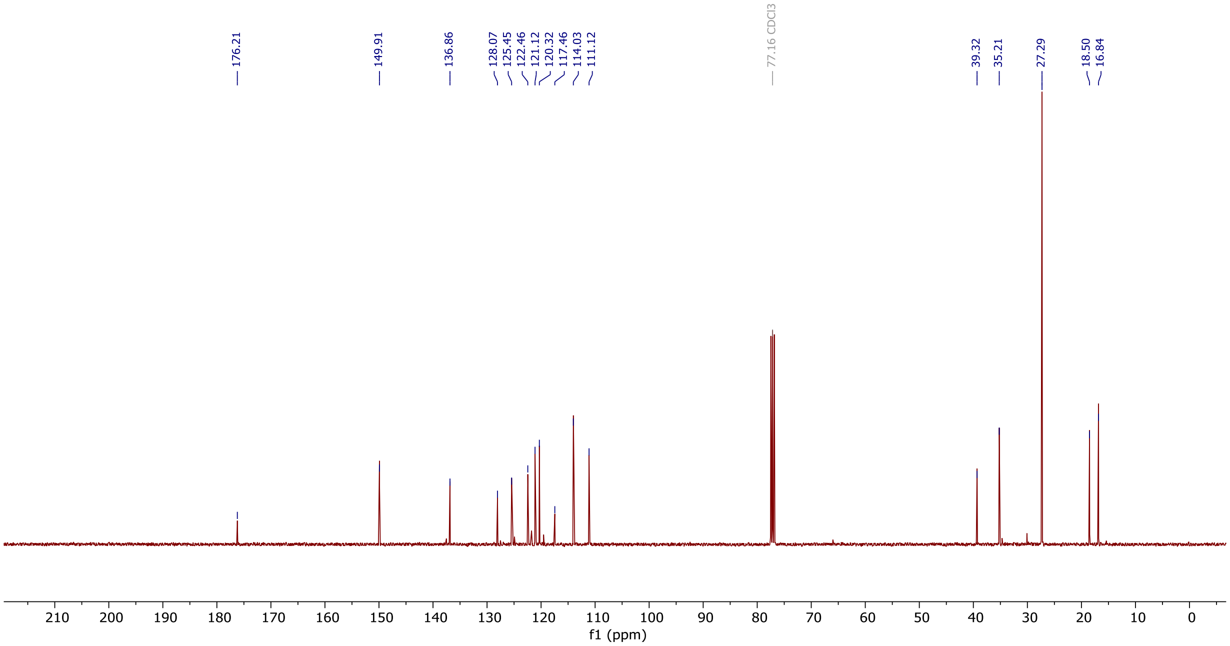

3ad ^{1}H NMR (400 MHz, $CDCl_3$) & ^{13}C NMR (101 MHz, $CDCl_3$):

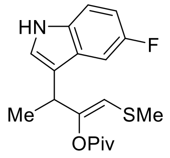

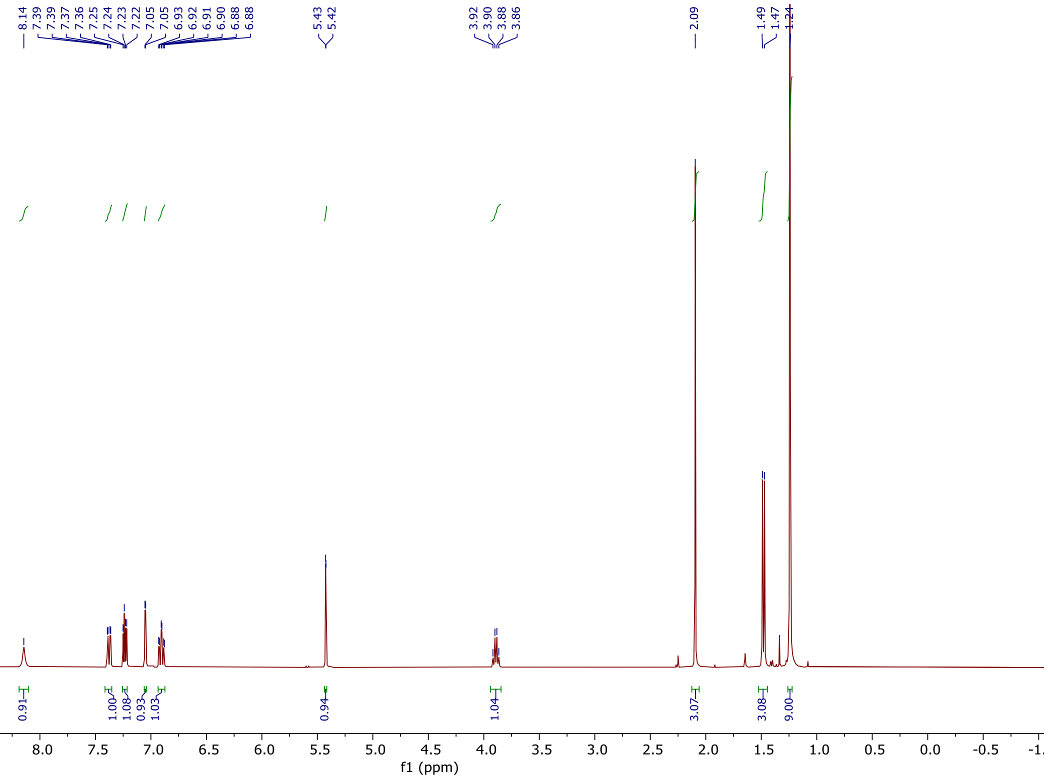

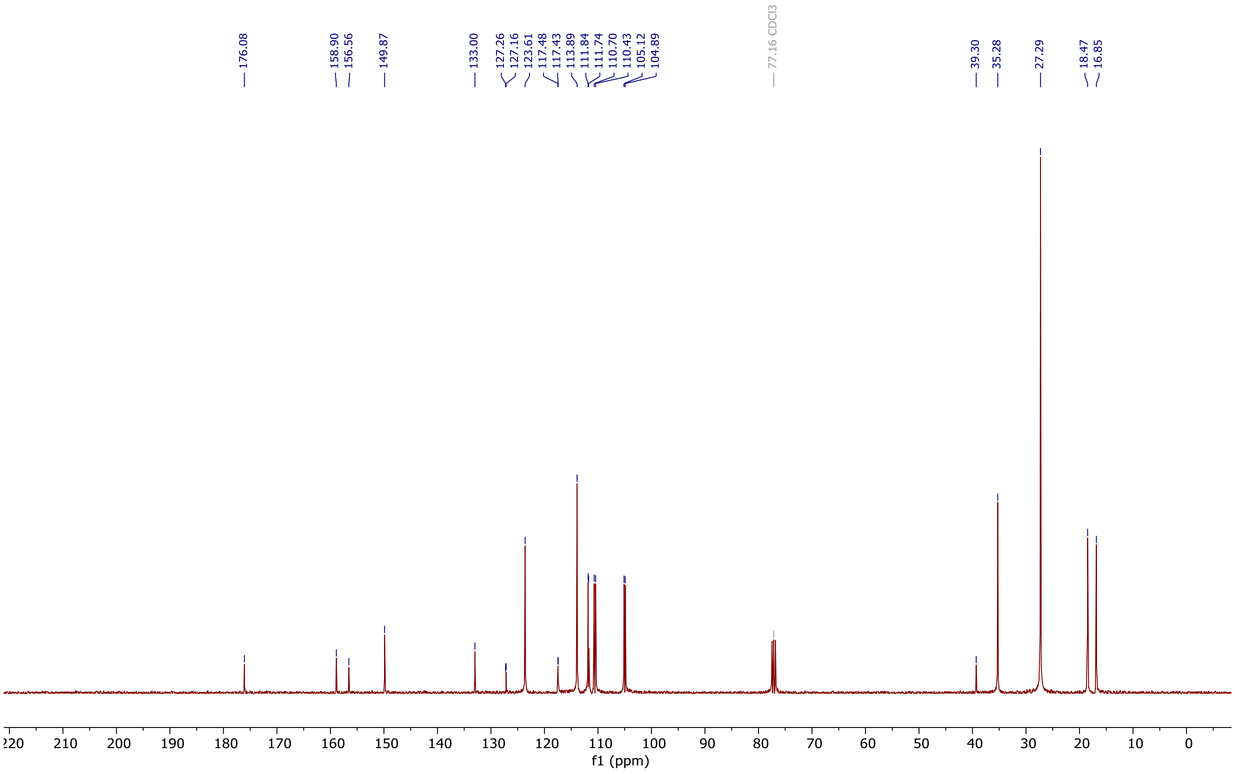

3ae ^{1}H NMR (400 MHz, $CDCl_3$) & ^{13}C NMR (101 MHz, $CDCl_3$):

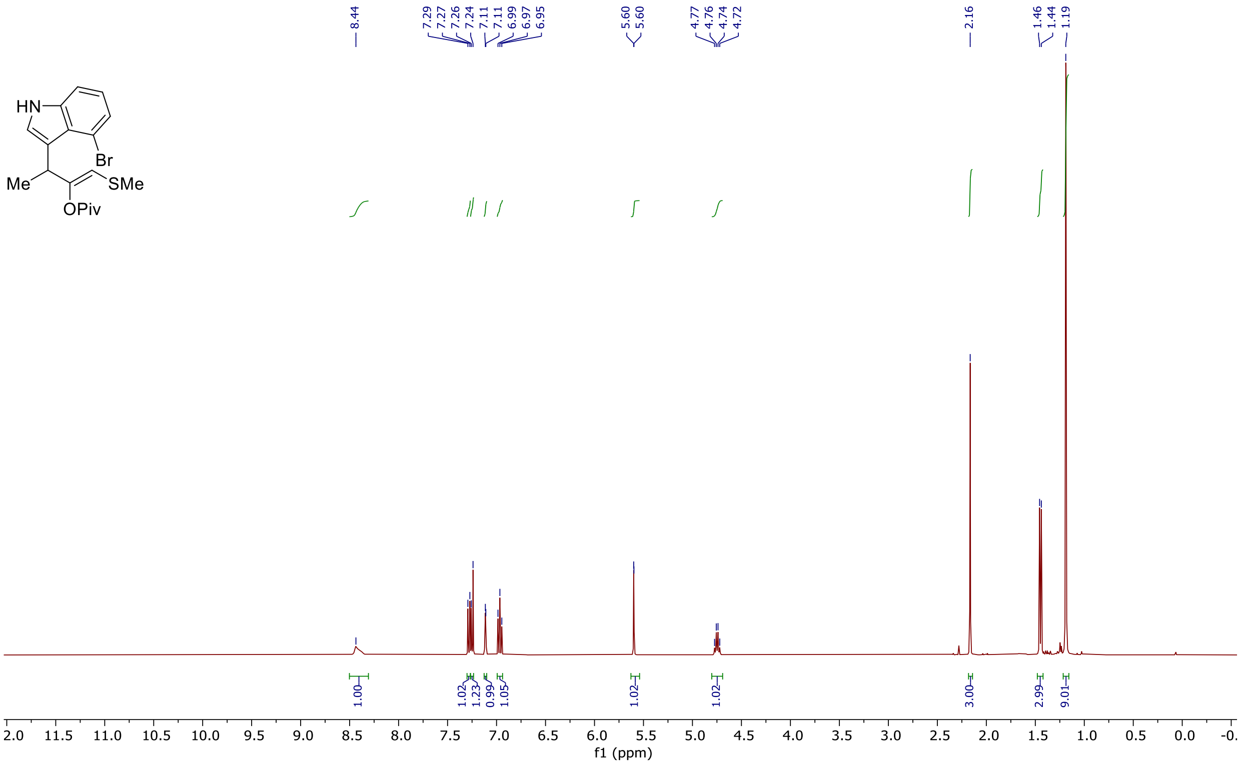

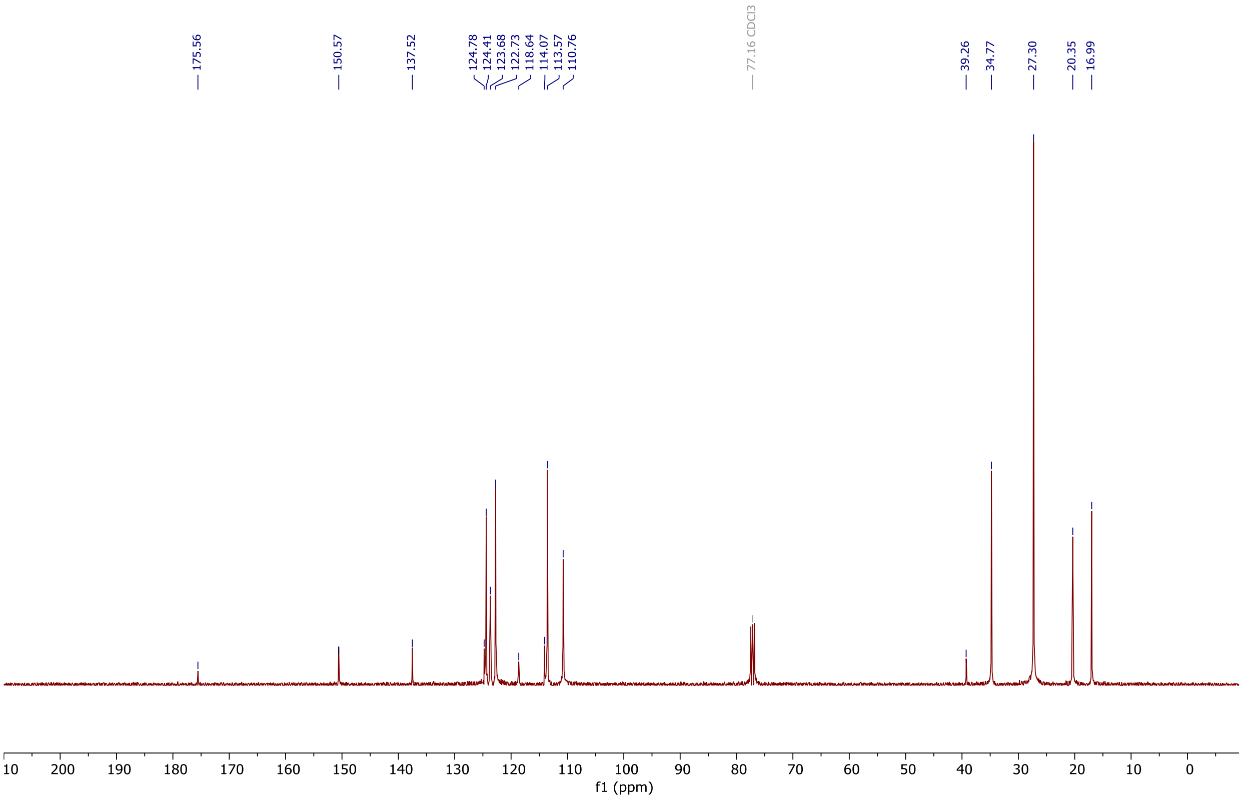

3af ^1H NMR (400 MHz, $CDCl_3$) & ^{13}C NMR (101 MHz, $CDCl_3$):

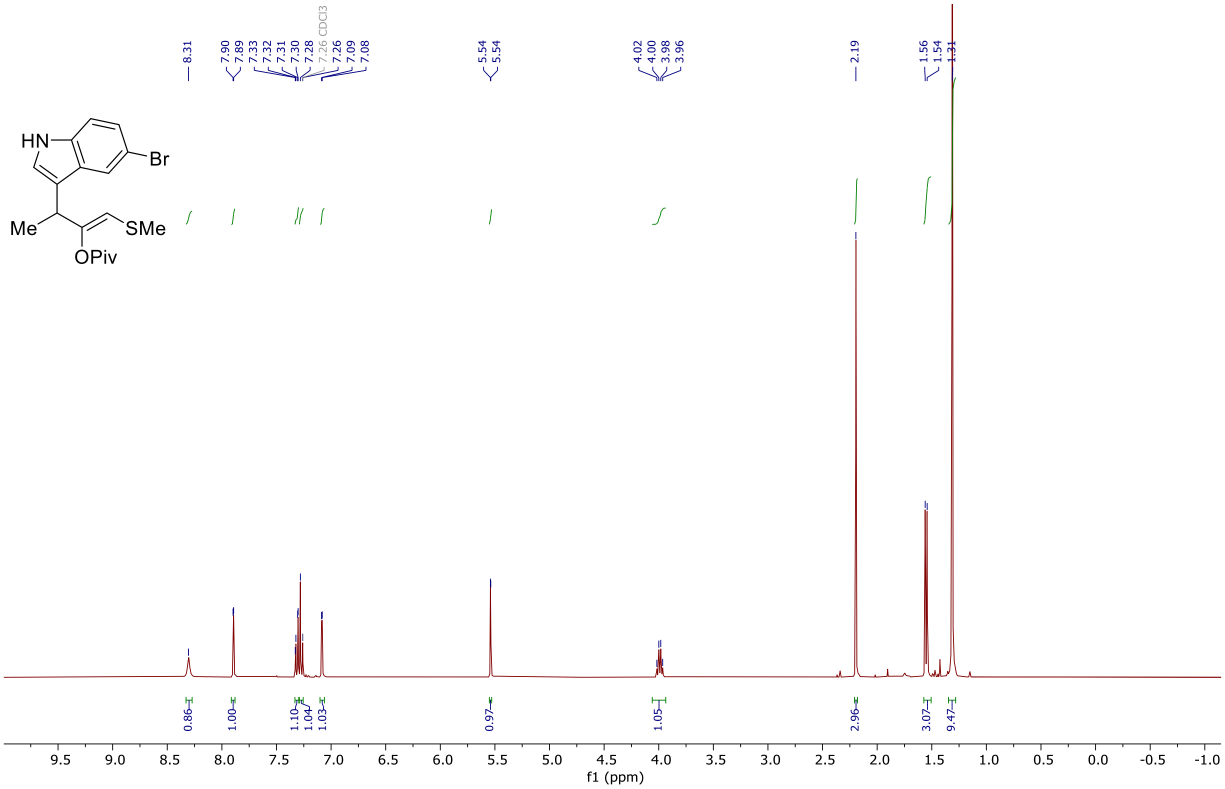

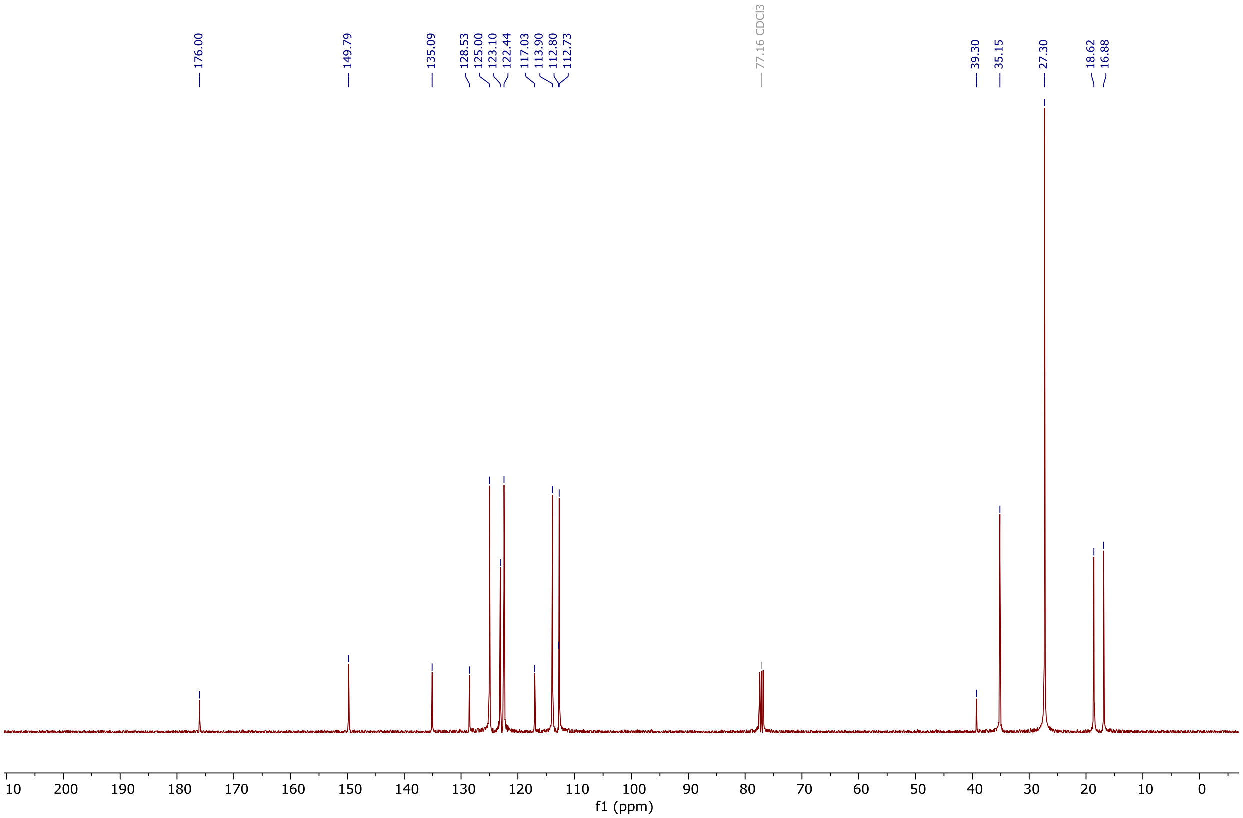

3bg ^{1}H NMR (400 MHz, $CDCl_3$) & ^{13}C NMR (101 MHz, $CDCl_3$):

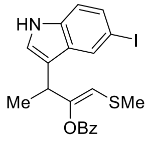

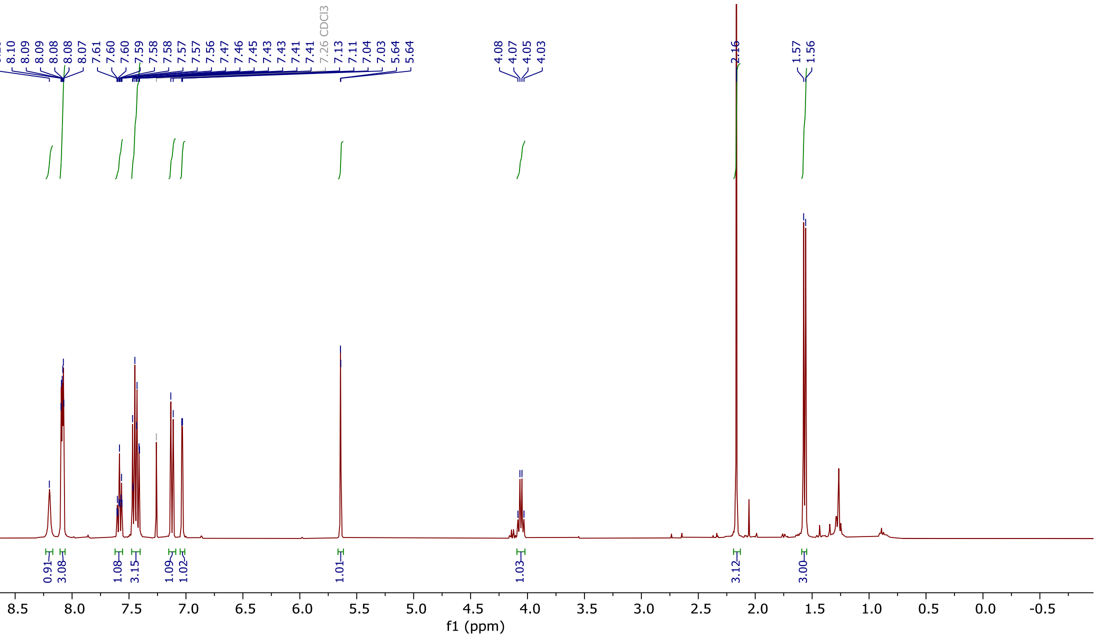

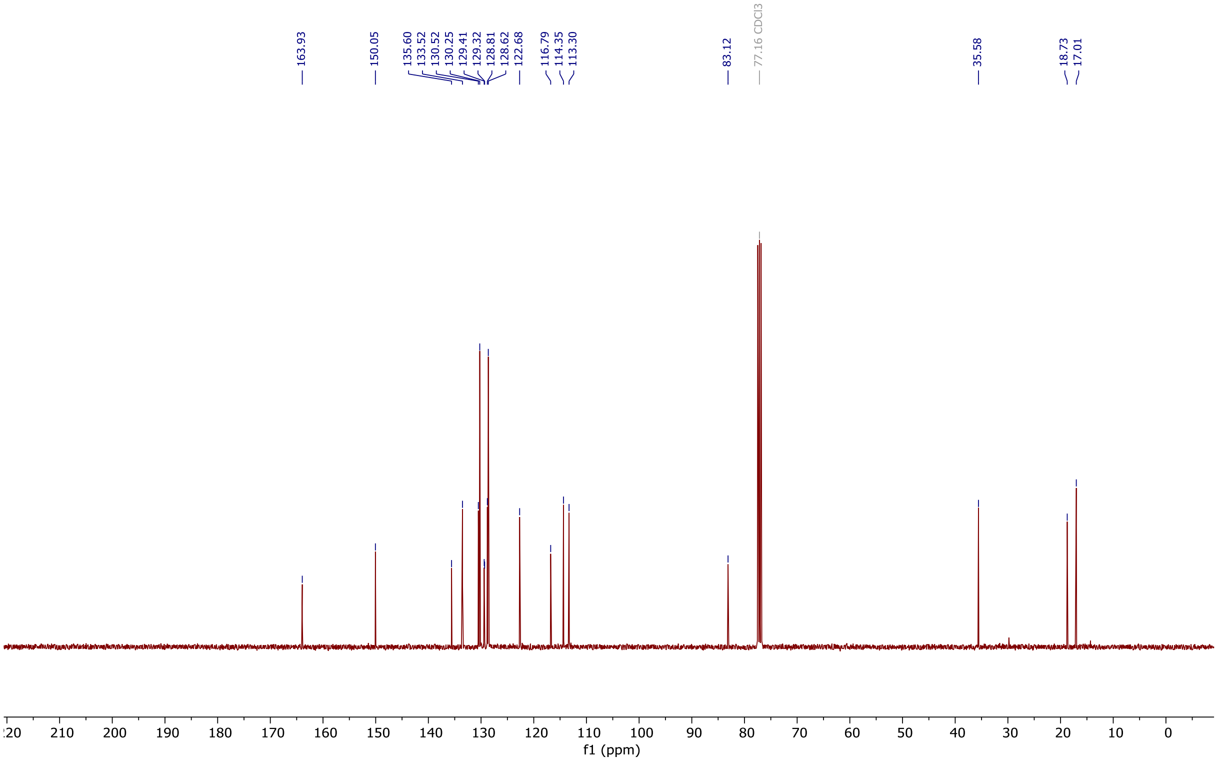

3ah ^{1}H NMR (300 MHz, $CDCl_3$) & ^{13}C NMR (101 MHz, $CDCl_3$):

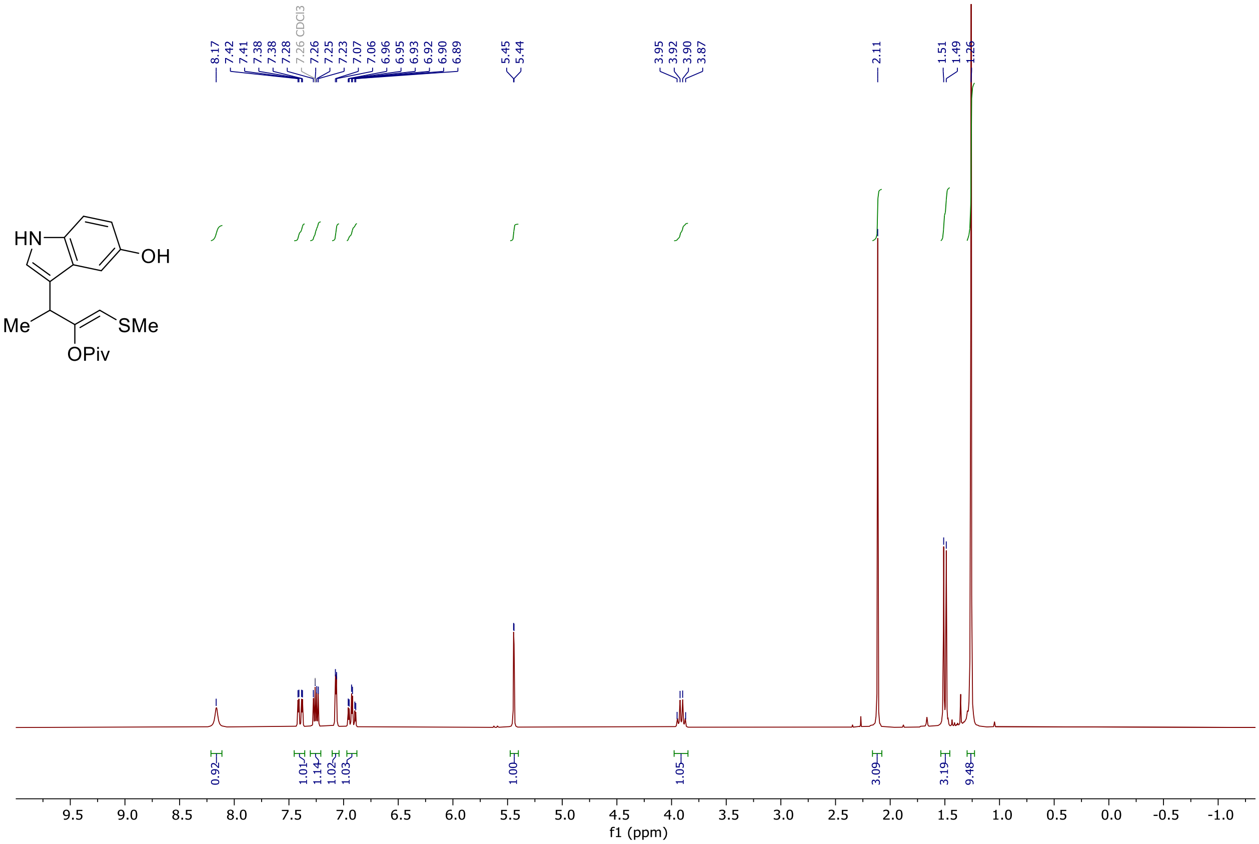

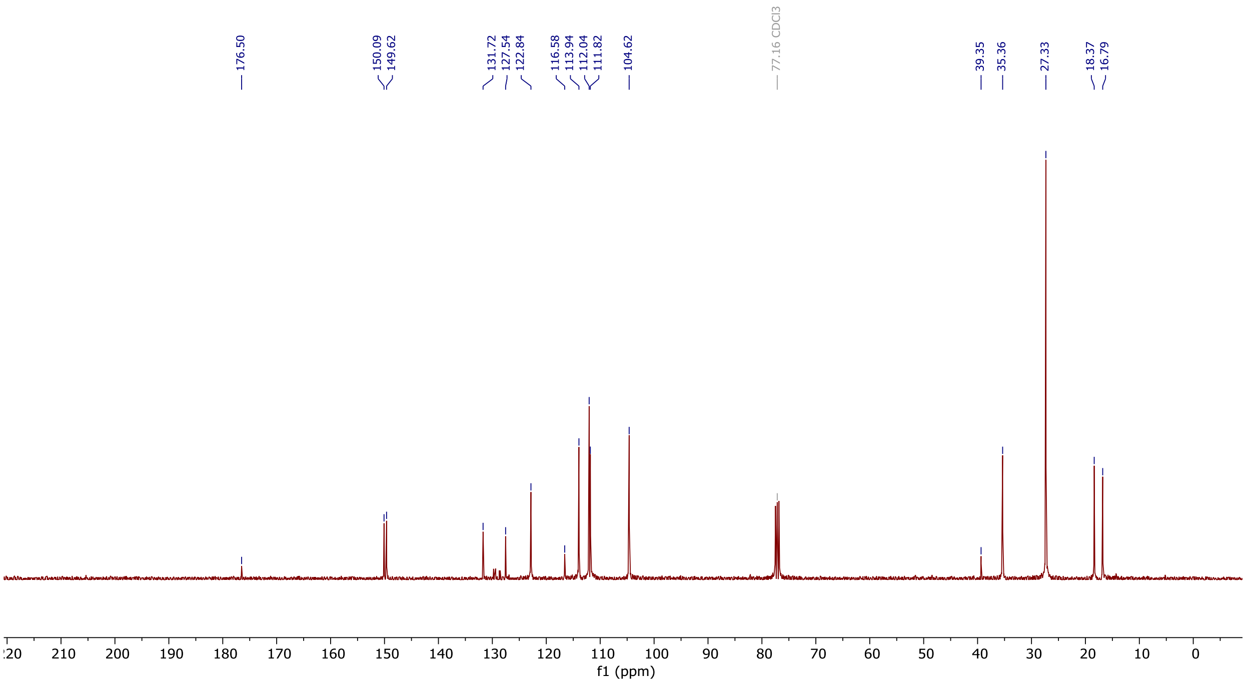

3ai ^{1}H NMR (400 MHz, $CDCl_3$) & ^{13}C NMR (101 MHz, $CDCl_3$):

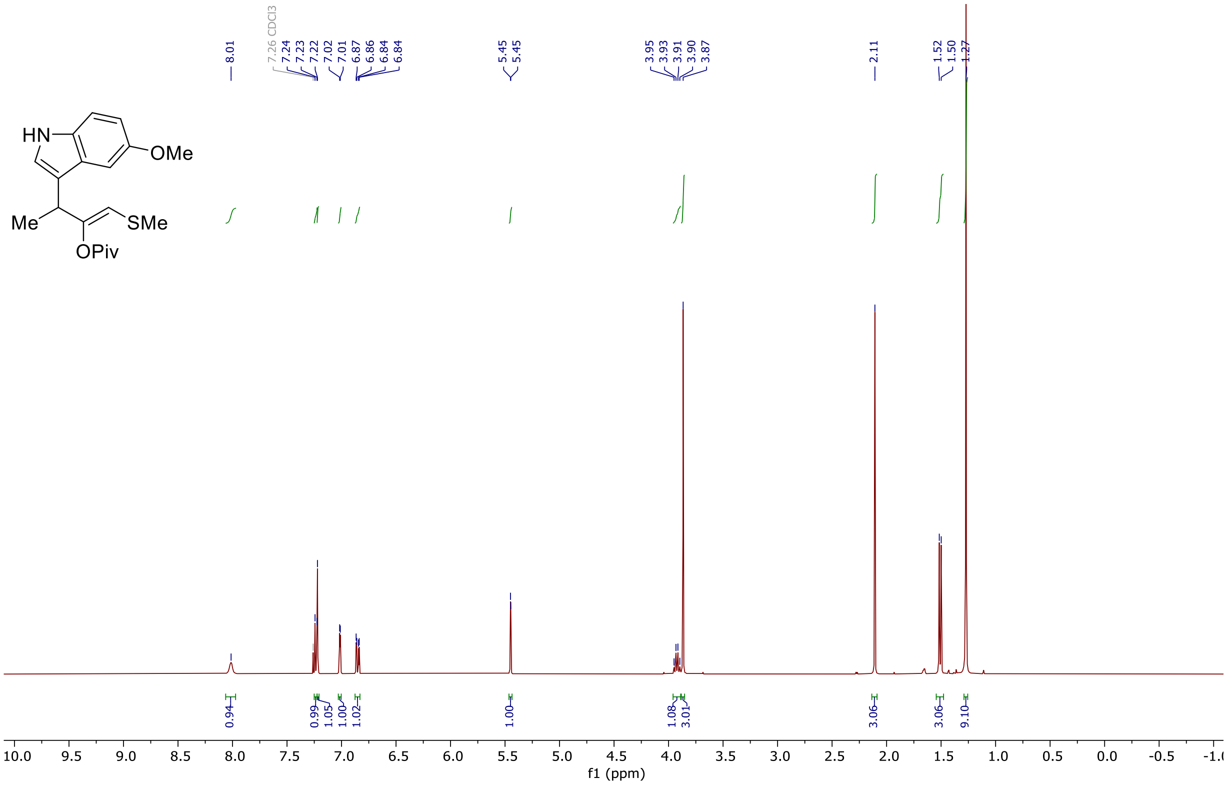

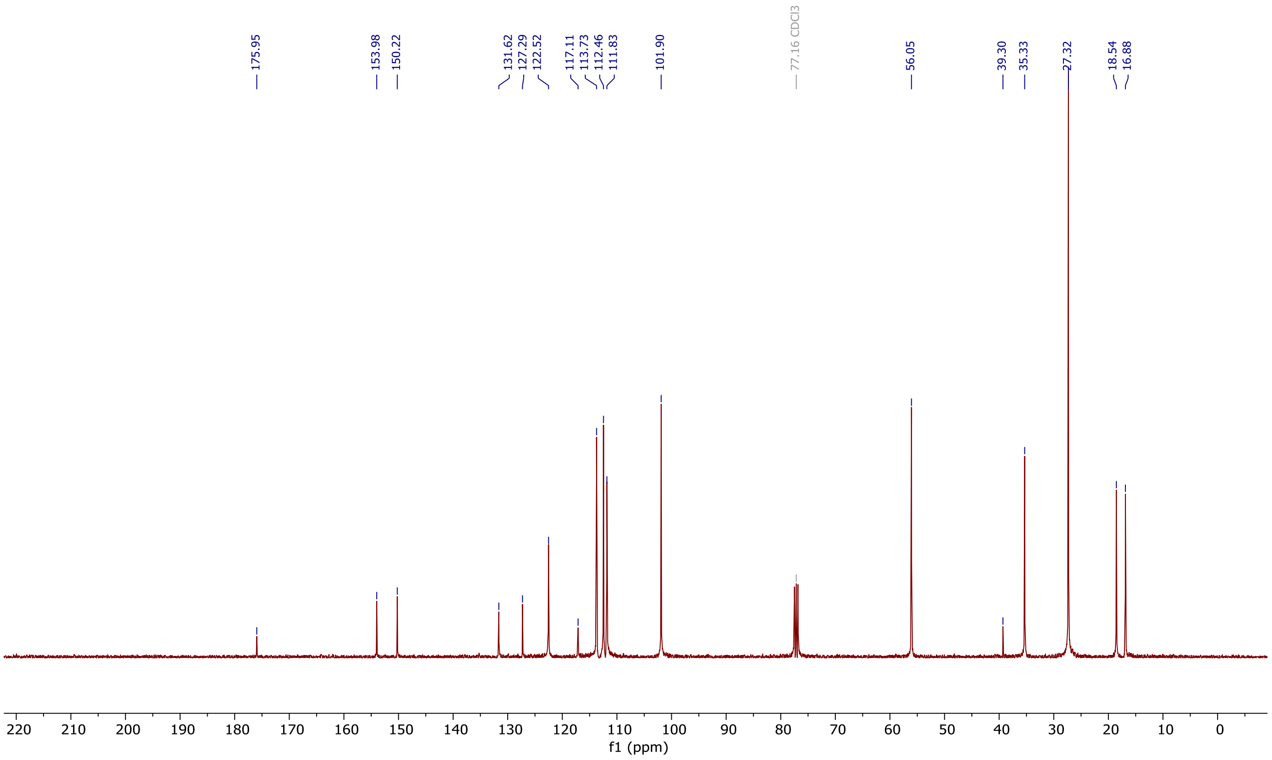

3ak ^{1}H NMR (400 MHz, $CDCl_3$) & ^{13}C NMR (101 MHz, $CDCl_3$):

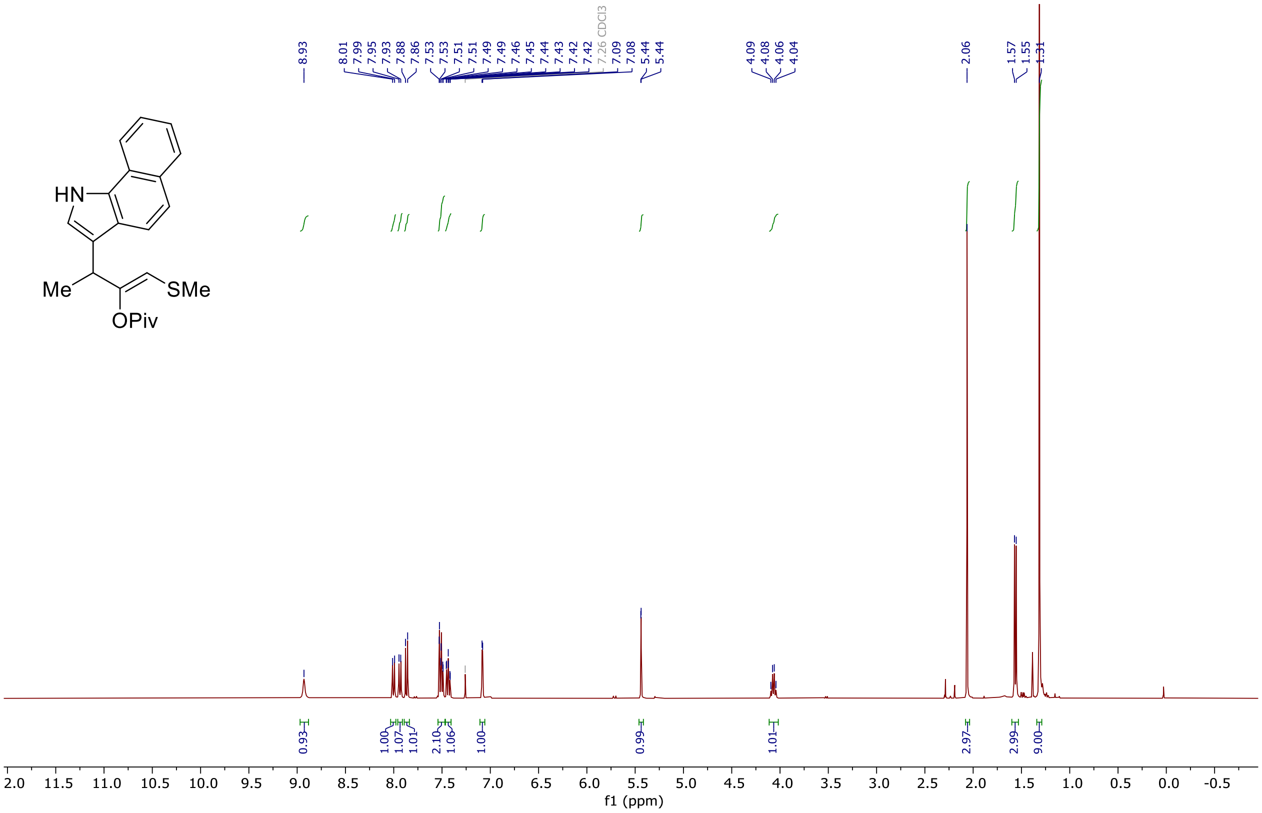

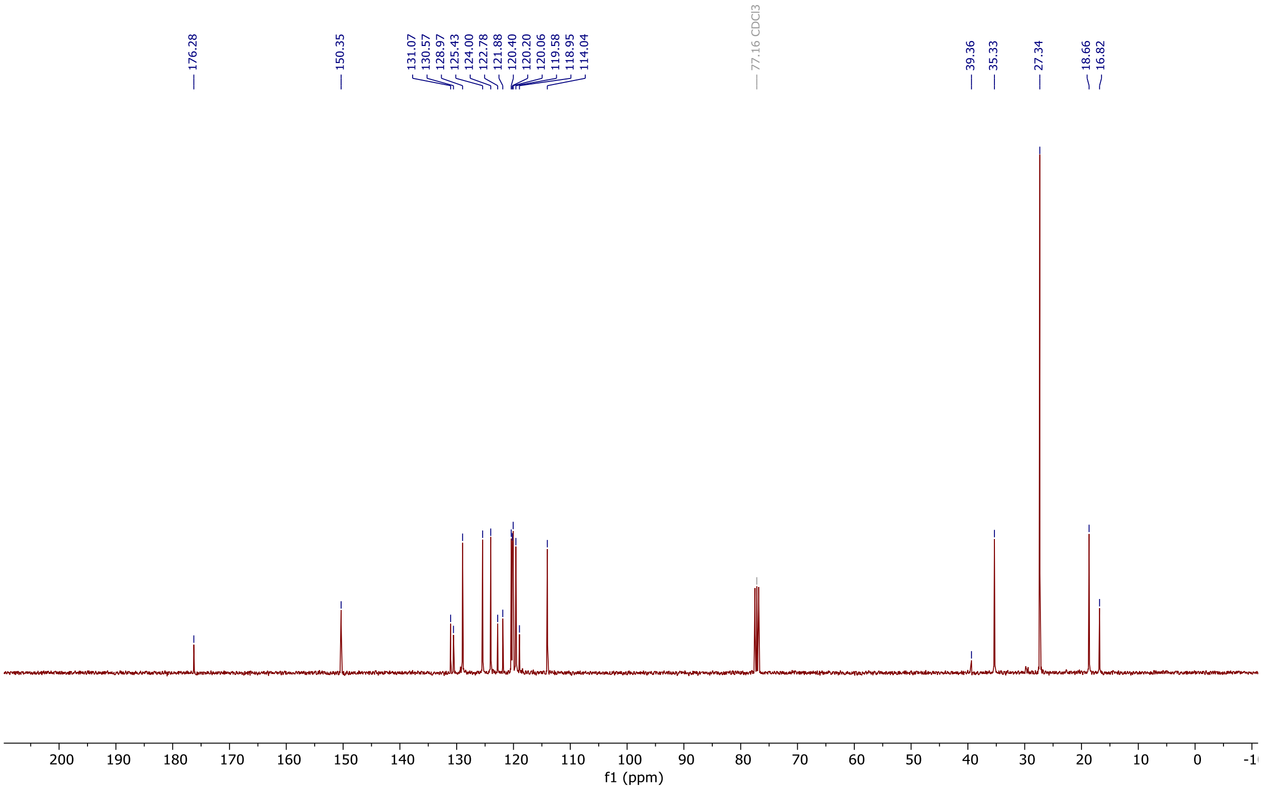

3al ^{1}H NMR (400 MHz, $CDCl_3$) & ^{13}C NMR (101 MHz, $CDCl_3$):

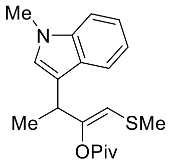

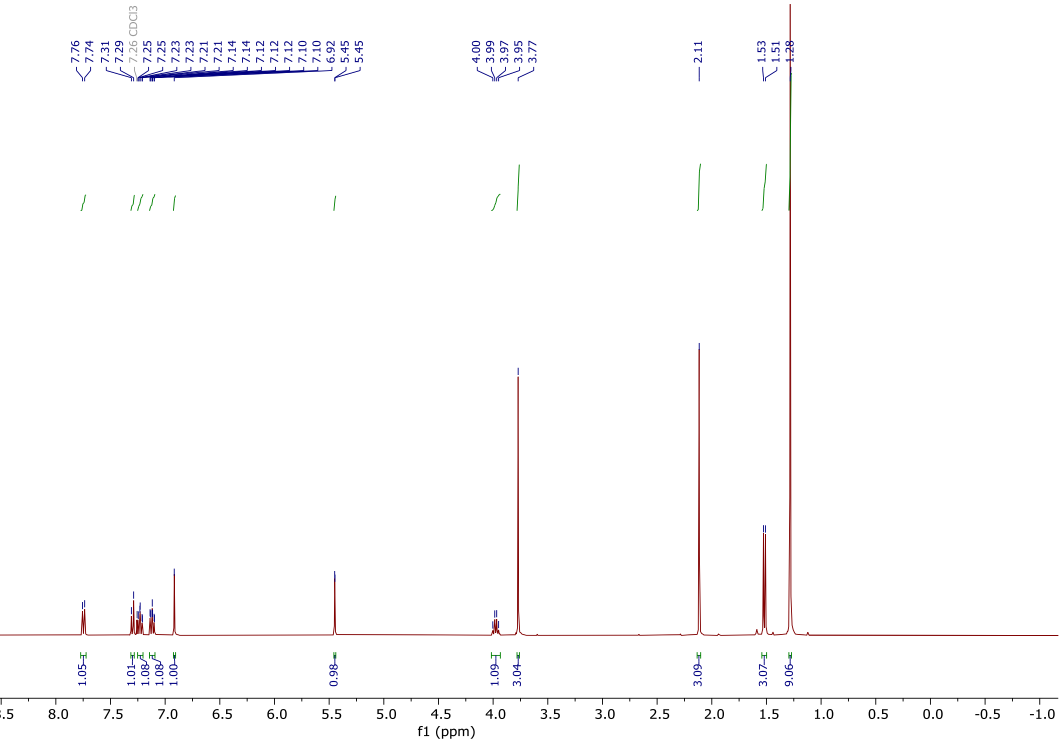

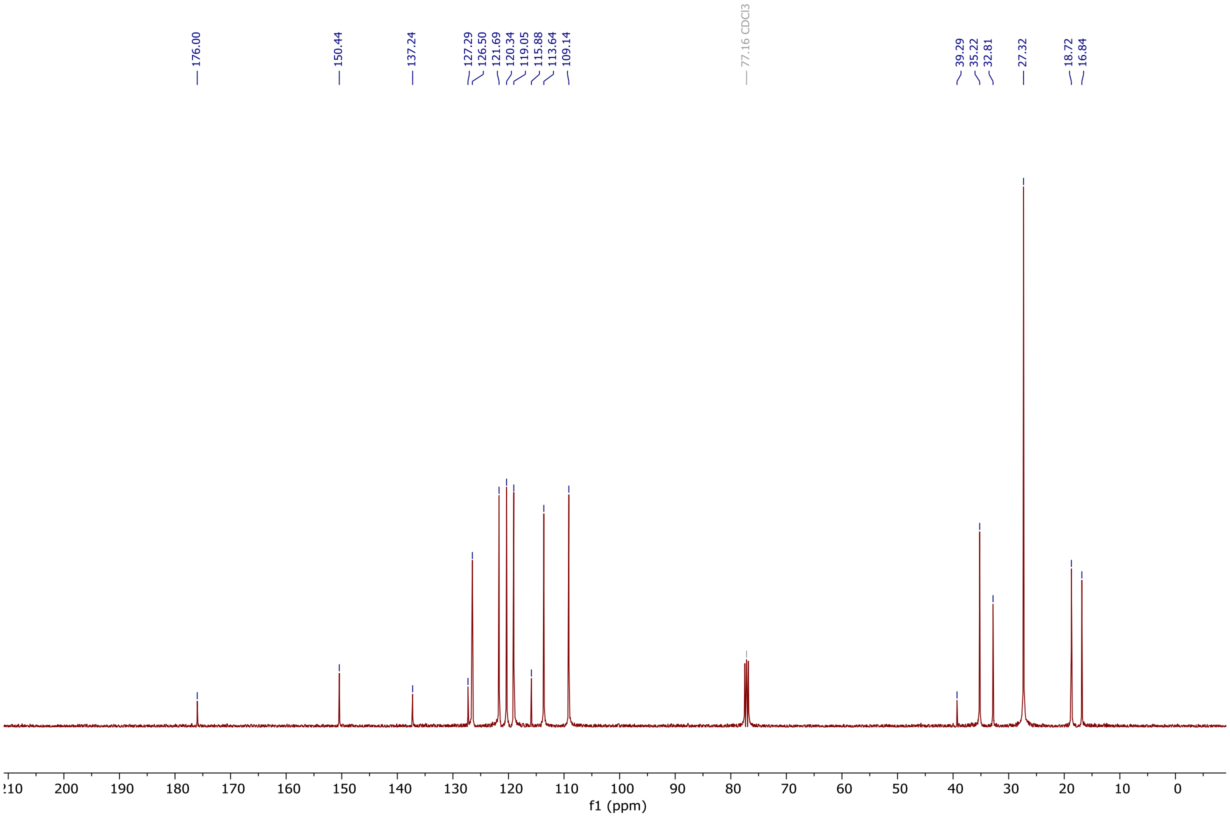

3bm ^{1}H NMR (400 MHz, $CDCl_3$) & ^{13}C NMR (101 MHz, $CDCl_3$):

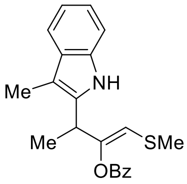

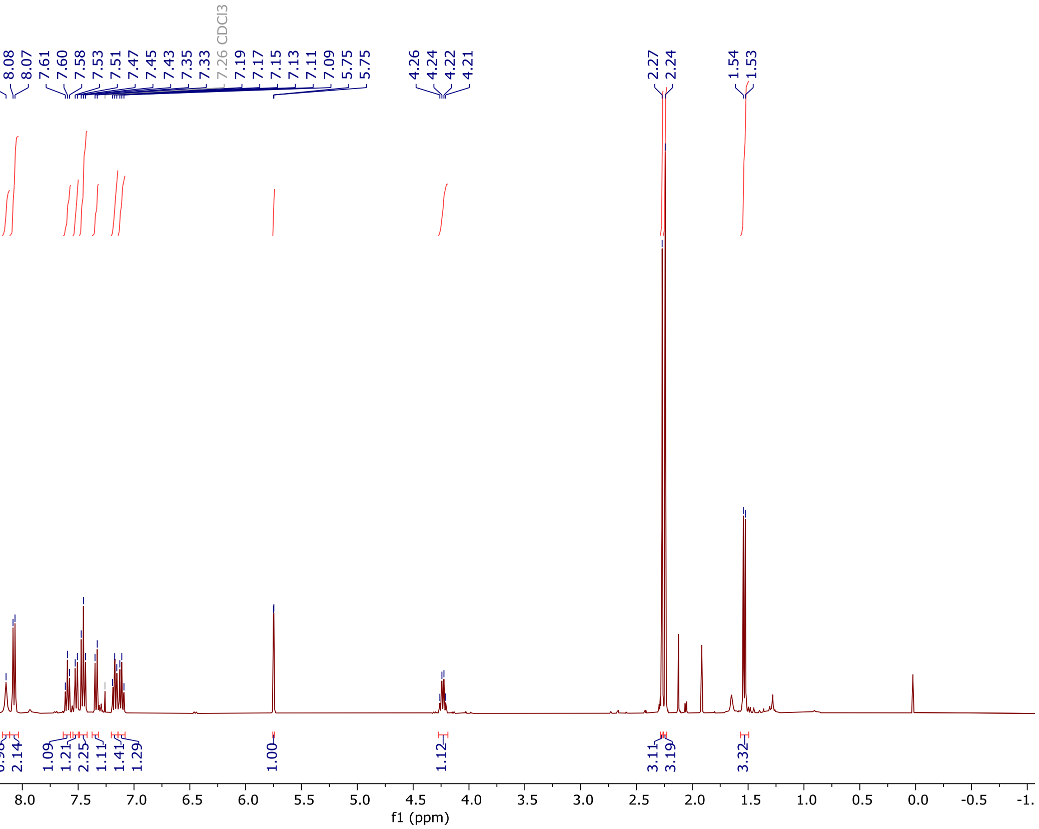

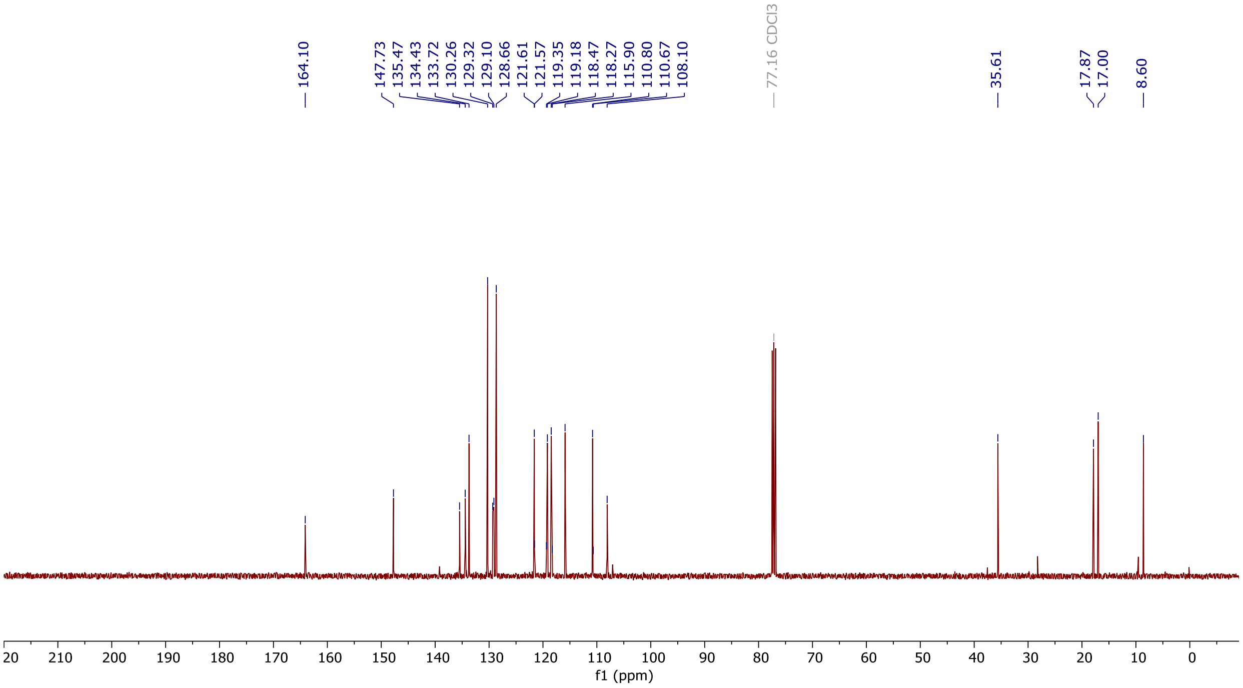

3am ^{1}H NMR (400 MHz, $CDCl_3$) & ^{13}C NMR (101 MHz, $CDCl_3$):

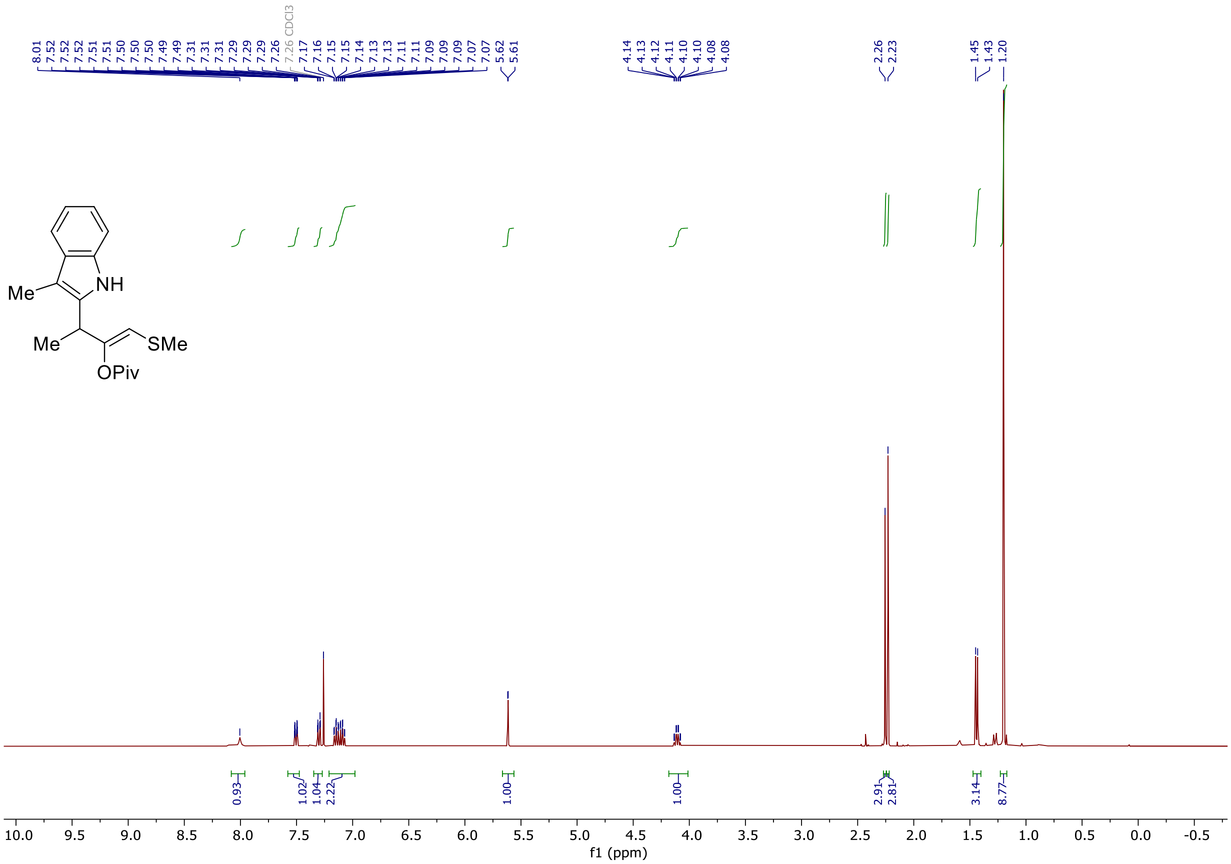

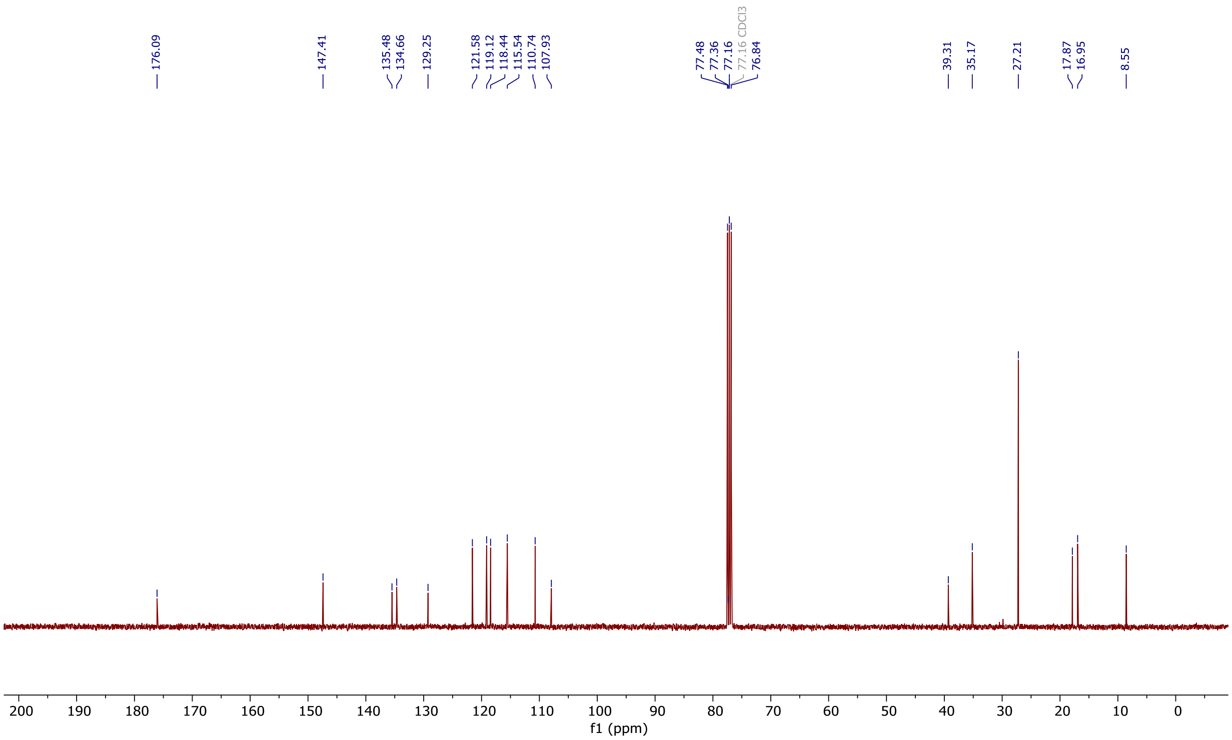

3an ^{1}H NMR (400 MHz, $CDCl_3$) & ^{13}C NMR (101 MHz, $CDCl_3$):

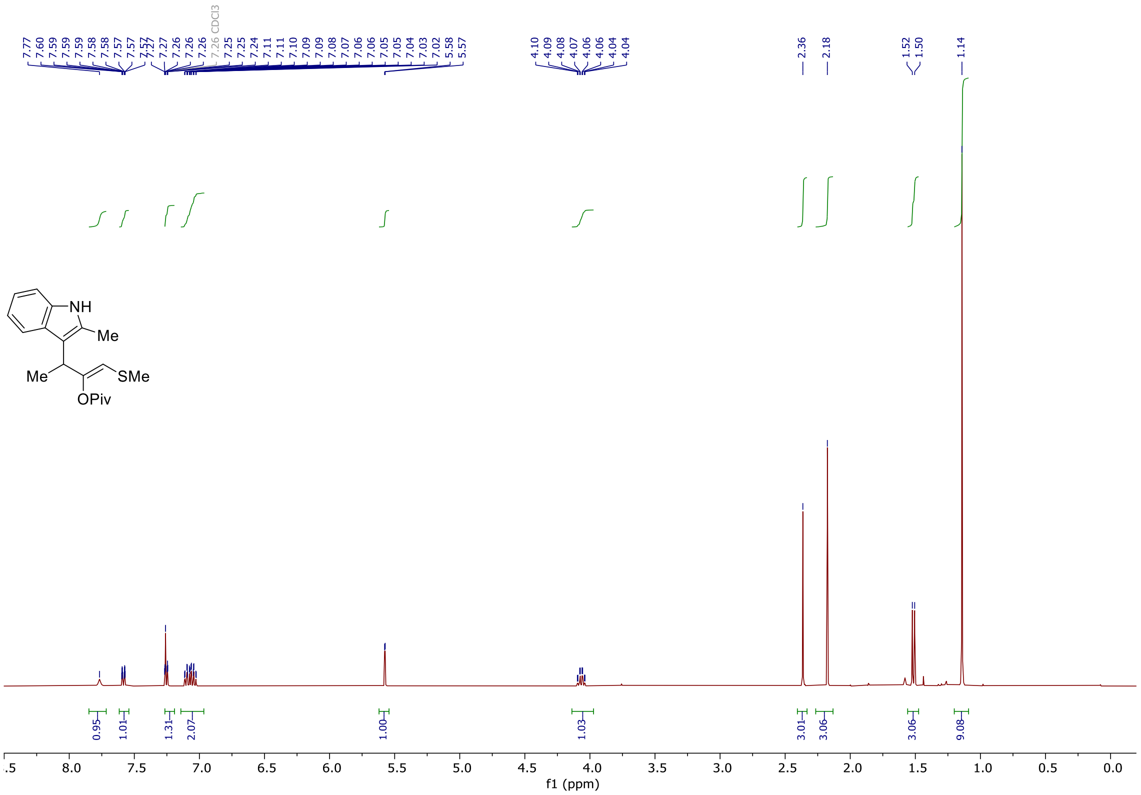

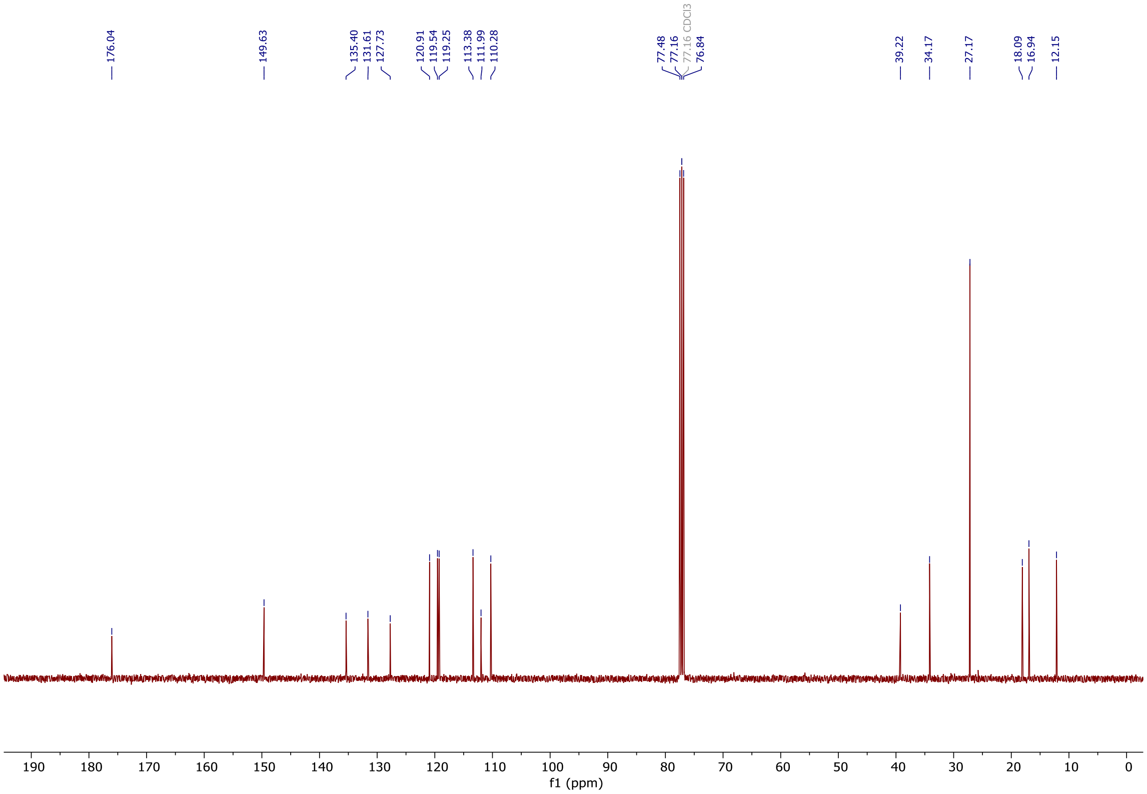

3ca ^{1}H NMR (400 MHz, $CDCl_3$) & ^{13}C NMR (101 MHz, $CDCl_3$):

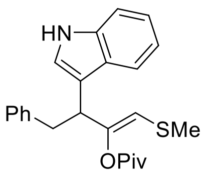

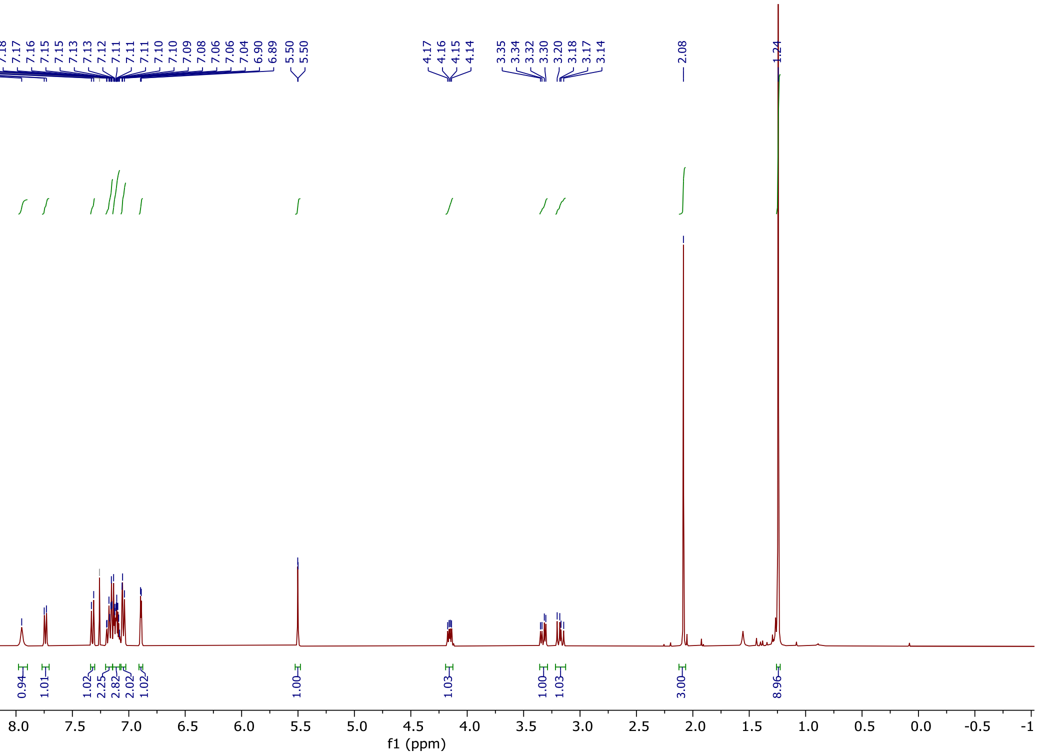

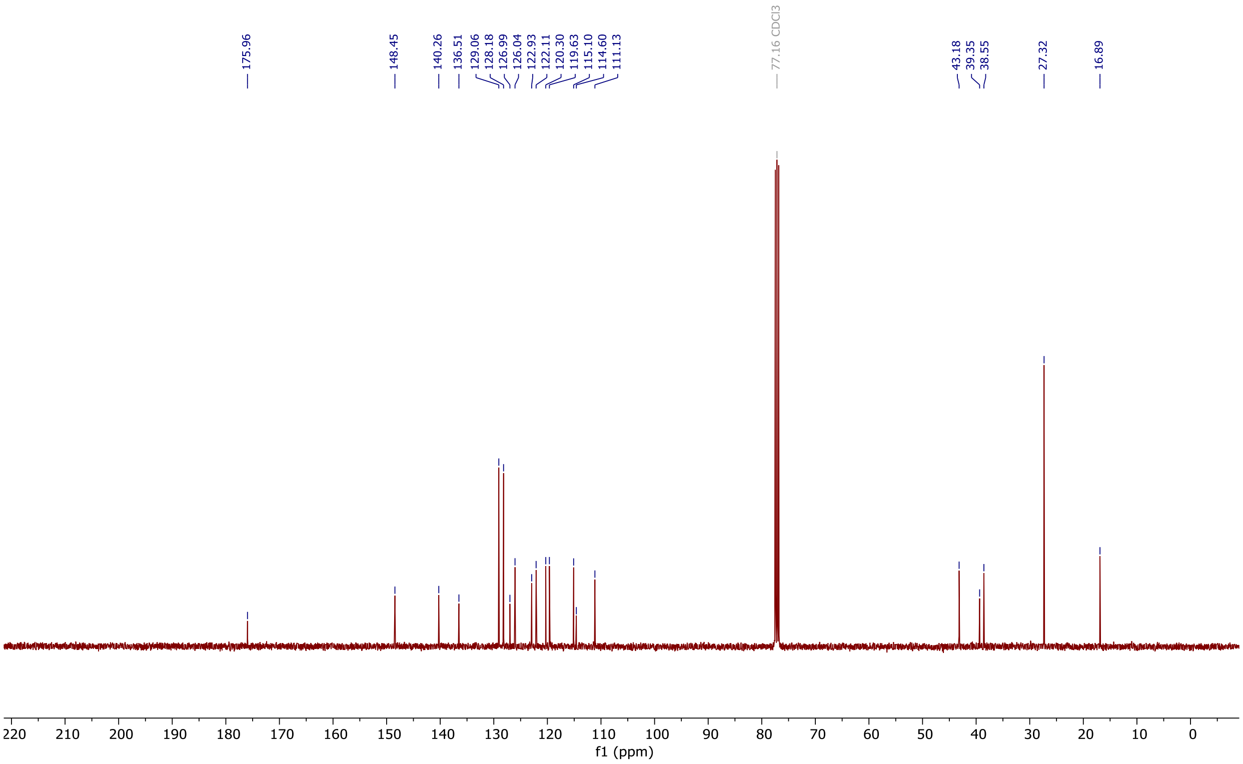

3da ^{1}H NMR (400 MHz, $CDCl_3$) & ^{13}C NMR (101 MHz, $CDCl_3$):

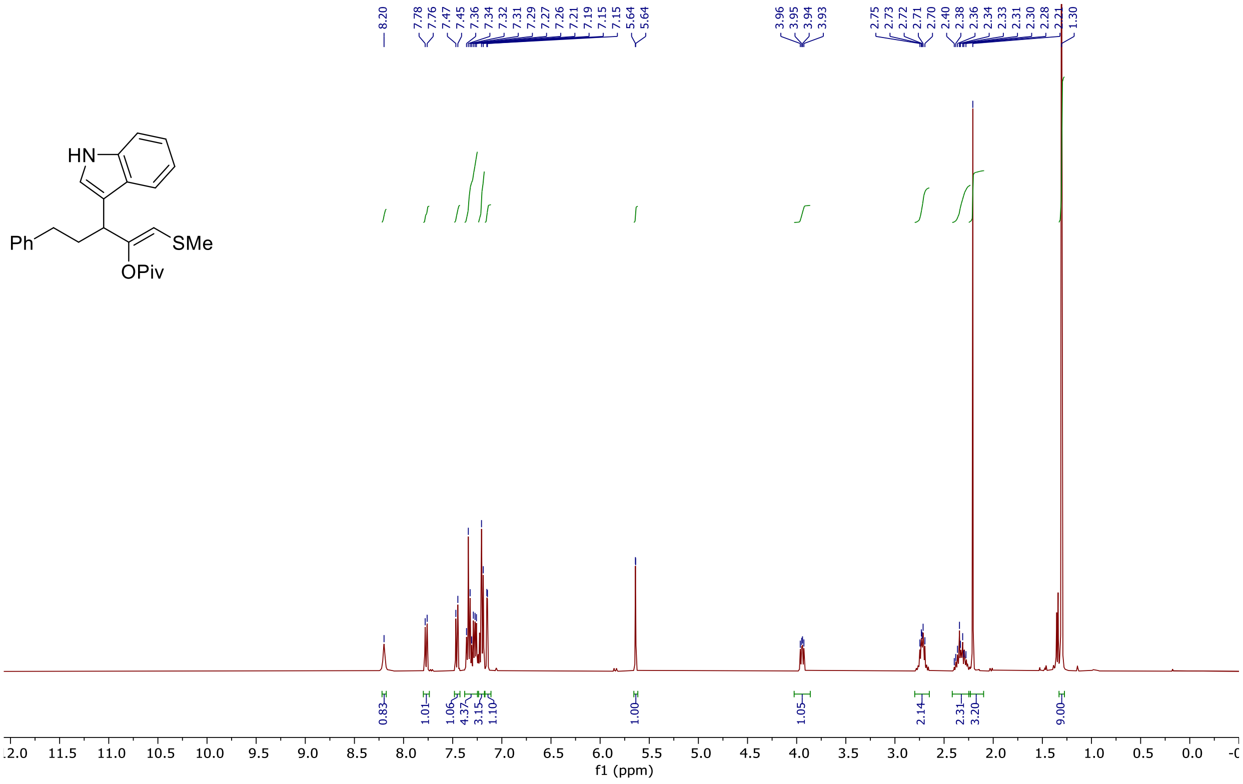

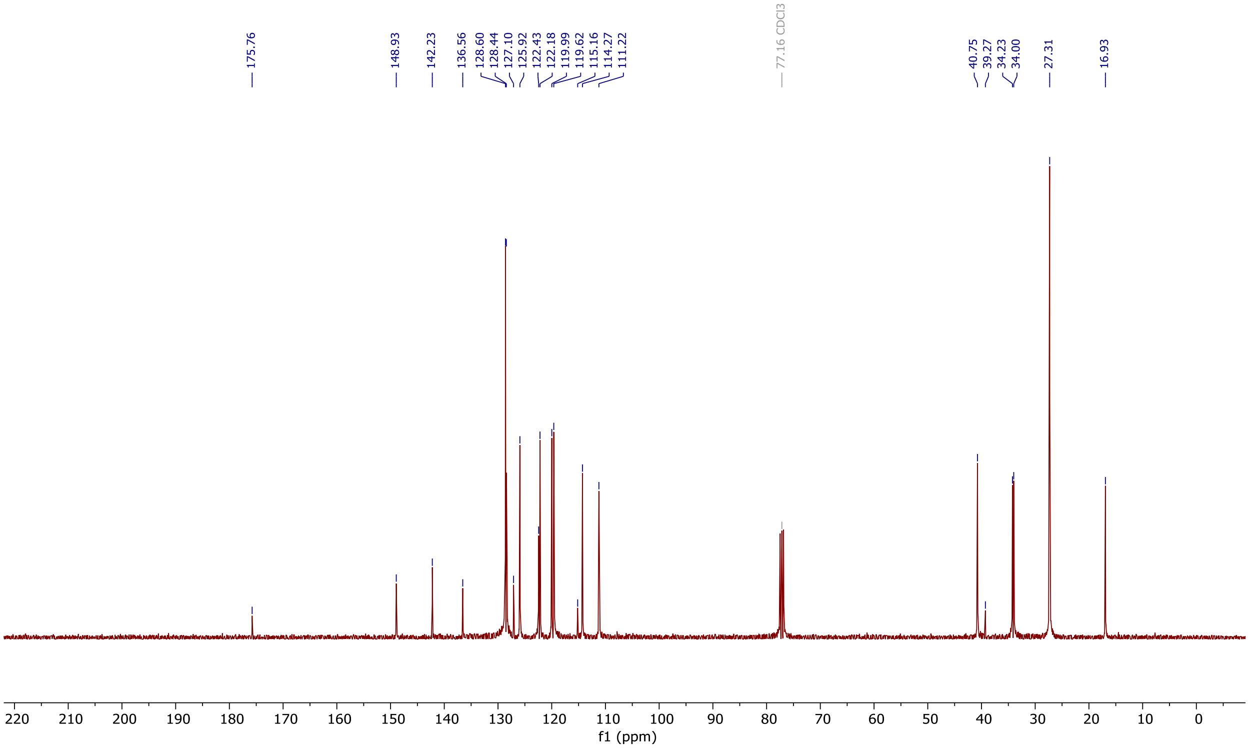

3ea ^{1}H NMR (400 MHz, $CDCl_3$) & ^{13}C NMR (101 MHz, $CDCl_3$):

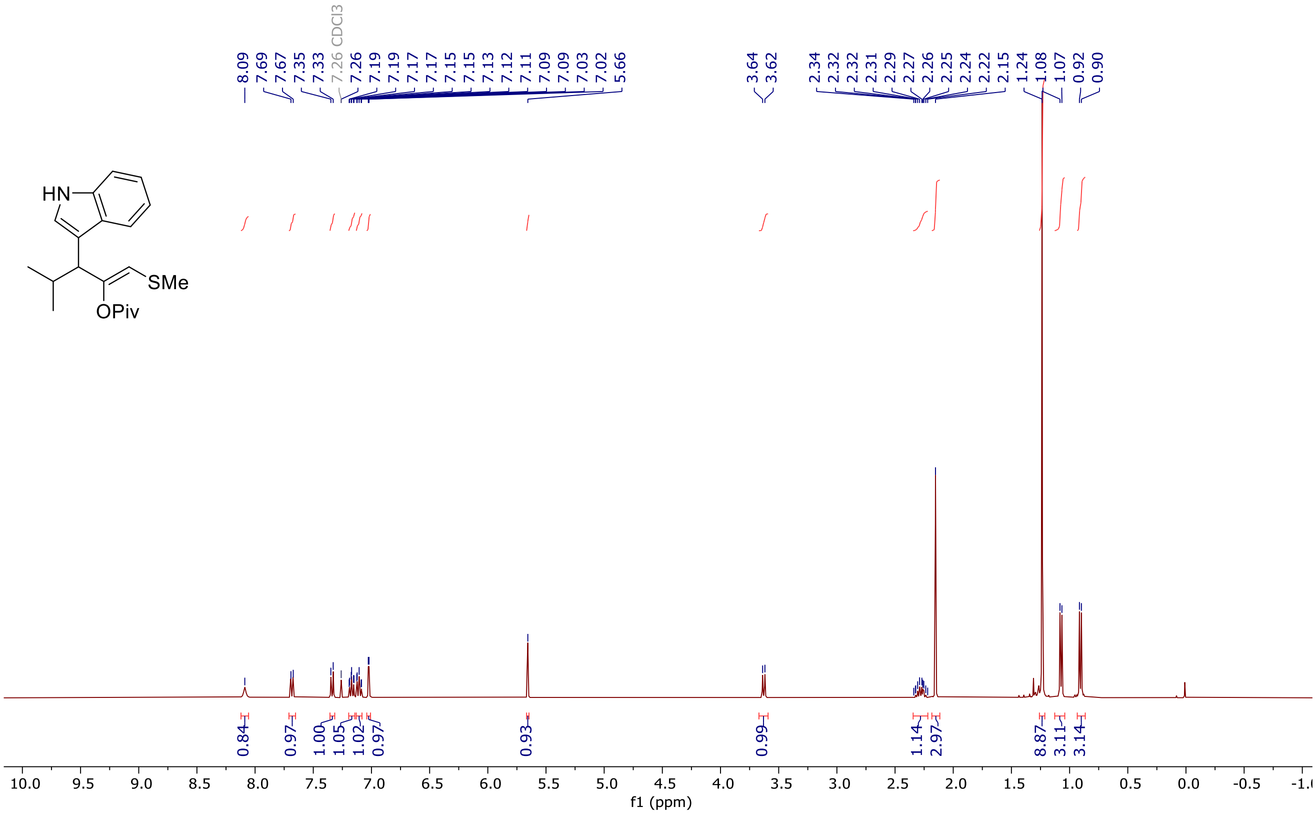

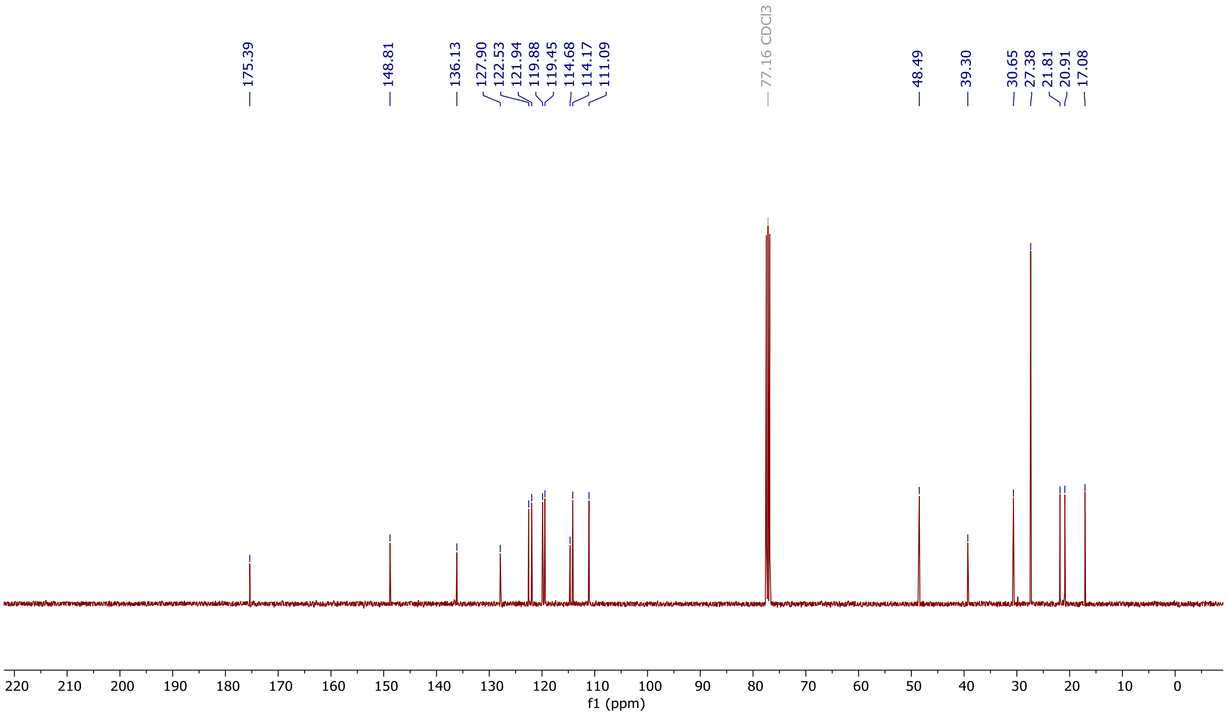

3fa ^{1}H NMR (400 MHz, $CDCl_3$) & ^{13}C NMR (101 MHz, $CDCl_3$):

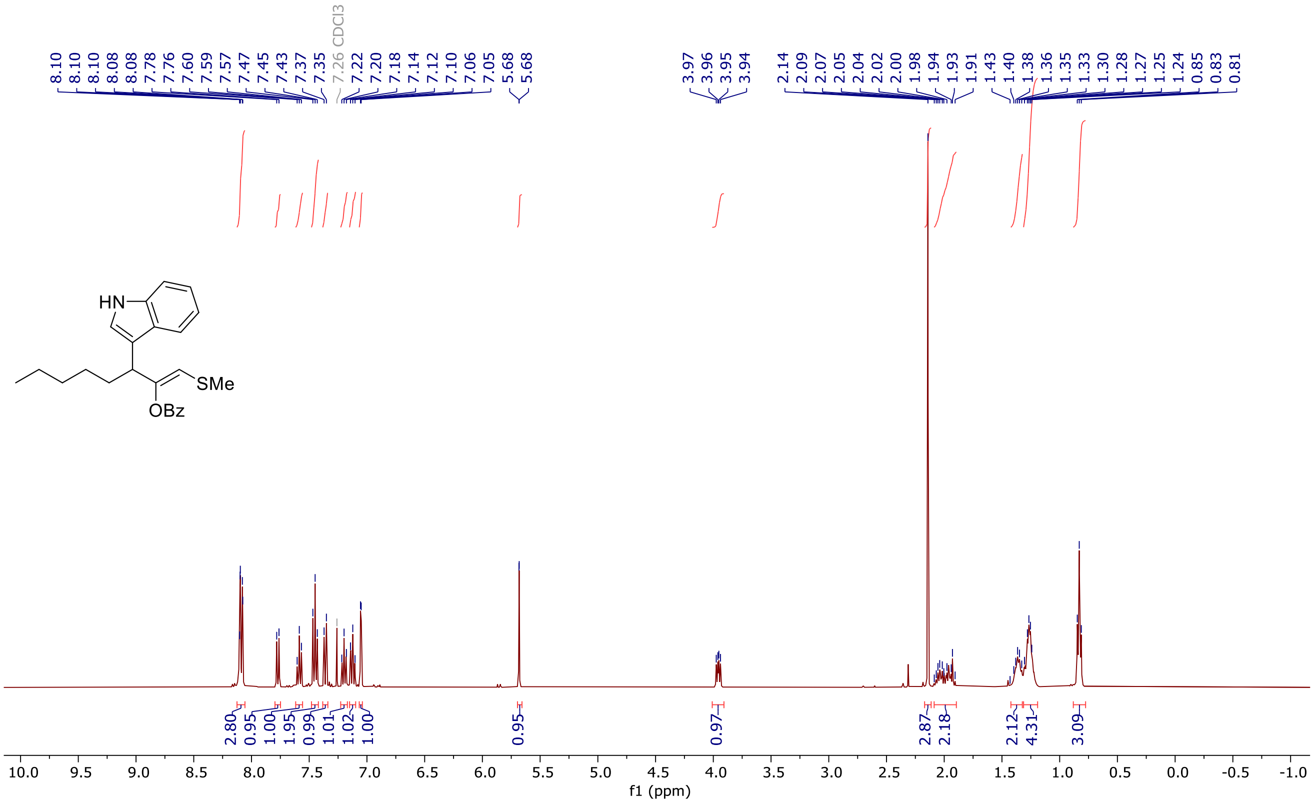

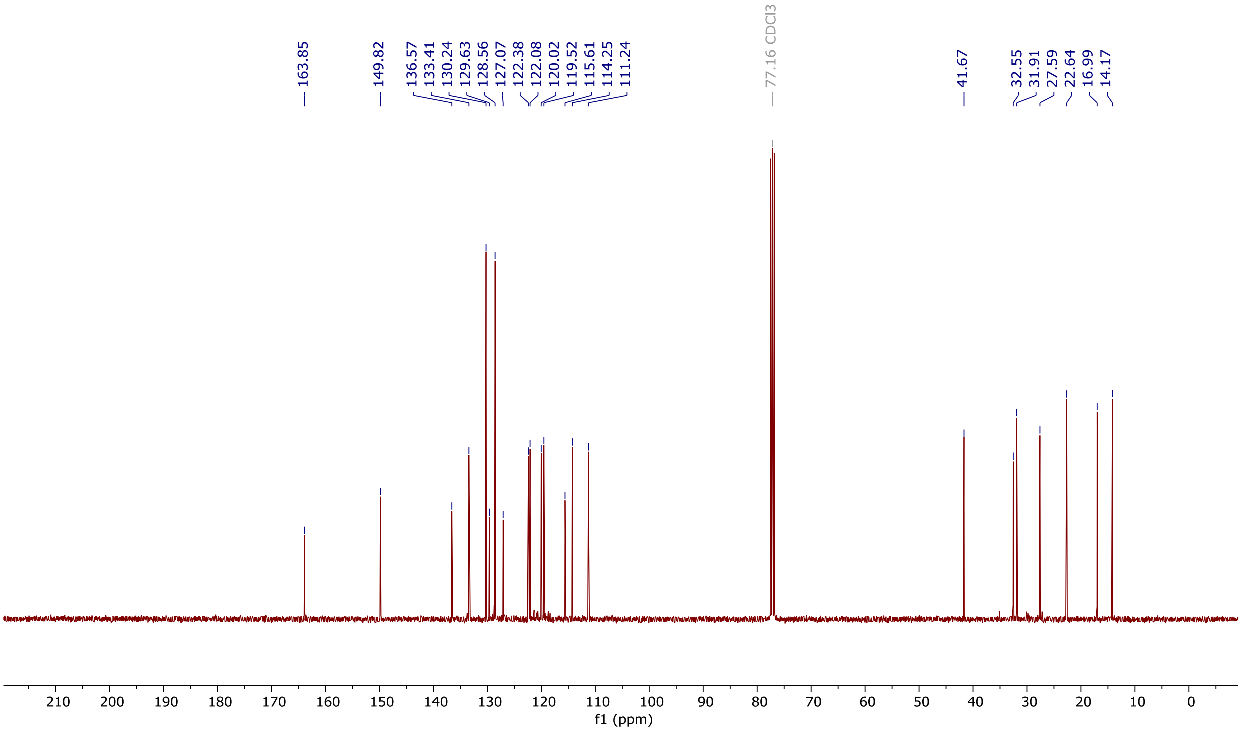

3ga ^{1}H NMR (400 MHz, $CDCl_3$) & ^{13}C NMR (101 MHz, $CDCl_3$):

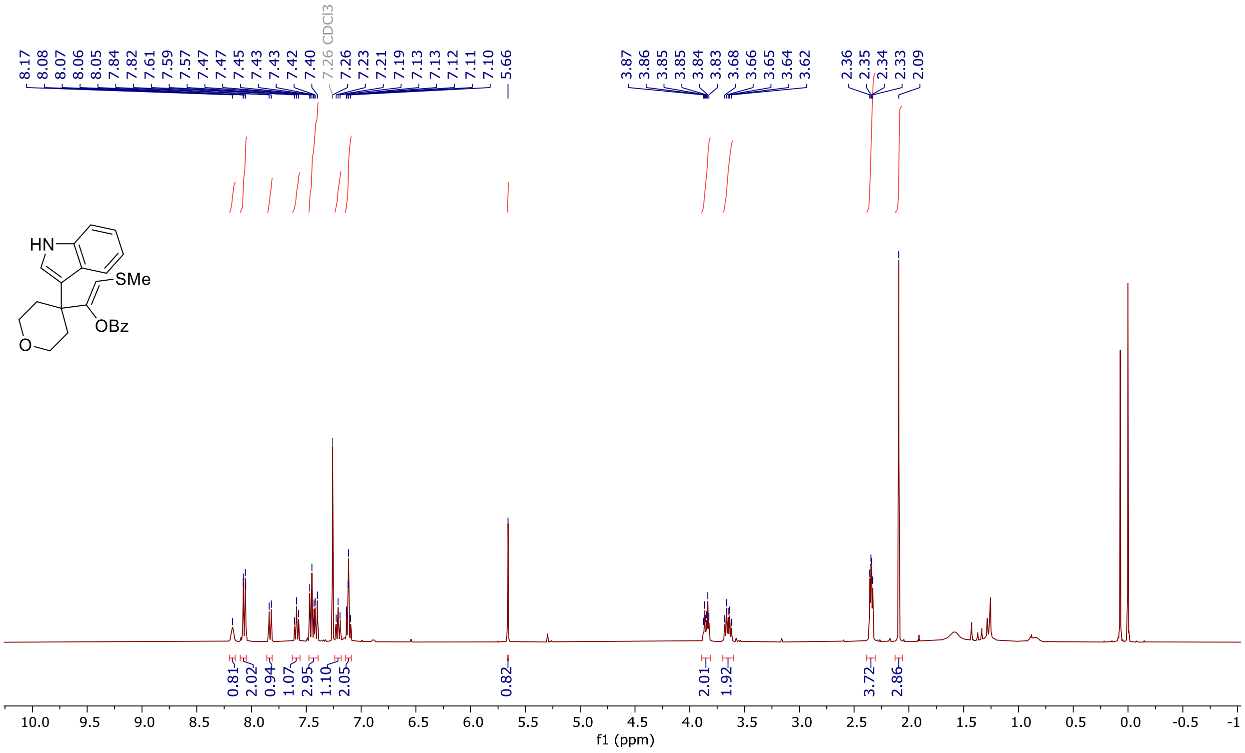

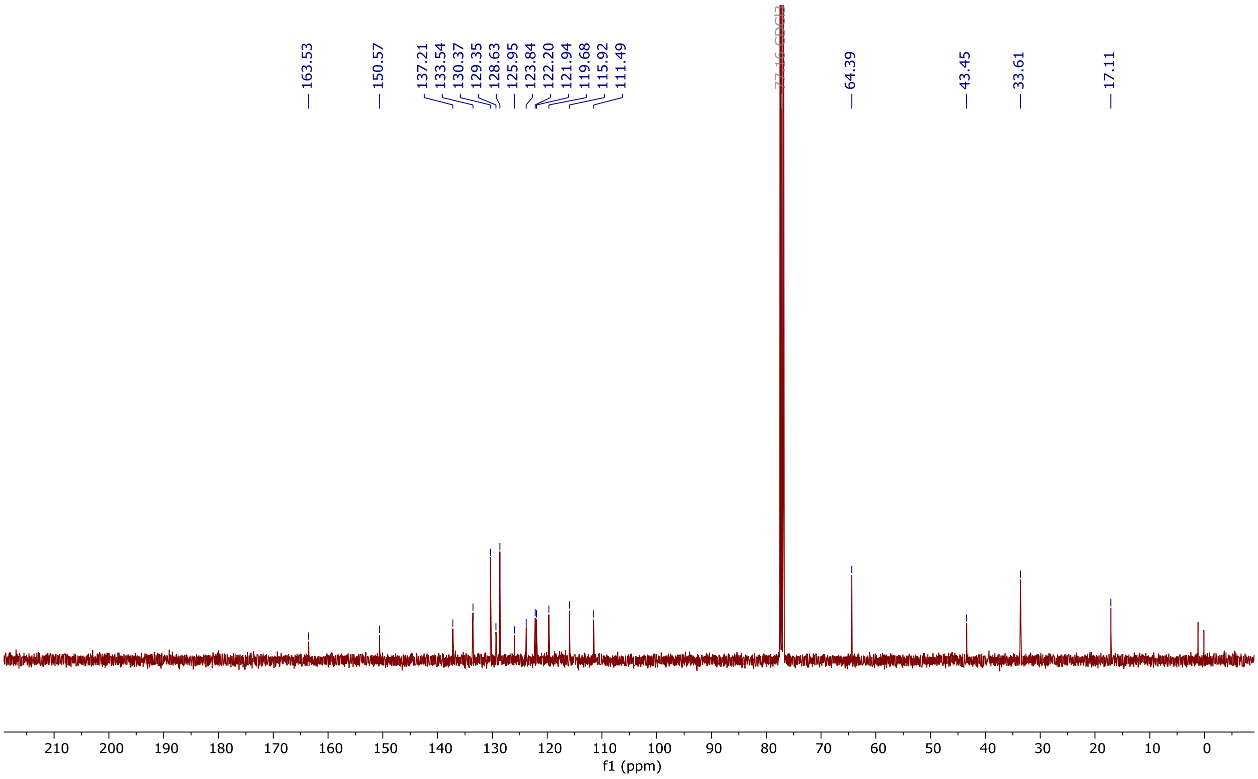

3ha ^{1}H NMR (400 MHz, $CDCl_3$) & ^{13}C NMR (101 MHz, $CDCl_3$):

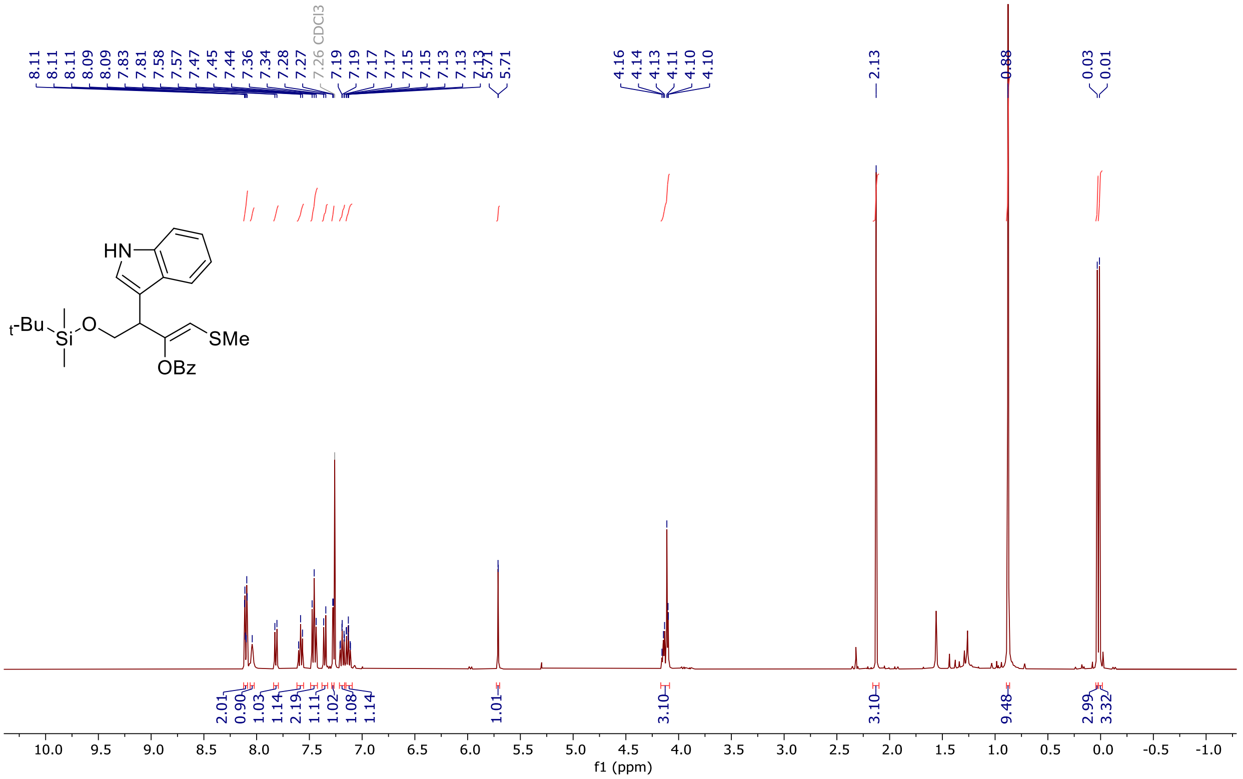

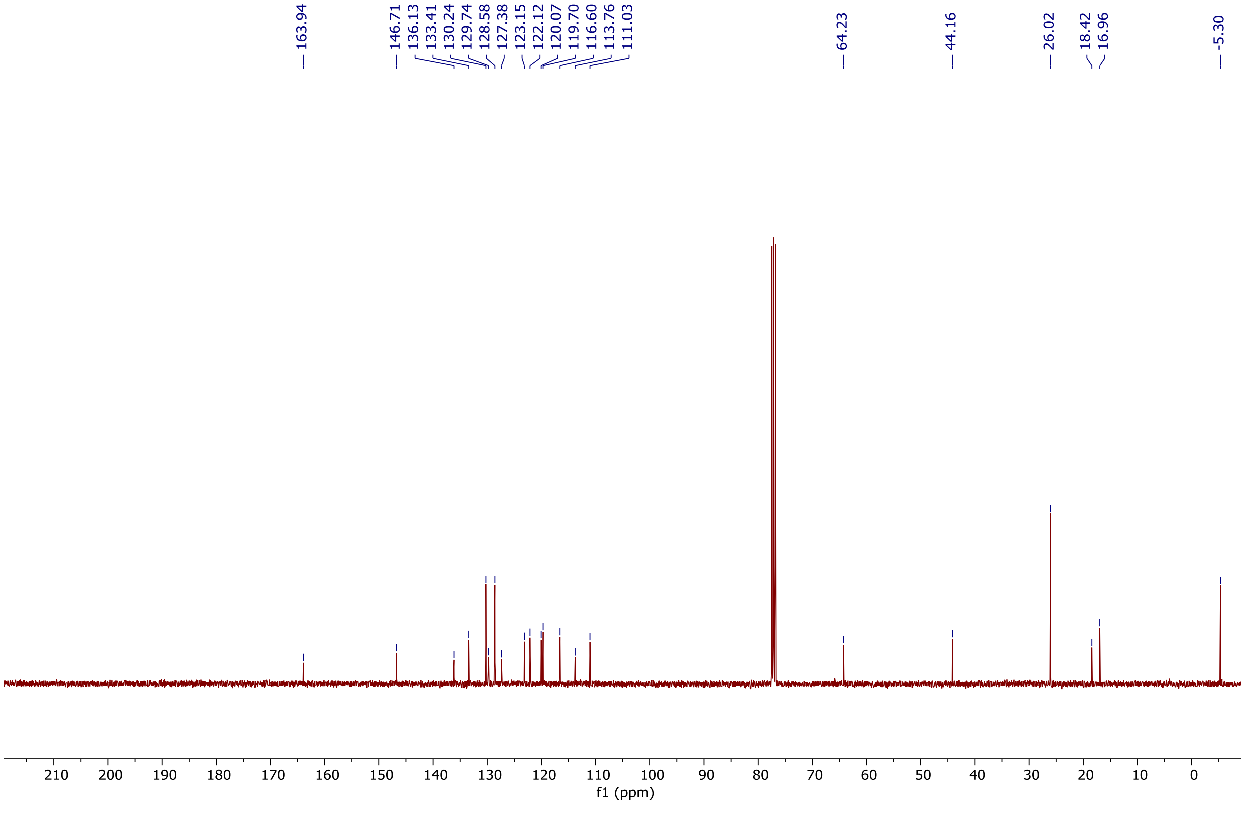

3ia ^{1}H NMR (400 MHz, $CDCl_3$) & ^{13}C NMR (101 MHz, $CDCl_3$):

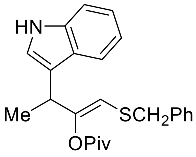

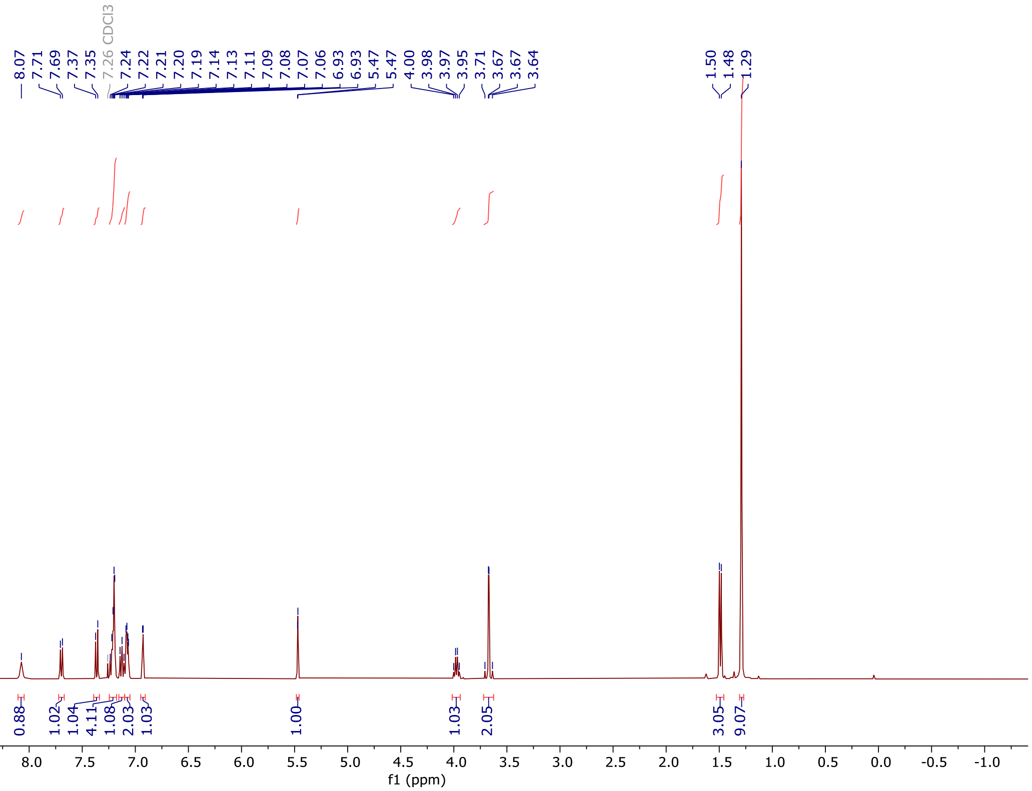

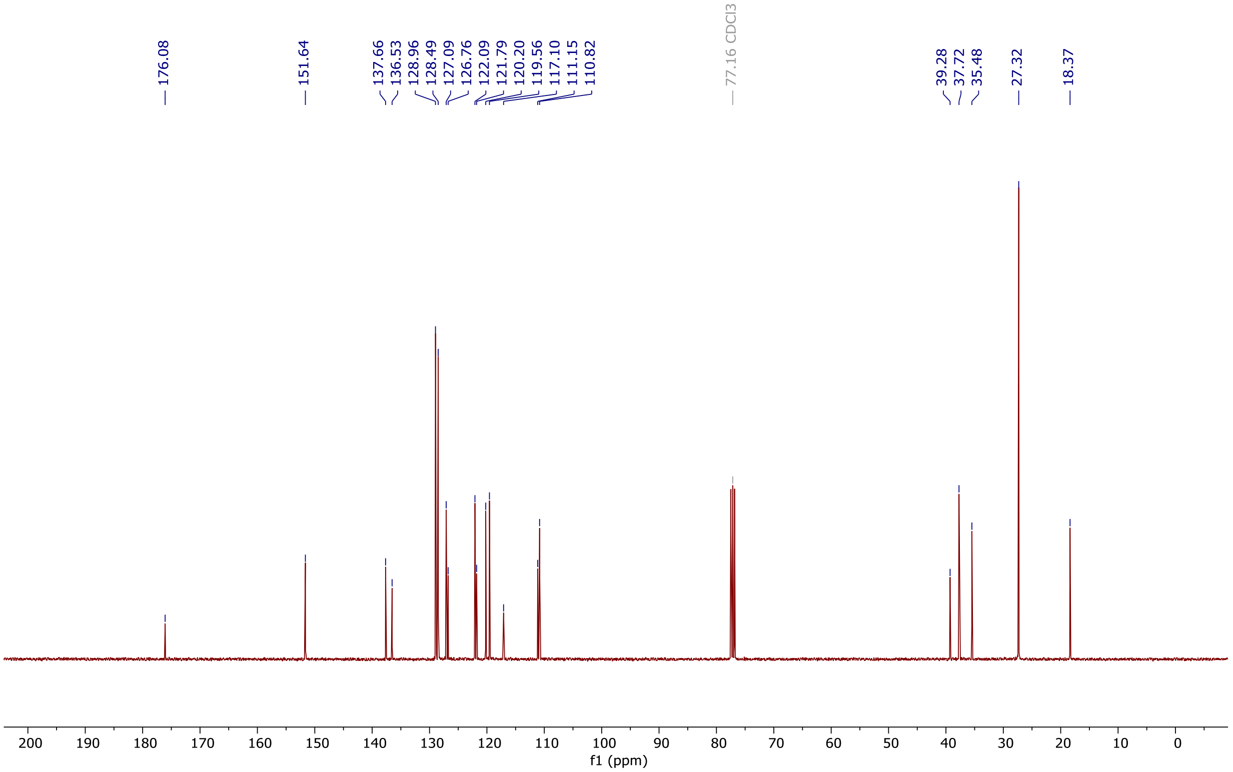

3ja ^{1}H NMR (400 MHz, $CDCl_3$) & ^{13}C NMR (101 MHz, $CDCl_3$):

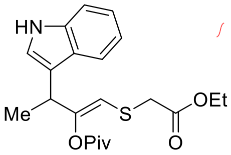

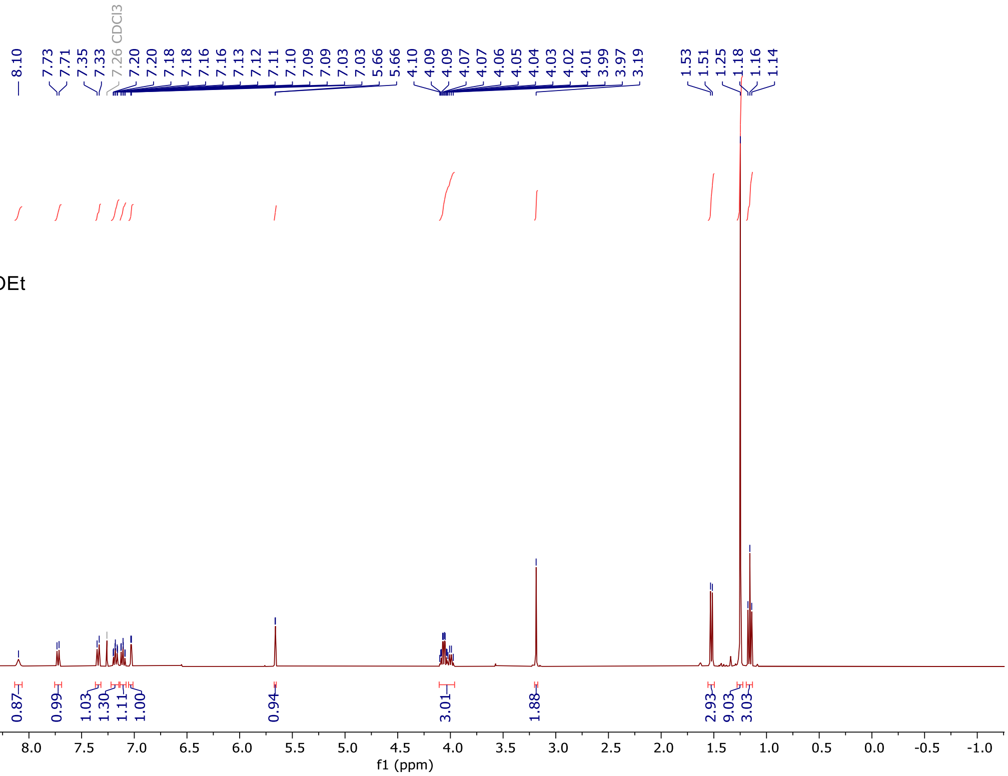

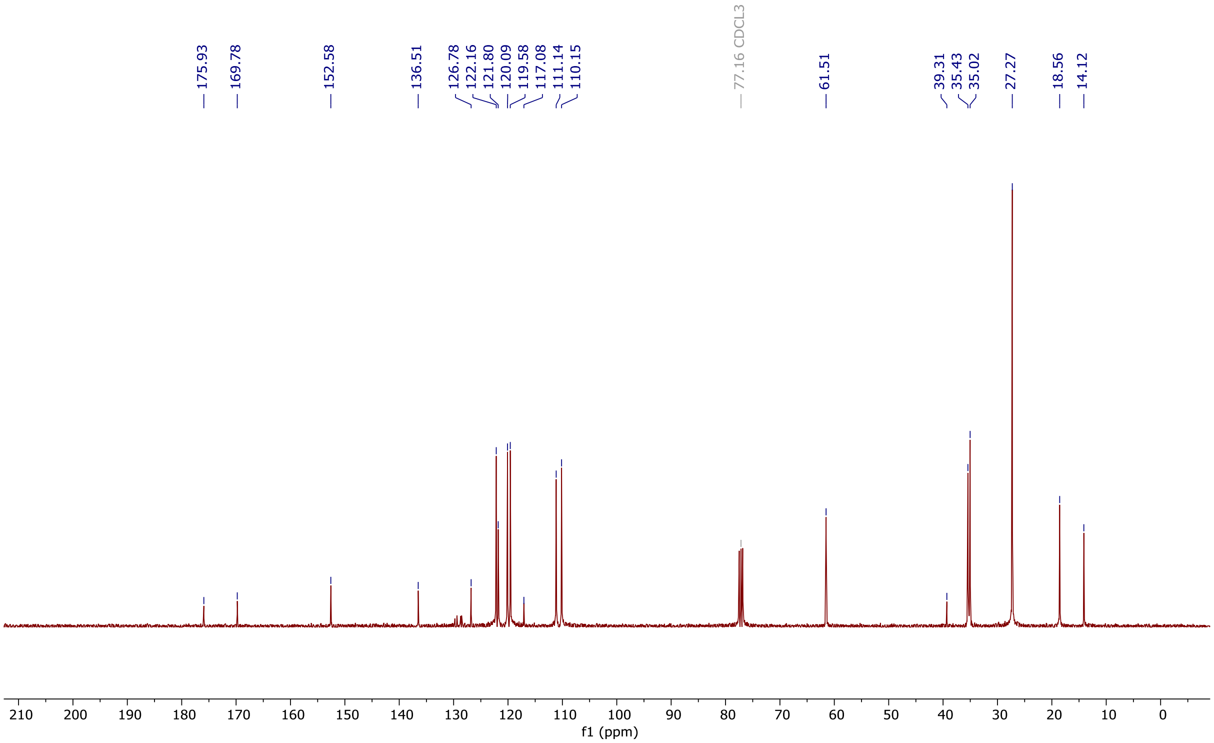

3ka ^{1}H NMR (400 MHz, $CDCl_3$) & ^{13}C NMR (101 MHz, $CDCl_3$):

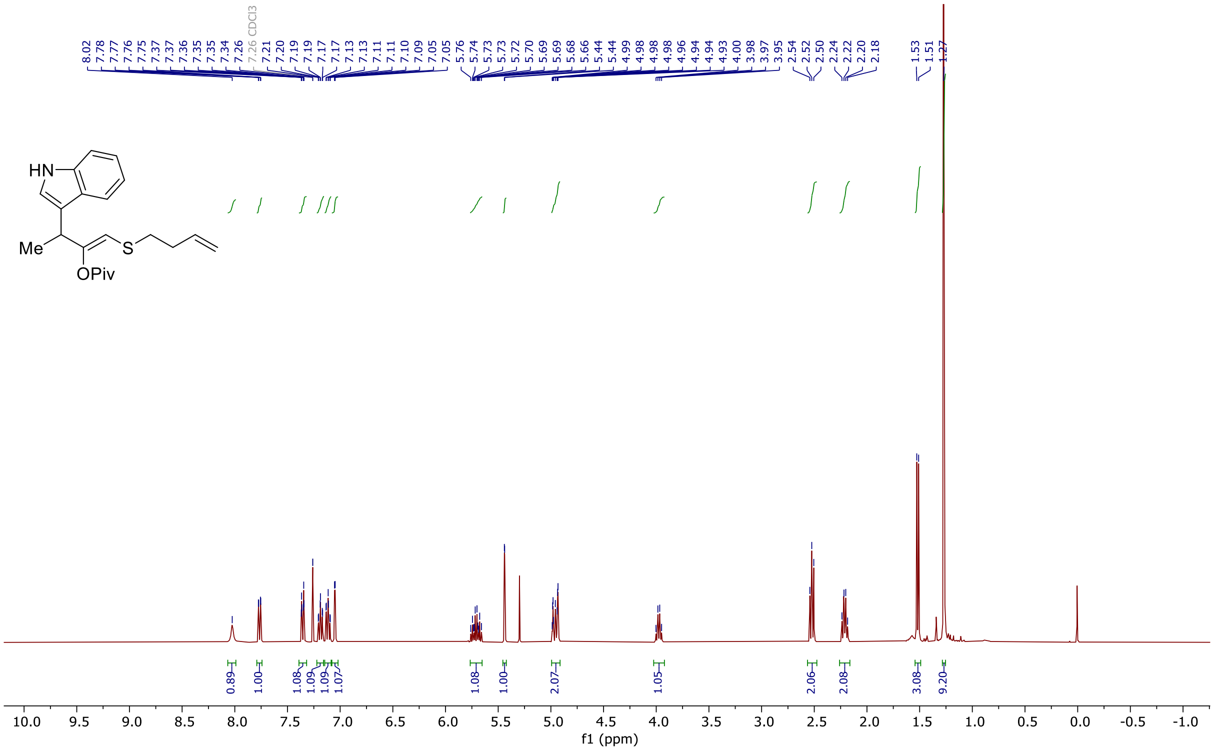

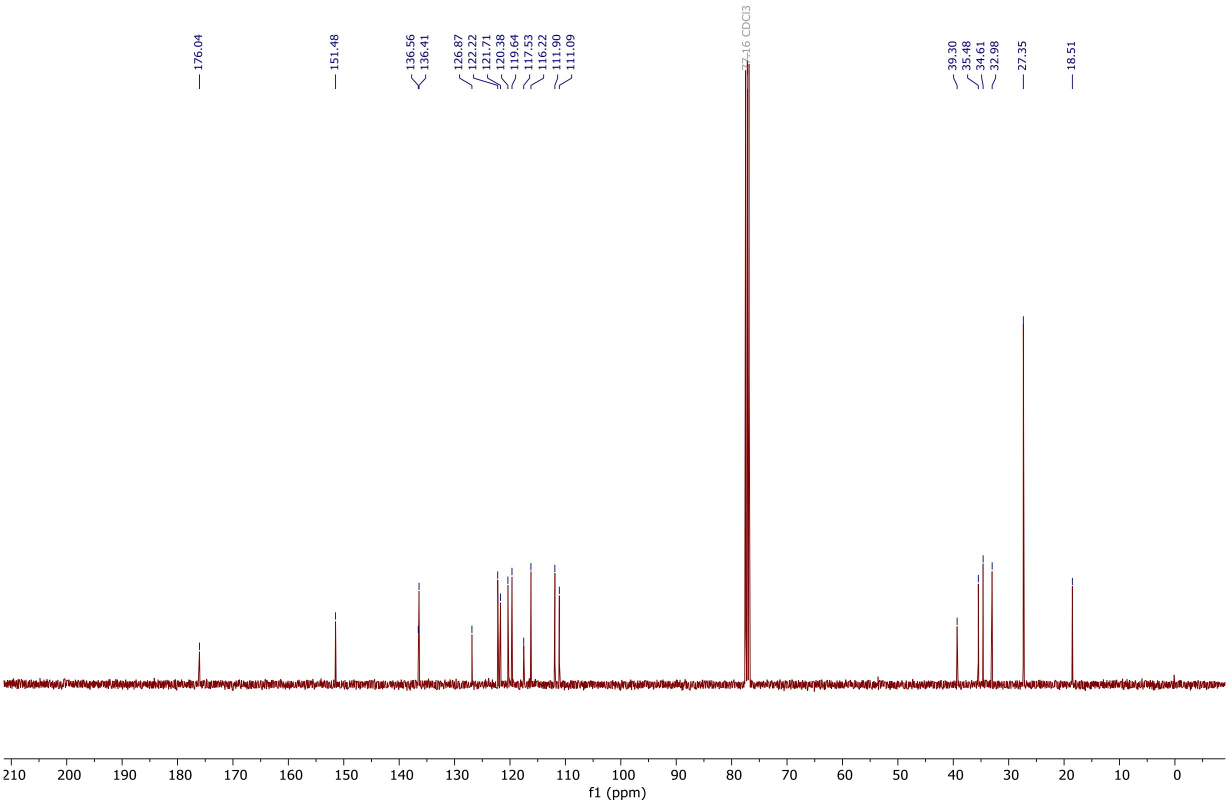

4aa ^{1}H NMR (400 MHz, $CDCl_3$) & ^{13}C NMR (101 MHz, $CDCl_3$):

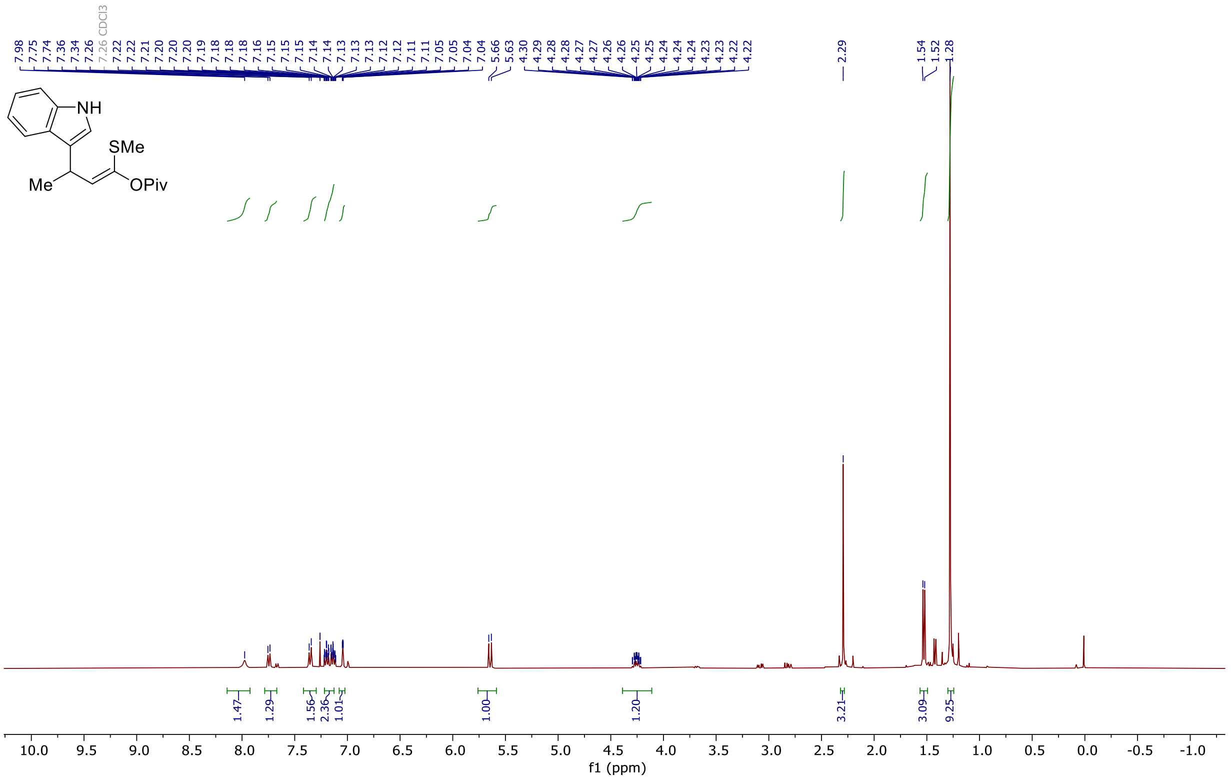

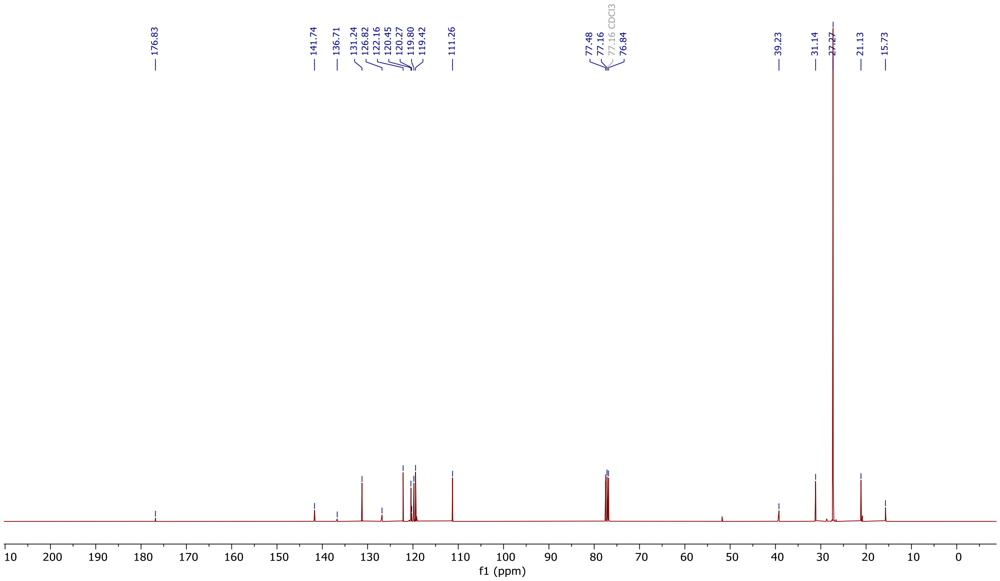

4la ^{1}H NMR (400 MHz, $CDCl_3$) & ^{13}C NMR (101 MHz, $CDCl_3$):

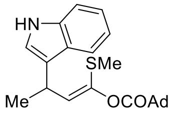

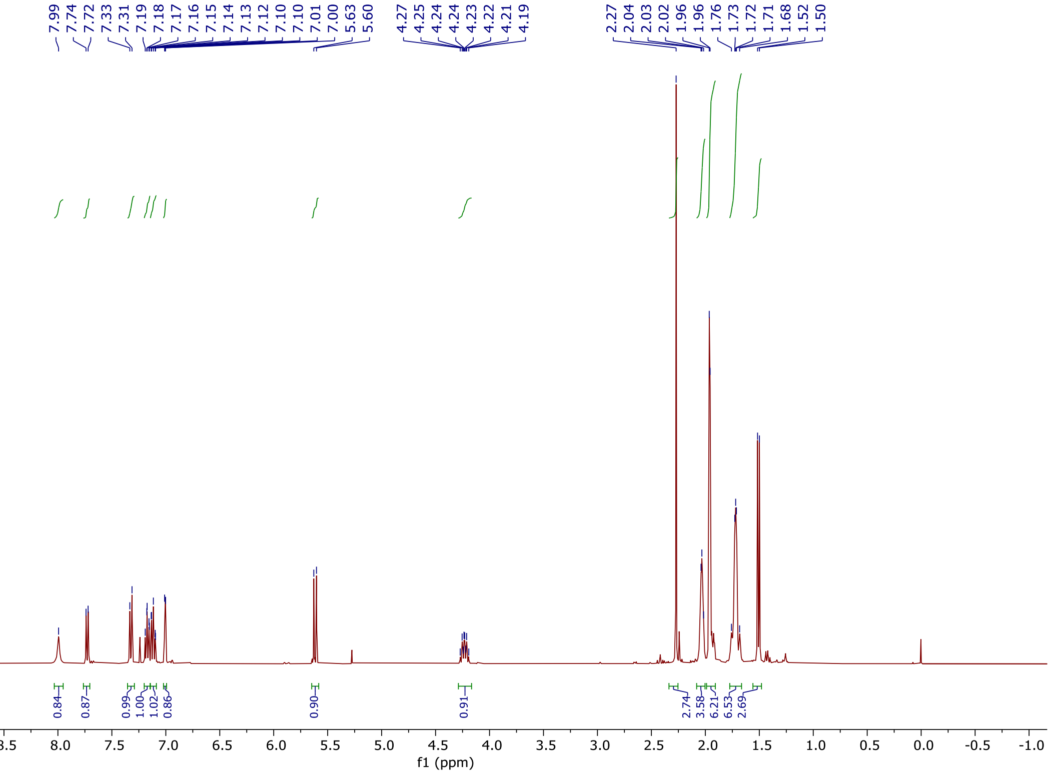

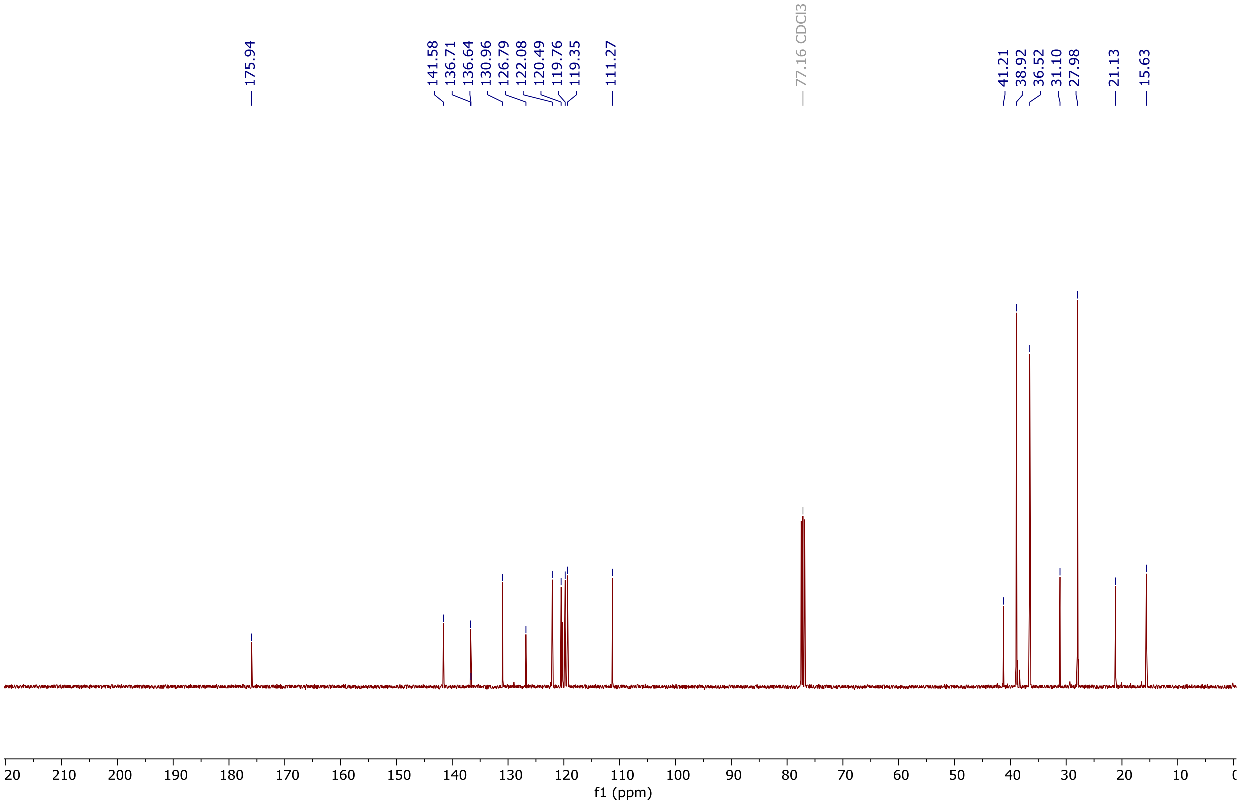

4lb ^{1}H NMR (400 MHz, $CDCl_3$) & ^{13}C NMR (101 MHz, $CDCl_3$):

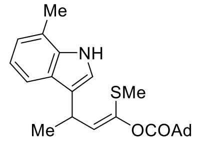

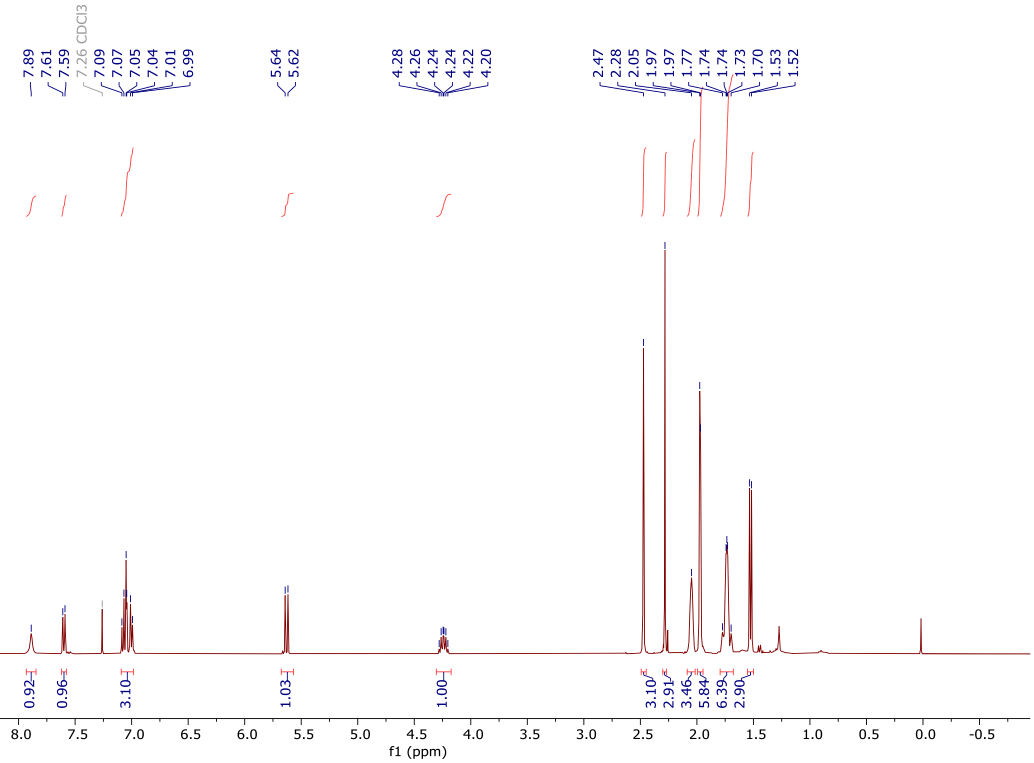

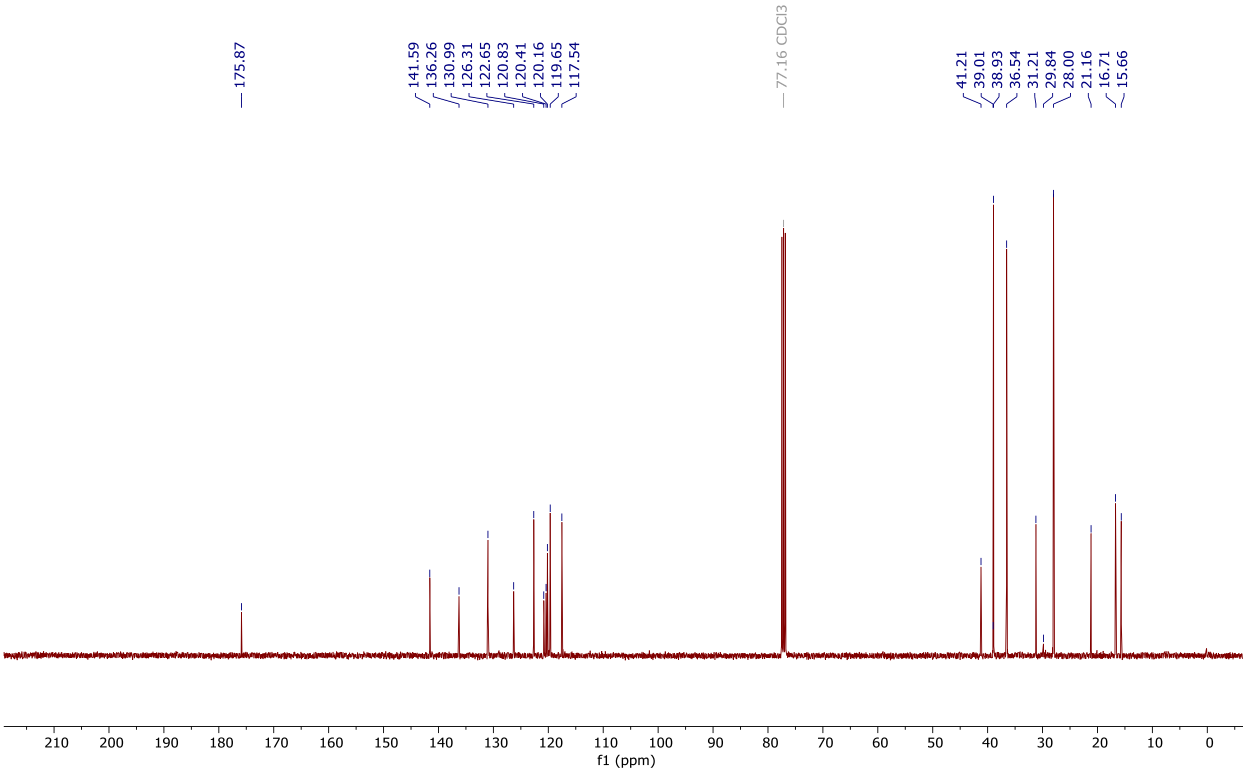

4lf ^{1}H NMR (400 MHz, $CDCl_3$) & ^{13}C NMR (101 MHz, $CDCl_3$):

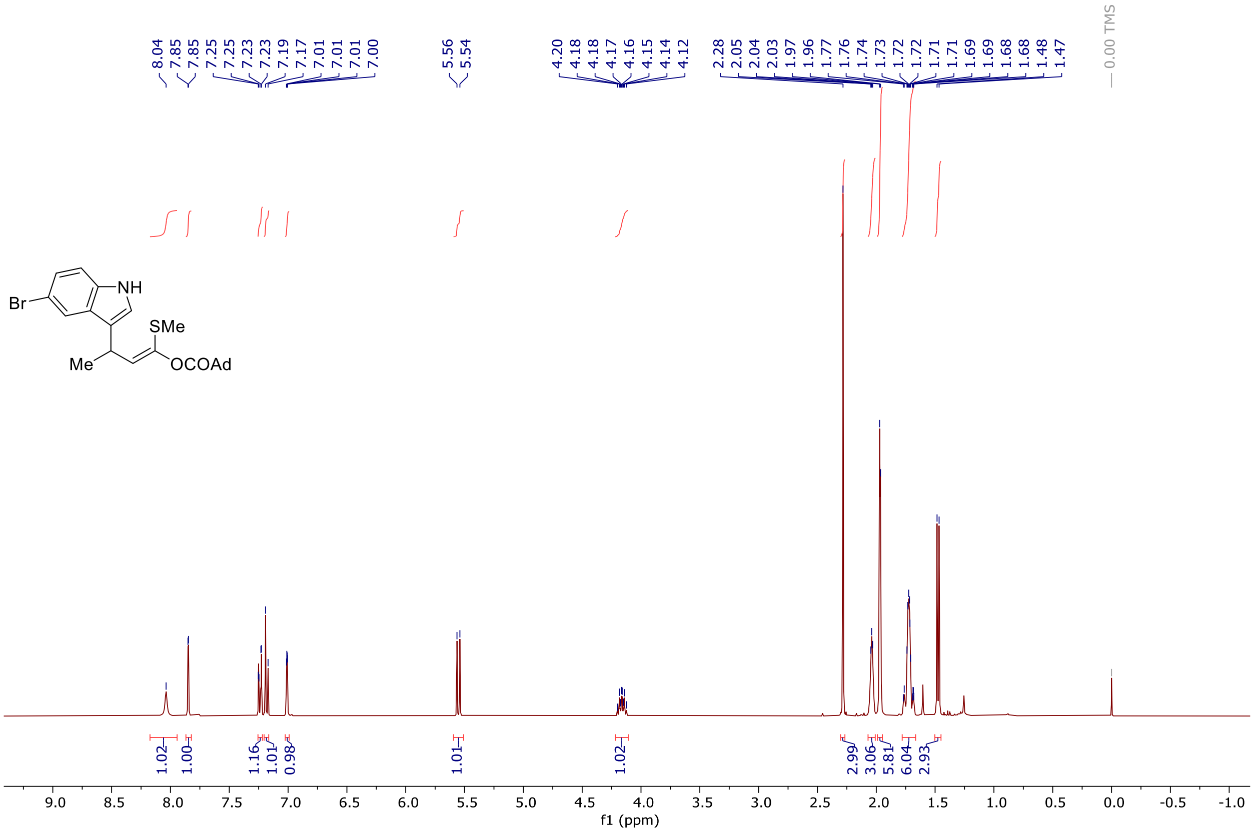

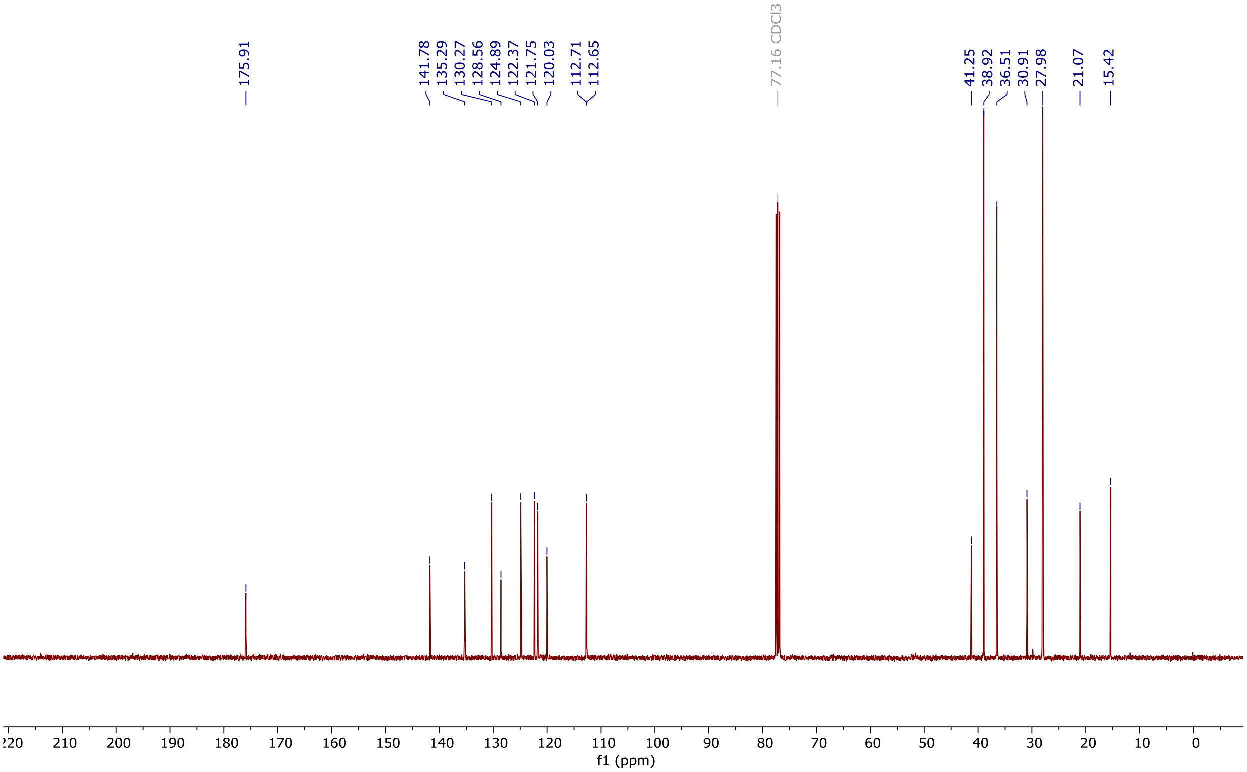

4mb ^{1}H NMR (400 MHz, $CDCl_3$) & ^{13}C NMR (101 MHz, $CDCl_3$):

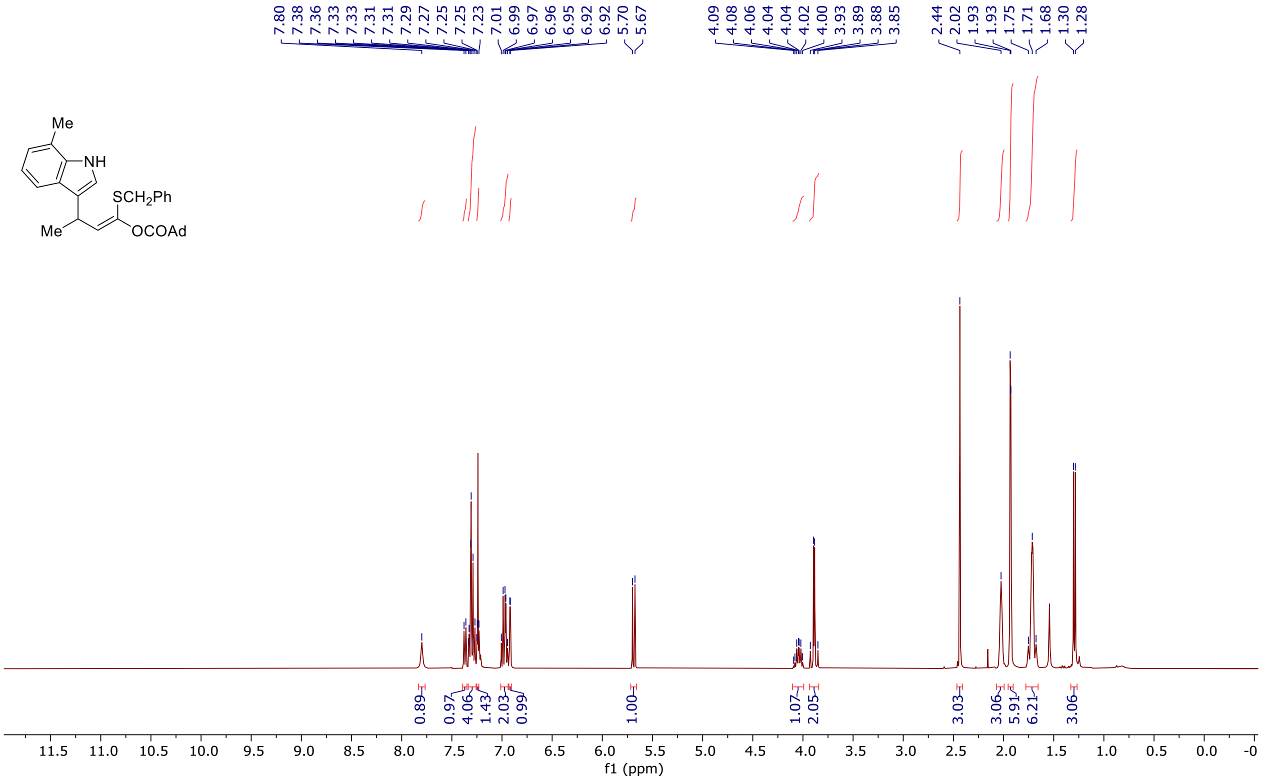

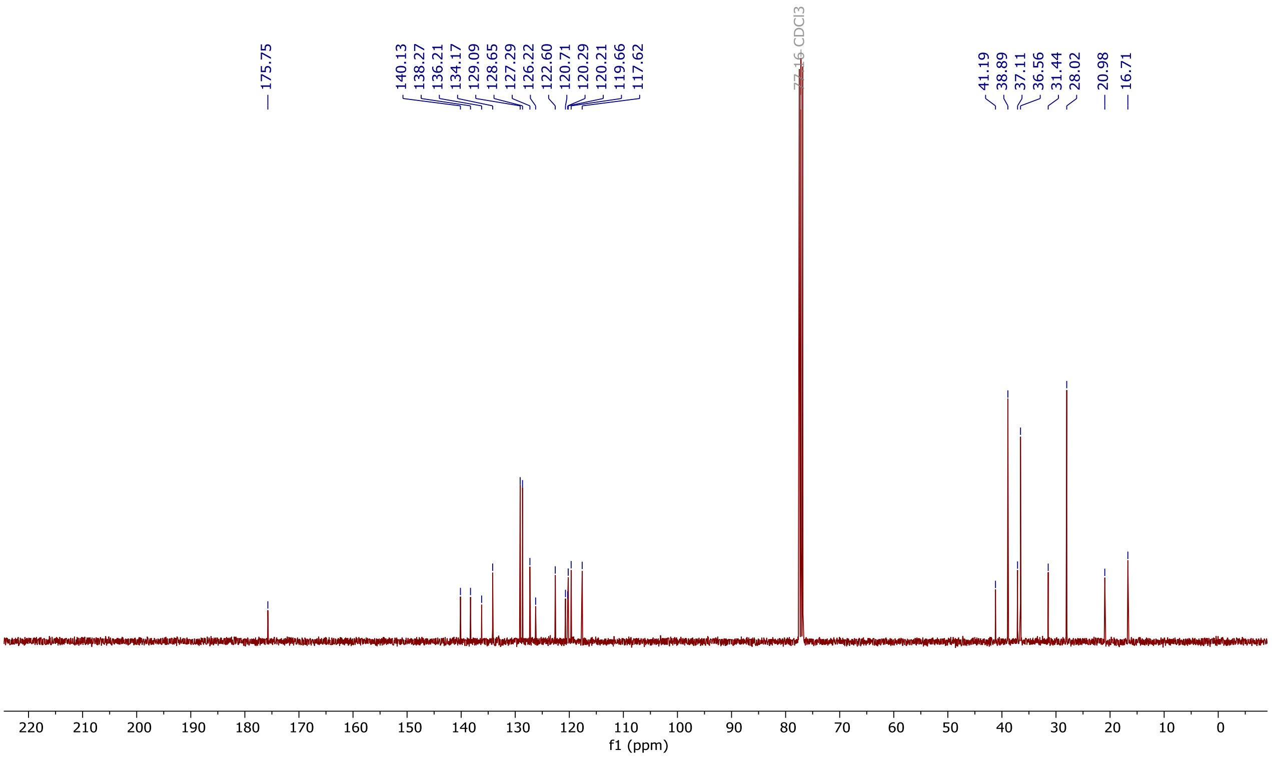

4nb ^{1}H NMR (400 MHz, $CDCl_3$) & ^{13}C NMR (101 MHz, $CDCl_3$):

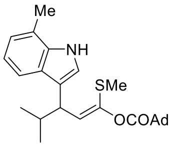

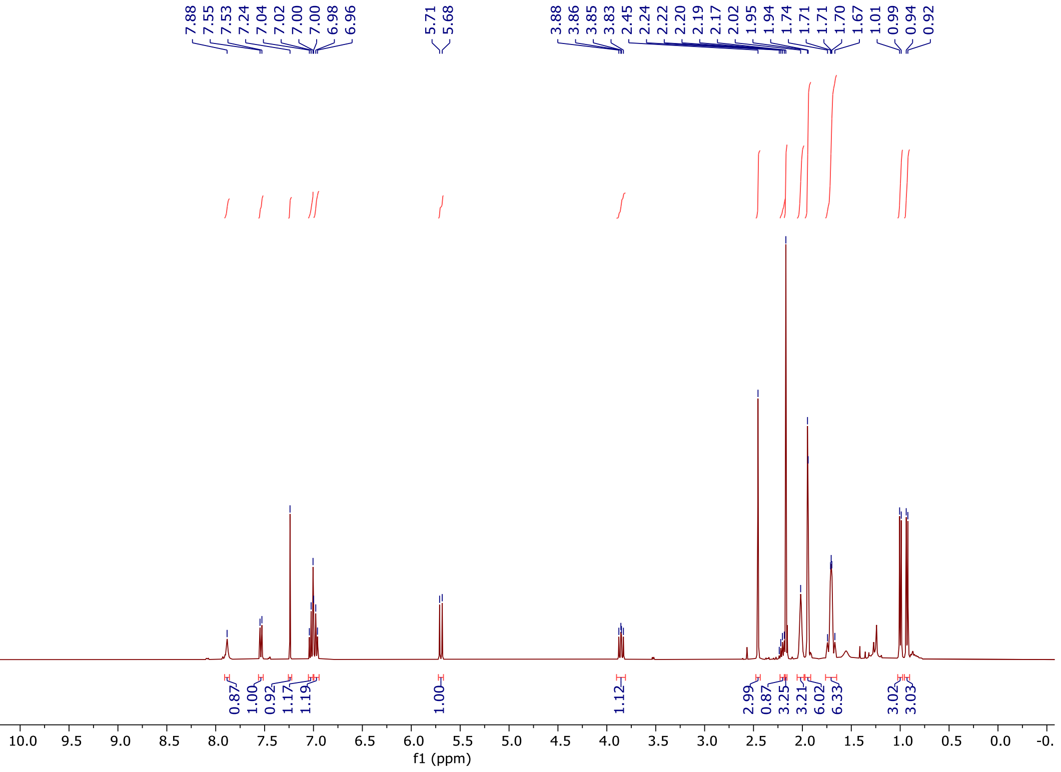

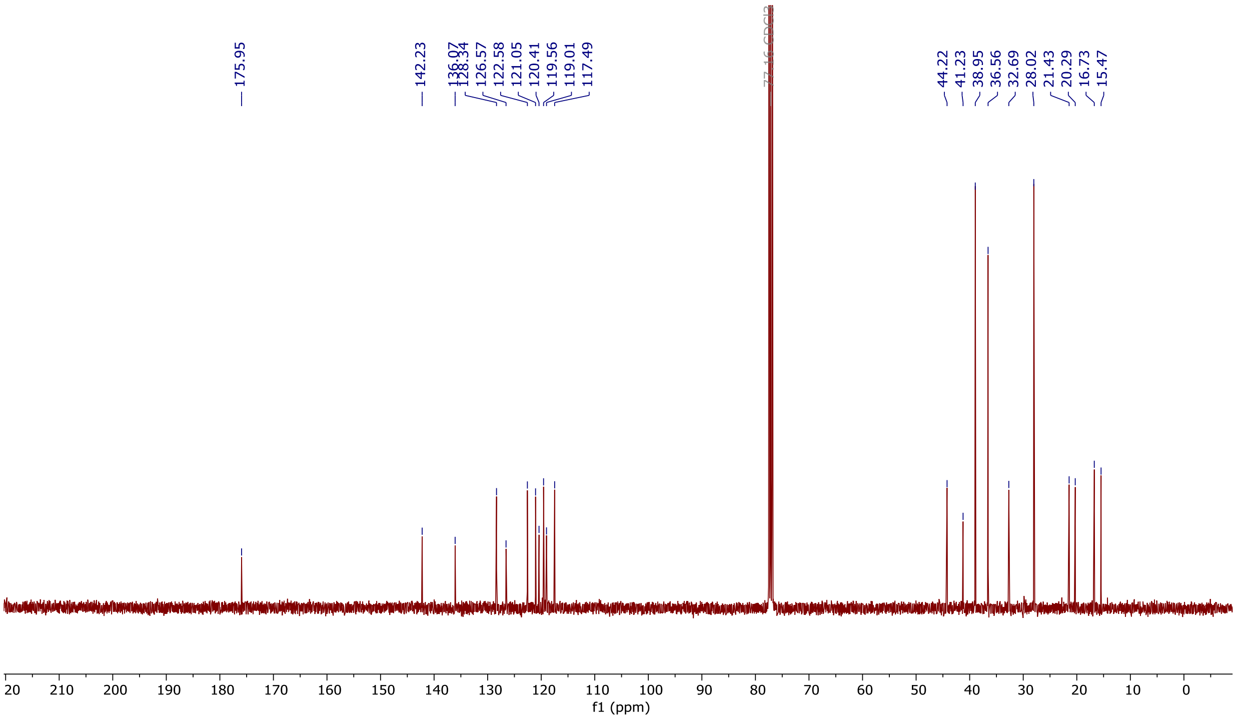

4ob ^{1}H NMR (400 MHz, $CDCl_3$) & ^{13}C NMR (101 MHz, $CDCl_3$):

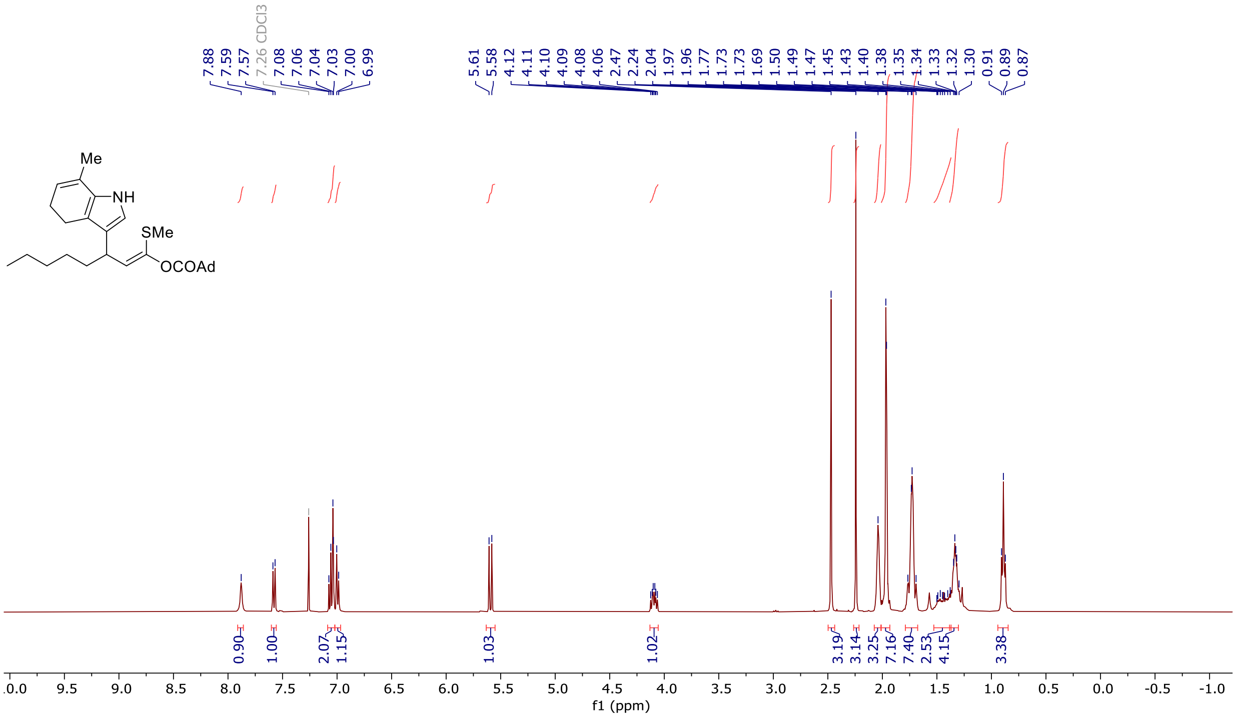

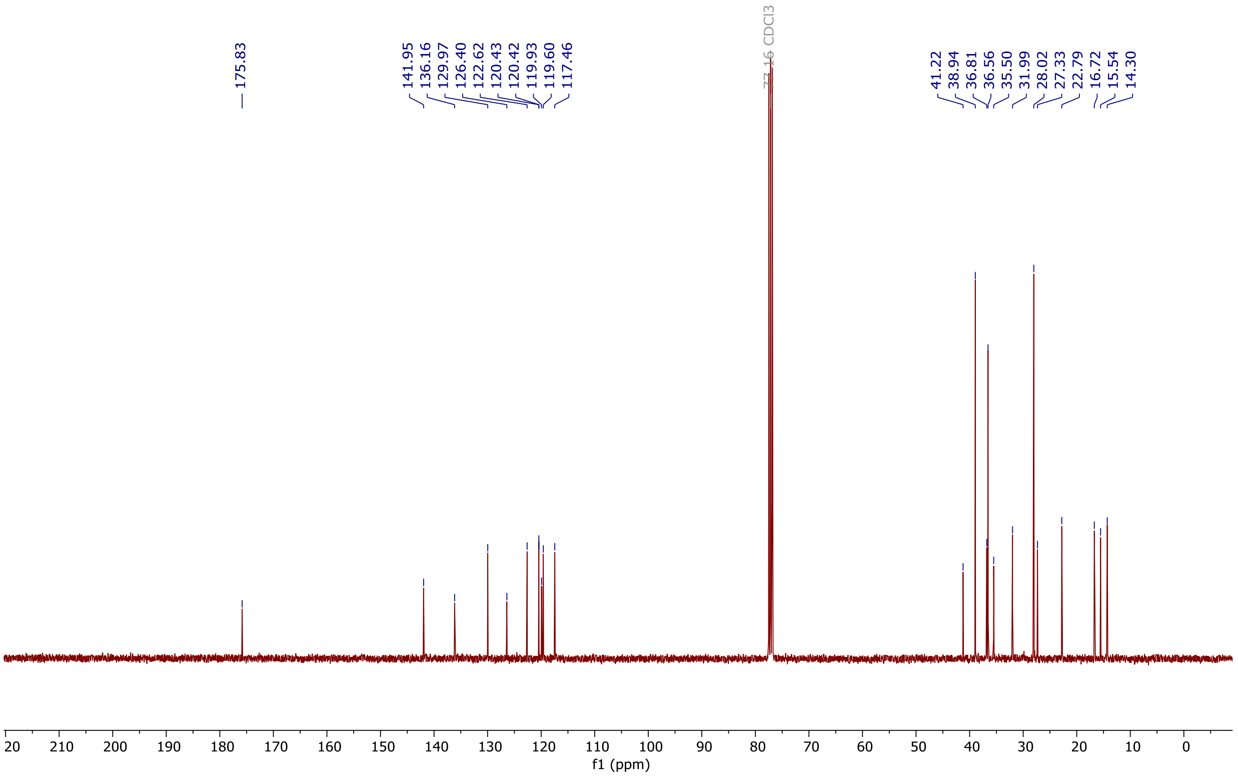

5 ^{1}H NMR (400 MHz, $CDCl_3$) & ^{13}C NMR (101 MHz, $CDCl_3$):

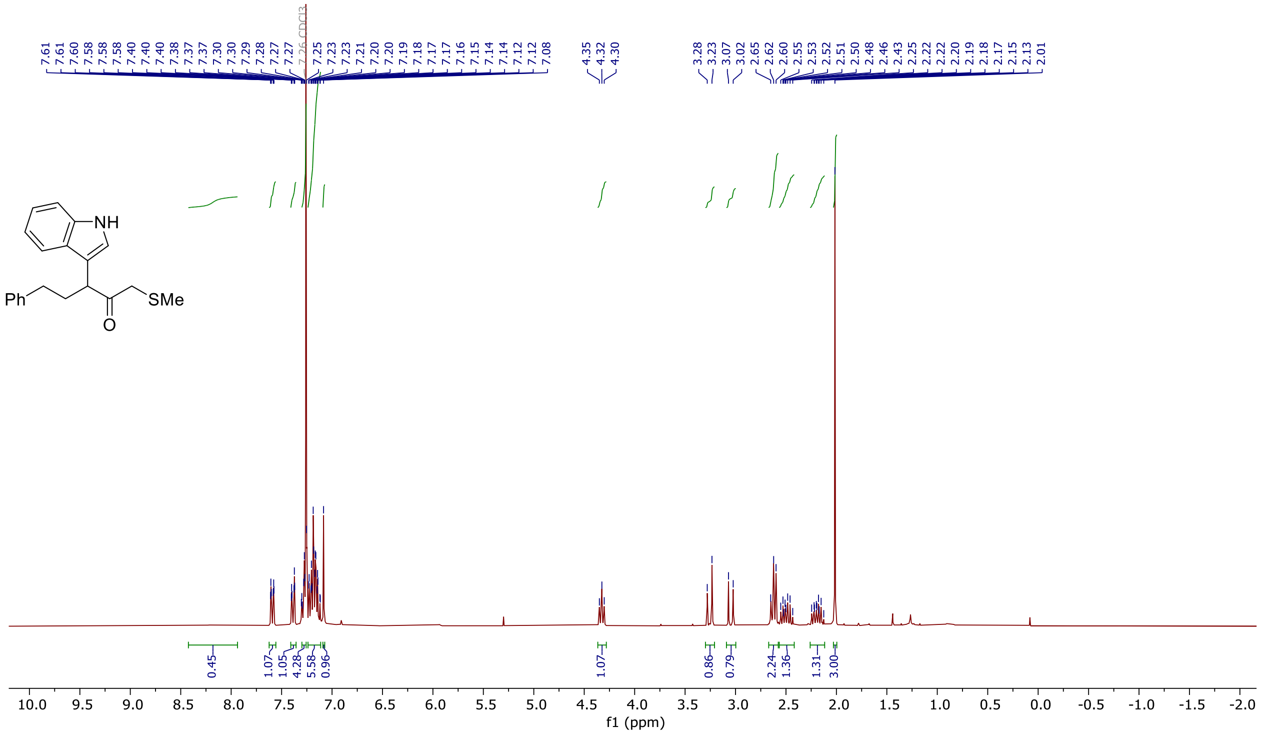

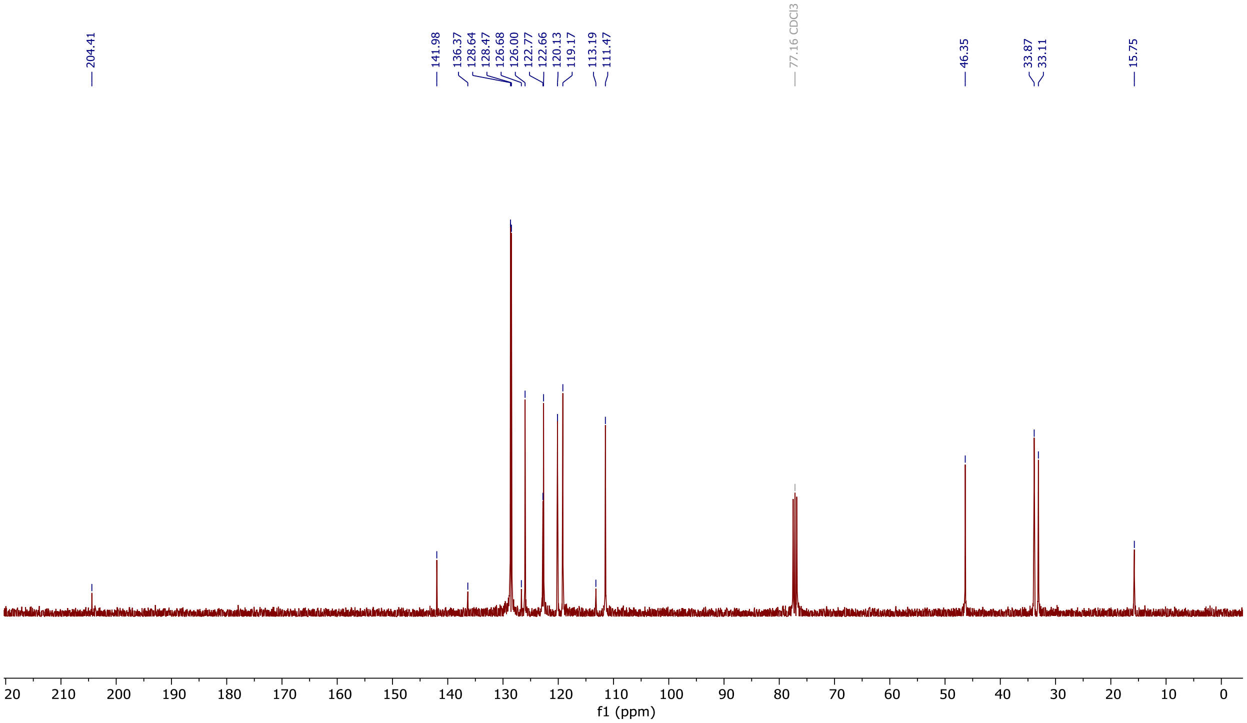

6 ^{1}H NMR (400 MHz, $CDCl_3$) & ^{13}C NMR (101 MHz, $CDCl_3$):

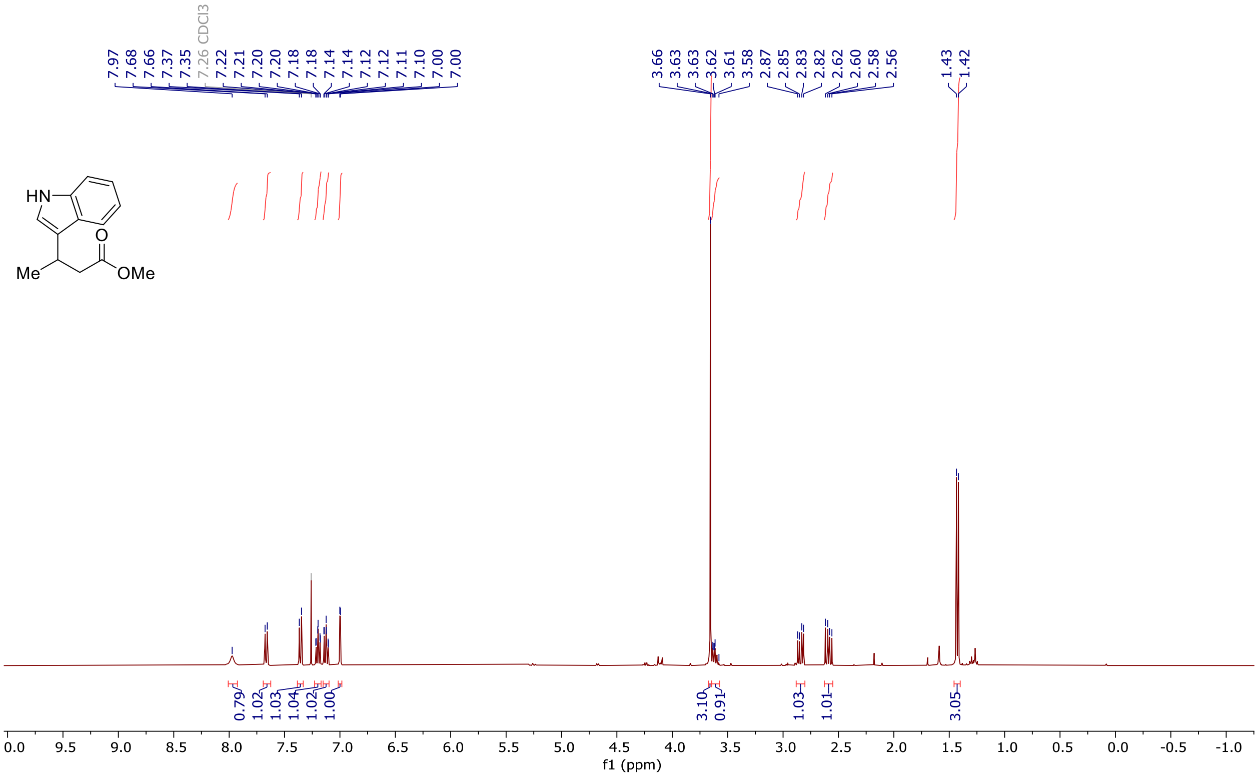

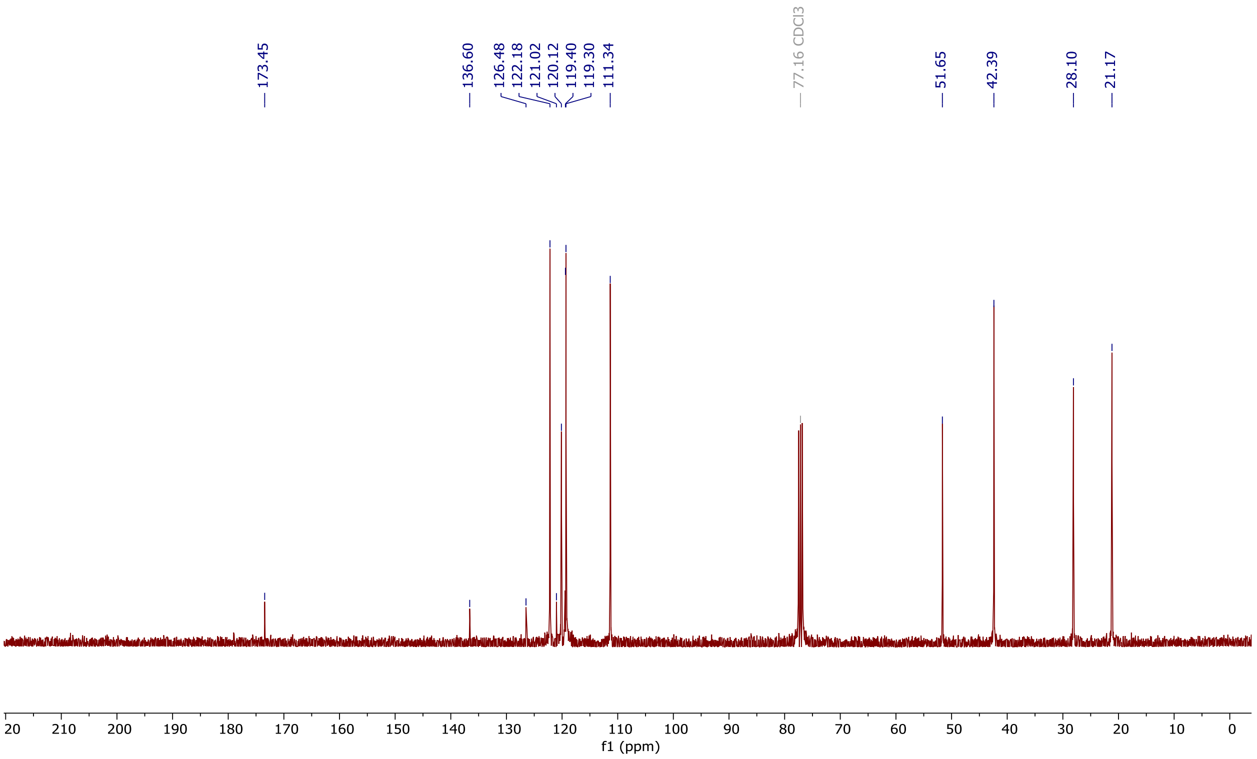

7aa ^{1}H NMR (400 MHz, $CDCl_3$) & ^{13}C NMR (101 MHz, $CDCl_3$):

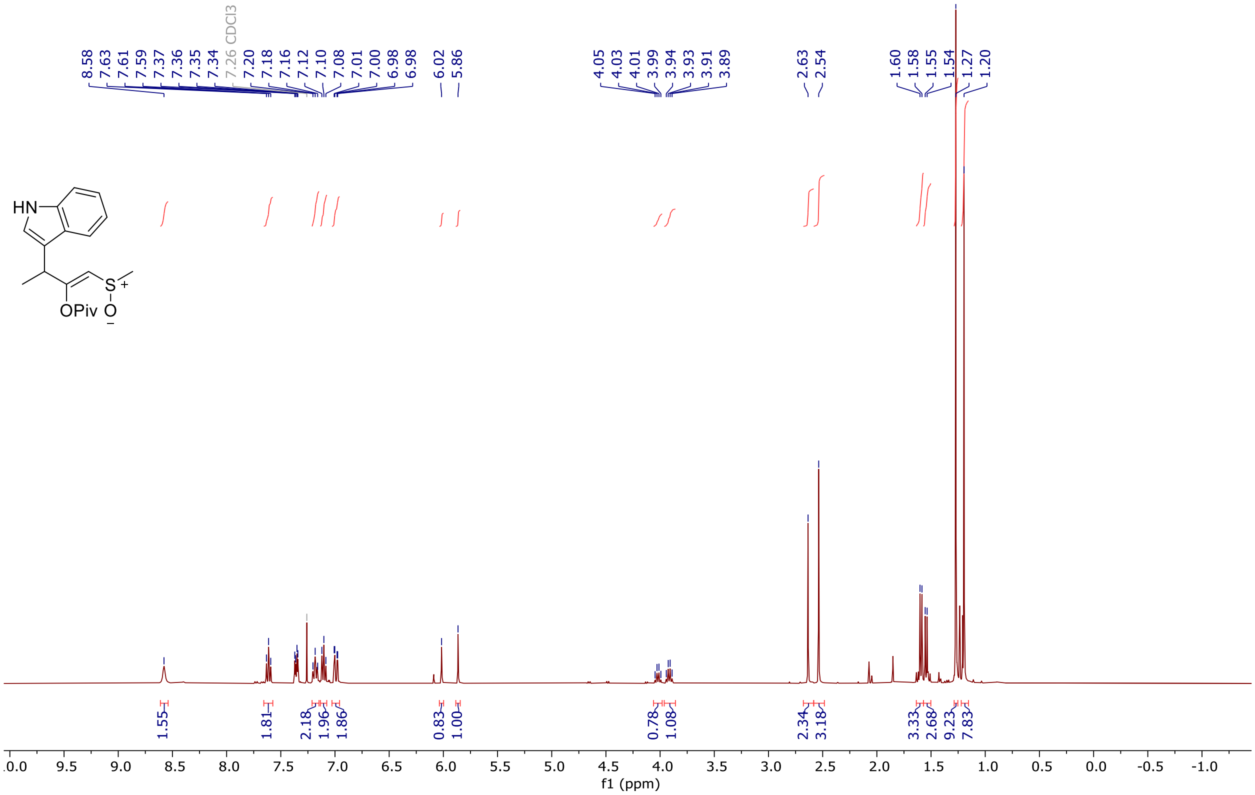

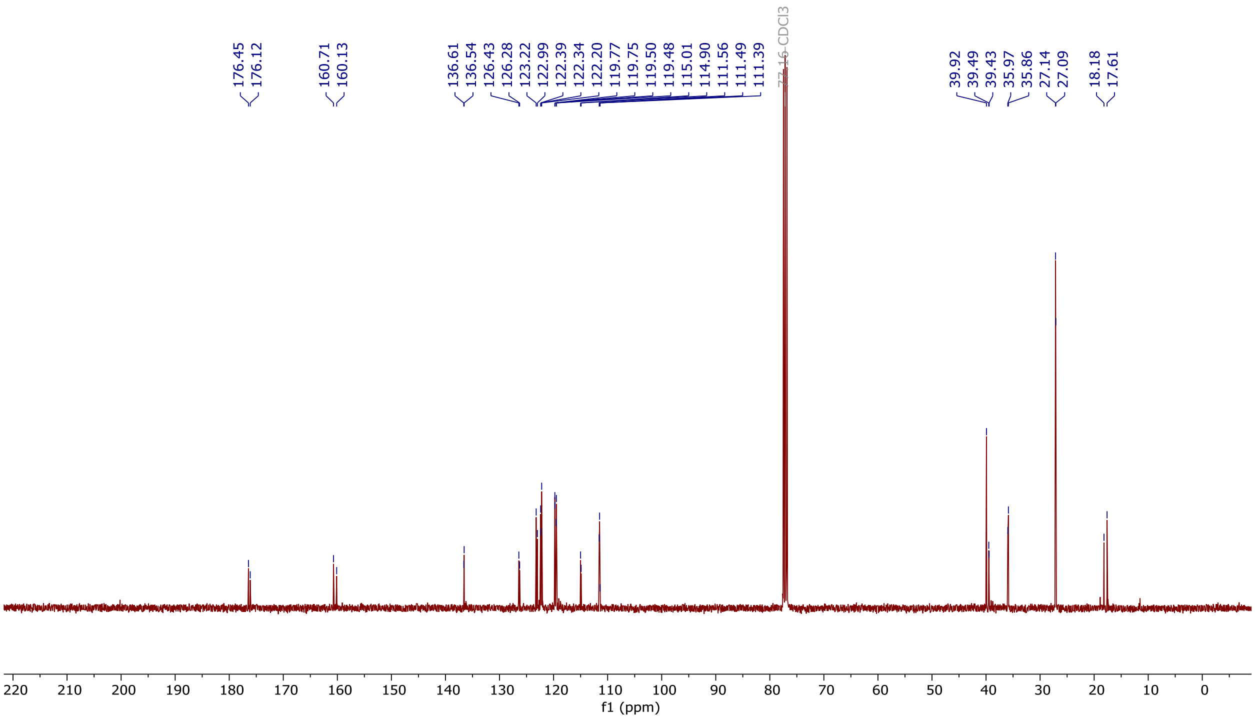

8aa ^{1}H NMR (400 MHz, $CDCl_3$) & ^{13}C NMR (101 MHz, $CDCl_3$):

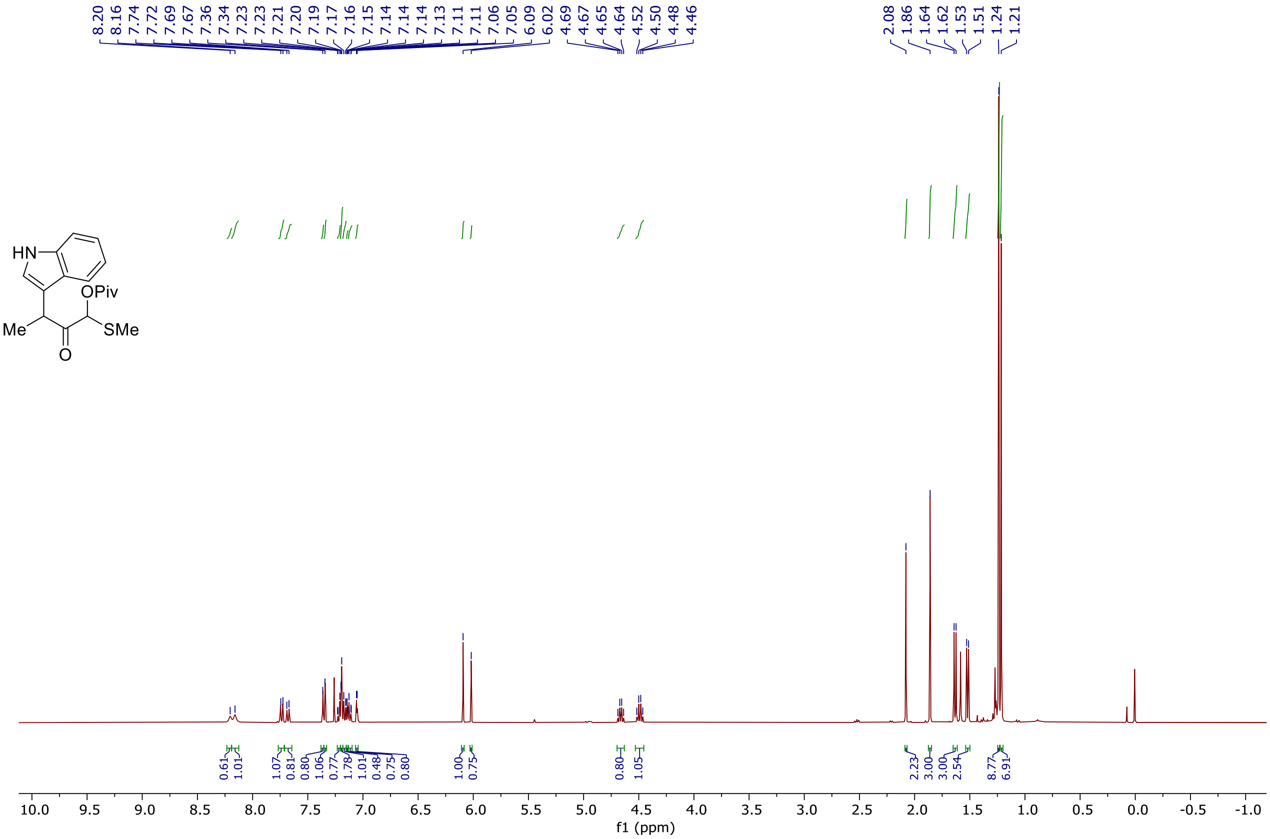

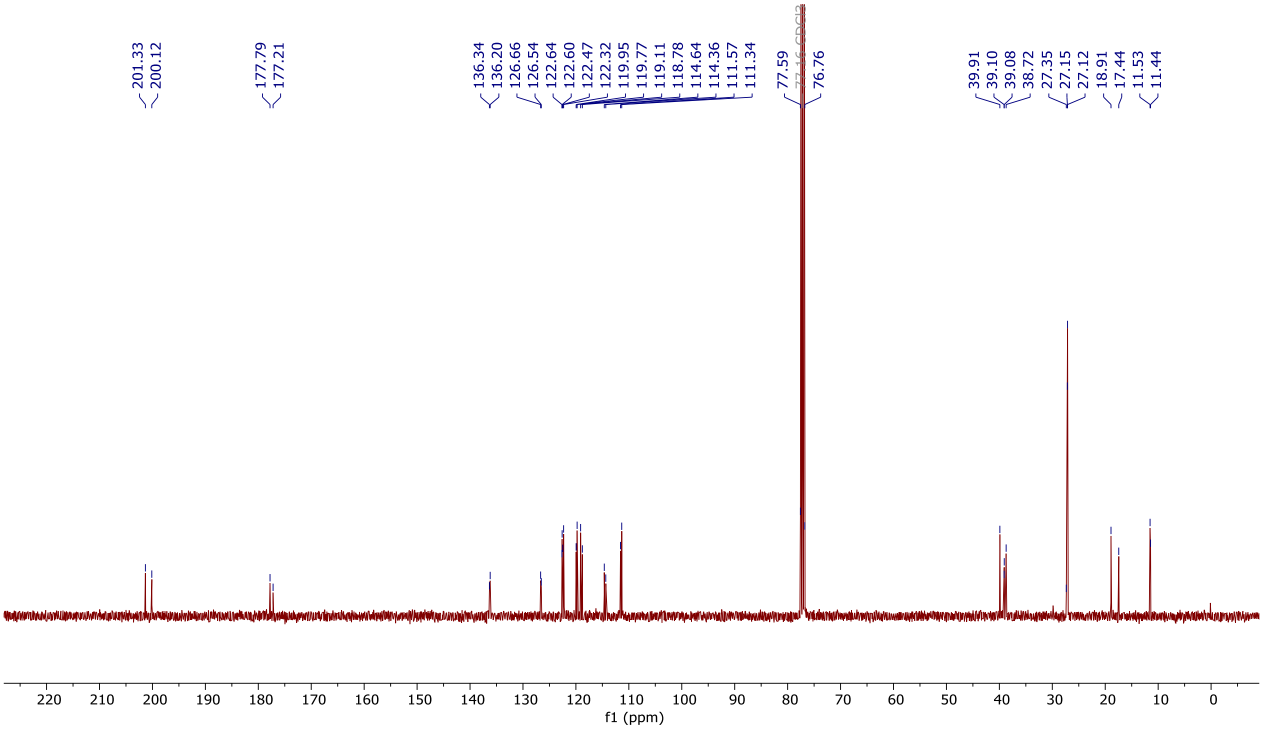

8ad ^{1}H NMR (400 MHz, $CDCl_3$) & ^{13}C NMR (101 MHz, $CDCl_3$):

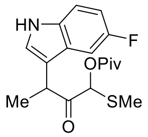

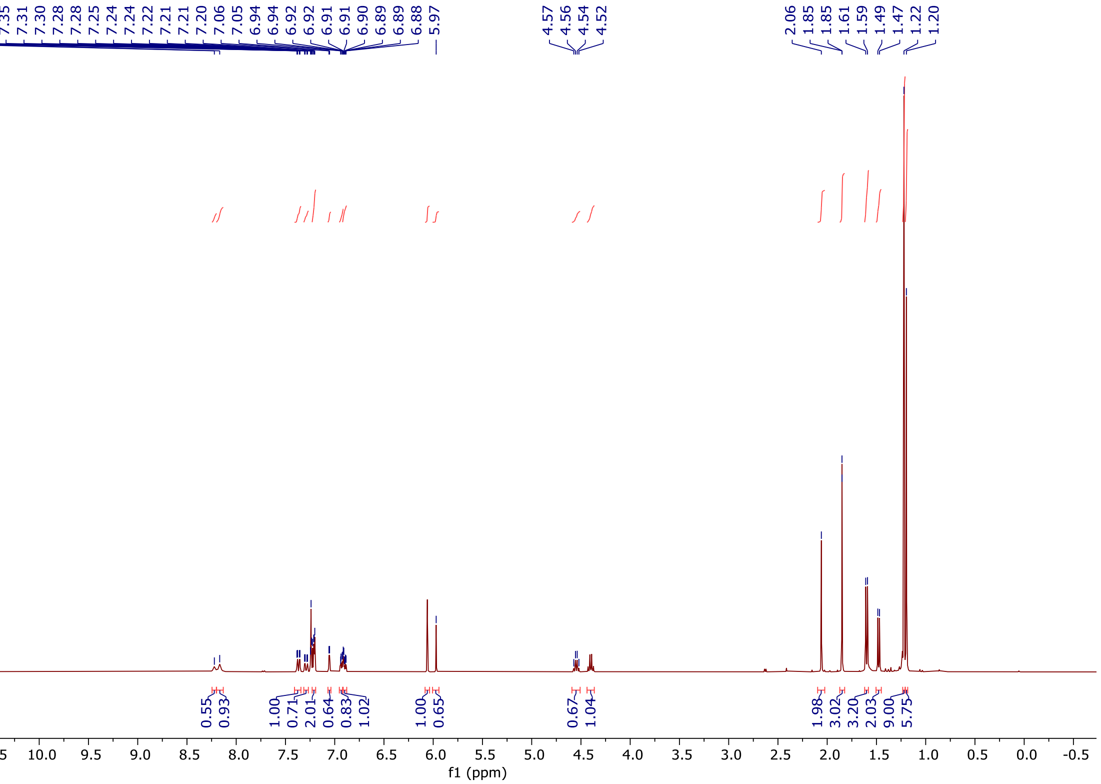

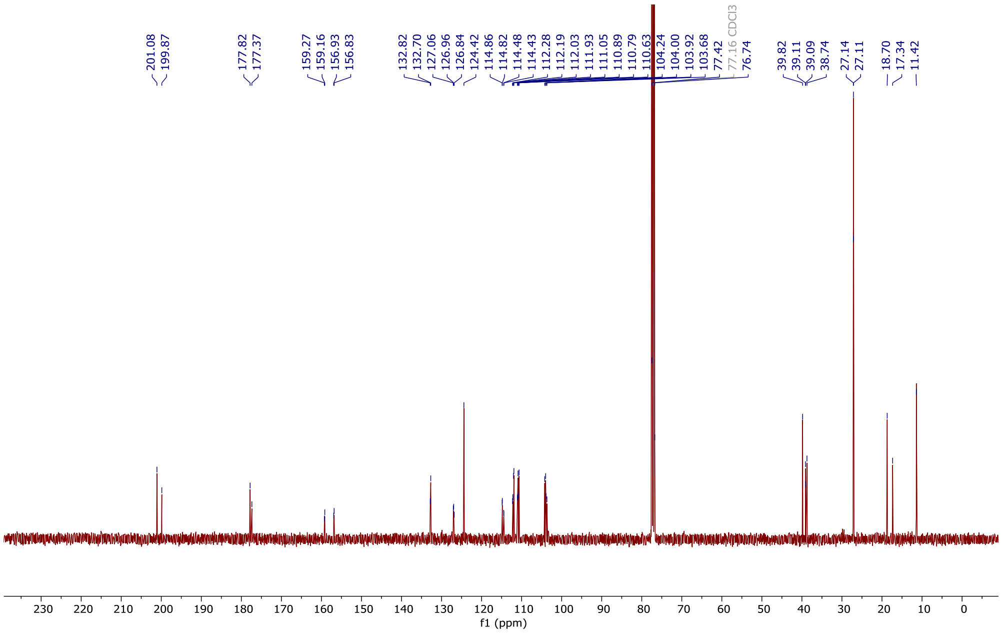

8da ^{1}H NMR (400 MHz, $CDCl_3$) & ^{13}C NMR (101 MHz, $CDCl_3$):

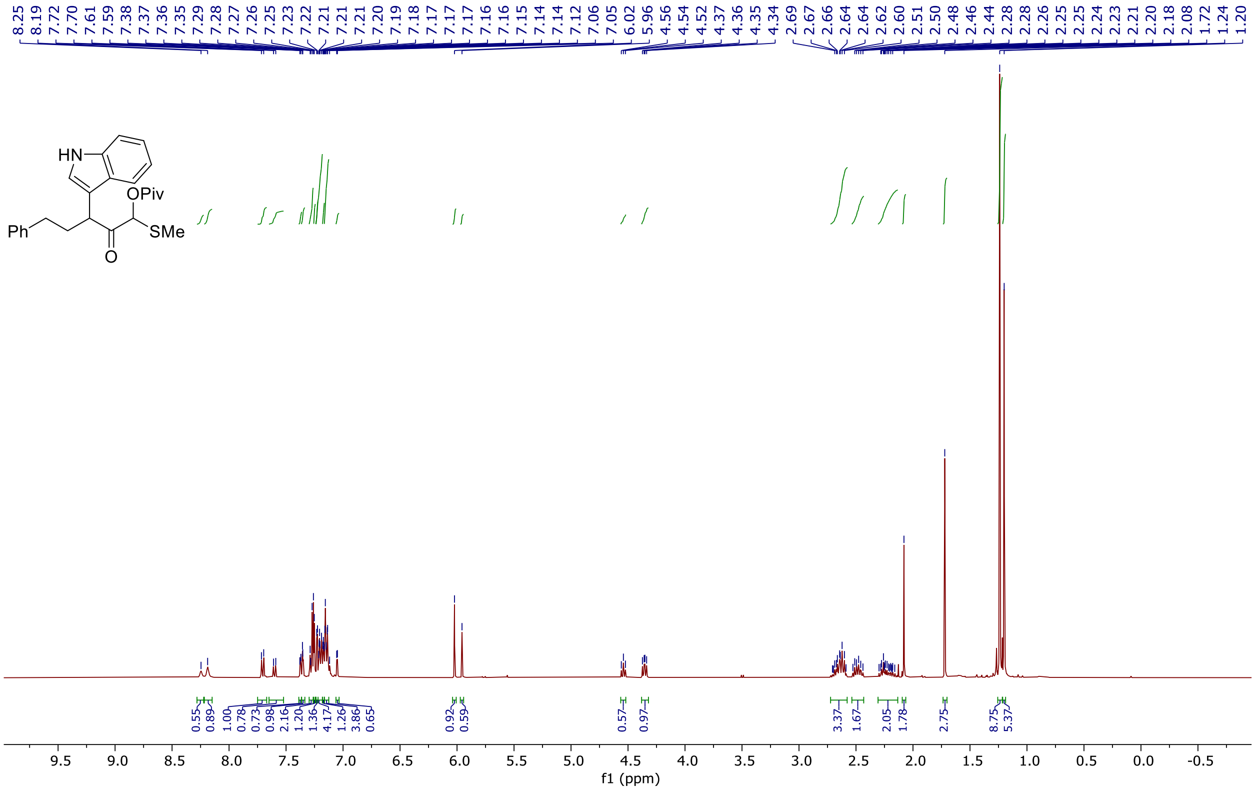

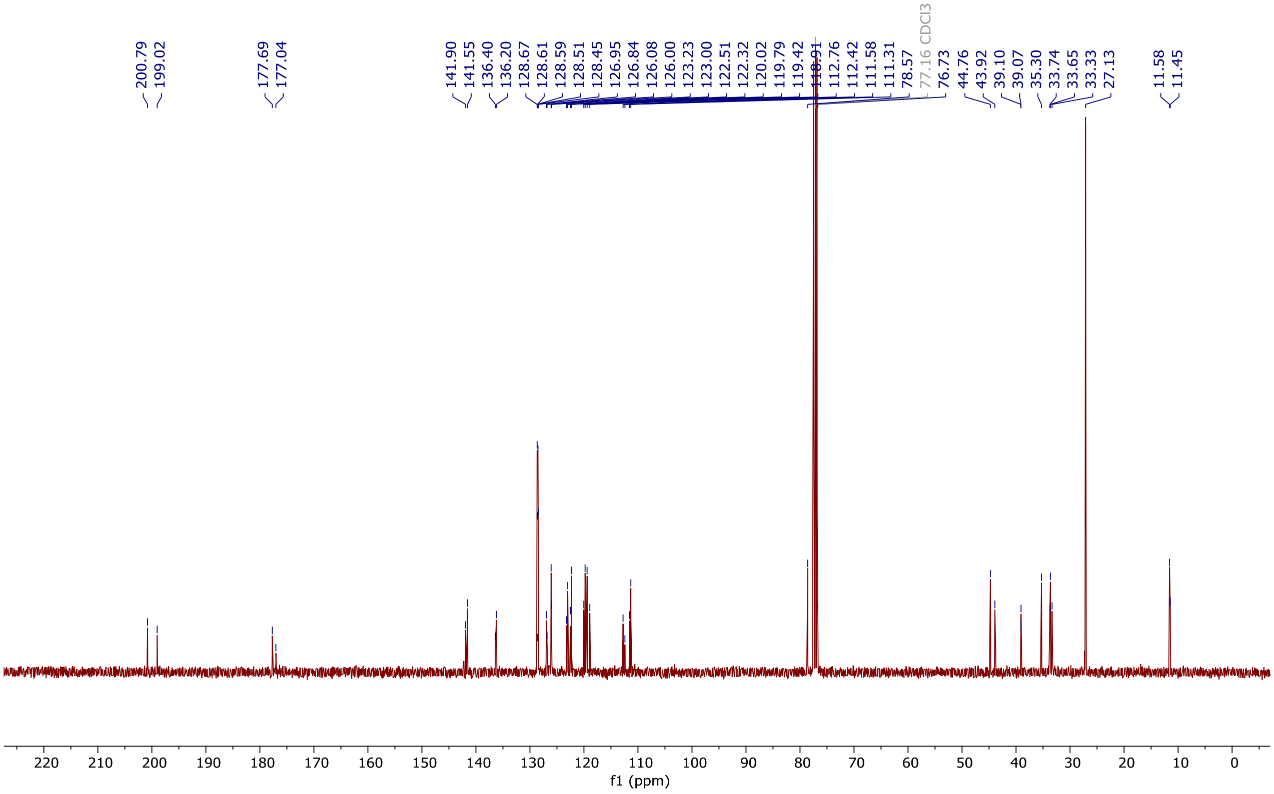

9 ^{1}H NMR (400 MHz, $CDCl_3$) & ^{13}C NMR (101 MHz, $CDCl_3$):

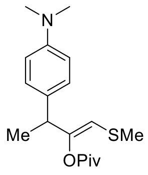

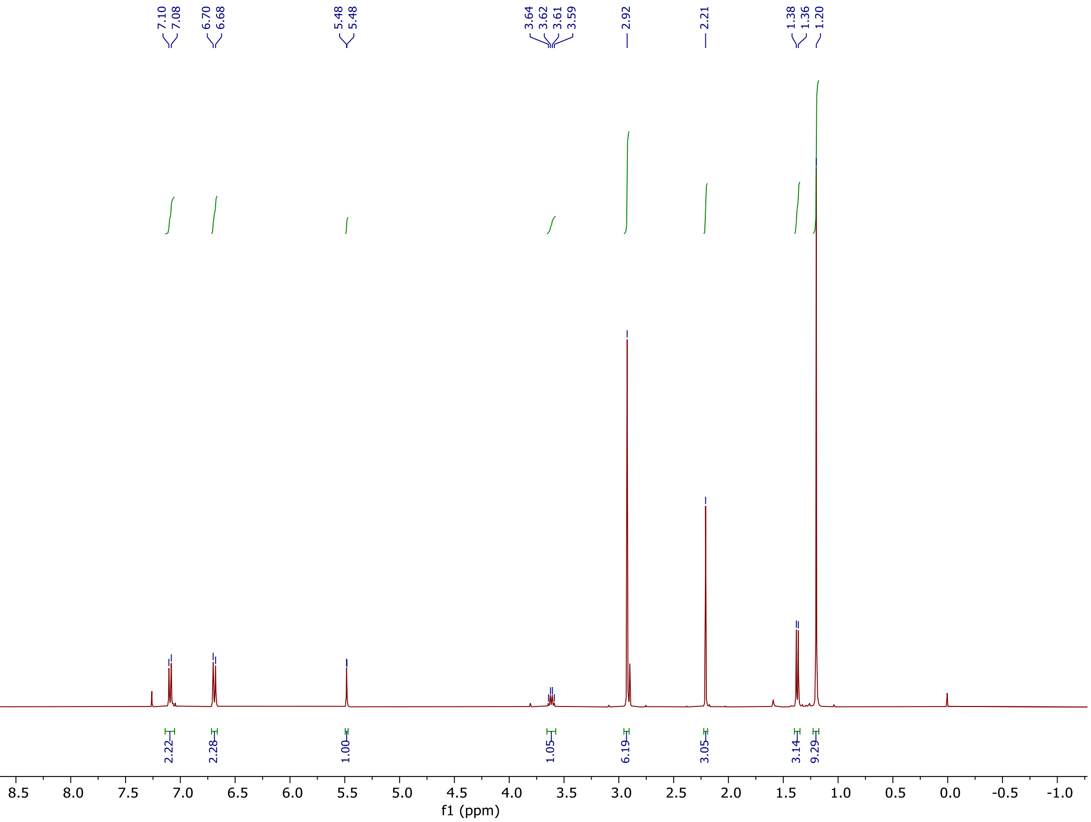

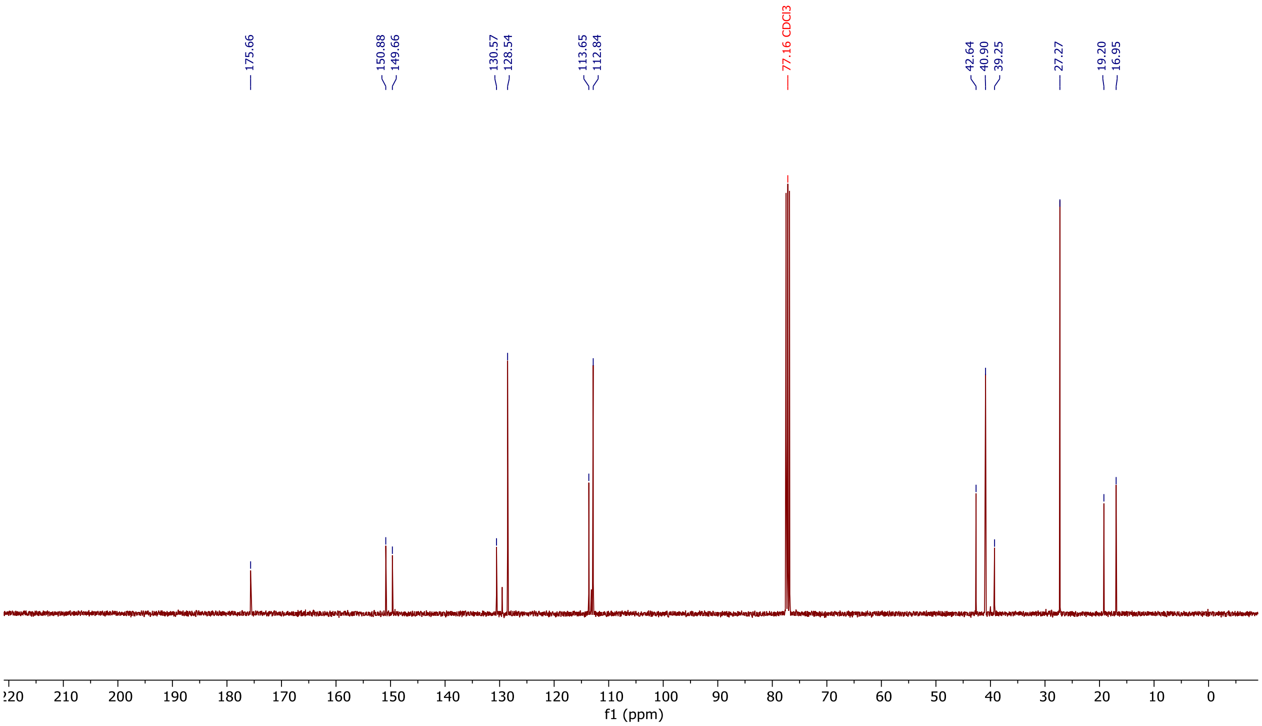

References

[1] Ju, C.; Meng, C.; Ma, J.; Zang, X.; Ding, S. *Chem. Commun*, **2020**, *56*, 3955–3958.

[2] Izquierdo, F.; Manzini, S.; Nolan, S. P. *Chem. Commun.*, **2014**, *23*, 14926–4937

[3] Zheng, W.; Zheng, F.; Hong, Y.; Hu, L. *Heteroat. Chem.* **2012**, *23*, 105–110.

[4] CrysAlisPro, Rigaku Oxford Diffraction, 2015.

[5] G. M. Sheldrick, *Acta Cryst.*, **2015**, *A71*, 3–8.

[6] G. M. Sheldrick, *Acta Cryst.* **2015**, *C71*, 3–8.

[7] O. V. Dolomanov, L. J. Bourhis, R. J. Gildea, J. A. K. Howard, H. Puschmann, *J. Appl. Crystallogr.* **2009**, *42*, 339–341.
